# A single intra-articular injection of 2.0% non-chemically modified sodium hyaluronate vs 0.8% hylan G-F 20 in the treatment of symptomatic knee osteoarthritis: A 6-month, multicenter, randomized, controlled non-inferiority trial

**DOI:** 10.1371/journal.pone.0226007

**Published:** 2019-12-10

**Authors:** Emmanuel Maheu, Bernard Avouac, Renée Liliane Dreiser, Thomas Bardin

**Affiliations:** 1 Department of Rheumatology, AP-HP, Hôpital Saint-Antoine, Paris, France, Private office, Paris, France; 2 Department of Rheumatology, CHU Henri Mondor, Créteil, France (currently: rheumatologist, consultant, Paris, France); 3 Department of Rheumatology, AP-HP, Hôpital Bichat-Claude Bernard, Paris, France; 4 Department of Rheumatology, AP-HP, Hôpital Lariboisière, Université Paris Diderot, Paris, France; Public Library of Science, UNITED KINGDOM

## Abstract

**Objectives:**

The aim of the study was to demonstrate the non-inferiority of a single intra-articular injection of 2.0% non-chemically modified sodium hyaluronate (SH) vs 0.8% hylan G-F 20 (control) in symptomatic knee osteoarthritis.

**Design:**

This was a double-blind, randomized, controlled trial conducted in patients with painful tibiofemoral osteoarthritis (American College of Rheumatology criteria) with insufficient response or intolerance to first-line analgesics and regular non-steroidal anti-inflammatory drugs. Subjects received a single intra-articular injection of either SH or hylan G-F 20. The primary outcome was the 6-month change from baseline in the Western Ontario and McMaster Universities Osteoarthritis Index pain subscale (WOMAC A), with a pre-specified lower margin for non-inferiority of 8 mm.

**Results:**

Of the 292 patients randomized (SH: 144), 288 received an injection (SH: 142), 266 completed the study (SH: 134). In the Per Protocol dataset (SH: 113, control: 112), the WOMAC A change at 6 months was -34.3 mm (95% confidence interval (CI): -37.8, -30.8) and -36.2 mm (95% CI: -40.3, -32.1) for the SH and hylan G-F 20 patients, respectively (*P* = 0.5). The intergroup difference was -1.9 mm (95% CI: -7.3, 3.5). Results were similar in the Full Analysis Set (SH: 139, control: 141) with a difference between the groups of -2.9 mm (95% CI: -7.9, 2.2).

A total of 31.3% of the injected patients reported a treatment-emergent adverse event, including injection site reactions (pain, inflammation or effusion) which occurred in 8.5% of the SH patients vs 13.0% of the hylan G-F 20 patients. No serious reactions were reported.

**Conclusions:**

This clinical trial demonstrated the non-inferiority of a single intra-articular injection of SH vs hylan G-F 20 on the WOMAC A change from baseline at 6 months.

## Introduction

Osteoarthritis (OA) is a degenerative disease mainly affecting the knee joints [1–3]. It highly restricts the ability of patients to perform their activities of daily living [3,4] and could be responsible for an excess mortality (standardized mortality ratio 1.55), walking disability being the main risk factor associated with this increased risk of death [5]. Although OA is considered as a major public health problem and its optimal management is critical for reducing the burden of illness in developed countries [6,7], there is still no well-established disease- nor structure-modifying therapy for this condition. Therefore, current treatment strategies for knee OA aim at relieving symptoms and are based on a combination of non-pharmacologic and pharmacologic treatments, as recommended by various scientific societies [8–11].

Intra-articular hyaluronic acid (HA) injections have been introduced in the therapeutic armamentarium for managing OA since the 80’s and are widely used in clinical practice, especially in the treatment of knee OA [12,13]. Despite some discrepancies, recent meta-analyses and systematic reviews advocate a moderate but true effect of intra-articular HA in relieving pain and improving joint function in knee OA, which lasts for up to six months post injection, i.e., much longer than intra-articular corticosteroid injections [14–27]. Intra-articular HA even seems to offer the best benefit-risk balance among pharmacologic treatments of knee OA [13,20,26,28] and is therefore recommended by the European Society for Clinical and Economic Aspects of Osteoporosis, Osteoarthritis and Musculoskeletal Diseases (ESCEO) as second-line treatment in patients with persistent symptomatic OA [13]. The American College of Rheumatology (ACR) also considers it as an alternative to unsatisfactory initial pharmacologic therapy, emphasizing its interest for patients older than 75 years who are not recommended to take oral non-steroidal anti-inflammatory drugs (NSAIDs) [9]. Finally, the Osteoarthritis Research Society International (OARSI) recommends intra-articular HA injections only after determining whether it can have merit in the context of the individual patient characteristics, comorbidities and preferences, based on an uncertain appropriateness [10].

HA is an ubiquitarious glycosaminoglycan, especially present in human synovial fluid, synovial membrane and cartilage. In OA, the rheological properties of the synovial fluid are reduced due to a decreased HA concentration and an increased amount of low molecular weight (MW) HA compared with healthy joints [29]. HA is injected into the affected joint to improve the viscoelasticity of OA synovial fluid and therefore to restore its lubricating and shock-absorbing properties. As the residence time of exogenous HA in the joint is not superior to 7 days, the prolonged effects lasting several months post injection suggest that other mechanisms of action must be at work [30]. Intra-articular HA has been found to promote proteoglycan and glycosaminoglycan synthesis within the cartilage (including endogenous HA), to decrease cartilage catabolic activities, to reduce chondrocyte apoptosis while increasing their proliferation, to suppress pro-inflammatory mediators, and to inhibit the action of pain mediators [31].

Currently marketed intra-articular HA preparations indicated for the treatment of OA may have differences in product characteristics such as in the posology, the injected volume, or the HA origin, structure (i.e., crosslinking degree), concentration, and MW. There is still no consensus on the clinical impact of each of these parameters at present. Most intra-articular HA preparations available today are based on a multiple injection dosing regimen. Over the last few years, the so called “one-shot” HAs have been developed, which are increasingly used worldwide. Most of the one-shot preparations contain crosslinked HAs, which are known to have an increased residence time within the joint in comparison with non-chemically modified HAs. In this randomized, controlled trial, we aimed at demonstrating the non-inferiority of a single intra-articular injection of 2.0% non-chemically modified intermediate MW sodium hyaluronate (SH) containing 0.5% of mannitol compared with a single intra-articular injection of 0.8% hylan G-F 20 in patients with symptomatic tibiofemoral OA at six months after treatment. One-shot hylan G-F 20 was chosen as comparator, since it has demonstrated a clinical superiority over intra-articular placebo in patients with knee OA [32].

## Patients and methods

### Ethics and registration

The trial was conducted in accordance with Good Clinical Practices, complied with the principles of the 2008 revised Declaration of Helsinki, and is reported according to the CONSORT guidelines (S1 Checklist). Ethical approval was obtained on 9 June 2011 from the Institutional Review Board of Robert Ballanger Hospital (Aulnay-sous-Bois, France). Patients were recruited from 21 June 2011 to 30 April 2012, with the last patient completing the study on 22 November 2012. The study was registered with the French Competent Authority (ID-RCB 2011-A00258-33) before it started, and in ClinicalTrials.gov (NCT03203408) after participant enrolment. The reason for this delay is due to an oversight on the part of the study sponsor, who rectified it afterward. We confirm that all ongoing and related trials evaluating a single intra-articular injection of 2.0% non-chemically modified SH (Ostenil^®^ Plus) are registered.

No major change to the protocol (S1 and S2 Protocols) were implemented after the study start. Some corrections had to be made to the frozen database on one occasion after unblinding the study (S1 Appendix). The procedure was duly recorded and approved by the Scientific Board of the study.

### Trial design

This was a multicenter, double-blind, randomized, active-controlled, non-inferiority trial with a two-arm parallel design. Patients eligible at screening entered a washout period for analgesics and NSAIDs lasting 2 to 5 days, depending on the drug. They were consecutively randomized at baseline (D0) to receive either a single intra-articular injection of SH (test product) or a single intra-articular injection of hylan G-F 20 (control) within 2 days after randomization. Patients were then examined for efficacy and safety at 30, 90 and 180 days (D30, D90, D180). Patients who prematurely discontinued were not replaced.

### Patients

Participants were recruited in private medical practices in France. Eligible patients were men and women (aged 40 to 85 years) diagnosed with primary knee OA according to the ACR criteria [33] and a modified Kellgren-Lawrence grade Ib-III (X-ray taken in the past 12 months), i.e., radiographically defined joint space narrowing between 25% and 75% and definite osteophyte [34]. Only patients with insufficient response or intolerance to first-line analgesics and regular NSAIDs were included. In addition, they should have knee pain on ≥15 days in the previous month and a pain level ≥40 mm on a 100 mm normalized Western Ontario and McMaster Universities Osteoarthritis Index pain subscale (WOMAC A) [35]. In case of bilateral knee OA, patients should also present a difference of ≥20 mm on WOMAC A between the studied and contralateral knees. Participants should have a health insurance and be able to understand the study instructions. Their written informed consent was collected prior to enter the study.

Subjects were excluded if their knee OA resulted from a trauma or was predominantly of patellofemoral origin. Further exclusion criteria comprised excessive varus/valgus knee deformity (≥15 degrees on a standard X-ray), inflammatory/metabolic rheumatic diseases, obesity (body mass index ≥30 kg/m^2^), history of injury to the studied knee in the past six months, lower limb pain other than knee OA, and severe disease likely to interfere with trial assessments. Patients with the following treatments were also excluded: intra-articular HA injection in the studied knee within the previous six months, intra-articular corticosteroid injection in the studied knee within the previous two months, symptomatic slow-acting drug in OA (SYSADOA) initiated or modified within the previous three months, any surgical intervention (including arthroscopy) within the past year, any surgery scheduled during the study. Finally several exclusion criteria were established to comply with the contra-indications and warnings associated with the study products: important joint effusion (requiring knee puncture), wound or skin disease on/around the studied knee, anticoagulant therapy with heparin or warfarin, known hypersensitivity to HA, avian proteins, mannitol or acetaminophen, pregnancy, breast-feeding.

### Treatment and blinding

The test product was Ostenil^®^ Plus (TRB Chemedica AG, Haar/Munich, Germany), a preparation containing 2.0% of a non-chemically modified intermediate MW (range 1 000–2 000 kDa) SH obtained by biofermentation and 0.5% mannitol. It is a 2.0 ml viscoelastic solution presented in a 3 ml pre-filled syringe. The comparator was Synvisc-One^®^ (Genzyme Biosurgery, Ridgefield, NJ), a preparation containing 0.8% of hylan G-F 20, an avian derived divinylsulfone crosslinked HA gel (80% hylan A + 20% hylan B) with a mean MW of 6 000 kDa. It is provided as a 6 ml viscoelastic fluid in a 10 ml pre-filled syringe. Both investigational products were packed in identical neutral packaging and labeled according to Good Manufacturing Practices for the manufacture of investigational medicinal products and in compliance with national regulations. Since the SH and hylan G-F 20 preparations differ in appearance (viscoelasticity, volume and syringe) and were identifiable by the injecting investigator, double-blind conditions were ensured by the intervention of an observer blinded to treatment (evaluating investigator), who had to perform clinical assessments at screening, baseline and post injection follow-up visits. Patient blinding was ensured by avoiding visual access to the injection field. Injections were performed according to the usual rules of asepsis, using a lateral patellofemoral approach, as commonly recommended [12].

Treatments that could interfere with the assessment of knee OA symptoms, including joint lavage and intra-articular injections of corticosteroids or other HA, were prohibited during the entire study. A knee effusion removal was tolerated only in case of severe joint effusion. The use of oral corticosteroids and topical or oral NSAIDs was restricted to short periods of time (3–5 days/month), provided that the patient fulfill the washout period before each assessment visit; anti-aggregant low dose Aspirin^®^ was permitted (<325 mg/day) during the trial. Rescue analgesic was acetaminophen up to 4 g/day with a 12-hour washout period before each follow-up visit.

### Randomization and treatment allocation

Patients were randomly assigned to one of the two treatment groups on a 1:1 ratio. A randomization list in blocks of four treatments, without stratification, generated by a computer algorithm, was produced by an independent company having no contact with the investigators or personnel involved in the conduct of the study. After verification of the inclusion and exclusion criteria, the evaluating investigator allocated a randomization number according to the chronological order of enrolment of each patient at his/her site. The patient was then sent to the injecting investigator who administered the investigational product corresponding to the randomization number. To maintain the balance between the two groups each evaluating investigator undertook to recruit a multiple of four patients.

### Outcome measures

The primary criterion was the change in WOMAC A between baseline and D180, using the visual analog scale (VAS) (version VA3.1) [35]. Changes were compared between groups at endpoint. Stiffness (WOMAC B) and function (WOMAC C) subscales of the WOMAC were assessed as secondary criteria. Results of each domain were normalized on a 0–100 mm scale. Other secondary efficacy parameters included the Lequesne index [36], patient global assessment of disease activity (PtGA) on a 100 mm VAS, global treatment efficacy evaluated by both the patient and the investigator (5-point Likert scale), and acetaminophen consumption. All these parameters were assessed at each study visit. In addition, the responder rate according to the Outcome Measures in Rheumatoid Arthritis Clinical Trials (OMERACT) group and the OARSI criteria was calculated at D180 [37].

All adverse events (AEs) and serious adverse events (SAEs) were collected from screening throughout the study. Local reactions such as site injection joint pain, joint effusion and acute pseudo-septic or septic arthritis were recorded at the first follow-up visit (D30).

### Statistics

The sample size was calculated on the primary outcome, based on an acceptable margin for non-inferiority between the treatment groups predefined at 8 mm (Δ) on a 100 mm VAS, i.e., less than the minimum perceptible clinical improvement of 10 mm usually considered for the WOMAC A [38], and as previously chosen in two similar non-inferiority trials [39,40]. Assuming a standard deviation (SD) of 24 mm for both groups and a type I error risk of 5% (one-sided), it was estimated that 111 assessable patients per group would be necessary to achieve an 80% power. Anticipating 15% of major protocol violations in addition to a 15% discontinuation rate at six months, it was decided to enroll a total of at least 290 patients.

Three efficacy datasets were defined in the Statistical Analysis Plan. The Intention-to-Treat (ITT) population comprising all patients included and randomized in the study was used for the analysis of demographic and baseline characteristics. The Full Analysis Set (FAS) was a subgroup of the ITT consisting of patients who received the investigational product and for whom at least one post injection evaluation of the primary efficacy criterion was available. Finally, the Per Protocol (PP) population was a subgroup of patients with no major protocol deviation selected from the FAS in a blinded fashion prior to breaking the randomization codes.

The primary statistical analysis was performed on the PP dataset. To ensure the robustness of the results a secondary analysis was also performed on the observed data of the FAS. Furthermore, to evaluate the potential risk of bias due to missing data, a multiple imputation approach was used to replace missing WOMAC A values at D180, allowing the primary efficacy outcome to be calculated for all patients of the FAS and compared with the one obtained from the analysis on observed data. Firstly, a comparative analysis of discontinued/lost to follow-up patients vs completers was performed to identify any covariates for which major differences had been highlighted. Secondly, assuming that missing data were of the missing at random type, multiple imputation based on the available data at D0, D30 and D90 was performed, with a regression model where the treatment and covariates identified earlier were included. Twenty imputations per case were performed [41]. Finally, sensitivity analyses were carried out to verify the influence of parameters for which the intergroup difference was statistically significant at baseline, using analysis of covariance and multiple regression and/or adjustment depending on the parameters.

Baseline characteristics were analyzed using descriptive statistics, i.e., mean, SD, median, minimum, maximum, or depending on the type of variable by the number of patients and percentage per category. For inferential comparisons between the two treatment arms, Chi-square test or Fisher’s exact test, and Student’s t-test or Wilcoxon-Mann-Whitney test were used, depending on the type and distribution of data. Paired t-test or signed rank test were used for comparisons within the treatment arms over time. For all the tests performed the approach was bilateral and the alpha risk was fixed at 5%.

Stata (College Station, TX) was used to model missing data by multiple imputation. Other statistical analyses were performed using SAS V9.2 (Cary, NC).

## Results

### Patient disposition

**Fig 1:**
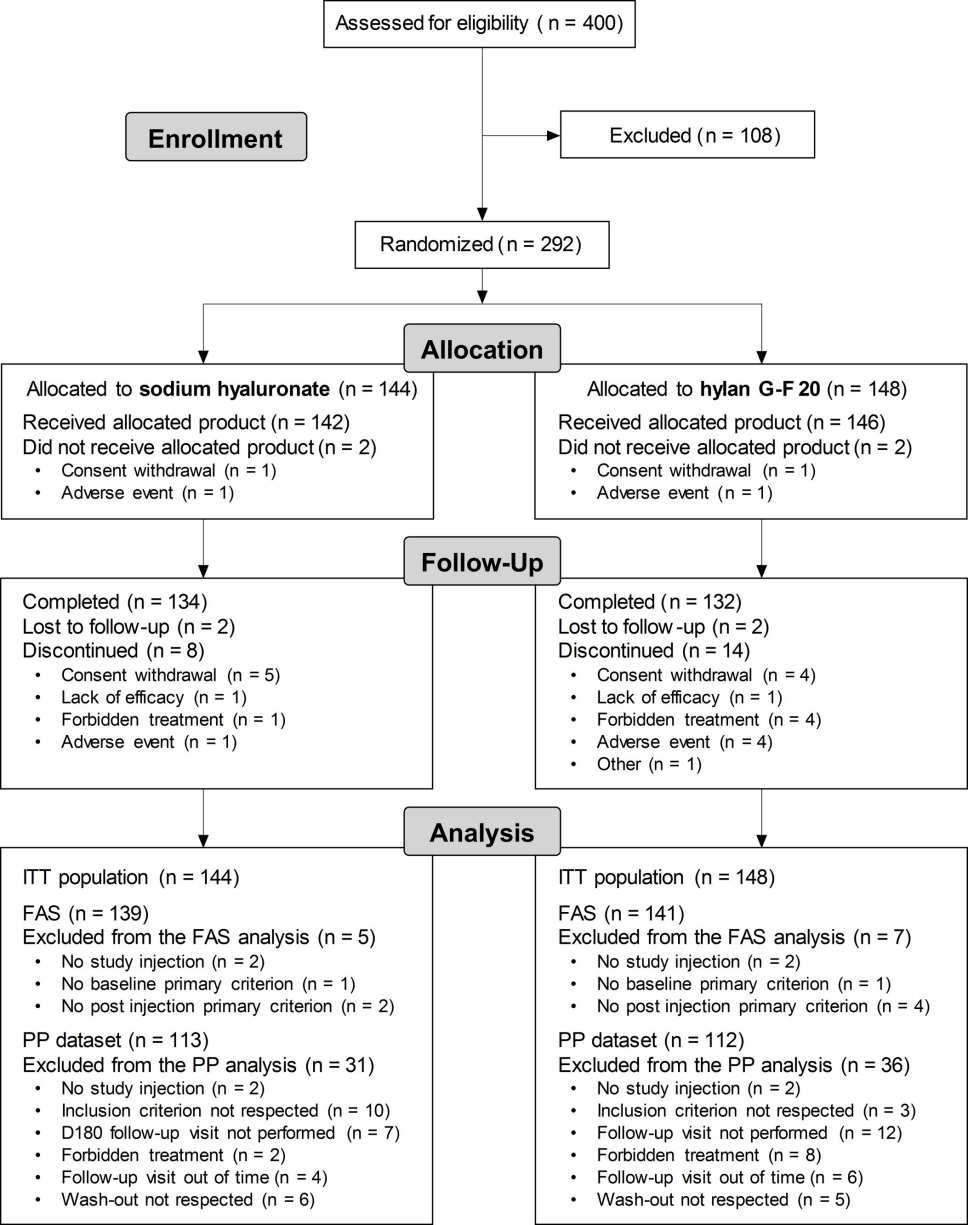
CONSORT flow diagram. FAS = Full Analysis Set; ITT = Intention-to-Treat; n = number of patients; PP = Per Protocol.

Fig 1 presents the CONSORT flow diagram of the trial.

Fifty centers (couples of injecting and blinded evaluating investigators) preselected 400 subjects, among whom 108 (27.0%) were not eligible at screening. Two hundred ninety two patients were included in the study: 144 allocated to the SH group (49.3%) and 148 to the control group (50.7%). Out of the 292 randomly assigned patients (ITT dataset), 288 received a single injection of SH (n = 142, 48.6%) or hylan G-F 20 (n = 146, 50.0%) and 4 (1.4%) did not. The overall dropout rate was 7.5%. Eighteen patients who received the investigational product prematurely discontinued the study (SH: 6, control: 12), and 2 in each group were lost to follow-up (S1 Table). A total of 266 patients (91.1%) completed the trial at 6 months (SH: 134, control: 132).

The FAS (SH: 139, control: 141) comprised all randomized patients but 12 who had no baseline or post injection value for the WOMAC A (8 patients) or who did not receive the study injection (4 patients). Sixty-seven patients with a major protocol violation were excluded from the PP dataset (see details in Fig 1 and S2 Table). As a result, 225 patients completed the study without any major deviation, i.e., 113 in the SH group and 112 in the control group.

### Demographic and other baseline characteristics

Baseline characteristics of the ITT dataset are summarized in Table 1. Most patients were women (66.4%), moderately overweight (body mass index 26.4 ± 2.9 kg/m^2^) with a mean age of 66.9 ± 10.2 years (39–86 years old). About 56.5% of patients had a knee OA severity grade II according to the modified Kellgren-Lawrence radiographic scoring. Knee OA was unilateral in half of the patients; most of them had a unicompartmental tibiofemoral OA (67.5%) without associated patellofemoral pain syndrome (75.0%). Symptoms were moderate to severe, with mean pain (58.3 ± 11.7 mm), stiffness (48.3 ± 20.3 mm) and function (47.7 ± 15.8 mm) WOMAC subscores of the studied knee and a Lequesne index score of 12.0 ± 6.7 points. Both groups had very similar baseline demographic, disease and symptoms characteristics; the only significant difference observed was the male/female ratio (*P* = 0.04) (SH: 72.2% women; control: 60.8% women).

**Table 1:** Patient demographics and baseline characteristics (Intention-to-Treat population).

Characteristic	SH n = 144	Control n = 148	*P*
Female, n (%)	104 (72.2)	90 (60.8)	0.04†
Age (years), mean (SD)	67.1 (9.7)	66.6 (10.7)	0.7‡
Body mass index (kg/m^2^), mean (SD)	26.4 (3.0)	26.3 (2.9)	0.8‡
Bilateral knee osteoarthritis, n (%)	70 (48.6)	76 (51.4)	0.6†
Studied knee (right), n (%)	85 (59.0)	74 (50.0)	0.1†
Bicompartmental knee osteoarthritis, n (%)	41 (28.5)	51 (34.5)	0.5†
Associated patellofemoral pain syndrome, n (%)	32 (22.2)	41 (27.7)	0.3†
Time since knee osteoarthritis diagnosis, n (%)			0.8†
<1 year	10 (6.9)	11 (7.4)	
≥1 and <5 years	58 (40.3)	51 (34.5)	
≥5 and <10 years	39 (27.1)	44 (29.7)	
≥10 years	37 (25.7)	42 (28.4)	
Modified Kellgren-Lawrence grade at studied knee			0.3†
Grade Ib	21 (14.6)	26 (17.6)	
Grade II	78 (54.2)	87 (58.8)	
Grade III	45 (31.3)	35 (23.6)	
WOMAC A (mm), mean (SD)	58.4 (11.5)	58.3 (12.0)	0.9‡
WOMAC B (mm), mean (SD)	48.0 (20.7)	48.5 (20.0)	0.8‡
WOMAC C (mm), mean (SD)	47.1 (16.0)	48.4 (15.6)	0.5‡
Lequesne index, mean (SD)	11.7 (3.5)	11.6 (3.5)	0.8‡
PtGA (mm), mean (SD)	59.6 (16.2)	59.9 (17.9)	0.9‡

† Chi-square test

‡ Student’s t-test.

Control = hylan G-F 20; PtGA = patient global assessment of disease activity; SD = standard deviation; SH = sodium hyaluronate; WOMAC A, B, C = Western Ontario and McMaster Universities Osteoarthritis Index pain, stiffness, function subscales.

All baseline characteristics were similar in the FAS (S3 Table) and PP datasets (S4 Table). Individual data are presented in S5 Table.

### Primary efficacy outcome

**Fig 2:**
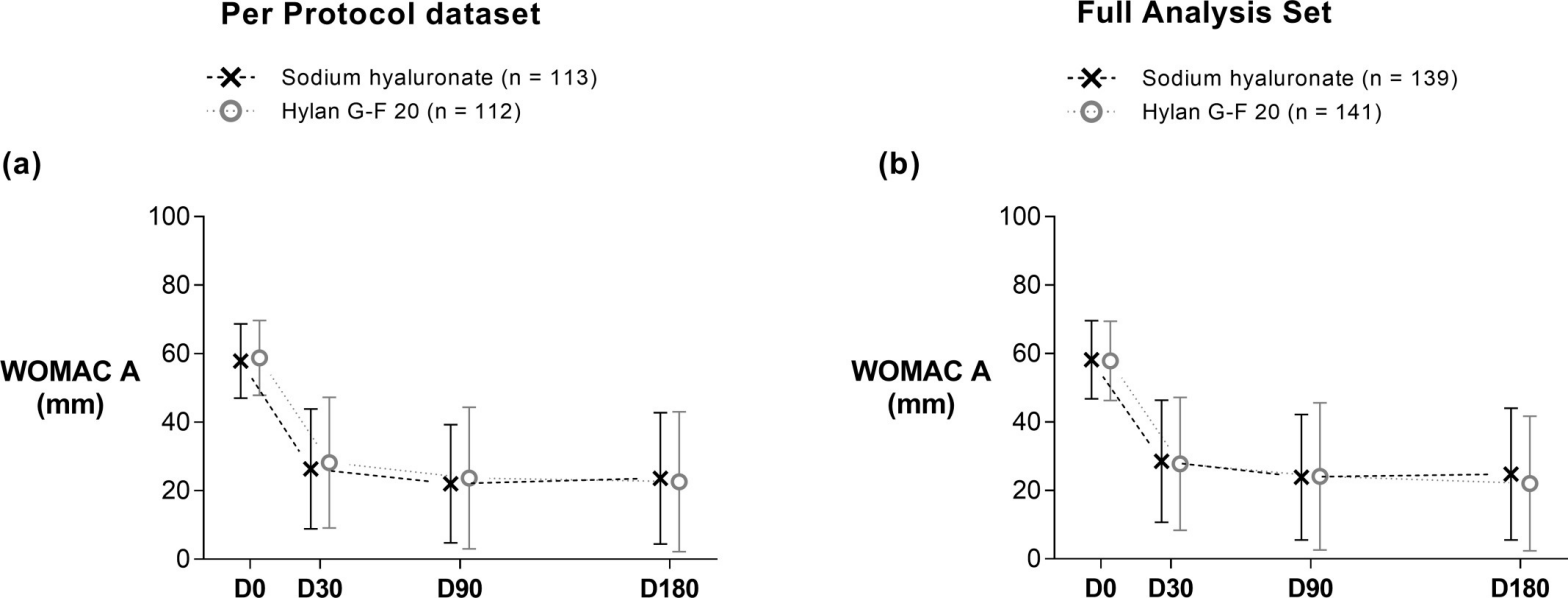
Evolution of the WOMAC pain subscale during the 6-month trial. The two graphs represent data of the Per Protocol dataset (a) and Full Analysis Set (b). They are presented as means of observed cases ± standard deviation. Sodium hyaluronate group: saltire with dashed line; control group: hollow circle with dotted line; n = number of patients; WOMAC A = Western Ontario and McMaster Universities Osteoarthritis Index pain subscale.

In the PP dataset, both treatment groups showed a marked improvement in pain at D30 compared with baseline (*P* <0.001), which was maintained for the entire study duration (Fig 2A). At D180, the mean WOMAC A had decreased by 34.3 mm (95% CI: -37.8, -30.8) in the SH group and 36.2 mm (95% CI: -40.3, -32.1) in the control group compared with baseline (Table 2), with no significant difference between both treatment groups (*P* = 0.5). Similar results were obtained in the FAS (Fig 2B, S6 Table). Individual efficacy data for the WOMAC A are displayed in S7 Table.

**Table 2:** Primary and secondary efficacy criteria at 6 months post injection (Per Protocol dataset).

Outcome	SH n = 113	Control n = 112	Difference	*P*
WOMAC A change from baseline (mm)				
Mean (SE)	-34.3 (1.8)	-36.2 (2.1)	-1.9 (2.7)	0.5†
95% CI	(-37.8, -30.8)	(-40.3, -32.1)	(-7.3, 3.5)	
WOMAC B change from baseline (mm)				
Mean (SE)	-22.5 (2.3)	-26.9 (2.1)	-4.4 (3.1)	0.2†
95% CI	(-27.0, -18.0)	(-31.1, -22.8)	(-10.5, 1.7)	
WOMAC C change from baseline (mm)				
Mean (SE)	-21.3 (1.6)	-25.7 (1.9)	-4.4 (2.5)	0.08†
95% CI	(-24.4, -18.1)	(-29.5, -21.8)	(-9.4, 0.5)	
Lequesne index change from baseline				
Mean (SE)	-4.3 (0.3)	-4.7 (0.4)	-0.4 (0.5)	0.4†
95% CI	(-4.9, -3.6)	(-5.4, -4.0)	(-1.4, 0.6)	
PtGA change from baseline (mm)				
Mean (SE)	-25.4 (2.2)	-27.0 (2.0)	-1.5 (2.9)	0.6†
95% CI	(-29.7, -21.1)	(-30.9, -23.0)	(-7.3, 4.3)	
Global treatment efficacy (good/very good), n (%)				
By the patient	75 (66.4)	75 (67.0)		>0.9‡
By the investigator	78 (69.0)	77 (68.8)		>0.9‡
Acetaminophen use post injection (yes), n (%)	82 (72.6)	74 (66.1)		0.3‡
OMERACT-OARSI responders, n (%)	93 (83.0)	96 (85.7)		0.6§

† Student’s t-test

‡ Fisher’s exact test

§ Chi-square test.

CI = confidence interval; OMERACT-OARSI = Outcome Measures in Rheumatoid Arthritis Clinical Trials—Osteoarthritis Research Society International; PtGA = patient global assessment of disease activity; SE = standard error; SH = sodium hyaluronate; WOMAC A, B, C = Western Ontario and McMaster Universities Osteoarthritis Index pain, stiffness, function subscales, respectively.

**Fig 3:**
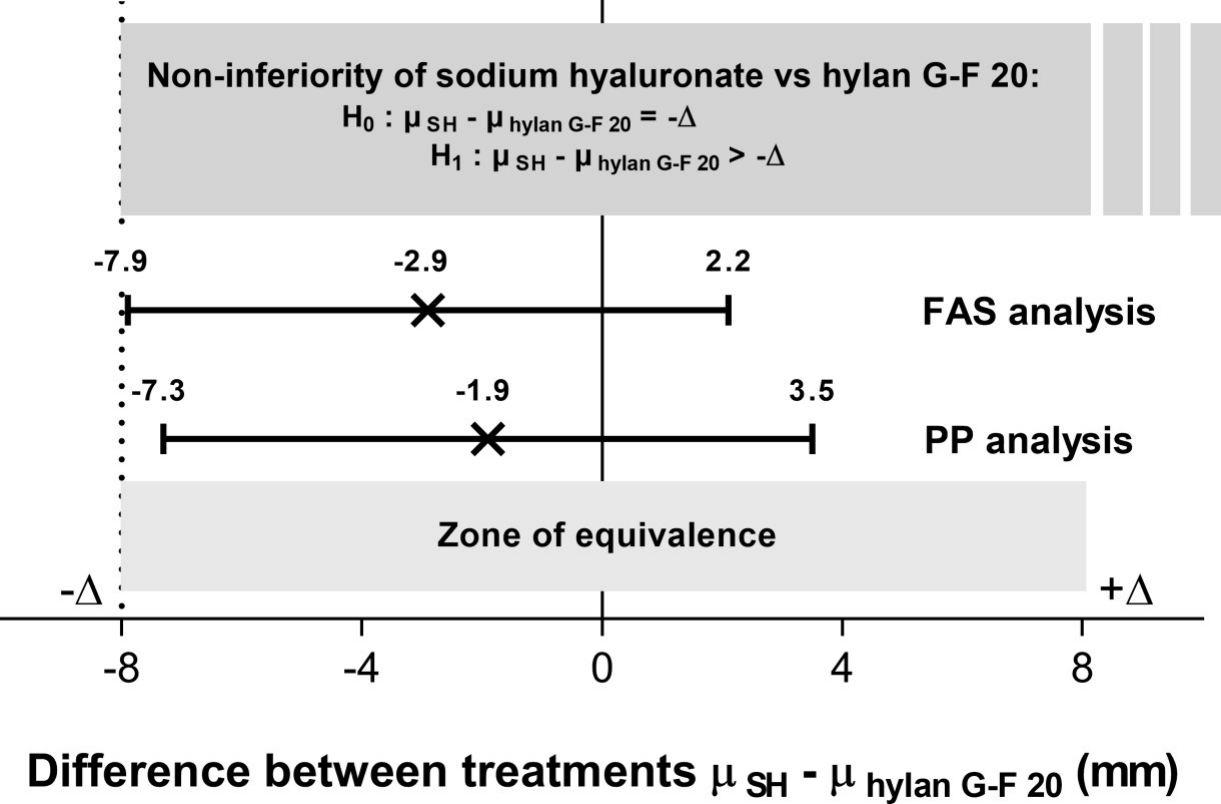
Non-inferiority of sodium hyaluronate vs hylan G-F 20 in the Per Protocol dataset and Full Analysis Set. Point estimates and 95% confidence interval of the intergroup difference in the primary efficacy endpoint (change from baseline in WOMAC A at 6 months). FAS = Full Analysis Set; PP = Per Protocol dataset; WOMAC A = Western Ontario and McMaster Universities Osteoarthritis Index pain subscale.

The intergroup difference for the primary efficacy endpoint was -1.9 mm (95% CI: -7.3, 3.5; *P* = 0.5) in the PP dataset (Fig 3). The lower limit of the 95% CI was higher than the predefined non-inferiority margin (-Δ) of -8 mm, demonstrating the non-inferiority of SH vs hylan G-F 20 on efficacy. In addition, the 95% CI of the intergroup difference lay within the equivalence zone from -8 mm to +8 mm allowing to conclude to an equivalence between SH and hylan G-F 20. Sensitivity analysis on the FAS showed similar results with an intergroup difference in the WOMAC A change at -2.9 mm (95% CI: -7.9, 2.2) (*P* = 0.3). These outcomes were confirmed after multiple imputation of all missing data (8 in each group) using the treatment, sex and age of the patient as covariates (estimated intergroup difference: -2.3 mm, 95% CI: -7.3, 2.7). Finally, as sex ratio was unbalanced between groups at baseline, an analysis conducted to assess whether this difference could impact the primary efficacy outcome showed no difference between men and women on WOMAC A changes and no significant interaction between sex and intergroup difference of efficacy (*P* >0.9) (S2 Appendix).

### Secondary efficacy outcomes

**Fig 4:**
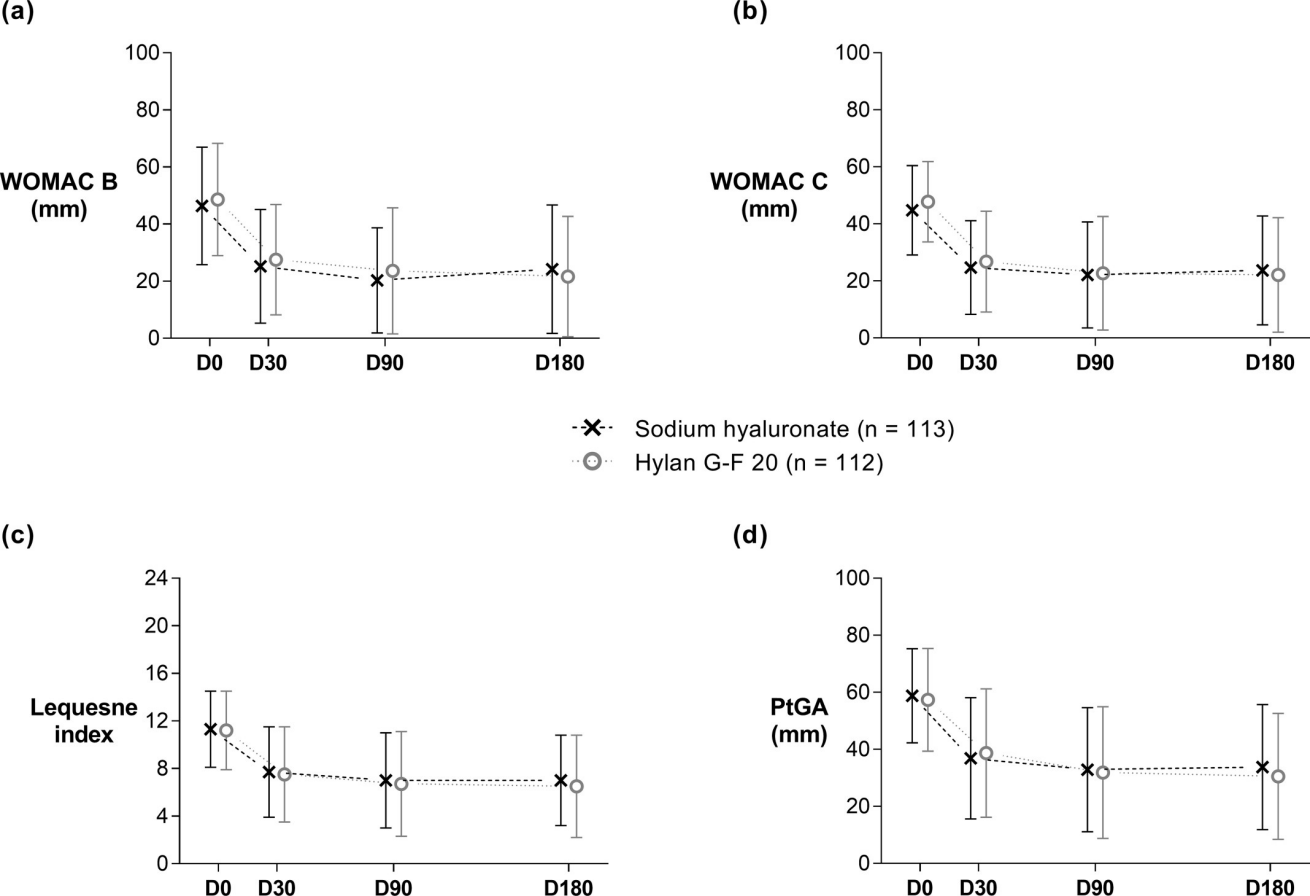
Evolution of the secondary efficacy criteria during the 6-month trial (Per Protocol dataset). The four graphs represent WOMAC stiffness subscale (a), WOMAC function subscale (b), Lequesne index (c) and patient global assessment of disease activity (d). Data are presented as means of observed cases ± standard deviation. Sodium hyaluronate: saltire with dashed line; control group: hollow circle with dotted line; PtGA = patient global assessment of disease activity; WOMAC B, C = Western Ontario and McMaster Universities Osteoarthritis Index stiffness, function subscales.

In the PP dataset, both groups showed a significant improvement in stiffness and function after treatment (*P* <0.001), which was maintained up to the end of the study (Fig 4A and 4B, Table 2). Similar figures could be observed for the Lequesne index and PtGA (Fig 4C and 4D, Table 2). The Lequesne index score decreased by 4.3 (95% CI: -4.9, -3.6) in the SH group and 4.7 (95% CI: -5.4, -4.0) in the control group (*P* = 0.4) at D180 (Table 2). Global treatment efficacy evaluated by the patient and the investigator were also similar in both groups, 66.4% of patients in the SH group considering their treatment as good to very good vs 67.0% of patients in the control group (*P* >0.9). Moreover, the OMERACT/OARSI responder rates at D180 were high: 83.0% in the SH group and 85.7% in the control group (*P* = 0.6). Similar proportions of patients in both groups took rescue medication during the study (*P* = 0.4): 82 in the SH group (72.6%) and 74 (66.1%) in the control group at a low daily mean dosage.

Secondary efficacy results in the FAS were consistent with those obtained in the PP dataset (S6 Table, S1 Fig). Individual secondary efficacy data are displayed in S8, S9, S10, S11, S12 and S13 Tables.

### Safety outcomes

Only 288 patients received the allocated injection and were considered for safety outcomes (Table 3). Overall, both intra-articular treatments were well tolerated. The proportions of patients reporting a treatment-emergent AE were similar: 39 (27.5%) vs 51 (34.9%) in the SH and control groups, respectively. Respectively 60 and 74 AEs were reported in the SH and control groups (*P* = 0.2), most of them being of mild or moderate intensity. Their distribution per frequency and type was similar in both groups (S14 and S15 Tables). All patients recovered from their AEs.

**Table 3:** Safety outcomes in the patients who received an injection of the investigational product.

Outcome, n (%)	SH n = 142	Control n = 146	*P*
Patients with at least one treatment-emergent AE	39 (27.5)	51 (34.9)	0.2†
Treatment-emergent AEs§	60 (42.3)	74 (50.7)	0.2‡
Patients with at least one SAE	1 (0.7)	2 (1.4)	>0.9†
SAEs§	2 (1.4)	2 (1.4)	0.6‡
Dropouts due to an AE	0 (0.0)	3 (2.1)	0.2†
Patients with at least one injection site reaction	12 (8.5)	19 (13.0)	0.3†
Injection site reactions§	17 (12.0)	25 (17.1)	0.2‡
Treatment-emergent AEs >2%§			
Injection site joint pain§	11 (7.7)	16 (11.0)	0.4‡
Arthralgia of the studied knee§	11 (7.7)	9 (6.2)	0.6‡
Injection site joint inflammation§	4 (2.8)	6 (4.1)	0.6‡
Loin pain§	4 (2.8)	6 (4.1)	0.6‡
Osteoarthritis flare up of the studied knee§	1 (0.7)	5 (3.4)	0.1‡
Bronchitis§	3 (2.1)	3 (2.1)	>0.9‡
Injection site joint effusion§	2 (1.4)	3 (2.1)	>0.9‡
Spinal osteoarthritis§	1 (0.7)	3 (2.1)	0.3‡

† Fisher’s exact test

‡ Wilcoxon-Mann-Whitney test.

§ Percentages indicates incidences, i.e., number of occurrences per total of patients.

AE = adverse event; SAE = serious adverse event; SH = sodium hyaluronate.

The most common AEs were injection site joint pain (n = 27) and pain in the studied knee (n = 20), followed by injection site joint inflammation (n = 10), loin pain (n = 10), and OA flare (n = 6). In total 8.5% of the patients presented a local reaction after injection in the SH group vs 13.0% in the hylan G-F 20 group (*P* = 0.3). They resolved spontaneously in most of the cases; only one fluid removal in a patient of the control group had to be performed. No acute pseudo-septic or septic arthritis were reported.

Only 3 patients were affected by an SAE. One in the SH group fell in the street at 20 days post injection with femoral fracture requiring surgery. In the control group, 1 patient had to undergo total knee replacement of the studied knee at 197 days post injection, another was hospitalized due to fever of viral origin at 22 days post injection. Due to the type of SAEs and their occurrence long after the injection, a direct relationship with the injected HAs was excluded by the investigator.

Three patients dropped out for safety reasons, all in the hylan G-F 20 group: 1 patient had a painful OA flare in his studied knee 29 days after injection, another had a joint effusion in the contralateral knee at 54 days post injection, and the third patient left the trial due to a viral fever (see the SAE described above).

Individual safety data are presented in S16 and S17 Tables.

## Discussion

We report the results of the first face-to-face double-blind randomized controlled trial having compared two single-injection HA preparations, SH and hylan G-F 20. This trial showed the non-inferiority of a single intra-articular injection of an intermediate MW SH solution containing mannitol compared with a single intra-articular injection of a crosslinked, high MW hylan G-F 20 solution, which had been previously shown to be superior to intra-articular placebo [32] in symptomatic knee OA patients. The results of our study, with a sample well representative of the common knee OA outpatient population, do not support those obtained by Raman et al. (2008) [42] suggesting an earlier and more sustained pain reduction with hylan G-F 20 than SH. On the contrary, all primary and secondary efficacy analyses demonstrated that both study products were equivalent on pain relief. Secondary outcomes were also similar in both groups. The rates of responders according to the OMERACT-OARSI response criteria were high with both intra-articular HA products. The magnitude of effect on pain, functional impairment and PtGA was clinically significant and in line with the estimated effect sizes reported in the meta-analysis by Bannuru et al. (2015) [20] and the systematic review by Xing et al. (2016) [43]. Our results are also consistent with those of published clinical trials, i.e., one superiority trial evaluating Synvisc-One^®^ vs placebo [32] and four non-inferiority trials comparing three-injection products: Structovial^®^ vs Synvisc^®^ [44], Sinovial^®^ vs Synvisc^®^ [39], Ostenil^®^ vs Synvisc^®^ [45], and GO-ON^®^ vs Hyalgan^®^ [40]. The primary results of these studies are summarized in Table 4.

**Table 4:** Summary of changes from baseline in pain at 6 months post treatment in previous published studies.

Pain variable	Study product	Reference product
Chevalier et al. (2010) [32]	Synvisc-One^®^	Placebo
**WOMAC A (0–4 Likert)**		
Mean (SE)	-0.84 (0.06)	-0.69 (0.06)
95% CI	*(-0*.*96*, *-0*.*72)*	*(-0*.*80*, *-0*.*58)*
Maheu et al. (2011) [44]	Structovial^®^	Synvisc^®^
**Global pain (100 mm VAS)**		
Mean (SE)	-38.8 (*2*.*3*)	-37.1 (*2*.*4*)
95% CI	(-43.3, -34.4)	(-41.8, -32.5)
Pavelka and Uebelhart (2011) [39]	Sinovial^®^	Synvisc^®^
**WOMAC A (100 mm VAS)**		
Mean (SE)	-32.5 (NA)	-32.5 (NA)
95% CI	NA	NA
Dreiser et al. (2012) [45]	Ostenil^®^	Synvisc^®^
**WOMAC A (100 mm VAS)**		
Mean (SE)	-28.9 (2.0)	-27.8 (2.4)
95% CI	(-32.8, -24.9)	(-32.5, 23.1)
Berenbaum et al. (2012) [40]	GO-ON^®^	Hyalgan^®^
**WOMAC A (100 mm VAS)**		
Mean (SE)	-22.9 (1.4)	-18.4 (1.5)
95% CI	(-25.7, -20.1)	(-21.3, -15.5)

*Italic*: calculated data. NA = not available; SE = standard error; VAS; visual analog scale; WOMAC A = Western Ontario and McMaster Universities Osteoarthritis Index pain subscale.

From a safety point of view, our clinical trial showed that a single intra-articular injection of SH or hylan G-F 20 was well tolerated, treatment-related AEs being mostly mild to moderate local post injection reactions and only few OA flares of the studied knee. No cases of acute pseudo-septic or septic arthritis occurred.

The main limitation of our trial is the absence of a placebo arm that could have been useful to discriminate between the effect resulting from the injection itself and that of the HA preparation. We decided to demonstrate the non-inferiority of SH vs hylan G-F 20 instead of superiority vs intra-articular placebo, since the superiority of hylan G-F 20 compared with intra-articular placebo had already been demonstrated in patients with knee OA [32] using the WOMAC A as a primary efficacy criterion. Additionally, treating patients with a placebo in a country where more than 13 various intra-articular HA preparations were available and reimbursed by the healthcare system would be complicated to perform and considered as unethical. On another hand, it is recognized that the placebo used in randomized trials evaluating intra-articular therapies in knee OA is not a “true” placebo, i.e., it has an effect size on pain that is superior to oral placebo [46]. The intra-articular placebo effect had already been highlighted in the 50’s, when Desmarais and colleagues showed that a single saline injection, or even a puncture with a needle (no injection), could provide pain relief in OA [47]. This has to be considered in addition to the known major placebo effect observed in OA trials [48]. The magnitude of the intra-articular placebo effect has been recently estimated at 0.29 in a recent network meta-analysis of alternative placebo preparations in OA [46], leading to the reaffirmation of "Needle Is Better Than Pill" [49]. To a lesser degree, this randomized controlled trial is also limited by its six month predefined endpoint, which did not allow knowing the true duration of the symptomatic effect of SH. A one year follow-up would have provided information on a potentially longer duration of the clinical benefit.

The prevalence of symptomatic knee OA has increased over the past decades and the burden associated to the disease is expected to dramatically grow within the next years [50]. This phenomenon is, in particular, reflected by the huge increase of total joint replacements performed each year in most countries, which is expected to be multiplied by 600% and 300% by 2030 in the US or the Netherlands, respectively [51,52]. Considering its cost and potential iatrogenicity, it is mandatory to limit total joint replacement surgery to refractory end-stage knee OA and to favor the use and prescription of conservative treatment modalities. Beside their proven and recognized effect on pain and functional limitations, there is an increasing amount of evidence that intra-articular HA injections could delay the need for total knee replacement, as recently reported by various authors [53–57]. As a result, intra-articular HA injections should be considered as a valuable therapeutic option in the symptomatic management of knee OA [28].

## Conclusion

This well-designed study has demonstrated the non-inferiority of a single intra-articular injection of SH vs a single intra-articular injection of hylan G-F 20, which had been previously shown to be superior to intra-articular placebo. The effect size observed on pain reduction and functional improvement were clinically significant, and the percentage of responders to the treatment according to the OMERACT-OARSI responder criteria was higher than 80% in each treatment group. Given the potentially safer and higher tolerability of “one-shot” preparations, the use of a single SH injection should be encouraged as a second-line treatment in knee OA patients.

## Supporting information

S1 Checklist CONSORT 2010 checklist of information to include when reporting a randomized clinical trial. (DOC)

S1 Protocol Original version of the study protocol (in French). (PDF)

S2 Protocol Translated version of the study protocol (in English). (PDF)

S1 Appendix Changes to the database. (DOCX)

S2 Appendix Adjustments for covariates. (DOCX)

S1 Table Patients who discontinued the trial or were lost to follow-up (Intention-to-Treat population). (DOCX)

S2 Table Patients with protocol deviations (Intention-to-Treat population). (DOCX)

S3 Table Patient demographics and baseline characteristics (Full Analysis Set). (DOCX)

S4 Table Patient demographics and baseline characteristics (Per Protocol dataset). (DOCX)

S5 Table Individual patient demographics and baseline characteristics (Intention-to-Treat population). (DOCX)

S6 Table Primary and secondary efficacy criteria at 6 months post injection (Full Analysis Set). (DOCX)

S7 Table Individual WOMAC A pain values (mm) (Intention-to-Treat population). (DOCX)

S8 Table Individual WOMAC B stiffness values (mm) (Intention-to-Treat population). (DOCX)

S9 Table Individual WOMAC C function values (mm) (Intention-to-Treat population). (DOCX)

S10 Table Individual Lequesne index (Intention-to-Treat population). (DOCX)

S11 Table Individual patient global assessment of disease activity (mm) (Intention-to-Treat population). (DOCX)

S12 Table Individual global treatment efficacy (injected patients). (DOCX)

S13 Table Individual acetaminophen use post injection (Intention-to-Treat population). (DOCX)

S14 Table Treatment-emergent adverse events (as number of occurrences) by System Organ Class (injected patients). (DOCX)

S15 Table Treatment-emergent adverse events (as number of patients) by System Organ Class (injected patients). (DOCX)

S16 Table Individual adverse events (Intention-to-Treat population). (DOCX)

S17 Table Individual local post injection reactions (injected patients). (DOCX)

S1 Fig Evolution of the secondary efficacy criteria during the 6-month trial (Full Analysis Set). (TIF)
