# Supplementary material for: A single intra-articular injection of 2.0% non-chemically modified sodium hyaluronate vs 0.8% hylan G-F 20 in the treatment of symptomatic knee osteoarthritis: A 6-month, multicenter, randomized, controlled non-inferiority trial
Source: PLoS One. 2019 Dec 10;14(12):e0226007. doi: 10.1371/journal.pone.0226007 (PMC6903764; doi:10.1371/journal.pone.0226007)
Supplement: S1 Protocol — (PDF) [file pone.0226007.s002.pdf]

S1 Protocol. Original version of the study protocol (in French).

ESSAI CLINIQUE D'EVALUATION DE L'EFFICACITE D'OSTENIL®PLUS (ACIDE HYALURONIQUE) VERSUS SYNVISC-ONE™ DANS LE TRAITEMENT DE LA GONARTHROSE FEMORO-TIBIALE

ETUDE EN DOUBLE AVEUGLE RANDOMISEE CONTROLEE D'OSTENIL® PLUS VERSUS SYNVISC-ONE™ DE DEUX GROUPES PARALLELES DE PATIENTS SUIVIS PENDANT SIX MOIS

PROTOCOLE

COORDINATEUR NATIONAL

Pr. THOMAS BARDIN (APHP LARIBOISIERE)
2, RUE AMBROISE PARE
75010 PARIS
☏ 01.49.95.62.90

PROMOTEUR ET CHEF DE PROJET

TRB CHEMEDICA
Dr. CHARLES JOLLES
IMMEUBLE ABC 1
SITE D'ARCHAMPS BP 218
74162 ARCHAMPS CEDEX
☏ 04.50.95.09.00 🗎 04.50.95.09.01

CONSEILLERE SCIENTIFIQUE

Dr. RENEE LILIANE DREISER
APHP BICHAT - CLAUDE BERNARD
46, RUE HENRI HUCHARD
75018 PARIS
☏ 01.42.80.95.73

COMITE SCIENTIFIQUE

Dr. RENEE LILIANE DREISER (APHP BICHAT - CLAUDE BERNARD)
Dr. BERNARD AVOUAC (APHP HENRI MONDOR)

GESTION DE L'ESSAI

SPRIT
7, RUE LALLIER
75009 PARIS
☏ 01.48.78.87.78 🗎 01.48.78.87.82

STATISTIQUES

CEMKA
Dr. ANTOINE LAFUMA
43, BOULEVARD DU MARECHAL JOFFRE
92340 BOURG LA REINE
☏ 01.40.91.30.30 🗎 01.40.91.30.31

1. PAGE DE TITRE

**Titre**	Essai clinique d'évaluation de l'efficacité d'OSTENIL® PLUS (acide hyaluronique) versus SYNVISC-ONE™ dans le traitement de la gonarthrose fémoro-tibiale. Etude en double aveugle randomisée contrôlée d'OSTENIL® PLUS versus SYNVISC-ONE™ de 2 groupes parallèles de patients suivis pendant 6 mois.
**Lieu de l'étude**	FRANCE
**But de l'étude**	Démontrer la non infériorité de l'efficacité d'OSTENIL® PLUS par rapport à SYNVISC-ONE™ dans la gonarthrose fémoro-tibiale symptomatique.
**Plan expérimental**	Etude contrôlée, randomisée en groupes parallèles comparant deux groupes de patients recevant soit 1 injection intra-articulaire d'OSTENIL® PLUS soit 1 injection de SYNVISC-ONE™. Etude en double aveugle : patient et investigateur évaluateur aveugles.
**Protocole**<br>**N° ID-RCB**	OSTP-EUR-10-01<br>2011-A00258-33
**Phase**	IV
**Promoteur**<br>**Coordinateur national**	TRB CHEMEDICA<br>Pr. THOMAS BARDIN (APHP LARIBOISIERE)
**Début d'essai**	Mai 2011
**Fin d'essai**	Juin 2013
**Gestion de l'essai**	SPRIT
**Date du protocole**	16 Mai 2011

2. SYNOPSIS

**Promoteur :** TRB CHEMEDICA	
**Nom du produit étudié :** OSTENIL® PLUS	
**Nom du principe actif :** Hyaluronate de sodium 2,0%	
**Titre de l'étude :** Essai clinique d'évaluation de l'efficacité d'OSTENIL® PLUS (acide hyaluronique) versus SYNVISC-ONE™ dans le traitement de la gonarthrose fémoro-tibiale. Etude en double aveugle randomisée contrôlée d'OSTENIL® PLUS versus SYNVISC-ONE™ de deux groupes parallèles de patients suivis pendant six mois.	
**Coordinateur :** Pr. THOMAS BARDIN (APHP LARIBOISIERE)	
**Nombre d'investigateurs et de centres:** 75 centres de deux médecins investigateurs l'un évaluateur et l'autre injecteur.	
**Période prévue pour l'étude :**<br>Mai 2011<br>Fin juin 2013	**Phase de développement :**<br>Phase IV
**Objectifs de l'étude :** Démontrer la non infériorité de l'efficacité d'OSTENIL® PLUS par rapport à SYNVISC-ONE™ dans la gonarthrose fémoro-tibiale symptomatique.	
**Plan expérimental :** Etude contrôlée, randomisée en groupes parallèles comparant deux groupes de patients recevant soit une injection intra-articulaire d'OSTENIL® PLUS soit une injection de SYNVISC-ONE™. Etude en double aveugle : patient et évaluateur aveugles.	
**Nombre de patients :** Deux groupes de 130, soit un total de 260 patients.	
**Principaux critères d'inclusion :** Patient homme ou femme âgé de 40 à 85 ans, souffrant d'une gonarthrose fémoro-tibiale unilatérale ou bilatérale, conforme aux critères de l'ACR et confirmée radiologiquement (stades Ib à III de KELLGREN / LAWRENCE) avec une douleur appréciée sur le score moyen des échelles de la douleur de WOMAC (section A) supérieure ou égale à 40 mm.	
**Produit étudié** : OSTENIL® PLUS	
**Forme galénique** : Solution viscoélastique pour injection.	

Promoteur : TRB CHEMEDICA

Nom du produit étudié : OSTENIL® PLUS

Nom du principe actif : Hyaluronate de sodium 2,0%

Voie d'administration : Intra-articulaire

Posologie : Une injection intra-articulaire.

Numéros de lots :

Durée du traitement : 1 injection, 6 mois de suivi par patient, 5 consultations.

Produit comparateur : SYNVISC-ONE™

Forme galénique : Solution viscoélastique pour injection.

Voie d'administration : Intra-articulaire.

Posologie : Une injection intra-articulaire.

Numéros de lots :

Critères d'évaluation de l'efficacité :
Le critère principal est :
L'évolution du score moyen des échelles de la douleur de WOMAC (section A) de J0 à J180
Les critères secondaires de jugement sont :
L'indice de LEQUESNE,
L'évolution du score moyen des échelles de la raideur et de la fonction physique de WOMAC (section B et C),
L'évaluation globale de l'efficacité et de la tolérance par le patient et l'investigateur,
La cotation du statut global du patient en relation avec sa gonarthrose sur EVA,
La consommation d'antalgiques et d'AINS,
Le pourcentage de patients répondeurs selon les critères de l'OMERACT-OARSI.

Critères d'évaluation de la tolérance :
On étudiera les proportions de sujets présentant ou non un ou plusieurs évènements indésirables.

PARTIE I : CONDUITE DE L'ESSAI

3. SOMMAIRE

1. PAGE DE TITRE

2. SYNOPSIS

3. SOMMAIRE

4. LISTE DES ABREVIATIONS

5. ASPECTS REGLEMENTAIRES ET ETHIQUES

6. INVESTIGATEURS, ORGANISATION ET PERSONNES PARTICIPANT A L'ETUDE

7. INTRODUCTION
7.1 Importance de la pathologie arthrosique
7.2 Prise en charge de l'arthrose
7.3 Place des acides hyaluroniques dans le traitement de la gonarthrose
7.4 Les acides hyaluroniques en France
7.4.1 Généralités
7.4.2 Ostenil® Plus
7.4.2.1 Essais cliniques d'Ostenil® Plus
7.4.2.2 Commercialisation d'Ostenil® Plus
7.4.3 Synvisc-One™
7.4.3.1 Essais cliniques de Synvisc-One™

8. OBJECTIF

9. PLAN EXPERIMENTAL
9.1 Schéma expérimental de l'étude
9.2 Discussion sur le plan expérimental
9.3 Population étudiée
9.3.1 Critères de sélection
9.3.1.1 Critères d'inclusion
9.3.1.2 Critères de non inclusion
9.3.2 Patient exclu en cours d'étude et arrêt prématuré de traitement
9.4 Traitements
9.4.1 Produits administrés
9.4.1.1 Forme pharmaceutique et dosage
9.4.2 Schéma thérapeutique
9.4.2.1 Présentation et étiquetage
9.4.2.2 Attribution des traitements
9.4.2.3 Aveugle
9.4.2.4 Stockage et gestion des traitements

9.4.3 Choix de la dose pour l'étude
9.4.4 Traitements antérieurs et concomitants
9.4.4.1 Traitements antérieurs
9.4.4.2 Traitements associés
9.4.4.3 Traitements interdits
9.4.5 Observance des patients
9.5 Evaluation de l'efficacité et de la tolérance
9.5.1 Critères d'évaluation
9.5.1.1 Critères principal d'évaluation
9.5.1.1.1 Efficacité
9.5.1.1.2 Tolérance
9.5.1.2 Critères secondaires d'évaluation
9.5.1.2.1 Efficacité
9.5.1.2.2 Tolérance
9.5.2 Pertinence des critères d'évaluation
9.5.2.1 Critère principal d'efficacité
9.5.3 Critères d'évaluation de la tolérance clinique
9.5.3.1 Evènements indésirables
9.5.3.2 Etude de la tolérance
9.6 Rapport final
9.7 Méthodes statistiques prévues et calcul du nombre de sujets
9.7.1 Effectif choisi
9.7.2 Paramètres utilisés pour le calcul du nombre de sujets
9.7.3 Evaluation statistique
9.7.3.1 Méthode de l'analyse statistique
9.7.3.1.1 Revue des données en aveugle
9.7.3.1.2 Description de l'échantillon
9.7.3.1.3 Comparaison des groupes avant traitement
9.7.3.2 Critères de jugement
9.7.3.2.1 Critère principal de l'efficacité
9.7.3.2.2 Critères secondaires
9.7.3.2.3 Critère de jugement de la tolérance
9.7.3.3 Analyses statistiques
9.7.3.3.1 Définition des populations analysées
9.7.3.3.1.1 Population en intention de traiter (ITT)
9.7.3.3.1.2 Population per protocole (PP)

10. CALENDRIER

PARTIE II : PRINCIPES GENERAUX

1. CONSIDERATIONS ETHIQUES
1.1 Déclaration d'Helsinki
1.2 Bonnes pratiques cliniques

2. RESPONSABILITES DES INVESTIGATEURS
2.1 Ethique
2.1.1 Déclaration d'Helsinki
2.1.2 CPP
2.1.3 Information au patient
2.1.4 Consentement éclairé du patient
2.1.5 Retrait d'un sujet
2.1.6 Anonymat du sujet
2.1.7 Frais
2.2 Amendements au protocole
2.3 Cahier d'observation
2.4 Vérification des documents sources
2.5 Evènements indésirables
2.5.1 Définition
2.5.1.1 Evènements indésirables graves
2.5.1.2 Rapport des évènements indésirables graves
2.5.1.3 Décès
2.5.1.4 Sorties d'étude
2.5.1.5 Evénements indésirables non graves
2.5.1.6 Traitement et suivi des évènements indésirables
2.6 Publications
2.7 Archivage
2.8 Audit

3. RESPONSABILITES DU PROMOTEUR ET DES SOCIETES DE SERVICE
3.1 Responsabilités générales
3.2 Assurance
3.3 Monitoring et responsabilités des sociétés de service
3.4 Assurance qualité et contrôle qualité
3.5 Archives

4. SIGNATURES

PARTIE III : ANNEXES

1. ANNEXES LIEES A L'ETUDE

1.1 Critères ACR de la gonarthrose

1.2 Stades radiologiques de Kellgren / Lawrence

1.3 Indice algo-fonctionnel de LEQUESNE pour la gonarthrose

1.4 WOMAC - Western Ontario McMaster's Arthritis Index –

1.5 Critères de l'OMERACT-OARSI

1.6 Liste des traitements AINS

1.7 Temps de wash out des AINS

1.8 Echelle visuelle analogique EVA

1.9 Certificat d'analyse

1.10 Curriculum vitae du coordinateur

1.11 Investigateurs participant à l'essai

1.12 Cahiers d'observations

1.13 Cahier patient

1.14 Notice d'information scientifique d'Ostenil® Plus et Synvisc-One™

1.15 Fiche de pharmacovigilance et rapport d'évènement indésirable grave (EIG) ou « Adverse Event (SAE) Report »

1.16 Accusé de réception

1.17 Approbation du protocole et engagement de confidentialité

2. ANNEXES LIEES AUX OBLIGATIONS LEGALES

2.1 Déclaration d'Helsinki

2.2 Loi Huriet

2.3 Circulaire d'application sur les DMOS

2.4 Bonnes pratiques cliniques

2.5 Avis du CPP

2.6 Avis de l'AFSSAPS

2.7 Notice d'information au patient

2.8 Consentement éclairé du patient

2.9 Certificat d'assurance

2.10 Références

4. LISTE DES ABREVIATIONS

ACR	American College of Rheumatology
AFSSAPS	Agence Française de Sécurité Sanitaire des Produits de Santé
AH	Acide Hyaluronique
AINS	Anti-Inflammatoire Non Stéroïdien
ANOVA	Analyse de Variance
ARC	Attaché de Recherche Clinique
AASAL	Anti-Arthrosique d'Action Lente
BPC	Bonnes Pratiques Cliniques
C	Consultation
CPP	Comité de Protection des Personnes
CM	Centimètre
CNIL	Commission Nationale Informatique et Liberté
CO	Cahier d'Observation
CRO	Contract Research Organisation
CV	Curriculum Vitae
DM	Donnée Manquante
EI	Evènement Indésirable
EIG	Evènement Indésirable Grave
ES	Effet Secondaire
EULAR	European League Against Rheumatism
EVA	Echelle Visuelle Analogique
F	Femme
G	Gramme
H	Homme
h	Heure
IA	Intra-Articulaire
ICH	International Conference on Harmonisation
ITT	Intent To Treat
J	Jour
KG	Kilogramme
LOCF	Last Observation Carried Forward
LPPR	Liste des Produits et des Prestations Remboursables
M	Mois
MAR	Missing At Random
MCAR	Missing Completely At Random
MedDRA	Medical Dictionary for Regulatory Activities
MG	Milligramme
PM	Poids Moléculaire
PP	Per Protocole
SD	Standard Deviation
WOMAC	Western Ontario McMaster's Arthritis Index

5. ASPECTS REGLEMENTAIRES ET ETHIQUES

Voir PARTIE II

6. INVESTIGATEURS, ORGANISATION ET PERSONNES PARTICIPANT A L'ETUDE

**COORDINATEUR NATIONAL**	Pr. THOMAS BARDIN (APHP LARIBOISIERE)<br>2, RUE AMBROISE PARE<br>75010 PARIS<br>☏ 01.49.95.62.90
**PROMOTEUR ET CHEF DE PROJET**	TRB CHEMEDICA<br>Dr. CHARLES JOLLES<br>IMMEUBLE ABC 1<br>SITE D'ARCHAMPS BP 218<br>74162 ARCHAMPS CEDEX<br>☏ 04.50.95.09.00 🗎 04.50.95.09.01
**CONSEILLERE SCIENTIFIQUE**	Dr. RENEE LILIANE DREISER<br>APHP BICHAT - CLAUDE BERNARD<br>46, RUE HENRI HUCHARD<br>75018 PARIS<br>☏ 01.42.80.95.73
**COMITE SCIENTIFIQUE**	Dr. RENEE LILIANE DREISER (APHP BICHAT - CLAUDE BERNARD)<br>Dr. BERNARD AVOUAC (APHP HENRI MONDOR)
**GESTION DE L'ESSAI**	SPRIT<br>7, RUE LALLIER<br>75009 PARIS<br>☏ 01.48.78.87.78 🗎 01.48.78.87.82
**INVESTIGATEURS**	VOIR ANNEXE
**STATISTIQUES**	CEMKA<br>Dr. ANTOINE LAFUMA<br>43, BOULEVARD DU MARECHAL JOFFRE<br>92340 BOURG LA REINE<br>☏ 01.40.91.30.30 🗎 01.40.91.30.31
**RAPPORT CLINIQUE**	Pr. THOMAS BARDIN (APHP LARIBOISIERE)<br>2, RUE AMBROISE PARE<br>75010 PARIS<br>☏ 01.49.95.62.90

7. INTRODUCTION

7.1 IMPORTANCE DE LA PATHOLOGIE ARTHROSIQUE

L'arthrose est la maladie articulaire la plus répandue dans la population. Elle affecte en effet plus de la moitié des personnes de plus de 65 ans et atteint 80 % des plus de 75 ans (1). Elle concerne plus fréquemment les femmes et les personnes âgées de plus de 45 ans. La prévalence de l'arthrose devrait considérablement augmenter en raison de l'allongement de l'espérance moyenne de vie, du vieillissement de la population et du maintien des activités professionnelles et sportives jusqu'à un âge avancé (2). Il s'agit donc d'un problème majeur de Santé Publique, dont le poids économique aux USA d'après ALTMAN « excède le produit national brut de plusieurs pays » (3).
Les principaux facteurs étiologiques ou facteurs de risque de gonarthrose sont l'âge, les antécédents familiaux, l'obésité, les traumatismes du genou (entorses, ruptures ligamentaires, méniscopathies), la méniscectomie, certaines activités professionnelles ou sportives arthrogènes. Il existe une corrélation étroite entre obésité et gonarthrose (4).
La gonarthrose symptomatique est une pathologie chronique invalidante responsable d'un handicap important. Elle a un fort retentissement sur la qualité de vie (5).
En 2006, le nombre de patients gonarthrosiques en France était estimé entre 1,8 et 2,3 millions de personnes (6).
Les symptômes principaux de l'arthrose sont la douleur, la gêne fonctionnelle qui en découle et la raideur articulaire. La plupart des traitements actuels vise à améliorer ces symptômes, en particulier le premier d'entre eux, la douleur. Ils n'y parviennent qu'incomplètement (4), et n'empêchent pas qu'un certain nombre de patients doivent recourir à la chirurgie (en général prothétique) pour améliorer leur état. On estime à 50 000 le nombre de prothèses de genou posées par an en France, ce qui représente aussi un coût très important pour la collectivité.
Aucun des traitements médicaux actuellement disponibles sur le marché ne permet de stopper la progression de l'arthrose ou d'en réduire durablement les symptômes de façon certaine.

7.2 PRISE EN CHARGE DE L'ARTHROSE

Toutes les recommandations récentes insistent sur l'importance au plan médical comme économique d'une prise en charge thérapeutique précoce de la gonarthrose, pour retarder voire limiter le recours aux traitements lourds et notamment à la chirurgie.
Un groupe de travail composé de délégués de treize pays européens, sous l'égide de la ligue européenne contre le rhumatisme (EULAR) a commencé en septembre 1998 à élaborer ses recommandations pour le traitement de la gonarthrose.

Elles ont été publiées à la fin 2000 et révisées en 2003 (7). La méthodologie a été élaborée en collaboration avec le Départment of Public Health du « Saint Georges Hospital Medical School » de Londres, pour répondre aux critères de l' «evidence based medicine» mais une partie non négligeable des mesures thérapeutiques dans le domaine de l'arthrose n'a toujours pas été étudiée scientifiquement. Aussi dans un processus décisionnel commun en plusieurs tours (méthode Delphi) parmi les 24 experts Européens 23 modalités de traitement de la gonarthrose ont été examinées et 10 recommandations ont été adoptées et publiées.
Le traitement doit être adapté à chaque patient (7, 8). Les anti-arthrosiques symptomatiques d'action lente (AASAL) peuvent être utilisés chez les patients dont les symptômes sont mal contrôlés. La recommandation n°8 concerne l'acide hyaluronique, qui est une des modalités thérapeutiques recommandées au chapitre des anti-arthrosiques symptomatiques d'action lente.

Plus récemment en 2010, l'OARSI a élaboré 25 recommandations concernant les modalités de prise en charge de l'arthrose (9). Les infiltrations intra-articulaires d'acide hyaluronique continuent de figurer parmi ces recommandations avec une taille d'effet jugée comme modérée (effect size 0.60).

7.3 PLACE DES AH DANS LE TRAITEMENT DE LA GONARTHROSE

L'acide hyaluronique (AH) est un macro polysaccharide, produit de la polymérisation de milliers d'unités disaccharidiques de base composées, de N-acétyl glucosamine et d'acide N-glucuronique. Son poids moléculaire à l'état normal dans le cartilage ou le liquide synovial est d'environ 4 à 5 millions de Daltons (Da).
Les arguments ayant conduit à l'utilisation des acides hyaluroniques dans le traitement de la gonarthrose sont les suivants :
Au cours de la maladie arthrosique, la concentration en acide hyaluronique du liquide synovial est très diminuée et la viscosité est en conséquence moins importante. L'augmentation d'acide hyaluronique de faible poids moléculaire contribue également à la baisse de la viscosité. Cette dégradation des propriétés rhéologiques entraîne des altérations sévères de la matrice intercellulaire et des fonctions cellulaires. La disparition des effets lubrifiants et protecteurs de l'acide hyaluronique permet aux forces de cisaillement, causées par le mouvement des articulations, de provoquer la destruction du cartilage articulaire par frottement.
Au début des années 1980, l'acide hyaluronique ou hyaluronane (appellation internationale désormais recommandée) a été essayé dans le traitement de la gonarthrose (10). Plus d'une soixantaine d'essais cliniques ont été publiés depuis, avec différents hyaluronanes, versus placebo, versus corticoïdes intra-articulaires et versus AINS. La plupart de ces essais thérapeutiques ont été réalisés dans la gonarthrose et fournissent des preuves de l'efficacité clinique symptômatique des acides hyaluroniques dans le traitement de celle-ci (10 ,11). Une méta-analyse conclut à l'efficacité des acides hyaluroniques dans le traitement de la gonarthrose (effets bénéfiques sur la douleur, la fonction, et l'évaluation globale du patient) (12). Cependant certaines méta-analyses (13, 14, 15) font état d'une efficacité modérée des acides hyaluroniques et c'est pourquoi l'AFSSAPS a demandé de nouveaux essais dans cette pathologie.

7.4 LES AH EN FRANCE

7.4.1 Généralités

Il existe en France une quinzaine de préparations différentes d'acide hyaluronique, pour le traitement de la gonarthrose, inscrites comme dispositifs médicaux, une étant détentrice d'une AMM de médicament.
Elles se distinguent par leur mode d'extraction (crête de coq ou bio-fermentation), leur structure réticulée avec un poids moléculaire supérieur à 6 millions Daltons (Synvisc® – Durolane®) ou leur structure non réticulée (Adant® – Sinovial® – Arthrum®) avec un poids moléculaire variant de 600.000 à 1,2 millions de Daltons, leur concentration (0,8 à 2%), et enfin leur dose unitaire (2 à 3 ml).

Depuis 1999 en France, les injections intra-articulaires d'acide hyaluronique ont obtenu un remboursement à 65% dans le traitement de la gonarthrose à la condition qu'elles soient réalisées par un rhumatologue, un médecin de rééducation physique ou un orthopédiste. Le traitement est pour presque tous les produits de trois injections à un rythme hebdomadaire. Le traitement n'est indiqué et remboursable qu'en cas de gonarthrose symptomatique, et après échec ou intolérance des antalgiques de première intention et des AINS, à raison d'un traitement de 3 injections par genou et par an au maximum. Cependant, deux produits sont utilisés avec une seule injection (Durolane® - Synvisc-one™).

7.4.2 Ostenil® Plus

Ostenil® Plus appartient aux acides hyaluroniques de structure non réticulée, obtenu par fermentation bactérienne et dépourvu de protéines animales.

Ostenil® Plus est une solution visco-élastique transparente d'acide hyaluronique naturel et hautement purifié à 2% d'hyaluronate de sodium, sel sodique de l'acide hyaluronique, réservée strictement aux injections intra-articulaires.

Ostenil® Plus contient aussi du mannitol, un piégeur de radicaux libres, qui aide à stabiliser les chaînes d'acide hyaluronique.

Le poids moléculaire moyen est compris entre 1,5 et 2 millions de Daltons.

7.4.2.1 Essais cliniques d'Ostenil® Plus

Lors d'une étude pilote, ouverte, non contrôlée, multicentrique, menée en Espagne, 80 patients souffrant d'arthrose du genou ont reçu une injection d'Ostenil® Plus (J0) et ont été suivis pendant 6 mois, avec des évaluations à 0, 15, 30, 60, 90, 120, 150 et 180 jours.

L'évaluation clinique de la douleur et de la fonction articulaire ont été effectuées à l'aide d'une échelle visuelle analogique (EVA) et de l'indice de WOMAC.

Une réduction significative de la douleur articulaire, de la raideur et de l'incapacité fonctionnelle par rapport à la ligne de base a été observée à chaque visite de suivi ($p < 0,001$), dès le 15ème jour.

La douleur articulaire a été améliorée de 40,7 % (EVA) et 38,7 % (WOMAC) à J 30, atteignant respectivement 46,5 % et 47,5 % à J 180.

L'évaluation de l'efficacité et de la tolérance par l'investigateur et le patient ont été considérées comme excellentes tout au long de l'étude.

Des effets secondaires bénins (douleur locale et gonflement de la zone d'infiltration) ont été signalés chez 4 patients à J 15.

Aucun événement indésirable grave n'a été observé.

7.4.2.2 Commercialisation d'Ostenil® Plus

Ostenil® Plus a été commercialisé la première fois en Allemagne en février 2009.

Depuis lors, le produit a été mis sur le marché dans une série de pays en Europe (Belgique, Bulgarie, Espagne, France, Grèce, Hongrie, Luxembourg, Pays-Bas, Roumanie, Royaume Uni) et en Suisse.

A fin décembre 2010, près de 87 900 seringues d'Ostenil® Plus ont été écoulées au niveau mondial. Aucun incident n'a été déclaré aux autorités compétentes.

7.4.3 Synvisc-One™

Synvisc-One™ contient un type de polymères hylanes techniquement appelé hylane G-F20, fluide élastovisqueux stérile et non pyrogène.

Les hylanes sont des dérivés de l'hyaluronane (l'hyaluronate de sodium) et elles sont composées d'unités disaccharidiques récurrentes de N-acétylglucosamine et de glucuronate de sodium.

Hylane G-F20 contient de l'hylane A et de l'hylane B (8,0 mg ± 2,0 mg par ml).

L'hylane A a un poids moléculaire moyen de 6 millions de Daltons et l'hylane B est un gel hydraté.

7.4.3.1 Essais cliniques de Synvisc-One™

Le Synvisc-One™ a fait récemment l'objet d'un essai comparatif versus placebo en double aveugle (injecteur et évaluateur aveugles). Cette étude randomisée a été réalisée sur deux groupes parallèles de 253 patients atteints de gonarthrose douloureuse et traités par une injection de Synvisc-One™ ou placebo selon la randomisation puis suivis durant 26 semaines.

Les résultats ont montré la supériorité de Synvisc-One™ une injection unique d'Hylan G-F20 de 6 ml par rapport au placebo concernant le critère principal ; WOMAC douleur (-0,15 (SE), $p=0,047$) ainsi que sur les critères secondaires (16).

Dans cet essai, la tolérance de Synvisc-One™ a été satisfaisante et non statistiquement différente de celle du placebo.

Aucun effet indésirable grave n'a été signalé.

Pour conclure, la plupart des préparations d'acide hyaluronique actuellement disponibles sont des solutions de 1,0 % et exigent 3 à 5 injections afin de rétablir les propriétés viscoélastiques du liquide synovial dans l'arthrose du genou.

Une concentration plus élevée (2 % d'acide hyaluronique) doit permettre une amélioration plus rapide et plus soutenue des propriétés viscoélastiques du liquide synovial.

Afin de confirmer les résultats de l'étude pilote, il a été jugé utile de réaliser un nouvel essai chez un plus grand nombre de patients avec Ostenil® Plus en comparaison avec un produit de référence : le Synvisc-One™.

8. OBJECTIF

Il s'agit d'une étude de non-infériorité de l'efficacité d'Ostenil® Plus par rapport à Synvisc-One™ dans la gonarthrose fémoro-tibiale symptomatique.
Le critère principal est l'évolution du score moyen des échelles de la douleur de WOMAC (section A) (17) (Annexe 1.4) de J0 à J180 chez des patients recevant un traitement par une injection intra-articulaire d'OSTENIL® PLUS versus une injection intra-articulaire de SYNVISC-ONE™.
Secondairement, celle-ci permettra de fournir des données concernant la tolérance respective des deux traitements.

9. PLAN EXPERIMENTAL

9.1 Schéma expérimental de l'étude

Etude de phase IV, prospective, randomisée, contrôlée en double aveugle de deux groupes parallèles, multicentrique comparant l'administration d'une injection intra-articulaire d'OSTENIL® PLUS à l'administration d'une injection intra-articulaire de SYNVISC-ONE™ chez des patients gonarthrosiques symptomatiques.
260 patients sont prévus au total.

L'étude se déroulera dans toutes les régions de France.
Il est prévu de recruter 75 investigateurs évaluateurs et 75 investigateurs injecteurs qui réaliseront 4 à 8 observations par centre.
La période de recrutement sera de dix huit mois après la première mise en place. Les patients seront traités une fois et suivis pendant six mois.
Le traitement sera une unique injection d'acide hyaluronique.
5 visites par patient sont prévues.

C 0 (J -7) Visite de présélection par le médecin évaluateur

A l'issue de la visite, si le patient prend des AINS il sera soumis à un wash out de 2 à 5 jours avec un minimum de 2 jours (Annexe 1.7).
Le paracétamol sera autorisé sous réserve d'un wash out d'au minimum 24 heures avant C 1.
Si un wash out n'est pas nécessaire C 0 et C 1 peuvent être confondues.
L'investigateur s'assurera que le patient possède des radiographies datant de moins d'un an.

C 1 (J 0) Visite d'inclusion par le médecin évaluateur

Evaluation du patient sur le plan douloureux.

Il est nécessaire que le patient présente une moyenne douloureuse supérieure ou égale à 40 mm (EVA cotées de 0 à 100 mm) sur les échelles de douleur (section A) de WOMAC (Annexe 1.4) pour le genou à infiltrer, avec une différence d'au moins 20 mm par rapport à la douleur du genou controlatéral si la gonarthrose est bilatérale. En cas d'arthrose bilatérale, le genou le plus douloureux sera retenu.

Données démographiques : âge, taille, poids, IMC, état général.

Caractéristiques de la gonarthrose.

Critères de l'ACR (18) (Annexe 1.1).

Critères radiologiques (19) (Annexe 1.2).

Données descriptives : antécédents, traitements antérieurs et concomitants.

Contrôle des critères d'inclusion et de non inclusion.

Indice algo-fonctionnel de LEQUESNE (20) (Annexe 1.3).

Cotation du statut global du patient en relation avec sa gonarthrose sur EVA (21) (Annexe 1.8).

Consentement éclairé signé du patient.

Déclaration d'inclusion.

Le patient sera alors adressé à un rhumatologue « injecteur » ou à un médecin de médecine physique ou à un chirurgien orthopédiste pour l'administration du produit.

A partir de cette visite et pendant toute la durée de l'étude, le patient notera sur un cahier la consommation d'antalgiques (paracétamol, nombre de comprimés et dose).
Tout traitement par le paracétamol devra être interrompu au moins 12 heures avant chaque visite (de préférence 24 heures).

C 2 (J 2) Visite d'injection (± 2 jours) par le médecin injecteur

Consommation de paracétamol, (vérification du cahier patient).

Description des évènements indésirables.

Modifications des traitements en cours.

Cotation du statut global du patient en relation avec sa gonarthrose sur EVA.

Injection du produit à l'essai.

Confirmation d'injection.

C 3 (J 30) Visite de suivi (± 15 jours) par le médecin évaluateur

Consommation de paracétamol, (vérification du cahier patient).

Modifications des traitements en cours.

Jugement de l'efficacité globale du traitement par le patient et l'investigateur.

Jugement de la tolérance globale au traitement par le patient et l'investigateur.

Jugement de la tolérance locale au traitement par le patient et l'investigateur.

Description des évènements indésirables.

WOMAC total (Annexe 1.4).

Indice algo-fonctionnel de LEQUESNE (Annexe 1.3).

Cotation du statut global du patient en relation avec sa gonarthrose sur EVA (Annexe 1.8).

C 4 (J 90) Visite de suivi (± 15 jours) par le médecin évaluateur

Consommation de paracétamol, (vérification du cahier patient).

Modifications des traitements en cours.

Jugement de l'efficacité globale du traitement par le patient et l'investigateur.

Jugement de la tolérance globale au traitement par le patient et l'investigateur.

Description des évènements indésirables.

WOMAC total (Annexe 1.4).

Indice algo-fonctionnel de LEQUESNE (Annexe 1.3).

Cotation du statut global du patient en relation avec sa gonarthrose sur EVA.

C 5 (J 180) Visite de fin d'essai (± 15 jours) par le médecin évaluateur
Consommation de paracétamol, (vérification du cahier patient).
Modifications des traitements en cours.
Jugement de l'efficacité globale du traitement par le patient et l'investigateur.
Jugement de la tolérance globale au traitement par le patient et l'investigateur.
Description des évènements indésirables.
WOMAC total (Annexe 1.4).
Indice algo-fonctionnel de LEQUESNE (Annexe 1.3).
Cotation du statut global du patient en relation avec sa gonarthrose sur EVA (Annexe 1.8).
Feuille de fin d'essai.

En cas d'arrêt prématuré de l'étude ou de consultation intermédiaire non réalisée, une visite finale devra être effectuée et les motifs d'interruption devront être clairement détaillés.

SCHEMA DE L'ETUDE

	J 0<br>C 1	J 2<br>C 2<br>IA	J 30<br>C 3<br>Suivi	J 90<br>C 4<br>Suivi	J 180<br>C 5<br>Fin
Données démographiques	X				
Antécédents médicaux chirurgicaux	X				
Traitements en cours	X				
Caractéristiques de la gonarthrose	X				
Critères ACR	X				
Classification d'ALTMAN	X				
Critères d'inclusion	X				
Critères de non-inclusion	X				
Injection groupe d'OSTENIL® PLUS ou SYNVISC-ONE ™		X			
Recueil du consentement éclairé	X				
Déclaration d'inclusion	X				
**Appréciation de l'efficacité**					
Total WOMAC	X		X	X	X
LEQUESNE	X		X	X	X
Cotation du statut global du patient EVA	X	X	X	X	X
Evaluation globale par l'investigateur			X	X	X
Evaluation globale par le patient			X	X	X
**Appréciation de l'observance**					
Consommation de paracétamol		X	X	X	X
Traitements concomitants		X	X	X	X
**Appréciation de la tolérance**					
Evaluation globale par l'investigateur			X	X	X
Evaluation globale par le patient			X	X	X
Evaluation locale par l'investigateur			X		
Evaluation locale par le patient			X		
Recherche des évènements indésirables		X	X	X	X

9.2 Discussion sur le plan expérimental

L'objectif de l'étude est de démontrer la non infériorité de l'efficacité d'OSTENIL® PLUS par rapport à SYNVISC-ONE™ dans le traitement de la gonarthrose fémoro-tibiale symptomatique.

9.3 Population étudiée

9.3.1 Critères de sélection
9.3.1.1 Critères d'inclusion

- Patient homme ou femme âgé de 40 à 85 ans.
- Patient atteint de gonarthrose fémoro-tibiale primitive répondant aux critères de l'ACR (Annexe 1.1) :
  Douleur mécanique du genou
  et au moins un des 3 critères suivants :
  1. Age > 50 ans
  2. Raideur matinale < 30 minutes
  3. Craquements du genou aux mouvements actifs
  et Ostéophytes sur la radiographie des deux genoux de face.
- Patient dont l'arthrose a été définie radiologiquement : pincement de l'interligne articulaire et ostéophyte sur des clichés datant de moins d'un an et stade Ib à III de KELLGREN/LAWRENCE modifié (19) (Annexe 1.2), (c'est-à-dire pincement de l'interligne articulaire compris entre 25% et 75% et ostéophyte certain).
- Patient symptomatique, d'un seul coté, avec un score moyen des échelles de la douleur de WOMAC (section A) ≥ 40 mm, ou bien si gonarthrose bilatérale avec une différence d'au moins 20 mm sur le même score entre le genou controlatéral et celui à évaluer (après wash out pour antalgiques ou AINS) (Annexe 1.7).
- Patient avec douleur présente au moins quinze jours dans le mois précédant l'inclusion.
- Patient nécessitant, un traitement par injection d'acide hyaluronique après échec ou intolérance des antalgiques de première intention et des AINS.
- Patient assuré social.
- Patient apte à comprendre et à suivre les instructions de l'étude.
- Patient ayant signé le consentement éclairé.

9.3.1.2 Critères de non inclusion

Outre les patients ne vérifiant pas l'ensemble des critères d'inclusion cités ci-dessus, les patients présentant au moins un des critères suivants seront exclus :

- Patient ayant une gonarthrose non ou insuffisamment symptomatique.
- Gonarthrose bilatérale symptomatique avec la même intensité des deux cotés.
- Gonarthrose secondaire post traumatique.
- Gonarthrose de stade radiologique I, Ia ou IV.
- Gonarthrose fémoro-patellaire exclusive ou dont la symptomatologie est principalement d'origine fémoro-patellaire (syndrome rotulien).
- Patient atteint de coxarthrose homolatérale symptomatique.
- Déformation en varus ou valgus du genou sélectionné (axe de déformation ≥ 15° sur les radiographies).
- Hydarthrose très importante (à ponctionner) au moment de l'inclusion.
- Patient atteint d'un rhumatisme inflammatoire (PR, rhumatisme psoriasique, chondrocalcinose articulaire, goutte, maladie de Paget, spondylarthrite ankylosante, lupus, etc.).
- Antécédent de traumatisme du genou sélectionné dans les six mois précédant l'inclusion.
- Plaie ou affection cutanée du genou sélectionné.
- Sténose veineuse ou lymphatique du membre inférieur.
- Radiculalgie crurale ou sciatique du membre inférieur à tester.
- Tendinopathie (périarthrite de hanche).
- Sujet ayant fait l'objet d'un traitement par acide hyaluronique intra-articulaire dans le genou sélectionné lors des 6 mois précédant l'inclusion,
- Sujet ayant reçu une injection de corticostéroïde intra-articulaire dans le genou sélectionné lors des 2 mois précédant l'inclusion,
- Sujet dont le traitement par anti arthrosique symptomatique d'action lente et/ou compléments alimentaires à visée anti arthrosique (chondroïtine sulfate, diacérhéine, insaponifiables de soja et d'avocat, oxaceprol, granions de cuivre, glucosamine) a été instauré depuis moins de 3 mois, ou dont la dose a été modifiée dans les 3 derniers mois, précédent l'inclusion.
- Traitement anticoagulant par héparine, warfarine, cependant les anti agrégants plaquettaires tels que aspirine ≤ 325 mg/j, Ticlopidine (TICLID®) ou, le Clopidogrel (PLAVIX®) sont possibles.
- Mise en place d'une prothèse totale dans le genou sélectionné.
- Chirurgie de l'autre genou ou de la hanche ou toute autre chirurgie programmée pendant la durée de l'essai.
- Antécédent de toute intervention chirurgicale arthroscopie, ostéotomie, etc. dans l'année précédent l'inclusion.
- Patient obèse (IMC) ≥ 30 kg/m².
- Sujet ayant des antécédents de maladies auto-immunes.
- Sujet ayant une hypersensibilité connue à l'acide hyaluronique et/ou aux protéines aviaires et /ou au paracétamol.
- Sujet ayant une hypersensibilité connue au mannitol.
- Patient ayant une affection sévère susceptible d'interférer avec l'évaluation, telle que : néoplasie, hémopathie maligne, néphropathie, hépatopathie, ou infection sévère.
- Sujet ayant participé à une investigation en recherche clinique dans les 3 mois précédents.
- Femme enceinte, ou susceptible de le devenir pendant l'essai, ou allaitante.

9.3.2 Patient exclu en cours d'étude et arrêt prématuré de traitement
L'investigateur évaluateur (et) ou injecteur peuvent décider à tout moment, pour chaque patient, de ne pas poursuivre l'essai.
De même, chaque patient peut dénoncer son consentement (Annexe 2.8), et sortir de l'étude à n'importe quel moment.
Les raisons de sortie d'essai peuvent être :
- Symptomatiques (inefficacité, décision de se faire opérer le genou cible, prothèse ou autre, etc.),
- Thérapeutiques (survenue d'évènement (s) indésirable (s), etc.),
- Médicales (survenue d'une affection intercurrente non compatible avec la poursuite de l'essai, etc.),
- Personnelles (disponibilité, démotivation, déplacements, etc.).

Les sorties de l'étude seront notées et documentées.

Dans tous les cas, une évaluation clinique finale devra être effectuée le plus près possible de la date de sortie du sujet.

Les sorties d'études prématurées seront comptées, identifiées et décrites par visite, et le pourcentage entre les deux groupes de traitement sera comparé en utilisant un test du Chi^2.

Les sujets sortis d'étude avant l'injection seront remplacés et non inclus dans l'analyse en intention de traiter.

Les autres sujets sortis prématurément de l'étude ne seront pas remplacés. Aux visites suivantes non effectuées seront prises en compte les dernières données nommées « dernières observations disponibles » (ou en anglais Last Observation Carried Forward ou LOCF).

Les sorties d'étude pour évènement indésirable grave (EIG) feront l'objet d'une notification particulière.

9.4 Traitements

9.4.1 Produits administrés
Le conditionnement des lots de traitements pour l'étude sera effectué par TRB CHEMEDICA.
Les traitements attribués seront les suivants : OSTENIL® PLUS ou SYNVISC-ONE™ après randomisation.

9.4.1.1 Forme pharmaceutique et dosage
Caractéristiques d'OSTENIL® PLUS
Nom commercial : OSTENIL® PLUS
Forme et présentation : Solution viscoélastique pour injection dans la cavité articulaire : Seringue préremplie de 40 mg/2 ml, sous emballage stérile, boite unitaire. Stérilisé par vapeur d'eau.
Composition : Solution isotonique (pH = 7,3) : par ml
Hyaluronate de sodium d'origine fermentative 20 mg
Excipients : chlorure de sodium, phosphate monosodique, phosphate disodique, mannitol, eau ppi.
Fabricant : TRB CHEMEDICA
Posologie : Injecter 1 seringue d'OSTENIL®PLUS dans l'articulation atteinte.
Voie d'administration : intra-articulaire
Condition de conservation : A conserver à température ambiante n'excédant pas 25°C.

Caractéristiques de SYNVISC-ONE™
Nom commercial : SYNVISC-ONE™
Forme et présentation : 6 ml : seringue en verre de 10 ml
Composition par seringue :
Pour 1 ml (Hylane G-F20) : hylane 8.0 mg, chlorure de sodium 8.5 mg, phosphate disodique 0.16 mg, phosphate monosodique hydraté 0.04 mg, eau pour préparations injectables qs.
SYNVISC-ONE contient 6 ml de Hylane G-F 20, acide hyaluronique extrait de crêtes de coq et réticulé.
Fabricant : GENZYME
Posologie : arthrose du genou, une injection
Voie d'administration : intra-articulaire exclusive
Condition de conservation : température comprise entre 2°C et 30°C

9.4.2 Schéma thérapeutique

9.4.2.1 Présentation et étiquetage
Chaque unité thérapeutique est identifiée par un numéro (de 001 à 400) et étiquetée selon les normes en vigueur.
Les traitements OSTENIL® PLUS et SYNVISC-ONE™ seront conditionnés dans un emballage neutre identique.

9.4.2.2 Attribution des traitements
Les lots de traitements numérotés seront attribués à chaque investigateur après une procédure de tirage au sort.
Une fois la liste définitive des investigateurs arrêtée, le tirage au sort désignera l'un des investigateurs de la liste qui recevra les lots de traitements N° 001 à N° 004.
L'investigateur immédiatement suivant sur la liste recevra les lots suivants soit N° 005 à N° 008, et ainsi de suite jusqu'au dernier investigateur.
Les traitements nécessaires seront remis aux investigateurs injecteurs lors de la mise en place de l'essai.
Chaque médecin injecteur recevra 4 à 8 lots.
Les lots de traitements seront utilisés selon l'ordre croissant de numérotation.

9.4.2.3 Aveugle
La liste de randomisation sera établie par le promoteur de l'étude.
Les patients seront randomisés entre les deux groupes de traitement OSTENIL® PLUS et SYNVISC-ONE™ selon une procédure de randomisation informatisée, par bloc de quatre traitements.
Les traitements seront conditionnés dans un emballage neutre identique.
Afin de respecter l'équilibre entre les groupes, chaque investigateur s'engage à recruter 4 patients.
Chaque investigateur évaluateur inclura les patients selon l'ordre de randomisation en commençant par le plus petit numéro pour le premier patient et ainsi de suite.
Chaque médecin injecteur pratiquera une injection du produit selon le même numéro de traitement en commençant par le plus petit numéro pour le premier patient et ayant soin de vérifier qu'il correspondait bien au numéro indiqué par le médecin évaluateur.
Il procédera de même pour les patients suivants et ce selon l'ordre croissant de la numérotation.
Chaque investigateur recevra pour chaque lot une enveloppe de « type inviolable », sur laquelle figurera le numéro du patient.
Dans chaque enveloppe le numéro du patient et son groupe d'appartenance seront indiqués.
En cas de nécessité l'investigateur pourra en le justifiant procéder à l'ouverture de l'enveloppe concernée. Toutes les enveloppes de randomisation seront collectées en fin d'essai.
La viscosité, le volume et la taille de la seringue d'OSTENIL® PLUS étant différents de ceux de la solution SYNVISC-ONE™ et donc facilement identifiables aussi la procédure de double aveugle nécessitera que le médecin évaluant les patients au plan clinique ne soit pas le médecin injecteur, de sorte que ni le patient ni le médecin évaluateur ne sachent quel produit a été administré.

9.4.2.4 Stockage et gestion des traitements

Les traitements devront être entreposés dans un lieu bien défini par l'investigateur garantissant leur bonne conservation et leur inaccessibilité à des tiers.

Ces traitements devront être utilisés uniquement dans le cadre de cette étude.

Pendant toute la durée de l'essai, l'Attachée de Recherche Clinique de la société en charge du monitoring pourra avoir accès aux traitements pour vérification.

A la fin de l'essai, tous les traitements utilisés ou non utilisés seront rendus à l'Attaché de Recherche Clinique de la société en charge du monitoring.

9.4.3 Choix de la dose pour l'étude

Celle préconisée à savoir, 1 injection.

L'injection intra-articulaire d'OSTENIL® PLUS ou de solution SYNVISC-ONE™ selon le groupe de randomisation, sera réalisée au cabinet du médecin injecteur, selon le respect des règles habituelles d'asepsie pour les infiltrations intra-articulaires, telles celles énumérées par le consensus du GRRIF (Groupe de Recherche en Rhumatologie Interventionnelle Français):

« L'opérateur n'est pas systématiquement ganté, le masque n'est nécessaire que si le médecin est porteur d'une infection ORL,

Le lavage des mains et des ongles avec une solution antiseptique est impératif, soigneux voir méticuleux,

La peau du patient est nettoyée mais non rasée (éventuellement tondue ou épilée à la crème), désinfectée avec des compresses stériles imprégnées d'un dérivé iodé type bétadine alcoolique, qu'il faut laisser sécher 3 minutes en moyenne,

En cas d'allergie à l'iode, il faut utiliser un autre antiseptique mais éviter les ammoniums quaternaires qui peuvent interagir avec l'acide hyaluronique (AH),

Après l'injection, un pansement propre, voire stérile est mis en place » (22).

L'injection devra préférentiellement être réalisée par voie externe, après repérage clinique du point d'injection.

9.4.4 Traitements antérieurs et concomitants

9.4.4.1 Traitements antérieurs

Tout traitement pris par le patient au moment de l'inclusion dans l'essai sera noté sur le cahier d'observation à la visite initiale.

Si le patient ne prend aucun AINS ou antalgique il pourra être inclus dans l'étude sous réserve de satisfaire aux autres critères d'inclusion ou de non inclusion.

Si le patient est sous AINS et (ou) antalgiques un sevrage sera nécessaire (annexe 1.7).

En cas de dérivés par oxicam le sevrage sera de 10 à 15 jours.

Pour tous les autres anti-inflammatoires il sera de 4 jours.

En cas de traitement par paracétamol un sevrage de 24 heures sera observé avant l'inclusion.

9.4.4.2 Traitements associés

Les traitements prescrits en cours d'essai pour une autre affection que la gonarthrose seront autorisés si nécessaire.

Ils seront détaillés et notés dans le cahier d'observation lors des visites de suivi.

Sont autorisés notamment :
Les AINS en brève administration (3 à 5 jours par mois), ceux-ci seront arrêtés au moins 5 demi-vies avant les visites d'évaluation (annexe 1.7), on privilégiera les AINS à demi vie courte,
Les AINS topiques (sevrage 2 jours),
Les corticoïdes per os (sevrage 2 jours),
Les anti-agrégants plaquettaires : Aspirine (avec un maximum quotidien de 325 mg), Ticlopidine (TICLID®) et Clopidogrel (PLAVIX®),
Les traitements par physiothérapie (ultrasons, électrothérapie, ionisations),
Les médecines alternatives (acupuncture, homéothérapie, mésothérapie),
Les crénothérapies,
La kinésithérapie.

Les traitements intercurrents suivants ne seront pas autorisés pendant toute la durée de l'essai :
Les traitements anti arthrosique symptomatique d'action lente et/ou compléments alimentaires à visée anti arthrosique (chondroïtine sulfate, diacérhéine, insaponifiables de soja et d'avocat, oxaceprol, granions de cuivre, glucosamine).
Les injections intra articulaire d'acide hyaluronique,
Les injections intra articulaire de corticoïdes,
Tout traitement, qui de l'avis de l'investigateur pourrait interférer avec l'efficacité du traitement à l'essai ou avec l'interprétation des critères de jugement.
En cas de doute sur la possibilité de prendre tel traitement le comité scientifique sera consulté.

9.4.4.3 Traitements interdits
Sont interdits jusqu'à la visite de fin d'essai à 6 mois pour le traitement de la gonarthrose :
Les traitements anti arthrosique symptomatique d'action lente et/ou compléments alimentaires à visée anti arthrosique (chondroïtine sulfate, diacérhéine, insaponifiables de soja et d'avocat, oxaceprol, granions de cuivre, glucosamine), sauf si ceux-ci ont été instaurés depuis au moins 3 mois de façon stable et dont leurs doses restent constantes, ils devront être poursuivis jusqu'à la fin de l'essai.
Les AINS y compris les topiques,
Les injections intra articulaire d'acide hyaluronique ou de corticoïde,
Les corticoïdes per os,
Les traitements locaux : lavage intra-articulaire, ponction (sauf en cas d'hydarthrose importante la nécessitant),
Les traitements de physiothérapie (cependant si ceux-ci ont été instaurés depuis au moins 1 an, et de façon stable, ils pourront être continués),
Les médecines alternatives.

En cas de nécessité la prise de paracétamol jusqu'à 4g/jours sera autorisée, sa consommation sera notée sur le cahier patient et reportée sur le cahier d'observation à chaque visite (elle sera utilisée comme critère accessoire d'efficacité). Un wash-out de 12h00 sera respecté avant les visites de suivi.

En cas de prise de traitement interdit le patient sera sorti d'essai.

9.4.5 Observance des patients
Elle sera appréciée par la réalisation de l'injection.

9.5 Evaluation de l'efficacité et de la tolérance

9.5.1 Critères d'évaluation
9.5.1.1 Critère principal d'évaluation
9.5.1.1.1 Efficacité
Le critère principal est la variation du score moyen des échelles de la douleur de WOMAC (section A) de J0 à J180 (Annexe 1.4).

9.5.1.1.2 Tolérance
On étudiera les proportions de sujets présentant ou non un ou plusieurs évènements indésirables pendant la durée de l'essai.

9.5.1.2 Critères secondaires d'évaluation
9.5.1.2.1 Efficacité
Les critères secondaires d'efficacité seront les critères cliniques et leur évolution pendant les 6 mois de suivi :

- Scores moyens des échelles de la raideur et de la fonction physique du WOMAC (section B et C),
- L'indice de LEQUESNE (Annexe 1.3),
- Evaluation de l'efficacité globale du traitement par le patient et l'investigateur,
- Cotation du statut global du patient en relation avec sa gonarthrose sur EVA,
- Consommation d'antalgiques et d'AINS,
- Pourcentage de patients n'ayant pas eu besoin de recourir à une thérapie locale,
- Pourcentage de patients répondeurs selon les critères de l'OMERACT-OARSI (23) (Annexe 1.5).

9.5.1.2.2 Tolérance
La tolérance globale est évaluée à C3, C4 et C5 :
- Par le jugement du patient et de l'investigateur selon une échelle verbale,
- Par le recueil de l'existence d'évènements indésirables.

La tolérance locale est évaluée à la consultation C3 par le jugement du patient et de l'investigateur selon une échelle verbale ainsi que par le recueil de manifestations telles que : douleur post-injection, réaction inflammatoire, présence ou non d'une hydarthrose, présence d'une arthrite aigue pseudo septique ou septique.
Tous les événements latéraux survenant au cours de cette étude seront portés sur le cahier d'observation.

9.5.2 Pertinence des critères d'évaluation

9.5.2.1 Critère principal d'efficacité

WOMAC : index de sévérité symptomatique de l'arthrose des membres inférieurs.

Le WOMAC est l'index validé dans l'évaluation de l'arthrose des membres inférieurs. Il existe 2 systèmes de cotation des réponses aux questions : soit l'échelle de LICKERT avec 5 réponses possibles (nulle = 0 ; minime = 1 ; modérée = 2 ; sévère = 3 ; extrême = 4), soit une échelle visuelle analogique de 100 mm. Il est possible de calculer les scores dans chaque domaine ou pour l'ensemble du WOMAC

Les moyennes des sous-scores du WOMAC seront calculées en additionnant les 5 items relatifs à la douleur, les 2 items relatifs à la raideur, et les 17 items relatifs à la fonction physique, divisé par le nombre de questions s'y rapportant de façon à obtenir une valeur comprise entre 0 et 100 pour chaque sous-score.

- Une douleur légère à modérée est définie par un score moyen de 25 mm à 60 mm
- Une douleur modérée à sévère est définie par un score moyen > à 60 mm

WOMAC Section A Domaine douleur : **quelle est l'importance de la douleur ?**
1. Lorsque vous marchez sur une surface plane ?
2. Lorsque vous montez ou descendez les escaliers ?
3. La nuit, lorsque vous êtes au lit ?
4. Lorsque vous vous levez d'une chaise ou vous asseyez ?
5. Lorsque vous vous tenez debout ?

WOMAC Section B Domaine raideur : **quelle est l'importance de la raideur de votre articulation ?**
1. Lorsque vous vous levez le matin ?
2. Lorsque vous bougez après vous être assis, couché ou reposé durant la journée ?

WOMAC Section C Domaine fonction : **quelle est l'importance de la difficulté que vous éprouvez à ?**
1. Descendre les escaliers ?
2. Monter les escaliers ?
3. Vous relever de la position assise ?
4. Vous tenir debout ?
5. Vous pencher en avant ?
6. Marcher en terrain plat ?
7. Entrer et sortir d'une voiture ?
8. Faire vos courses ?
9. Enfiler collants ou chaussettes ?
10. Sortir du lit ?
11. Enlever vos collants ou vos chaussettes ?
12. Vous étendre sur le lit ?
13. Entrer ou sortir d'une baignoire ?
14. Vous asseoir ?
15. Vous asseoir et vous relever des toilettes ?
16. Faire le ménage " à fond " de votre domicile ?
17. Faire l'entretien quotidien de votre domicile ?

9.5.3 Critères d'évaluation de la tolérance clinique

9.5.3.1 Evènements indésirables

Le terme « évènement indésirable » indique toute manifestation nocive et non recherchée subie par une personne participant à une recherche biomédicale, quelle que soit la cause de cette manifestation.
Tout évènement découvert par l'investigateur ou spontanément rapporté par le patient sera décrit dans le cahier d'observation sur les formulaires réservés à cet effet.
En cas de survenue de tout évènement médical grave, inattendu ou menaçant le pronostic vital du patient, considéré ou non comme lié au traitement, le laboratoire TRB CHEMEDICA ainsi que la CRO SPRIT devront être immédiatement contactés.

Est qualifié de grave, tout évènement dont l'évolution est fatale ou susceptible de mettre la vie en danger, ou entraînant une invalidité ou une incapacité, ou provoquant une hospitalisation ou une prolongation d'hospitalisation.
Les évènements indésirables graves devront être signalés dans les 24 heures par téléphone ou par fax au promoteur. Cette déclaration sera confirmée par la transmission immédiate de la page « Fiche de pharmacovigilance » dûment complétée.
Tout décès devra être signalé par téléphone ou par fax au promoteur, dès que l'investigateur en aura eu connaissance.
Dans les trois jours suivants, un rapport détaillé écrit devra être envoyé au promoteur (Annexe 1.16).

Un patient pourra sortir de l'étude pour évènement indésirable de sa propre initiative ou sur l'initiative de l'investigateur.
Dans tous les cas, la raison pour laquelle un patient sort de l'étude devra être clairement détaillée.
Dans la mesure du possible, une dernière évaluation devra être réalisée à la date de sortie d'essai ou dès que le patient pourra être revu.

9.5.3.2 Etude de la tolérance

Elle sera purement descriptive.
Les effets secondaires seront catégorisés au moyen de la classification MedDRA qui est un catalogue de termes médicaux développé par la conférence internationale d'harmonisation ou ICH.
Elle sera effectuée sur tous les patients ayant reçu l'injection au traitement.
Elle reposera sur l'étude des évènements indésirables survenus (nombre, nature, durée, évolution) et sur les sorties d'étude pour intolérance.
Tout évènement indésirable sera suivi jusqu'à sa résolution.

9.6 Rapport final

A l'issue de l'analyse statistique, un rapport complet de l'étude sera rédigé et signé après approbation par le coordonnateur, le promoteur, le responsable statistique, le conseiller scientifique, et le gestionnaire de l'essai.

Le promoteur, le coordonnateur national et les investigateurs s'interdisent de divulguer, de quelque manière que ce soit, les résultats complets de l'étude sans l'autorisation formelle de chacune des parties.

9.7 Méthodes statistiques prévues et calcul du nombre de sujets

9.7.1 Effectif choisi
Dans le but de montrer une réduction de la douleur spontanée globale durant les dernières 24 heures sur une EVA 100 mm entre la visite d'inclusion et la visite de suivi à 6 mois entre le groupe d'OSTENIL® PLUS et le groupe SYNVISC®, **111 sujets au moins** devront être recrutés dans chaque groupe de traitement.

9.7.2 Paramètres utilisés pour le calcul du nombre de sujets
Ce nombre de sujet est basé sur les hypothèses suivantes :

Erreur de type I	$\alpha = 0.05$
Erreur de type II	$\beta = 0.20$
Différence cliniquement signifiante	$\Delta = 8$ mm
Ecart-type	$\sigma = 24$ mm

$$n = \frac{2\sigma^2}{\Delta^2}(z_{1-\alpha} + z_{1-\beta})^2$$

L'objectif de l'étude est de mettre en évidence la non infériorité d'OSTENIL® PLUS comparativement au produit de référence (selon les recommandations de l'EMEA :
http://www.emea.eu.int/pdfs/human/ewp/048299en.pdf

Le nombre de sujets à inclure sera donc estimé en approche unilatérale.
L'effectif théorique calculé à partir de ces données est de 111 sujets par groupe.
Anticipation d'un taux de perte de ± 15% à M6
Compte tenu des patients éventuellement "perdus de vue", le nombre total de 260 patients est retenu.

9.7.3 Evaluation statistique

9.7.3.1 Méthode de l'analyse statistique
Un plan d'analyse statistique détaillé sera préparé et validé par le comité scientifique.

9.7.3.1.1 Revue des données en aveugle
Description de l'échantillon (en fonction du type des paramètres), des violations du protocole, des données aberrantes ou manquantes.
Demandes de décisions au coordinateur et au comité scientifique de l'essai, en particulier pour le classement des violations au protocole en « majeure » et « mineure ».
A l'issue de cette revue des données en aveugle les populations suivantes seront définies, population en ITT et population PP.
Propositions éventuelles, en fonction des résultats de cette analyse, de modifications du plan et (ou) des méthodes d'analyse, ou de la définition de critères, faisant l'objet d'un amendement au protocole.

9.7.3.1.2 Description de l'échantillon
En fonction des paramètres, moyennes et déviations standard, médianes et extrêmes, effectifs des classes.

9.7.3.1.3 Comparaison des groupes avant traitement
Elle sera étudiée à J 0 par les tests statistiques classiques :
Test de Chi^2 pour les variables qualitatives,
Test de Student et analyse de variance pour les variables quantitatives.

9.7.3.2 Critères de jugement

9.7.3.2.1 Critère principal de l'efficacité
L'analyse principale portera sur la population per protocole (PP), l'analyse secondaire sur la population ITT en Intention de Traiter (afin de s'assurer de la stabilité des résultats obtenus).
Les moyennes des différences du score de WOMAC entre l'inclusion et la visite à 6 mois seront calculées pour chacun des groupes.
Pour que OSTENIL® PLUS soit non inférieur à SYNVISC-ONE™ la borne inférieure de l'intervalle de confiance à 95% de la différence (entre OSTENIL® PLUS et SYNVISC-ONE™) des moyennes des échelles de WOMAC doit être supérieure à la valeur de - 8 mm (cf schéma ci-dessous).
La zone d'équivalence sera donc constituée par l'intervalle de confiance à 95% de la différence (supposée nulle) de + 8 mm à – 8 mm.

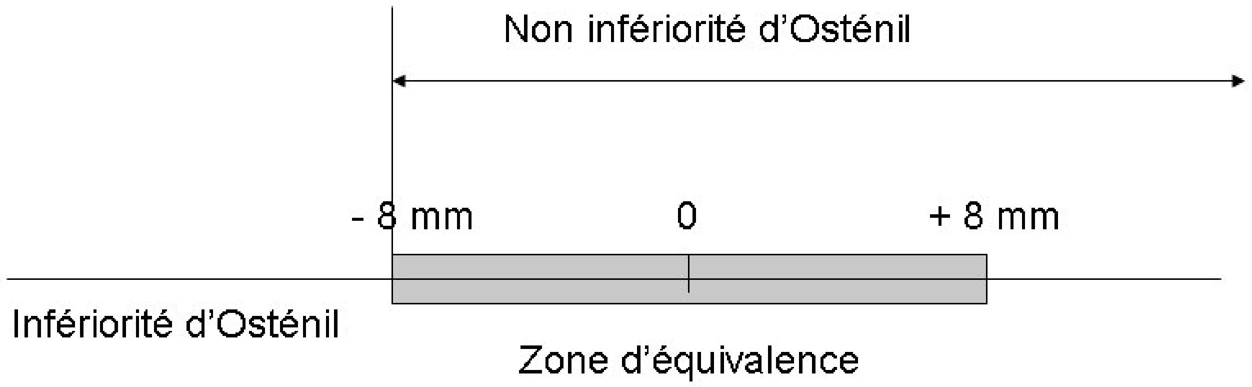

Afin de limiter tout risque de biais lors des analyses, les données manquantes feront l'objet d'un traitement spécifique.
Dans un premier temps, une analyse comparative des patients perdus de vue sera réalisée afin de mesurer les biais potentiels et d'identifier d'éventuelles covariables sur lesquelles des différences importantes auront été notées.
Puis dans un second temps sous l'hypothèse que les données manquantes sont de type MAR (24) une imputation des données manquantes sera réalisée à l'aide de la PROC MI (Multiple Imputation sous SAS) ou à l'aide du logiciel STATA.
L'analyse sous LOCF, même sous l'hypothèse que les données manquantes sont MCAR (24) n'est probablement pas correcte dans cette étude.
Les résultats avec imputation seront comparés à ceux obtenus sans imputation afin de juger du biais induit par ces données manquantes.

9.7.3.2.2 Critères secondaires
L'aire sous la courbe de l'indice algo-fonctionnel de LEQUESNE sera également comparée entre les groupes, à l'aide d'un test t de STUDENT pour données indépendantes ou d'un test de WILCOXON (en fonction de la normalité de la distribution de la variable observée).

L'évolution de l'indice algo-fonctionnel de LEQUESNE entre l'inclusion et la fin de la période de suivi (ou de la dernière observation recueillie) sera testée dans chacun des deux groupes en utilisant un test t de STUDENT pour données appariées puis comparée entre les deux groupes à l'aide d'un test t de STUDENT pour données indépendantes ou d'un test de WILCOXON (en fonction de la normalité de la distribution de la variable observée).

L'appréciation globale par le patient de son état en relation avec sa gonarthrose cotée sur EVA de 0 à 100 mm, sera comparée entre les groupes à l'aide d'un test t de STUDENT pour données indépendantes ou d'un test de WILCOXON (en fonction de la normalité de la distribution de la variable observée).
L'évaluation globale de l'efficacité par le patient et l'investigateur,
Les variations des critères qualitatifs (OMERACT-OARSI) seront analysées.

9.7.3.2.3 Critère de jugement de la tolérance
Il reposera sur l'évaluation de la tolérance globale et locale par le patient et l'investigateur.
Les évènements indésirables seront étudiés sur la population randomisée.
Les évènements indésirables seront classés selon la classification WHO par système/organe, et seront comptés et décrits par visite.
On s'intéressera aux évènements indésirables apparus durant l'essai, qu'ils aient ou non entraîné un arrêt prématuré et quelque soit leur imputabilité ou leur sévérité.
Le nombre d'évènements indésirables sera calculé.

Une comparaison entre les groupes sera réalisée à l'aide d'un test du Chi^2 pour les variables suivantes :

<u>Incidence globale</u>
Nombre de sujets présentant au moins un événement indésirable / Nombre de sujets inclus.

<u>Incidence par événement</u>
Nombre de sujets présentant l'événement indésirable considéré / Nombre de sujets inclus.
Une description de la sévérité des évènements sera également réalisée dans chaque groupe.

9.7.3.3 Analyses statistiques
L'analyse principale est l'analyse per protocole (PP), l'analyse secondaire est l'analyse ITT.
Les items du cahier d'observation seront entrés dans la base de données de l'étude par double saisie, effectuée par deux opérateurs différents. Ces deux bases de saisie seront ensuite comparées et les différences seront systématiquement vérifiées. De plus, 10% de ces cahiers seront intégralement vérifiés par rapport à la base de saisie corrigée.
Les données seront ensuite vérifiées par la programmation de contrôles d'incohérences et l'édition de listings de données individuelles, les corrections évidentes seront effectuées et les autres erreurs ou données manquantes feront l'objet de demandes de clarification auprès des investigateurs.
Lorsque la base de données sera déclarée complète et valide, elle sera gelée. Tout changement dans cette base de données après ce point devra faire l'objet d'une demande écrite par le personnel autorisé. Les données seront anonymisées.

Toutes les analyses seront effectuées en utilisant le logiciel SAS et éventuellement le logiciel STATA pour l'imputation multiple des données manquantes.
Toutes les données seront listées.
Toutes les tables statistiques seront présentées par groupe de traitement après le début du traitement, et par groupe de traitement pour la population totale à l'inclusion. Si cela s'avère approprié, les données seront également présentées par centre.
Les analyses statistiques seront effectuées en utilisant des statistiques descriptives, des tests d'hypothèse et des intervalles de confiance.

La comparabilité initiale des groupes sera effectuée, concernant la démographie, les critères d'évaluation de l'efficacité, la prise ou non de traitements symptomatiques de la gonarthrose ainsi que les paramètres de sécurité :
Pour les variables quantitatives, un test de normalité sera d'abord effectué sur la population totale, et dépendamment de la normalité, un test t de STUDENT ou un test non paramétrique (test de WILCOXON) sera utilisé pour comparer les deux groupes.
Pour les variables ordinales, un test de MANN-WHITNEY sera réalisé.
Pour les variables qualitatives, la comparaison sera effectuée en utilisant soit un test du Chi^2, soit un test exact de FISHER.
Les données quantitatives seront caractérisées par au moins : le nombre de données non manquantes, le nombre de données manquantes, la moyenne, la médiane, l'écart-type, le minimum et le maximum.
Les données qualitatives seront caractérisées par la fréquence et le pourcentage par modalité, et par le nombre de données manquantes.
Pour tous les tests effectués, l'approche sera bilatérale et le risque de première espèce fixé à 5%.
La robustesse des conclusions de l'étude sera analysée en testant l'influence des paramètres pour lesquels la différence entre les deux groupes sera statistiquement significative à l'inclusion (analyse de la covariance et régressions multiples et/ou ajustement, en fonction de la nature des paramètres).

9.7.3.3.1 Définition des populations analysées
Les populations seront définies selon la norme ICH Topic E 9 Statistical Principles for Clinical Trials EMEA septembre 1998, chapitre 5.2 (25).

9.7.3.3.1.1 Population en intention de traiter (ITT)
Les patients retenus dans la population en intention de traiter seront caractérisés par les critères suivants :

Pour la comparabilité initiale et la tolérance : Tout patient inclus (c.-à-d. randomisé) dans l'essai et ayant reçu l'injection intra-articulaire du produit à l'essai appartiendra à la population en intention de traiter pour la tolérance et la comparabilité initiale entre les deux groupes.

Pour l'efficacité :
- Tout patient randomisé dans l'essai
- ayant reçu l'injection du produit à l'essai
- ayant au moins une mesure du critère principal d'évaluation d'efficacité (WOMAC A) lors du suivi
- ne présentant pas de déviation grave aux critères de sélection (les déviations graves seront définies précisément par le comité scientifique lors de la revue des données en aveugle).

Le fait d'exclure de ce groupe les patients qui ne reçoivent pas d'injection se justifie parce que la raison pour laquelle un patient ne recevrait pas d'injection ne dépend pas de la connaissance du traitement assigné (puisque les deux traitements sont du même type).

Les patients qui recevraient une injection du produit de l'autre groupe que celui auquel ils avaient été assignés seront analysés selon ce dernier groupe (et non selon le produit effectivement reçu).

9.7.3.3.1.2 Population per protocole (PP)
La population per protocole comprendra les patients du groupe ITT qui présentent en plus :
- l'absence de déviations majeures au protocole (les déviations majeures seront définies précisément par le comité scientifique lors de la revue des données en aveugle
- la réalisation de la mesure du WOMAC A à J180.

10. CALENDRIER

Mise en place de l'essai	Mai 2011
Fin des inclusions	Décembre 2012
Fin de l'essai	Juin 2013
Analyses statistiques et résultats	Septembre 2013
Rapport clinique	Décembre 2013

PARTIE II : PRINCIPES GENERAUX

1. CONSIDERATIONS ETHIQUES

1.1 Déclaration d'Helsinki

La déclaration d'Helsinki (Annexe 2.1), dans sa révision la plus récente, est acceptée comme base éthique des études cliniques et doit être pleinement suivie et respectée par tous les intervenants à un protocole sur les personnes.

Toute exception doit être justifiée et mentionnée dans ce même protocole.

1.2 Bonnes Pratiques Cliniques

Les études cliniques doivent être réalisées selon les Bonnes Pratiques Cliniques (Annexe 2.4). Par étude clinique on entend la conception, la conduite, le monitoring, la clôture, l'audit, les analyses, les rapports et la documentation des études.

Les Bonnes Pratiques Cliniques garantissent que les études sont éthiquement et scientifiquement justifiées, et que les propriétés cliniques des produits à l'essai à visée diagnostique, thérapeutique ou prophylactique sont correctement documentées.

2. RESPONSABILITES DES INVESTIGATEURS

Il est de la responsabilité des investigateurs de réaliser l'essai selon le protocole et de s'assurer qu'ils ont le recrutement nécessaire pour réaliser l'étude dans les délais définis par le protocole d'étude.

2.1 Ethique

2.1.1 Déclaration d'Helsinki
L'étude se déroulera en conformité avec les principes de l'éthique médicale, tels qu'ils sont définis par la déclaration d'Helsinki, révisée en 2008.

2.1.2 CPP
L'avis favorable préalable du CPP doit être obtenu avant le début de l'étude (Annexe 2.5).
Il doit être notifié par écrit au coordinateur.
Une copie doit être adressée au promoteur.
Une copie figure en annexe du protocole.

2.1.3 Information au patient
Chaque patient doit être informé de manière complète et sans ambiguïté qu'il est libre de refuser de participer à l'étude ou qu'il peut dénoncer son consentement à tout moment et ce, pour quelque raison que ce soit, sans encourir de préjudices quant aux soins qu'il doit ou devra recevoir de la part de l'investigateur en raison de son état de santé.

Une notice d'information sera remise au patient (Annexe 2.7).

2.1.4 Consentement éclairé du patient
Il est de la responsabilité des investigateurs d'obtenir le consentement éclairé écrit de chaque patient participant à l'étude (Annexe 2.8), après lui en avoir correctement expliqué les buts, les méthodes, les bénéfices et les risques potentiels.
Le consentement doit être obtenu avant que toute procédure spécifique à l'étude soit entreprise.
Un double du consentement est remis au patient.
Pour les patients qui pour quelque raison que ce soit ne peuvent donner leur consentement éclairé écrit, celui-ci devra être obtenu du tuteur légal.
Le consentement éclairé signé doit être archivé par l'investigateur et documenté dans le cahier d'observation et le dossier médical du patient.

2.1.5 Retrait d'un sujet
L'investigateur a le droit de retirer un sujet de l'étude pour quelque raison que ce soit, si c'est l'intérêt du patient (y compris en cas de maladie intercurrente, évènements indésirables ou échec du traitement).
Chaque fois qu'un patient est sorti d'essai pour quelque raison que ce soit, une évaluation de fin d'essai devra être effectuée en mentionnant la raison du retrait.
Les retraits pour non participation doivent être suivis afin d'en identifier les raisons.
Les retraits pour maladie intercurrente ou évènements indésirables doivent être documentés de manière complète dans le cahier d'observation, avec toute l'information complémentaire disponible et adéquate.

2.1.6 Anonymat du sujet
Les investigateurs doivent s'assurer que l'anonymat du patient est préservé.
Sur tous les documents qui seront remis au promoteur, le patient ne doit être identifié que par un code d'identification, et non par son nom ou son numéro de dossier hospitalier.
Les investigateurs doivent tenir un formulaire d'inclusion confidentiel qui établi un lien entre le code d'identification et les noms/adresses des patients.
Les documents qui ne seront pas remis au promoteur seront détenus par les investigateurs en stricte confidentialité.

2.1.7 Frais
Seront pris en charge par TRB CHEMEDICA promoteur de cette étude :

- Les honoraires des médecins investigateurs liés aux consultations de l'étude
- Les traitements liés à l'étude

2.2 Amendements au protocole

Conformément aux articles L 1123-9 et R 1123-35 du code de la santé publique toute modification substantielle de la recherche à l'initiative du promoteur doit obtenir préalablement à sa mise en œuvre, un avis favorable du comité de protection des personnes et/ou une autorisation de l'AFSSAPS en fonction des aspects de la recherche qui sont modifiés.

Chaque amendement au protocole sera signé par le coordonnateur et le promoteur.

L'avis favorable du CPP et/ou l'autorisation de l'AFSSAPS pour chaque amendement sera notifié par écrit au coordinateur, à TRB CHEMEDICA, et à chaque investigateur.

2.3 Cahier d'observation

Le cahier d'observation (Annexe 1.12) tripliqué est conçu de manière à y recueillir toutes les données nécessaires à l'analyse de l'étude, y compris les antécédents des patients, ainsi que les éventuels évènements indésirables.
La transcription des données dans le cahier sera réalisée à la main et de préférence au stylo à bille bleu.
En cas de modification ou d'ajout apporté au cahier d'observation, l'investigateur devra barrer d'un seul trait la donnée initiale, écrire la nouvelle donnée à côté, puis parapher et dater du jour du paraphe.
Les mentions suivantes seront utilisées : DM donnée manquante, NA donnée non applicable, NC donnée non connue, NF non fait.

2.4 Vérification des documents sources

Toutes les données obtenues au cours d'un essai clinique doivent être traitées confidentiellement afin de garantir les droits du patient à la confidentialité de sa vie privée.

Les investigateurs s'engagent à accepter que le moniteur, l'auditeur, l'inspecteur aient accès à tout document nécessaire à la vérification des données sources et au suivi approprié de l'avancement de l'étude.
Si la vérification directe des documents sources n'était pas autorisée par la loi, alors l'investigateur s'engage à assister le moniteur, l'auditeur, l'inspecteur dans le processus de validation de la qualité des données.

2.5 Evènements indésirables

2.5.1 Définition
L'expression indique toute manifestation nocive et non recherchée subie par une personne participant à une recherche biomédicale quelle que soit la cause de cette manifestation.

Cet évènement indésirable est grave lorsque :

- Il présente un risque pour la vie du patient ou entraîne une issue fatale et/ou provoque un handicap permanent grave.
- Il entraîne l'hospitalisation du patient ou la prolongation de son hospitalisation.
- Il y a apparition d'une malformation congénitale ou maligne.
- Il est lié à une overdose accidentelle ou intentionnelle.
- Il semble indiquer un surdosage imminent, une contre-indication, une précaution particulière ou un évènement indésirable grave.

Lien de causalité :
Exclu :
Doit être réservé pour les évènements qui se produisent avant l'administration du médicament (par exemple, période de sevrage) ou pour les évènements qui ne peuvent être, même vaguement, en relation avec la participation à l'étude (par exemple, blessures subies dans un accident de voiture).

Improbable :
Du fait de la relation chronologique entre le début du traitement et la date de survenue de la manifestation associée indésirable (potentiellement un évènement indésirable).

Possible :
Relation chronologique raisonnable par rapport au début du traitement.
Relation possible avec l'état clinique du patient ou avec un autre traitement.

Probable :
Relation chronologique raisonnable par rapport au début du traitement.
Disparition de la manifestation associée indésirable après l'arrêt du traitement par le produit suspecté.
Absence d'explication évidente et raisonnable compte tenu de l'état clinique connu du patient.

Certain :
Relation chronologique raisonnable par rapport au début du traitement.
Disparition de la manifestation associée indésirable après l'arrêt du traitement par le produit suspecté.
Après reprise du traitement par le produit suspecté, réapparition de la manifestation associée indésirable.

Tout évènement découvert par l'investigateur ou spontanément rapporté par le patient sera décrit dans le cahier d'observation sur les formulaires réservés à cet effet.
En cas de survenue de tout évènement médical grave, inattendu ou menaçant le pronostic vital du patient, considéré ou non comme lié au traitement, TRB CHEMEDICA et SPRIT devront être immédiatement contactés.

2.5.1.1 Evènements indésirables graves
Est qualifié de grave tout évènement dont l'évolution est fatale ou qui est susceptible de mettre la vie en danger, ou qui entraîne une invalidité ou une incapacité ou qui provoque une hospitalisation ou une prolongation d'hospitalisation.

2.5.1.2 Rapport des évènements indésirables graves
Les évènements indésirables graves devront être signalés dans les 24 heures par téléphone ou par fax au promoteur TRB CHEMEDICA ainsi qu'à SPRIT.

Cette déclaration doit être confirmée par la transmission immédiate de la page « Rapport d'événement indésirable grave (EIG) » dûment complétée.

Conformément à l'article R 1123-48 du code de la santé publique, les suspicions d'effets indésirables graves et inattendus et les évènements indésirables graves pouvant être liés au geste de mise en œuvre du dispositif à étudier devront être déclarés par le promoteur dès qu'il en aura eu connaissance et au plus tard dans les 15 jours calendaires qui suivent la date à laquelle il en aura été informé, à l'AFSSAPS et au comité de protection des personnes.

Quelque soit le délai de survenue après la fin de l'essai, tout EIG susceptible d'être dû à la recherche doit être déclaré lorsqu'il est porté à la connaissance du promoteur et qu'aucune autre cause que la recherche ne peut être raisonnablement incriminée.

2.5.1.3 Décès
Tout décès doit être signalé par téléphone ou par fax au promoteur TRB CHEMEDICA et à SPRIT dès que l'investigateur en aura eu connaissance. Dans les trois jours suivants, un rapport détaillé écrit doit être envoyé à la direction médicale de TRB CHEMEDICA.
Les rapports de décès ou d'événements susceptibles de mettre la vie en danger seront déclarés par le promoteur dès qu'il en aura eu connaissance et, au plus tard, dans les 7 jours calendaires qui suivent la date à laquelle il aura été informé.
Un rapport complémentaire détaillé écrit sera adressé aux autorités compétentes dans un délai de 15 jours à compter de la date de première information.

2.5.1.4 Sorties d'étude
Un patient pourra interrompre l'essai pour cause d'évènement indésirable à tout moment, de sa propre initiative ou sur l'initiative de l'investigateur.

Dans tous les cas, la raison pour laquelle un patient sort de l'étude devra être clairement détaillée sur la page de fin d'étude et, dans la mesure du possible, une dernière évaluation sera réalisée à la date de sortie d'essai ou dès que le patient pourra être revu.

2.5.1.5 Evénements indésirables non graves
Les évènements indésirables non mentionnés dans les définitions ci-dessus ne sont pas graves et peuvent être classés comme étant d'intensité :
Légère :
Apparition de symptômes facilement tolérés et ne nécessitant pas de traitement.

Modérée :
Inconfort suffisant pour perturber les activités habituelles (un jour au moins de traitement correcteur est nécessaire).

Sévère :
Apparition d'un risque significatif entraînant :
- Des contre-indications au traitement,
- Ou nécessitant des précautions particulières,
- Ou induisant une impossibilité de mener à bien les activités habituelles,
- Ou nécessitant des contre-mesures importantes,
- Ou nécessitant l'interruption du traitement expérimenté.

2.5.1.6 Traitement et suivi des évènements indésirables
Les évènements indésirables doivent être documentés et suivis jusqu'à ce que l'évènement soit ou résolutif, ou expliqué de manière adéquate, même après que le sujet ait achevé le traitement à l'essai.

2.6 Publications

TRB CHEMEDICA se réservent le droit d'examiner tous les manuscrits et (ou) résumés des résultats de l'essai avant leur soumission pour publication ou présentation.
Cette disposition n'est pas destinée à restreindre ou empêcher la publication ou présentation mais de permettre à TRB CHEMEDICA de protéger des informations brevetées et (ou) d'apporter des commentaires sur la base d'informations qui pourraient ne pas être disponibles pour les investigateurs.

2.7 Archivage

Les investigateurs sont tenus de conserver toutes les données se rapportant à l'étude, y compris la feuille de recueil du consentement éclairé pour chaque patient, pendant au minimum 15 ans après la fin de celle-ci.

2.7 Audit

Les investigateurs s'engagent à se conformer aux demandes de TRB CHEMEDICA et des Autorités de Santé en ce qui concerne l'audit de l'étude (voir aussi vérification des documents sources).

3. RESPONSABILITES DU PROMOTEUR ET DES SOCIETES DE SERVICE

3.1 Responsabilités générales

TRB CHEMEDICA fournit une brochure d'information scientifique (Annexe 1.14) à jour ou toute information pertinente pour chacun des produits en essai clinique, enregistré ou non.
Pour les produits commercialisés, la notice du produit et/ou la monographie du produit à jour est fournie.
TRB CHEMEDICA, le conseiller scientifique, le statisticien, la société de monitoring élaborent une version finale du protocole.
SPRIT fournit aux investigateurs un nombre suffisant de cahiers d'observation.
TRB CHEMEDICA se réserve le droit d'arrêter prématurément l'essai pour cause de violation répétée et persistante du protocole ou pour toute autre raison valable et éthiquement justifiée.
Dans cette éventualité, les différents intervenants après examen et consultation mettront en œuvre les procédures nécessaires pour garantir la protection des intérêts des sujets.

3.2 Assurance

Dans la mesure où l'essai a été mené conformément au protocole, la responsabilité civile des investigateurs est couverte par la police d'assurance en responsabilité civile contractée par TRB CHEMEDICA (Annexe 2.9).

3.3 Monitoring et responsabilités des sociétés de service

L'étude est réalisée en conformité avec les Bonnes Pratiques Cliniques.
L'étude sera suivie régulièrement par un Attaché de Recherche Clinique (ARC) afin que soient vérifiées la bonne observance au protocole et la transcription des données dans le cahier d'observation.
Les investigateurs s'engagent conformément aux Bonnes Pratiques Cliniques à tenir des archives médicales contenant des données fiables.
L'Attaché de Recherche Clinique doit avoir accès à ces données lors des visites de contrôle.
Des contacts téléphoniques avec les investigateurs auront lieu.

3.4 Assurance qualité et contrôle qualité

Tout document utilisé dans les études cliniques est sujet à un contrôle de qualité.
Des audits d'assurance qualité peuvent être réalisés par TRB CHEMEDICA ou toute Autorité de Santé pendant la réalisation de l'étude ou après son achèvement.

3.5 Archives

TRB CHEMEDICA, ainsi que les sociétés de services (SPRIT, CEMKA), doivent conserver les documents relatifs à cette étude (protocole, documentation, approbation du protocole, rapport d'audit et d'inspection, ainsi que toute donnée relative à la recherche biomédicale) durant une période de 15 années après la fin de l'investigation ou de son arrêt anticipé (arrêté du 11 août 2008).
Tous les documents doivent être archivés en lieu sûr et traités de manière strictement confidentielle.

4. SIGNATURES

ESSAI CLINIQUE D'EVALUATION DE L'EFFICACITE
D'OSTENIL® PLUS (ACIDE HYALURONIQUE) VERSUS SYNVISC-ONE™
DANS LE TRAITEMENT DE LA GONARTHROSE FEMORO-TIBIALE

Fait en six exemplaires originaux, le 16 MAI 2011

Le Coordinateur National
Pr. THOMAS BARDIN

Le Promoteur
TRB CHEMEDICA
Dr. CHARLES JOLLES

La Conseillère Scientifique
Dr. RENEE LILIANE DREISER

Le Statisticien
CEMKA
Dr. ANTOINE LAFUMA

Le Gestionnaire de l'essai
SPRIT
MARC PIOCHAUD

PARTIE III : ANNEXES

1. ANNEXES LIEES A L'ETUDE

1.1 Critères ACR de la gonarthrose

1.2 Stades radiologiques de Kellgren / Lawrence

1.3 Indice algo-fonctionnel de LEQUESNE pour la gonarthrose

1.4 WOMAC - Western Ontario McMaster's Arthritis Index –

1.5 Critères de l'OMERACT-OARSI

1.6 Liste des traitements AINS

1.7 Temps de wash out des AINS

1.8 Echelle visuelle analogique EVA

1.9 Certificat d'analyse

1.10 Curriculum vitae du coordinateur

1.11 Investigateurs participant à l'essai

1.12 Cahiers d'observations

1.13 Cahier patient

1.14 Notice d'information scientifique d'Ostenil® Plus et Synvisc-One™

1.15 Fiche de pharmacovigilance et rapport d'évènement indésirable grave (EIG) ou « Adverse Event (SAE) Report »

1.16 Accusé de réception

1.17 Approbation du protocole et engagement de confidentialité

2. ANNEXES LIEES AUX OBLIGATIONS LEGALES

2.1 Déclaration d'Helsinki

2.2 Loi Huriet

2.3 Circulaire d'application sur les DMOS

2.4 Bonnes pratiques cliniques

2.5 Avis du CPP

2.6 Avis de l'AFSSAPS

2.7 Notice d'information au patient

2.8 Consentement éclairé du patient

2.9 Certificat d'assurance

2.10 Références

ANNEXE 1.1
CRITERES ACR DE LA GONARTHROSE

CRITERES DE CLASSIFICATION DE L'ARTHROSE DU GENOU DE L'AMERICAN COLLEGE OF RHEUMATOLOGY

Critères de définition de la gonarthrose

Un patient est considéré comme souffrant d'une gonarthrose s'il souffre d'une

1. Douleur du genou

ET

s'il répond à au moins 1 des 3 critères suivants :

1. Age > 50 ans
2. Raideur matinale < 30 minutes
3. Crépitement

ET

qu'il ait des ostéophytes à la radiographie du genou

Ces critères ont une sensibilité de 91% et une spécificité de 86%.

(18) Référence : Altman and Al., Arthritis Rheum., 1986, 29, pp. 1039-1049

ANNEXE 1.2
STADES RADIOLOGIQUES
KELLGREN / LAWRENCE

STADES RADIOLOGIQUES DE KELLGREN/LAWRENCE

**Stade I**	Léger pincement de l'interligne articulaire
**Stade I_A**	Pincement inférieur à 25%<br>Ostéophytose minime
**Stade I_B**	Pincement entre 25 et 50%<br>Ostéophytose minime
**Stade II**	Pincement net de l'interligne articulaire d'environ 50%<br>Ostéophytose évidente<br>Légère ostéosclérose
**Stade III**	Pincement marqué de l'interligne articulaire supérieur à 50%<br>Ostéophytose importante<br>Tissu osseux péri-articulaire scléreux avec déformation débutante des extrémités osseuses
**Stade IV**	Pincement très évolué de l'interligne articulaire (pincement supérieur à 75%)<br>Ostéophytose importante<br>Tissu osseux péri-articulaire très scléreux et géodique<br>Déformation des extrémités osseuses

(19) Référence : Kellgren JH, Lawrence DM - Radiological assessment of osteoarthritis. *Ann Rheum Dis* 1957;16:494-502.

ANNEXE 1.3
INDICE ALGO-FONCTIONNEL DE LEQUESNE POUR LA GONARTHROSE

INDICE ALGO-FONCTIONNEL DE LEQUESNE POUR LA GONARTHROSE

**DOULEUR OU GENE NOCTURNE ?**		|__| pt
Absence	0 pt	
Seulement aux mouvements et dans certaines postures	1 pt	
Même sans bouger	2 pt	
**DEROUILLAGE MATINAL ?**		|__| pt
Moins de 1 minute	0 pt	
Pendant 1 à 15 minutes	1 pt	
Plus de 15 minutes	2 pt	
**RESTER DEBOUT OU PIETINER ½ HEURE AUGMENTE-T-IL LA DOULEUR ?**		|__| pt
Non	0 pt	
Oui	1 pt	
**DOULEUR A LA MARCHE ?**		|__| pt
Absence	0 pt	
Après une certaine distance	1 pt	
Ou dès le début et de façon croissante	2 pt	
**POUVEZ-VOUS VOUS RELEVER SANS L'AIDE DES BRAS ?**		|__| pt
Oui	0 pt	
Non	1 pt	
**PERIMETRE DE MARCHE MAXIMAL** (y compris en consentant à souffrir) **?**		|__| pt
Illimité	0 pt	
Limité mais supérieur à 1 km (environ 15 minutes)	1 pt	
Environ 1 km	2 pt	
500 à 900 mètres (environ 8 à 15 minutes)	3 pt	
300 à 500 mètres	4 pt	
100 à 300 mètres	5 pt	
Moins de 100 mètres	6 pt	
Une canne ou une béquille nécessaire	+1 pt	
2 cannes ou cannes-béquilles nécessaires	+2 pt	
**AUTRES DIFFICULTES***		
Pouvez-vous monter un étage ?	0 à 2 pt	|__|,|__| pt
Pouvez-vous descendre un étage ?	0 à 2 pt	|__|,|__| pt
Pouvez-vous vous accroupir ou rester à genoux ?	0 à 2 pt	|__|,|__| pt
Pouvez-vous marcher en terrain irrégulier ?	0 à 2 pt	|__|,|__| pt

*** Cotation : 0 = pas de difficulté ; 1 = possibilité avec difficulté (ou 0,5 ou 1,5 selon le degré) ; 2 = impossible**

TOTAL |__|__|,|__| pt

(20) Référence: Lequesne M, Méry C, Samson M, Gérard P - Indexes of severity for osteoarthritis of the hip and knee. *Scand J Rheumatol* 1987;65(Suppl):85-9.

ANNEXE 1.4
WOMAC

WOMAC : index de sévérité symptomatique de l'arthrose des membres inférieurs
Le WOMAC est l'index validé dans l'évaluation d'une arthrose des membres inférieurs. Il existe 2 systèmes de cotation des réponses aux questions : soit l'échelle de LICKERT avec 5 réponses possibles (nulle = 0 ; minime = 1 ; modérée = 2 ; sévère = 3 ; extrême = 4), soit une échelle visuelle analogique de 100 mm. Il est possible de calculer les scores dans chaque domaine ou pour l'ensemble du WOMAC

Les moyennes des sous scores du WOMAC seront calculées en additionnant les 5 items relatifs à la douleur, les 2 items relatifs à la raideur, et les 17 items relatifs à la fonction physique, divisé par le nombre de questions s'y rapportant de façon à obtenir une valeur comprise entre 0 et 100 pour chaque sous score.

SECTION A

WOMAC Domaine douleur : quelle est l'importance de la douleur :

1. Lorsque vous marchez sur une surface plane ?

Absence de douleur ________________________ Douleur maximale

2. Lorsque vous montez ou descendez les escaliers ?

Absence de douleur ________________________ Douleur maximale

3. La nuit, lorsque vous êtes au lit ?

Absence de douleur ________________________ Douleur maximale

4. Lorsque vous vous levez d'une chaise ou vous asseyez ?

Absence de douleur ________________________ Douleur maximale

5. Lorsque vous vous tenez debout ?

Absence de douleur ________________________ Douleur maximale

SECTION B

WOMAC Domaine raideur : quelle est l'importance de la raideur de votre articulation :

1. Lorsque vous vous levez le matin ?

Absence de raideur ________________________ Raideur extrême

2. Lorsque vous bougez après vous être assis, couché ou reposé durant la journée ?

Absence de raideur ________________________ Raideur extrême

SECTION C

WOMAC Domaine fonction : quelle est l'importance de la difficulté que vous éprouvez à :

1. Descendre les escaliers ?

Absence de difficulté	______________________	Difficulté extrême

2. Monter les escaliers ?

Absence de difficulté	______________________	Difficulté extrême

3. Vous relever de la position assise ?

Absence de difficulté	______________________	Difficulté extrême

4. Vous tenir debout ?

Absence de difficulté	______________________	Difficulté extrême

5. Vous pencher en avant ?

Absence de difficulté	______________________	Difficulté extrême

6. Marcher en terrain plat ?

Absence de difficulté	______________________	Difficulté extrême

7. Entrer et sortir d'une voiture ?

Absence de difficulté	______________________	Difficulté extrême

8. Faire vos courses ?

Absence de difficulté	______________________	Difficulté extrême

9. Enfiler collants ou chaussettes ?

Absence de difficulté	______________________	Difficulté extrême

10. Sortir du lit ?

Absence de difficulté	______________________	Difficulté extrême

11. Enlever vos collants ou vos chaussettes ?

Absence de difficulté ______________________________ Difficulté extrême

12. Vous étendre sur le lit ?

Absence de difficulté ______________________________ Difficulté extrême

13. Entrer ou sortir d'une baignoire ?

Absence de difficulté ______________________________ Difficulté extrême

14. Vous asseoir ?

Absence de difficulté ______________________________ Difficulté extrême

15. Vous asseoir et vous relever des toilettes ?

Absence de difficulté ______________________________ Difficulté extrême

16. Faire le ménage "à fond" de votre domicile ?

Absence de difficulté ______________________________ Difficulté extrême

17. Faire l'entretien quotidien de votre domicile ?

Absence de difficulté ______________________________ Difficulté extrême

(17) Référence : Bellamy N, Buchan WW, Goldsmith CH, Campbell J, Stit LWJ. Validation of WOMAC: a health status instrument for measuring clinically important patient relevant outcomes to antirheumatic drug therapy in patients with osteoarthritis of the hip or knee. J Rheumatol 1995; 15: 1833-40

ANNEXE 1.5
CRITERES DE L'OMERACT-OARSI

Patients répondeurs selon les critères de l'OMERACT-OARSI

Les patients répondeurs sont définis par :

A) Une amélioration au moins égale à 50% (≥ 50%) de leur douleur ou de leur fonction et un changement absolu au moins égal à 20 mm (≥ 20 mm) sur une échelle EVA de 100 mm

B) Ou une amélioration d'au moins 2 des 3 critères suivants :

1. Douleur au moins améliorée de 20% (≥ 20%) et variation absolue au moins égale à 10 mm (≥ 10 mm)
2. Fonction au moins améliorée de 20% (≥ 20%) et variation absolue au moins égale à 10 mm (≥ 10 mm)
3. Opinion globale du patient au moins améliorée de 20% (≥ 20%) et variation absolue au moins égale à 10 mm (≥ 10 mm)

(23) Référence : Pham T, van der Heijde D, Altman RD, Anderson JJ, Bellamy N, Hochberg M, Simon L, Strand V, Woodworth T, Dougados M. OMERACT-OARSI Initiative: Osteoarthritis research society international set of responder criteria for osteoarthritis clinical trials revisited. Osteoarthritis cartilage 2004; 12: 389-399

ANNEXE 1.6
LISTE DES TRAITEMENTS
AINS

ANNEXE 1.6
AINS DEMI-VIE COURTE

**ACECLOFENAC**	Cartrex	100 mg		
**ALMINOPROFENE**	Minalfène	300 mg		
**DICLOFENAC**	Artotec	50 mg	75 mg	
	Voltarène	25 mg	50 mg	
	Xénid	50 mg		
**ETODOLAC**	Lodine	200 mg	300 mg	
**FLURBIPROFENE**	Antadys	100 mg		
	Cebutid	50 mg	100 mg	200 mg
**IBUPROFENE**	Advil	200 mg	400 mg	
	Antarène	100 mg	200 mg	400 mg
	Brufen	400 mg		
	Nurofen	200 mg		
**INDOMETACINE**	Indocid	25 mg		
**KETOPROFENE**	Kétum	100 mg		
	Profénid	50 mg	100 mg	
	Topfena	50 mg		
**NIFLUMIQUE ACIDE**	Nifluril	250 mg		
**NIMESULIDE**	Nexen	100 mg		
**SULINDAC**	Arthrocine	100 mg	200 mg	
**ACIDE TIAPROFENIQUE**	Flanid	100 mg	200 mg	
	Surgam	100 mg	200 mg	

ANNEXE 1.6

AINS DEMI-VIE LONGUE

**CELECOXIB**	Celebrex	100 mg	200 mg	
**DICLOFENAC**	Voltarène	75 mg LP	100 mg LP	
**ETORICOXIB**	Arcoxia	30 mg	60 mg	
**IBUPROFENE**	Nureflex	300 mg LP		
**INDOMETACINE**	Chrono-Indocid	75 mg		
**KETOPROFENE**	Bi-profénid	100 mg LP		
	Kétum	200 mg LP		
	Profénid	200 mg LP		
	Topfena	200 mg LP		
**MELOXICAM**	Mobic	7,5 mg	15 mg	
**NABUMETONE**	Nabucox	500 mg		
**NAPROXENE**	Apranax	275 mg	550 mg	750 mg
	Naprosyne	250 mg	500 mg	
	Naprosyne	750 mg	1000 mg	
**PIROXICAM**	Brexin	20 mg		
	Cycladol	20 mg		
	Feldène	10 mg	20 mg	
	Proxalyoc	20 mg		
**TENOXICAM**	Tilcotil	20 mg		

ANNEXE 1.7
TEMPS DE WASH OUT DES AINS

AINS DEMI VIE COURTE	Nom commercial	1/2 vie	Wash out en jours
**ACECLOFENAC**	Cartrex	4 h	2 jours
**ALMINOPROFENE**	Minalfène	4h - 6h	2 jours
**DICLOFENAC**	Artotec	3h - 6h	2 jours
	Voltarène	2h	2 jours
	Xénid	2h	2 jours
**ETODOLAC**	Lodine	7h	2 jours
**FLURBIPROFENE**	Antadys	$3h^{1/2}$- 4h	2 jours
	Cebutid	$3h^{1/2}$- 4h	2 jours
**IBUPROFENE**	Advil	2h	2 jours
	Antarène	2h	2 jours
	Brufen	2h	2 jours
	Nurofen	2h	2 jours
**INDOMETACINE**	Indocid	2h	2 jours
**KETOPROFENE**	Kétum	2h - 3h	2 jours
	Profénid	2h	2 jours
	Topfena	$1h^{1/2}$ - 2 h	2 jours
**NIFLUMIQUE ACIDE**	Nifluril	4h - 6h	2 jours
**NIMESULIDE**	Nexen	3h - 6h	2 jours
**SULINDAC**	Arthrocine	8h	2 jours
**ACIDE TIAPROFENIQUE**	Flanid	$1h^{1/2}$ - $2h^{1/2}$	2 jours
	Surgam	$1h^{1/2}$ - $2h^{1/2}$	2 jours

AINS DEMI VIE LONGUE	Nom commercial	1/2 vie	Temps de sevrage
**CELECOXIB**	Celebrex	8h - 12h	3 jours
**DICLOFENAC**	Voltarène LP	2h	2 jours
**ETORICOXIB**	Arcoxia	22h	5 jours
**IBUPROFENE**	Nureflex LP	7h	2 jours
**INDOMETACINE**	Chrono-Indocid	2,6h - 11,2h	3 jours
**KETOPROFENE**	Kétum LP	6h	2 jours
	Profénid LP	2h	2 jours
	Topfena LP	6h	2 jours
**MELOXICAM**	Mobic	20h	4 jours
**NABUMETONE**	Nabucox	20h - 24h	5 jours
**NAPROXENE**	Apranax	13h - 15h	3 jours
	Naprosyne	13h - 15h	3 jours
**PIROXICAM**	Brexin	50h	10 jours
	Cycladol	50h	10 jours
	Feldène	50h	10 jours
	Proxalyoc	50h	10 jours
**TENOXICAM**	Tilcotil	70h	15 jours

ANNEXE 1.8
ECHELLE VISUELLE ANALOGIQUE
EVA

ECHELLE VISUELLE ANALOGIQUE - EVA - DE LA DOULEUR SPONTANEE

SEVERITE DE LA DOULEUR

Absence de douleur	________________________________	Douleur insupportable

EVA |__|__|__| **mm**

(21) Référence : Huskisson EC. Measurement of pain. Lancet 1974 ; II: 1127-31

ANNEXE 1.9
CERTIFICAT D'ANALYSE
(SANS OBJET)

ANNEXE 1.10
CURRICULUM VITAE
DU COORDINATEUR

CURRICULUM VITAE

NOM : BARDIN
Prénoms : Thomas Pierre Jean

PERSONAL INFORMATION

TITRES HOSPITALIERS

PERSONAL INFORMATION

TITRES UNIVERSITAIRES

PERSONAL INFORMATION

SOCIETES SAVANTES

PERSONAL INFORMATION

COMITES DE LECTURE

PERSONAL INFORMATION

RESPONSABILITES A L'AGENCE DE SECURITE DES PRODUITS DE SANTE

PERSONAL INFORMATION

PRINCIPALES ACTIVITES DE RECHERCHE

PERSONAL INFORMATION

ANNEXE 1.11
INVESTIGATEURS
PARTICIPANT A L'ESSAI

INVESTIGATEUR PRINCIPAL MEDECIN RHUMATHOLOGUE

Titre	Nom	Prénom	Adresse	Code	Ville	Téléphone	N° Cnom
PERSONAL INFORMATION							

INVESTIGATEURS MEDECINS GENERALISTES

Titre	Nom	Prénom	Adresse	Code	Ville	Téléphone	N° Cnom

PERSONAL INFORMATION

INVESTIGATEURS MEDECINS GENERALISTES (SUITE)

Titre	Nom	Prénom	Adresse	Code	Ville	Téléphone	N° Cnom

PERSONAL INFORMATION

INVESTIGATEURS MEDECINS GENERALISTES (SUITE ET FIN)

Titre	Nom	Prénom	Adresse	Code	Ville	Téléphone	N° Cnom

PERSONAL INFORMATION

INVESTIGATEURS MEDECINS RHUMATOLOGUES

Titre	Nom	Prénom	Adresse	Code	Ville	Téléphone	N° Cnom

PERSONAL INFORMATION

INVESTIGATEURS MEDECINS RHUMATOLOGUES (SUITE)

Titre	Nom	Prénom	Adresse	Code	Ville	Téléphone	N° Cnom

PERSONAL INFORMATION

INVESTIGATEURS MEDECINS RHUMATOLOGUES (SUITE)

Titre	Nom	Prénom	Adresse	Code	Ville	Téléphone	N° Cnom

PERSONAL INFORMATION

INVESTIGATEURS MEDECINS RHUMATOLOGUES (SUITE)

Titre	Nom	Prénom	Adresse	Code	Ville	Téléphone	N° Cnom

PERSONAL INFORMATION

INVESTIGATEURS MEDECINS RHUMATOLOGUES (SUITE)

Titre	Nom	Prénom	Adresse	Code	Ville	Téléphone	N° Cnom

PERSONAL INFORMATION

INVESTIGATEURS MEDECINS RHUMATOLOGUES (SUITE ET FIN)

Titre	Nom	Prénom	Adresse	Code	Ville	Téléphone	N° Cnom

PERSONAL INFORMATION

INVESTIGATEUR MEDECIN DE MEDECINE PHYSIQUE ET DE READAPTATION

Titre	Nom	Prénom	Adresse	Code	Ville	Téléphone	N° Cnom
PERSONAL INFORMATION							

ANNEXE 1.12
CAHIERS D'OBSERVATION
(VOIR DOCUMENTS SEPARES)

ANNEXE 1.13
CAHIER PATIENT
(VOIR DOCUMENT SEPARE)

ANNEXE 1.14
NOTICE D'INFORMATION
OSTENIL®PLUS & SYNVISC-ONE™

OSTENIL®PLUS dispositif médical

FORMES et PRÉSENTATIONS :
Solution viscoélastique pour injection dans la cavité articulaire : Seringues préremplies de 40 mg/2 ml, sous emballage stérile, boite unitaire. Stérilisé par vapeur d'eau.

COMPOSITION :
Solution isotonique* : par ml
Hyaluronate de sodium** 20 mg
Excipients : chlorure de sodium, phosphate monosodique, phosphate disodique, mannitol, eau ppi.
*pH = 7,3
**d'origine fermentative

PROPRIÉTÉS :
Le liquide synovial, dont la viscoélasticité est liée à la présence d'acide hyaluronique, est présent dans toutes les articulations synoviales, et particulièrement dans les articulations supportant un poids important, où il permet des mouvements normaux et sans douleur grâce à ses propriétés lubrifiantes et d'absorption des chocs. Il assure également l'apport nutritionnel du cartilage.
Dans le cas d'affections dégénératives articulaires telles que l'ostéoarthrose, la viscoélasticité du liquide synovial est notablement diminuée, ce qui entraîne une réduction des fonctions lubrifiantes et d'absorption des chocs. Ceci augmente la charge mécanique de l'articulation et la destruction du cartilage. Ces phénomènes ont pour conséquence une mobilité réduite de l'articulation atteinte et l'apparition de douleurs. Une supplémentation du liquide synovial par des injections intra-articulaires d'acide hyaluronique hautement purifié permet d'améliorer les propriétés viscoélastiques du liquide synovial.
On observe une amélioration des propriétés lubrifiantes et d'absorption des chocs ainsi qu'une diminution de la surcharge mécanique de l'articulation. En règle générale, le résultat se traduit par une diminution de la douleur et une amélioration de la mobilité articulaire qui peut durer pendant plusieurs mois après le traitement.
OSTENIL®PLUS est une solution transparente d'acide hyaluronique naturel et hautement purifié, obtenu par fermentation bactérienne et dépourvu de protéines animales.
OSTENIL®PLUS contient aussi du mannitol, un piégeur de radicaux libres, qui aide à stabiliser les chaînes d'acide hyaluronique. Des études de biocompatibilité ont démontré que OSTENIL®PLUS était bien toléré.

UTILISATION :
Douleur et diminution de la mobilité dans les altérations dégénératives et traumatiques de l'articulation du genou et autres articulations synoviales.

MODE D'EMPLOI :
Injecter 1 seringue d'OSTENIL®PLUS dans l'articulation atteinte.
Il est possible de renouveler les injections jusqu'à 3 injections consécutives au total avec un intervalle entre les injections d'au minimun une semaine.
Plusieurs articulations peuvent être traitées simultanément.
Il est possible de répéter les cycles de traitement si nécessaire.
En cas d'épanchement articulaire, il est conseillé de réduire l'épanchement par aspiration, repos, application d'une poche de glace et/ou injection intra-articulaire d'un corticostéroïde. Le traitement avec OSTENIL®PLUS pourra alors reprendre deux à trois jours après.
Le contenu et la surface externe de la seringue préremplie d'OSTENIL®PLUS demeurent stériles tant que l'emballage stérile reste intact. Sortir la seringue préremplie de l'emballage stérile. Avant l'utilisation, la fermeture de sécurité de la seringue préremplie doit être rompue. Rompre la pièce transversale de la fermeture de sécurité. L'embout peut être retiré avec le capuchon. Attacher une aiguille appropriée (par exemple 18 à 25 G) et s'assurer de sa bonne fixation en la tournant légèrement. Avant l'injection, veiller à retirer de la seringue toute bulle d'air éventuellement présente.

CONTRE-INDICATIONS :
Antécédents d'hypersensibilité à l'un des constituants.

PRÉCAUTIONS D'EMPLOI :
Une attention particulière doit être portée aux patients présentant une hypersensibilité connue aux médicaments.
Les précautions générales liées aux injections intra-articulaires doivent être prises, y compris les mesures destinées à éviter une infection articulaire.
L'injection d'OSTENIL®PLUS doit être réalisée spécifiquement dans la cavité articulaire, si nécessaire sous contrôle scopique. Eviter l'injection dans les vaisseaux sanguins et dans les tissus avoisinants
En l'absence de données cliniques sur l'emploi de l'acide hyaluronique chez l'enfant, la femme enceinte ou allaitante, ou dans les affections inflammatoires articulaires telles que la polyarthrite rhumatoïde ou la maladie de Bechterew, l'administration d'OSTENIL® PLUS n'est pas recommandée chez ces patients.
Ne pas utiliser si la seringue préremplie ou l'emballage stérile sont endommagés.
Interactions :
A ce jour, aucune donnée n'est disponible sur l'incompatibilité d'OSTENIL®PLUS avec d'autres solutions à usage intra-articulaire. La prise simultanée d'analgésiques ou d'une médication anti-inflammatoire par voie orale pendant les premiers jours du traitement peut être utile au patient.
Effets indésirables :
Des manifestations locales telles que douleur, chaleur, rougeur et gonflement peuvent apparaître au niveau de l'articulation traitée par OSTENIL® PLUS. L'application d'une poche de glace pendant 5 à 10 minutes au niveau de la zone traitée réduira l'incidence de ces effets.

CONDITIONS DE CONSERVATION :
A conserver à température ambiante n'excédant pas 25°C.
Ne pas utiliser après la date d'expiration mentionnée sur la boîte.
Tenir hors de portée des enfants.

Prix conseillé 76.00 € (1 seringue)
ACL 3401096650204
Marquage CE. Classe III.

TRB CHEMEDICA
Immeuble ABC 1 Site d'Archamps
BP 40218.
74162 Archamps cedex
Tél : 04 50 95 09 00 Fax : 04 50 95 09 01

(26) Référence : Dictionnaire Vidal OCP éd, Paris 2011

SYNVISC-ONE™ HYLANE G-F 20

FORMES ET PRESENTATIONS :
SYNVISC-ONE, 6 ml : seringue en verre de 10 ml (ACL 486 402.2).
Hylane G-F 20 est un fluide élastoviqueux, stérile et non pyrogène qui contient des hylanes. Les hylanes sont des dérivés de l'hyaluronane (hyluronate de sodium) et elles sont composées d'unités disaccharidiques récurrentes de N-acétylglucosamine et de glucaronate de sodium.
L'hylane A a un poids moléculaire moyen de 6 000 000 daltons et l'hylane B est un gel hydraté.
Hylane G-F 20 contient de l'hylane A et l'hylane B (8.0 mg ± 2.0 mg/ml) dans une solution physiologique de chlorure de sodium tamponnée (pH 7.2 ± 0.3).

COMPOSITION : par seringue
Pour 1 ml (Hylane G-F20) : hylane 8.0 mg, chlorure de sodium 8.5 mg, phosphate disodique 0.16 mg, phosphate monosodique hydraté 0.04 mg, eau pour préparations injectables qs.
SYNVISC-ONE contient 6 ml de Hylane G-F 20.

PROPRIETES
Hylane G-F 20 est biologiquement semblable à l'hyaluronane. L'hyaluronane est un constituant du liquide synovial qui lui confère sa viscoélasticité. Les propriétés mécaniques (viscoélastiques) de Hylane G-F 20 sont toutefois supérieures à celles du liquide synovial et des solutions d'hyaluronane de concentration semblable. Hylane G-F 20 a une élasticité (coefficient de stockage G') à 2.5 Hz de 111 Pascals (Pa) ± 13 et une viscosité (coefficient de perte G'') de 25 Pa ± 2. L'élasticité et la viscosité du liquide synovial du genou de sujets âgés de 18 à 27 ans mesurées avec une méthode comparable à 2.5 Hz est G' = 117 Pa ± 13 ; G''= 45 Pa ± 8. Les hylanes sont métabolisées dans l'organisme par la même voie que l'hyaluronane et les produits de leur dégradation ne sont pas toxiques.

INDICATIONS :
Hylane G-F 20 :
est un agent de remplacement et un supplément temporaire du liquide synovial ;
se révèle efficace lorsqu'il est administré dans tous les stades de l'affection articulaire ;
se révèle le plus efficace lorsqu'il est administré à des patients dont l'activité physique régulière mobilise l'articulation atteinte ;
exerce ses effets thérapeutiques en agissant comme viscosupplémentation, en rétablissant les propriétés physiologiques et rhéologiques des tissus de l'articulation touchée par l'arthrose.
La viscosupplémentation avec Hylane G-F 20 est un traitement qui permet de réduire la douleur et la gêne et d'accroître ainsi l'amplitude du mouvement de l'articulation. Les études in vitro ont démontré que Hylane G-F 20 protège les cellules du cartilage contre certaines lésions physiques et chimiques.

MODE D'EMPLOI ET POSOLOGIE :
SYNVISC-ONE ne doit être administré que par voie intra-articulaire, et par un médecin, pour soulager la douleur associée à l'arthrose du genou.
Aspirer le liquide synovial ou tout épanchement avant chaque injection de Hylane G-F 20.
Ne pas utiliser Hylane G-F 20 si l'emballage est ouvert ou endommagé.
Injecter à la température ambiante.
Pour retirer la seringue de la coque thermoformée (ou du plateau), la saisir par le corps sans toucher la tige du piston.
Injecter en appliquant les méthodes d'asepsie strictes, et en accordant une attention particulière au retrait du capuchon à l'extrémité de la seringue.
Dévisser le capuchon gris à l'extrémité de la seringue avant de le retirer afin de minimiser les fuites du produit.

Utiliser une aiguille de diamètre appropriée : SYNVISC-ONE seringue de 18 à 20 gauges.
Utiliser une aiguille de longueur adéquate, selon l'articulation à traiter.
Pour assurer un raccordement étanche et prévenir toute fuite lors de l'administration, adapter solidement l'aiguille au collier de serrage de type Luer de la seringue.
Ne pas serrer ou exercer une pression excessive pour fixer l'aiguille ou retirer sa gaine sous peine de casser l'extrémité de la seringue.
Ne pas stériliser de nouveau Hylane G-F 20.
Injecter seulement dans l'espace synovial, en effectuant l'opération, si besoin est, sous guidage fluoroscopique, notamment dans le cas du traitement des articulations de la hanche et de l'épaule.
Le contenu de la seringue est à usage unique exclusivement.
Lorsque l'on travaille sous contrôle radioscopique, un produit de contraste ionique peut être utilisé. Ne pas utiliser plus de 1 ml de produit de contraste par 2 ml de Hylane G-F 20.

Posologie :
La posologie de Hylane G-F 20 dépend de l'articulation à traiter.

Arthrose du genou :
Il est recommandé d'administrer SYNVISC-ONE selon un schéma posologique d'une injection dans le genou. Une seconde injection peut être pratiquée 6 mois après la première, si les symptômes du patient le justifient.

Durée des effets :
Le traitement avec Hylane G-F 20 n'agit que sur l'articulation traitée : il n'entraîne aucun effet systématique.
SYNVISC-ONE : les données cliniques prospectives montrent un effet du traitement jusqu'à 26 semaines après une seule injection de SYNVISC-ONE.

CONTRE-INDICATIONS :
En présence d'une stase veineuse ou lymphatique dans le membre correspondant, on ne doit pas injecter Hylane G-F 20 dans l'articulation.
On ne doit pas injecter Hylane G-F 20 dans une articulation infectée ou gravement enflammée ou chez des patients ayant une affection cutanée ou une infection au niveau du site de l'injection.

PRÉCAUTIONS D'EMPLOI :
Mises en garde :
Ne pas injecter par voie intravasculaire.
Ne pas injecter en dehors de la cavité articulaire ou dans le tissu synovial ou la capsule.
Ne pas utiliser en concomitance avec des désinfectants contenant des sels d'ammonium quaternaire pour la préparation de la peau car le hyaluronate peut précipiter en leur présence.

Précautions :
Ne pas administrer Hylane G-F 20 en présence d'un épanchement important dans l'articulation avant l'injection.
Comme après toute intervention invasive dans l'articulation, il est recommandé au patient d'éviter toute activité physique intense après l'injection intra-articulaire et de reprendre des activités normales après quelques jours.
Hylane G-F 20 n'a pas été évalué chez les femmes enceintes ni chez les enfants de moins de 18 ans.
Hylane G-F 20 contient une faible quantité de protéines aviaires et il ne doit pas être administré à des personnes présentant une hypersensibilité à ces protéines.

Effets indésirables :
Les injections intra-articulaires de Hylane G-F 20 peuvent entraîner de la douleur, un œdème et/ou un épanchement passagers. Synvisc a démontré que dans certains cas, l'épanchement peut être plus important et entraîner une douleur plus marquée ; il est alors essentiel de drainer et d'analyser ce liquide pour éliminer une cause infectieuse ou microcristalline. Ces réactions se résolvent généralement en quelques jours. Un bénéfice thérapeutique est toujours possible après des réactions de ce type. Aucun cas d'infection intra-articulaire n'est survenu lors des essais cliniques avec SYNVISC/SYNVISC-ONE; de rares cas ont été rapportés après la commercialisation de SYNVISC.
Les réactions systémiques rares, rapportées après l'administration de SYNVISC, sont les suivantes : érythème, urticaire, démangeaison, fièvre, nausée, céphalée, étourdissement, frissons, crampes musculaires, paresthésie, œdème périphérique, malaise, difficultés respiratoires, bouffées de chaleur et œdème facial. Les essais cliniques contrôlés avec SYNVISC n'ont permis de relever aucune différence statistique significative dans le nombre ou les types d'effets systémiques indésirables entre le groupe de patients traité par SYNVISC, et le groupe témoin.
Dans l'essai contrôlé avec SYNVISC-ONE, la fréquence et les types d'effets indésirables étaient similaires dans le groupe ayant reçu SYNVISC-ONE et le groupe ayant reçu un placebo.

CONDITIONS DE CONSERVATION :
Conserver entre +2 °C et +30 °C.
Ne pas congeler.
Ne pas utiliser si l'emballage est ouvert ou endommagé.

Tarif LPPR
76.00 € code : LPPR 1130495 (Synvisc one boîte de 1 seringue)
Remb Séc soc exclusivement pour les patients atteints de gonarthrose, après échec des antalgiques et échec ou intolérance aux anti-inflammatoires non stéroïdiens (AINS) dans la limite:
D'un traitement composé d'une seule injection par an et par genou (Synvisc one, JO du 22.01.09)
Prise en charge subordonnée à la prescription et à la réalisation de l'injection intra-articulaire soit par un rhumatologue, soit par un chirurgien orthopédique, soit par un médecin de médecine physique et de réadaptation.
Marquage CE 0088.

GENZYME SAS
33-35, boulevard de la Paix. 78105 Saint-Germain-en-Laye cedex
Info et commandes : Tél n° Indigo : 0825825861 Matériovigilance : n° Indigo : 0825801051
Info médic : n° Indigo : 08 25 80 14 03

(27) Référence : Dictionnaire Vidal OCP éd, Paris 2010

ANNEXE 1.15
FICHE DE PHARMACOVIGILANCE ET RAPPORT D'EVENEMENT INDESIRABLE GRAVE (EIG)
« SERIOUS ADVERSE EVENT (SAE) REPORT

**Date**<br>Jour Mois Année	**Description**	**Gravité**<br>0 = Non grave<br>1 = Grave (a)	**Intensité**<br>0 = Légère<br>1 = Modérée<br>2 = Sévère	**Début**<br>0 = Avant l'injection<br>1 = Pendant ou juste après l'injection<br>2 = Après l'injection (b)	**Durée**<br>Jours Heures Minutes	**Imputabilité**<br>0 = Exclue<br>1 = Improbable<br>3 = Probable<br>4 = Certaine<br>5 = Inconnue/ non évaluable	**Intervention médicale**<br>0 = Aucune<br>1 = Traitement (c)<br>2 = Possible	**Evolution**<br>0 = Guérison<br>1 = En cours<br>2 = Hospitalisation	**Commentaires**<br>1 = Poursuit l'essai<br>2 = Arrête l'essai
Date de début<br>|__|__| |__|__| |__|__| Date de fin<br>|__|__| |__|__| |__|__| ou ❑ en cours					|__|__| |__|__| |__|__| (sauf si en cours)				
Date de début<br>|__|__| |__|__| |__|__| Date de fin<br>|__|__| |__|__| |__|__| ou ❑ en cours					|__|__| |__|__| |__|__| (sauf si en cours)				
Date de début<br>|__|__| |__|__| |__|__| Date de fin<br>|__|__| |__|__| |__|__| ou ❑ en cours					|__|__| |__|__| |__|__| (sauf si en cours)				

(a) Si grave complétez le formulaire EIG (Cf. protocole partie II paragraphe 2.5 pour la définition des termes des évènements indésirables)
(b) Détailler le nombre de jours et d'heures si possible
(c) Description du traitement : ____

Rapport d'évènement indésirable grave (EIG)

Serious Adverse Event (SAE) Report

Page 1/3

Faxez ce rapport <u>immédiatement</u> ou au plus tard dans les 24 heures. Téléphonez au no. indiqué ci-après pour confirmer sa réception :
TRB Chemedica International SA, Rue Michel-Servet 12, Case postale 352, 1211 Genève 12, Suisse. Fax: +41 22 703 49 01.
Tél. : +41 22 703 49 21 pendant les heures de bureau et fax : +41 22 703 49 01 en dehors des heures de bureau.
A : Dr. My-Lam Nguyen

Cette case est destinée au personnel de TRB Chemedica International SA	Cas n° : ………………………………………………..	Avis de réception ☐☐ ☐☐ ☐☐☐☐ jour mois année

Données du patient

Patient n° :	Initiales du patient :	Date de naissance :	Grossesse :	Sexe :
	……………………….. nom/prénom	------/-------/------------- jour mois année	☐ oui ☐ non ☐ ne s'applique pas	☐ masculin ☐ féminin

Données de l'étude

Protocole n° :	Centre n° :	Pays où a eu lieu l'EIG :
O S T P - E U R - 1 0 - 0 1 ☐	....	……………………...

Rapport de l'EIG

Rapporteur	Nom :……………………………………………………………….……………………................ Adresse :…………………………………………………………….…………………….......... N° fax :……………………………………..……...………………………………….............. N° tél :………….……………………………………………………...………..……….............

Critères de l'EIG

Cochez toutes les cases pertinentes	☐ décès ☐ hospitalisation du patient ou prolongation d'une hospitalisation existante ☐ handicap persistant ou significatif ☐ représentant un danger pour la vie ☐ anomalie congénitale/défaut de naissance ☐ autre condition médicalement importante Précisez : ………………………………………………………………………………

Détails de l'EIG

	Complétez par ordre d'importance svp		
Diagnostic(s) (si aucun diagnostic n'est disponible, indiquez les symptômes principaux)	1 ..…………………………..... …………………………….. ……………………………..	2 ..…………………………..... …………………………….. ……………………………..	3 ..…………………………..... …………………………….. ……………………………..
Date et heure d'apparition	☐☐ ☐☐ ☐☐☐☐ jour mois année ☐☐ : ☐☐ heures min	☐☐ ☐☐ ☐☐☐☐ jour mois année ☐☐ : ☐☐ heures min	☐☐ ☐☐ ☐☐☐☐ jour mois année ☐☐ : ☐☐ heures min
Date et heure de fin (cochez la case "en cours" le cas échéant)	☐☐ ☐☐ ☐☐☐☐ jour mois année ☐☐ : ☐☐ heures min ☐ en cours	☐☐ ☐☐ ☐☐☐☐ jour mois année ☐☐ : ☐☐ heures min ☐ en cours	☐☐ ☐☐ ☐☐☐☐ jour mois année ☐☐ : ☐☐ heures min ☐ en cours
Intensité maximum	☐ légère ☐ modérée ☐ sévère	☐ légère ☐ modérée ☐ sévère	☐ légère ☐ modérée ☐ sévère

Serious Adverse Event (SAE) Report

Page 2/3

Protocole n° :	Site n° :	Patient n° :	Initiales du patient<br>…………………………...<br>Initiales: nom/prénom
O S T P - E U R - 1 0 - 0 1			

Imputabilité			
Imputabilité	☐ exclue<br>☐ improbable<br>☐ possible<br>☐ probable<br>☐ certaine<br>☐ inconnue/non évaluable	☐ exclue<br>☐ improbable<br>☐ possible<br>☐ probable<br>☐ certaine<br>☐ inconnue/non évaluable	☐ exclue<br>☐ improbable<br>☐ possible<br>☐ probable<br>☐ certaine<br>☐ inconnue/non évaluable

Description de l'EIG, y compris le déroulement de l'événement, et commentaires

Spécifiez les informations pertinentes concernant le diagnostic, l'évaluation de la causalité, le dosage du médicament étudié, les résultats de laboratoires pertinents, *etc*.

………......................

………......................

Administration du médicament étudié

Médicament étudié	Indication : …………………………………………….
« Code cassé » (ouverture de l'enveloppe scellée)	☐ oui ➔ [ ][ ] [ ][ ] [ ][ ][ ][ ]<br>☐ non jour mois année<br>☐ ne s'applique pas
Médicament étudié	Si l'étude est ouverte ou si le code a été cassé, précisez :…………………………………………………<br>Route :……………………………………Dosage journalier : ……………………………....
Première administration du médicament étudié	[ ][ ] [ ][ ] [ ][ ][ ][ ] jour mois année [ ][ ] : [ ][ ] heures min
Dernière administration du médicament étudié avant l'EIG	[ ][ ] [ ][ ] [ ][ ][ ][ ] jour mois année [ ][ ] : [ ][ ] heures min
Action entreprise liée à l'EIG du médicament étudié	☐ pas d'action<br>☐ interruption du traitement puis reprise<br>☐ réduction de la dose<br>☐ augmentation de la dose<br>☐ arrêt du traitement<br><br>Commentaires (durées, changements de dosage *etc*) :<br>…………………………………………………………………..............<br>…………………………………………………………………..............<br>…………………………………………………………………..............<br>…………………………………………………………………..............
Cet événement a-t-il eu lieu précédemment ?	☐ oui ☐ non
L'EIG a-t-il disparu après l'arrêt ?	☐ oui ☐ non ☐ ne s'applique pas
L'EIG est-il réapparu après réintroduction du produit ?	☐ oui ☐ non ☐ ne s'applique pas

Traitements concomitants

Nom générique (nom de marque pour les médicaments combinés)	Indication	Dosage total journalier	Route	Première administration	Dernière administration
………………………………	…………	…………		[ ][ ] [ ][ ] [ ][ ][ ][ ]<br>jour mois année	[ ][ ] [ ][ ] [ ][ ][ ][ ]<br>jour mois année<br>☐ en cours
………………………………	…………	…………		[ ][ ] [ ][ ] [ ][ ][ ][ ]<br>jour mois année	[ ][ ] [ ][ ] [ ][ ][ ][ ]<br>jour mois année<br>☐ en cours

**Serious Adverse Event (SAE) Report**			**Page 3/3**
Protocole n° :	Site n° :	Patient n° :	Initiales du patient<br>…………………..<br>Initiales: nom/prénom
O S T P - E U R - 1 0 - 0 1			

**Traitement spécifique de l'EIG (médicaments, procédures)**	
Traitement spécifique pour cet EIG	☐ aucun<br>☐ traitement médicamenteux ⌦ précisez ci-dessous<br>☐ traitement non médicamenteux ⌦ précisez ci-dessous<br>……………………………………………………………………………………………..<br>……………………………………………………………………………………………..
**Evolution**	
Cochez la case appropriée	☐ récupération/guérison<br>☐ en cours de récupération/guérison<br>☐ pas de guérison/en cours ➤**de plus amples informations doivent être fournies dès que possible**<br>☐ guérison avec séquelles ⌦ précisez<br>…………………………………………...<br>……………………………………………………………………………………………..<br>☐ fatale/⌦ date du décès __ __ | __ __ | __ __ __ __<br>jour mois année<br>☐ inconnue
En cas de décès	Cause du décès<br>☐ progression de la maladie étudiée<br>☐ EIG<br>☐ autre<br><br>Détails sur la cause du décès<br>……………………………………………………………………...<br>……………………………………………………………………………………………...<br>oui non<br>autopsie faite ☐ ☐<br>rapport d'autopsie disponible ☐ ☐
**Antécédents du patient**	
(Antécédents, maladies concomitantes, allergies, facteurs de risque importants, résultats de laboratoire, opérations chirurgicales, biopsies, circonstances non usuelles, diagnostic différentiel, *etc.*)<br>……………………………………………………………………………………...<br>……..<br>……..	
Type de rapport: initial ☐ suivi ☐ n° …………	
………………………………..<br>Date	…………………………………………………………………..<br>Signature de l'investigateur (attestant de l'exactitude des informations)

ANNEXE 1.16
ACCUSE DE RECEPTION

ACCUSE DE RECEPTION MEDECIN EVALUATEUR

ESSAI CLINIQUE D'EVALUATION DE L'EFFICACITE D'OSTENIL® PLUS (ACIDE HYALURONIQUE) VERSUS SYNVISC- ONE™ DANS LE TRAITEMENT DE LA GONARTHROSE FEMORO-TIBIALE

Je soussigné(e) Docteur ____ certifie avoir reçu ce jour :

☑ 1 Brochure d'information scientifique

☑ 1 Protocole

☑ 4 Cahiers d'observation

☑ 4 Cahiers patient

☑ 4 Formulaires de consentement éclairé

☑ 4 Notices d'information au patient

☑ 2 Conventions financières investigateur

☑ 1 Feuille de signature d'approbation du protocole et d'engagement de confidentialité

☑ 4 Enveloppes de randomisation |__|__|__| |__|__|__| |__|__|__| |__|__|__|

<u>Ces éléments sont</u> :

☑ En bon état

❑ Endommagés

Observations éventuelles : ____

____

Date |__|__| |__|__| |__|__|__|__| Signature de l'investigateur

ACCUSE DE RECEPTION MEDECIN INJECTEUR

ESSAI CLINIQUE D'EVALUATION DE L'EFFICACITE D'OSTENIL® PLUS (ACIDE HYALURONIQUE) VERSUS SYNVISC- ONE™ DANS LE TRAITEMENT DE LA GONARTHROSE FEMORO-TIBIALE

Je soussigné(e) Docteur ____ certifie avoir reçu ce jour :

☑ 1 Brochure d'information scientifique

☑ 1 Protocole

☑ 4 Cahiers d'observation

☑ 2 Conventions financières investigateur

☑ 1 Feuille de signature d'approbation du protocole et d'engagement de confidentialité

☑ 4 Lots thérapeutiques |__|__|__| |__|__|__| |__|__|__| |__|__|__|

☑ 4 Enveloppes de randomisation |__|__|__| |__|__|__| |__|__|__| |__|__|__|

Ces éléments sont :

☑ En bon état

❑ Endommagés

Observations éventuelles : ____

____

Date |__|__| |__|__| |__|__|__|__| Signature de l'investigateur

ANNEXE 1.17
APPROBATION DU PROTOCOLE ET ENGAGEMENT DE CONFIDENTIALITE

APPROBATION DU PROTOCOLE
ENGAGEMENT DE CONFIDENTIALITE

ESSAI CLINIQUE D'EVALUATION DE L'EFFICACITE D'OSTENIL® PLUS (ACIDE HYALURONIQUE) VERSUS SYNVISC- ONE™ DANS LE TRAITEMENT DE LA GONARTHROSE FEMORO-TIBIALE

Je soussigné Docteur____

déclare avoir pris connaissance du protocole scientifique et des directives qu'il comporte pour la conduite de l'étude dénommée ci-dessus.

Je m'engage à respecter le protocole et la confidentialité de l'étude.

Date |__|__| |__|__| |__|__|__|__| Signature de l'investigateur

ANNEXE 2.1
DECLARATION D'HELSINKI

DECLARATION D'HELSINKI DE L'ASSOCIATION MEDICALE MONDIALE

Principes éthiques applicables aux recherches médicales sur des sujets humains

Adoptée par la 18ème Assemblée Médicale Mondiale
Helsinki (Finlande), Juin 1964
et amendée par :

29ème Assemblée Médicale Mondiale,
Tokyo (Japon), Octobre 1975

35ème Assemblée Médicale Mondiale,
Venise (Italie), Septembre 1983

41ème Assemblée Médicale Mondiale,
Hong Kong, Septembre 1989

48ème Assemblée générale
Somerset West (République d'Afrique du Sud), Octobre 1996

52ème Assemblée générale, Edimbourg, Octobre 2000

Assemblée générale de l'AMM, Washington 2002
(addition d'une note explicative concernant le paragraphe 29)

Assemblée générale de l'AMM, Tokyo 2004
(addition d'une note explicative concernant le paragraphe 30)

59ème Assemblée générale, Séoul, (Corée), Octobre 2008

Introduction]

La **Déclaration d'Helsinki**, élaborée par l'Association médicale mondiale, constitue une déclaration de principes éthiques dont l'objectif est de fournir des recommandations aux médecins et autres participants à la recherche médicale sur des êtres humains. Celle-ci comprend également les études réalisées sur des données à caractère personnel ou des échantillons biologiques non anonymes.

La mission du médecin est de promouvoir et de préserver la santé de l'être humain. Il exerce ce devoir dans la plénitude de son savoir et de sa conscience.

Le Serment de Genève de l'Association médicale mondiale lie le médecin dans les termes suivants : "*La santé de mon patient sera mon premier souci*" et le Code international d'éthique médicale énonce que "*le médecin devra agir uniquement dans l'intérêt de son patient lorsqu'il lui procure des soins qui peuvent avoir pour conséquence un affaiblissement de sa condition physique ou mentale*".

Les progrès de la médecine sont fondés sur des recherches qui, in fine, peuvent imposer de recourir à l'expérimentation humaine.

Dans la recherche médicale sur les sujets humains, les intérêts de la science et de la société ne doivent jamais prévaloir sur le bien-être du sujet.

L'objectif essentiel de la recherche médicale sur des sujets humains doit être l'amélioration des méthodes diagnostiques, thérapeutiques et de prévention, ainsi que la compréhension des causes et des mécanismes des maladies. Les méthodes diagnostiques, thérapeutiques et de prévention, même les plus éprouvées, doivent constamment être remises en question par des recherches portant sur leur efficacité, leur efficience et leur accessibilité.

Dans la recherche médicale comme dans la pratique médicale courante, la mise en œuvre de la plupart des méthodes diagnostiques, thérapeutiques et de prévention expose à des risques et à des contraintes.

La recherche médicale est soumise à des normes éthiques qui visent à garantir le respect de tous les êtres humains et la protection de leur santé et de leurs droits. Certaines catégories de sujets sont plus vulnérables que d'autres et appellent une protection adaptée. Les besoins spécifiques des sujets défavorisés au plan économique comme au plan médical doivent être identifiés. Une attention particulière doit être portée aux personnes qui ne sont pas en mesure de donner ou de refuser elles-mêmes leur consentement, à celles qui sont susceptibles de donner leur consentement sous la contrainte, à celles qui ne bénéficieront pas personnellement de la recherche et à celles pour lesquelles la recherche est conduite au cours d'un traitement.

L'investigateur doit être attentif aux dispositions éthiques, légales et réglementaires applicables à la recherche sur les sujets humains dans son propre pays ainsi qu'aux règles internationales applicables. Aucune disposition nationale d'ordre éthique, légal et réglementaire ne doit conduire à affaiblir ou supprimer les mesures protectrices énoncées dans la présente déclaration.

Principes fondamentaux applicables à toute forme de recherche médicale

Dans la recherche médicale, le devoir du médecin est de protéger la vie, la santé, la dignité et l'intimité de la personne.

La recherche médicale sur des êtres humains doit se conformer aux principes scientifiques généralement reconnus. Elle doit se fonder sur une connaissance approfondie de la littérature scientifique et des autres sources pertinentes d'information ainsi que sur une expérimentation appropriée réalisée en laboratoire et, le cas échéant, sur l'animal.

Des précautions particulières doivent entourer les recherches pouvant porter atteinte à l'environnement et le bien-être des animaux utilisés au cours des recherches doit être préservé.

La conception et l'exécution de chaque phase de l'expérimentation sur des sujets humains doivent être clairement définies dans un protocole expérimental. Ce protocole doit être soumis pour examen, commentaires, avis et, le cas échéant, pour approbation, à un comité d'éthique mis en place à cet effet. Ce comité doit être indépendant du promoteur, de l'investigateur ou de toute autre forme d'influence indue. Il doit respecter les lois et règlements en vigueur dans le pays où s'effectuent les recherches. Il a le droit de suivre le déroulement des études en cours. L'investigateur a l'obligation de fournir au comité des informations sur le déroulement de l'étude portant en particulier sur la survenue d'événements indésirables d'une certaine gravité. L'investigateur doit également communiquer au comité, pour examen, les informations relatives au financement, aux promoteurs, à toute appartenance à une ou des institutions, aux éventuels conflits d'intérêt ainsi qu'aux moyens d'inciter des personnes à participer à une recherche.

Le protocole de la recherche doit contenir une déclaration sur les implications éthiques de cette recherche. Il doit préciser que les principes énoncés dans la présente déclaration sont respectés.

Les études sur l'être humain doivent être conduites par des personnes scientifiquement qualifiées et sous le contrôle d'un médecin compétent. La responsabilité à l'égard d'un sujet inclus dans une recherche doit toujours incomber à une personne médicalement qualifiée et non au sujet, même consentant.

Toute étude doit être précédée d'une évaluation soigneuse du rapport entre d'une part, les risques et les contraintes et d'autre part, les avantages prévisibles pour le sujet ou d'autres personnes. Cela n'empêche pas la participation à des recherches médicales de volontaires sains. Le plan de toutes les études doit être accessible.

Un médecin ne doit entreprendre une étude que s'il estime que les risques sont correctement évalués et qu'ils peuvent être contrôlés de manière satisfaisante. Il doit être mis un terme à la recherche si les risques se révèlent l'emporter sur les bénéfices escomptés ou si des preuves consistantes de résultats positifs et bénéfiques sont apportées.

Une étude ne peut être réalisée que si l'importance de l'objectif recherché prévaut sur les contraintes et les risques encourus par le sujet. C'est particulièrement le cas lorsqu'il s'agit d'un volontaire sain.

Une recherche médicale sur des êtres humains n'est légitime que si les populations au sein desquelles elle est menée ont des chances réelles de bénéficier des résultats obtenus.

Les sujets se prêtant à des recherches médicales doivent être des volontaires informés des modalités de leur participation au projet de recherche.

Le droit du sujet à la protection de son intégrité doit toujours être respecté. Toutes précautions doivent être prises pour respecter la vie privée du sujet, la confidentialité des données le concernant et limiter les répercussions de l'étude sur son équilibre physique et psychologique.

Lors de toute étude, la personne se prêtant à la recherche doit être informée de manière appropriée des objectifs, méthodes, financement, conflits d'intérêts éventuels, appartenance de l'investigateur à une ou des institutions, bénéfices attendus ainsi que des risques potentiels de l'étude et des contraintes qui pourraient en résulter pour elle. Le sujet doit être informé qu'il a la faculté de ne pas participer à l'étude et qu'il est libre de revenir à tout moment sur son consentement sans crainte de préjudice. Après s'être assuré de la bonne compréhension par le sujet de l'information donnée, le médecin doit obtenir son consentement libre et éclairé, de préférence par écrit. Lorsque le consentement ne peut être obtenu sous forme écrite, la procédure de recueil doit être formellement explicitée et reposer sur l'intervention de témoins.

Lorsqu'il sollicite le consentement éclairé d'une personne à un projet de recherche, l'investigateur doit être particulièrement prudent si le sujet se trouve vis-à-vis de lui dans une situation de dépendance ou est exposé à donner son consentement sous une forme de contrainte. Il est alors souhaitable que le consentement soit sollicité par un médecin bien informé de l'étude mais n'y prenant pas part et non concerné par la relation sujet-investigateur.

Lorsque le sujet pressenti est juridiquement incapable, physiquement ou mentalement hors d'état de donner son consentement ou lorsqu'il s'agit d'un sujet mineur, l'investigateur doit obtenir le consentement éclairé du représentant légal en conformité avec le droit en vigueur. Ces personnes ne peuvent être incluses dans une étude que si celle-ci est indispensable à l'amélioration de la santé de la population à laquelle elles appartiennent et ne peut être réalisée sur des personnes aptes à donner un consentement.

Lorsque le sujet, bien que juridiquement incapable (un mineur par exemple), est cependant en mesure d'exprimer son accord à la participation à l'étude, l'investigateur doit obtenir que cet accord accompagne celui du représentant légal.

La recherche sur des personnes dont il est impossible d'obtenir le consentement éclairé, même sous forme de procuration ou d'expression préalable d'un accord, ne doit être conduite que si l'état physique ou mental qui fait obstacle à l'obtention de ce consentement est une des caractéristiques requises des sujets à inclure dans l'étude. Les raisons spécifiques d'inclure des sujets dans une étude en dépit de leur incapacité à donner un consentement éclairé doivent être exposées dans le protocole qui sera soumis au comité pour examen et approbation. Le protocole doit également préciser que le consentement du sujet ou de son représentant légal à maintenir sa participation à l'étude doit être obtenu le plus rapidement possible.

Les auteurs et les éditeurs de publications scientifiques ont des obligations d'ordre éthique. Lors de la publication des résultats d'une étude, les investigateurs doivent veiller à l'exactitude des résultats. Les résultats négatifs aussi bien que les résultats positifs doivent être publiés ou rendus accessibles. Le financement, l'appartenance à une ou des institutions et les éventuels conflits d'intérêt doivent être exposés dans les publications. Le compte-rendu d'une étude non conforme aux principes énoncés dans cette déclaration ne doit pas être accepté pour publication.

Principes applicables à la recherche médicale conduite au cours d'un traitement

Le médecin ne peut mener une recherche médicale au cours d'un traitement que dans la mesure où cette recherche est justifiée par un possible intérêt diagnostique, thérapeutique ou de prévention. Quand la recherche est associée à des soins médicaux, les patients se prêtant à la recherche doivent bénéficier de règles supplémentaires de protection.

Les avantages, les risques, les contraintes et l'efficacité d'une nouvelle méthode doivent être évalués par comparaison avec les meilleures méthodes diagnostiques, thérapeutiques ou de prévention en usage. Cela n'exclut ni le recours au placebo ni l'absence d'intervention dans les études pour lesquelles il n'existe pas de méthode diagnostique, thérapeutique ou de prévention éprouvée.

Afin de clarifier la position de l'AMM sur l'utilisation des essais avec témoins sous placebo, le Conseil de l'AMM a rédigé en octobre 2001 une note explicative, que vous trouverez sur cette page.

Tous les patients ayant participé à une étude doivent être assurés de bénéficier à son terme des moyens diagnostiques, thérapeutiques et de prévention dont l'étude aura montré la supériorité. (Note explicative)

Le médecin doit donner au patient une information complète sur les aspects des soins qui sont liés à des dispositions particulières du protocole de recherche. Le refus d'un patient de participer à une étude ne devra en aucun cas porter atteinte aux relations que le médecin entretient avec ce patient.

Lorsqu'au cours d'un traitement, les méthodes établies de prévention, de diagnostic ou de thérapeutique s'avèrent inexistantes ou insuffisamment efficaces, le médecin, avec le consentement éclairé du patient, doit pouvoir recourir à des méthodes non éprouvées ou nouvelles s'il juge que celles-ci offrent un espoir de sauver la vie, de rétablir la santé ou de soulager les souffrances du malade. Ces mesures doivent, dans toute la mesure du possible, faire l'objet d'une recherche destinée à évaluer leur sécurité et leur efficacité. Toute nouvelle information sera consignée et, le cas échéant, publiée. Les autres recommandations appropriées énoncées dans la présente déclaration s'appliquent.

Note explicative concernant le paragraphe 29

L'AMM note avec préoccupation que le paragraphe 29 de la Déclaration d'Helsinki (Octobre 2000) est l'objet d'interprétations diverses et de possibles malentendus. Elle réaffirme par ailleurs que les essais avec témoins sous placebo ne doivent être utilisés qu'avec de grandes précautions et, d'une façon générale, lorsqu'il n'existe pas de traitement éprouvé. Toutefois, même s'il existe un traitement éprouvé, les essais avec témoins sous placebo peuvent être éthiquement acceptables dans les conditions suivantes :

lorsque, pour des raisons méthodologiques impérieuses et scientifiquement solides, il n'existe pas d'autres moyens qui permettent de déterminer l'efficacité ou l'innocuité d'une méthode prophylactique, diagnostique ou thérapeutique ; ou lorsqu'une méthode prophylactique, diagnostique ou thérapeutique est mise à l'essai pour une affection bénigne et que la participation à l'essai n'expose pas à des risques supplémentaires de dommages significatifs ou durables.

Toutes les dispositions énoncées dans la DoH doivent être respectées, en particulier, la nécessité d'un examen éthique et scientifique approfondi.

Note explicative concernant le paragraphe 30

L'AMM réaffirme par la présente qu'il est important, pendant la phase de planification d'une étude, d'identifier l'accès après étude des patients impliqués aux traitements prophylactiques, diagnostiques et thérapeutiques, jugés comme bénéfiques au cours de l'étude ou bien à des soins adéquats. Le mode d'accès post étude ou les autres soins doivent être décrits dans le protocole de l'étude afin que le comité d'éthique puisse étudier ces dispositions.

ANNEXE 2.2
LOI HURIET

Ministère de la Solidarité, de la Sante
et de la Protection Sociale

LOI HURIET

Dispositions législatives

(Paru au Journal Officiel Loi N° 88-1138 du 20 décembre 1988, modifiée par la Loi N° 90-86 du 23 janvier 1990, modifiée par la Loi N° 94-630 du 25 juillet 1994)

PROTECTION DES PERSONNES QUI SE PRETENT

A DES RECHERCHES BIOMEDICALES

Article L.209-1
Les essais ou expérimentations organisés et pratiqués sur l'être humain en vue du développement des connaissances biologiques ou médicales sont autorisés dans les conditions prévues au présent livre et sont désignés ci-après par les termes : « recherche biomédicale ».
Les recherches biomédicales dont on attend un bénéfice direct pour la personne qui s'y prête sont dénommées recherches biomédicales avec bénéfice individuel direct. Toutes les autres recherches, qu'elles portent sur des personnes malades ou non, sont dénommées sans bénéfice individuel direct.
La personne physique ou morale qui prend l'initiative d'une recherche biomédicale sur l'être humain est dénommée ci-après le promoteur. La ou les personnes physiques qui dirigent et surveillent la réalisation de la recherche sont dénommées ci-après les investigateurs.
Lorsque plusieurs personnes prennent l'initiative d'une même recherche, elles peuvent désigner une personne physique ou morale qui aura la qualité de promoteur et assumera les obligations correspondantes en application du présent livre.
Lorsque le promoteur d'une recherche confie sa réalisation à plusieurs investigateurs, il désigne parmi eux un investigateur coordonnateur.

TITRE I DISPOSITIONS GENERALES

Article L.209-2
Aucune recherche biomédicale ne peut être effectuée sur l'être humain.
- si elle ne se fonde pas sur le dernier état des connaissances scientifiques et sur une expérimentation pré clinique suffisante
- si le risque prévisible encouru par les personnes qui se prêtent à la recherche est hors de proportion avec le bénéfice escompté pour ces personnes ou l'intérêt de cette recherche
- si elle ne vise pas à étendre la connaissance scientifique de l'être humain et les moyens susceptibles d'améliorer sa condition.

Article L.209-3
Les recherches biomédicales ne peuvent être effectuées que :
- sous la direction et sous la surveillance d'un médecin justifiant d'une expérience appropriée
- dans des conditions matérielles et techniques adaptées à l'essai et compatibles avec les impératifs de rigueur scientifique et de sécurité des personnes qui se prêtent à ces recherches.

Les recherches biomédicales concernant le domaine de l'odontologie ne peuvent être effectuées que sous la direction et la surveillance d'un chirurgien-dentiste et d'un médecin justifiant d'une expérience appropriée.
Dans les sciences du comportement humain, une personne qualifiée, conjointement avec l'investigateur, peut exercer la direction de la recherche.

Article L.209-4
Les recherches sans bénéfice individuel direct sur les femmes enceintes, les parturientes et les mères qui allaitent ne sont admises que si elles ne présentent aucun risque prévisible pour leur santé ou celle de leur enfant, si elles sont utiles à la connaissance des phénomènes de la grossesse ou à l'allaitement et si elles ne peuvent être réalisées autrement.

Article L.209-5
Les personnes privées de liberté par une décision judiciaire ou administrative, les malades en situation d'urgence et les personnes hospitalisées sans consentement en vertu des articles L.333 et L.342 qui ne sont pas protégées par la loi ne peuvent être sollicitées pour se prêter à des recherches biomédicales que s'il en est attendu un bénéfice direct et majeur pour leur santé.

Article L.209-6
Les mineurs, les majeurs protégés par la loi et les personnes admises dans un établissement sanitaire ou social à d'autres fins que celle de la recherche ne peuvent être sollicités pour une recherche biomédicale que si l'on peut en attendre un bénéfice direct pour leur santé.
Toutefois, les recherches sans bénéfice individuel direct sont admises si les trois conditions suivantes sont remplies :
- ne présenter aucun risque sérieux prévisible pour leur santé
- être utiles à des personnes présentant les mêmes caractéristiques d'âge, de maladie ou de handicap
- ne pouvoir être réalisées autrement.

Article L.209-7
Pour les recherches biomédicales sans bénéfice individuel direct, le promoteur assume, même sans faute, l'indemnisation des conséquences dommageables de la recherche pour la personne qui s'y prête et celle de ses ayants droits, sans que puisse être opposé le fait d'un tiers ou le retrait volontaire de la personne qui avait initialement consenti à se prêter à la recherche.
Pour les recherches biomédicales avec le bénéfice individuel direct, le promoteur assume l'indemnisation des conséquences dommageables de la recherche pour la personne qui s'y prête et celle de ses ayants droits, sauf preuve à sa charge que le dommage n'est pas imputable à sa faute, ou à celle de tout intervenant sans que puisse être opposé le fait d'un tiers ou le retrait volontaire de la personne qui avait initialement consenti à se prêter à la recherche.
La recherche biomédicale exige la souscription préalable, par son promoteur, d'une assurance garantissant sa responsabilité civile telle qu'elle résulte du présent article et celle de tout intervenant, indépendamment de la nature des liens existant entre les intervenants et le promoteur. Les dispositions du présent article sont d'ordre public.

Article L.209-8
La recherche biomédicale ne donne lieu à aucune contrepartie financière directe ou indirecte pour les personnes qui s'y prêtent, hormis le remboursement des frais exposés et sous réserve de dispositions particulières prévues par l'article L.209-15 du présent code relatif aux recherches sans bénéfice individuel direct.

TITRE II DU CONSENTEMENT

Article L.209-9

Préalablement à la réalisation d'une recherche biomédicale sur une personne, le consentement libre, éclairé et exprès de celle-ci doit être recueilli après que l'investigateur, ou un médecin qui le représente, lui a fait connaître :
- L'objectif de la recherche, sa méthodologie et sa durée
- Les bénéfices attendus, les contraintes et les risques prévisibles, y compris en cas d'arrêt de la recherche avant son terme
- L'avis du comité mentionné à l'article L.209-12 du présent code
- Le cas échéant, son inscription dans le fichier national prévu à l'article L.209-17.

Il informe la personne dont le consentement est sollicité de son droit de refuser de participer à une recherche ou de retirer son consentement à tout moment sans encourir aucune responsabilité.
L'objectif d'une recherche en psychologie, ainsi que sa méthodologie et sa durée, peuvent ne faire l'objet que d'une information préalable succincte dès lors que la recherche ne porte que sur des volontaires sains et ne présente aucun risque sérieux prévisible. Une information complète sur cette recherche est fournie à l'issue de celle-ci aux personnes s'y étant prêtées.
Le projet visé au premier alinéa de l'article L.209-12 mentionne la nature des informations préalables transmises aux personnes se prêtant à la recherche.
A titre exceptionnel, lorsque dans l'intérêt d'une personne malade le diagnostic de sa maladie n'a pu lui être révélé, l'investigateur peut, dans le respect de sa confiance, réserver certaines informations liées à ce diagnostic. Dans ce cas, le protocole de la recherche doit mentionner cette éventualité.
Les informations communiquées sont résumées dans un document écrit remis à la personne dont le consentement est sollicité.
Le consentement est donné par écrit ou, en cas d'impossibilité, attesté par un tiers. Ce dernier doit être totalement indépendant de l'investigateur et du promoteur.
Toutefois, en cas de recherches biomédicales à mettre en œuvre dans des situations d'urgence qui ne permettent pas de recueillir le consentement préalable de la personne qui y sera soumise, le protocole présenté à l'avis du comité instauré par l'article L.209-11 du présent code peut prévoir que le consentement de cette personne ne sera pas recherché et que seul sera sollicité celui des membres de sa famille s'ils sont présents, dans les conditions prévues ci-dessus. L'intéressé sera informé dès que possible et son consentement lui sera demandé pour la poursuite éventuelle de cette recherche.

Article L.209-10

Lorsqu'une recherche biomédicale est effectuée sur des mineurs ou des majeurs protégés par la loi :
- Le consentement doit être donné, selon les règles prévues à l'article L.209-9 du présent code, par les titulaires de l'exercice de l'autorité parentale pour les mineurs non émancipés. Pour les mineurs ou les majeurs protégés par la loi, le consentement est donné par le représentant légal pour les recherches avec bénéfice individuel direct ne présentant pas un risque prévisible sérieux et, dans les autres cas, par le représentant légal autorisé par le conseil de famille ou le juge des tutelles.
- Le consentement du mineur ou du majeur protégé par la loi doit également être recherché lorsqu'il est apte à exprimer sa volonté. Il ne peut être passé outre à son refus ou à la révocation de son consentement.

TITRE III DISPOSITIONS ADMINISTRATIVES

Article L.209-11

Dans chaque région, le ministre chargé de la santé agrée un ou, selon les besoins, plusieurs comités consultatifs de protection des personnes dans la recherche biomédicale.
Le ministre fixe par arrêté le nombre de comités dans chaque région. Le champ de compétence territorial d'un comité peut être étendu à plusieurs régions.
Les comités exercent leur mission en toute indépendance. Ils sont dotés de la personnalité juridique.

Les comités sont compétents au sein de la région où ils ont leur siège. Un décret en Conseil d'Etat fixe les conditions minimales d'activité en deçà desquelles le champ de compétence territorial d'un comité peut être étendu à plusieurs régions.
Les comités sont composés de manière à garantir leur indépendance et la diversité des compétences dans le domaine biomédical et à l'égard des questions éthiques, sociales, psychologiques et juridiques.
Leurs membres sont nommés par le représentant de l'Etat dans la région où le comité a son siège. Ils sont choisis parmi les personnes figurant sur une liste établie sur proposition d'organismes ou d'autorités habilitées à la faire dans les conditions déterminées par décret.
Les membres des comités, les personnes appelées à collaborer à leurs travaux, les agents de l'Etat et les agents relevant de la loi 86-33 du 9 janvier 1986 portant dispositions statutaires relatives à la fonction publique hospitalière qui en sont dépositaires sont tenus, dans les conditions et sous les peines prévues à l'article 378 du code pénal, de garder secrètes les informations dont ils peuvent avoir connaissance à raison de leurs fonctions et qui sont relatives à la nature des recherches, aux personnes qui les organisent ou qui s'y prêtent ou aux produits, objets ou méthodes expérimentés.
Ne peuvent visiblement participer à une délibération les personnes qui ne sont pas indépendantes du promoteur et de l'investigateur de la recherche examinée.
Le frais de fonctionnement des comités sont financés par le produit d'un droit fixe versé par les promoteurs pour chacun des projets de recherches biomédicales faisant l'objet d'une demande d'avis. Le montant de ce droit est arrêté par le ministre chargé de la santé.
Le ministre chargé de la santé peut retirer l'agrément d'un comité si les conditions d'indépendance, de composition ou de fonctionnement nécessaires pour assurer sa mission dans les meilleures conditions ne sont pas satisfaites

Article L.209-12

Avant de réaliser une recherche biomédicale sur l'être humain, tout investigateur est tenu d'en soumettre le projet à l'un des comités consultatifs de protection des personnes dans la recherche biomédicale compétents pour la région où l'investigateur exerce son activité. Il ne peut solliciter qu'un seul avis par projet de recherche.
Dans le cas d'une recherche confiée à plusieurs investigateurs, cet avis est demandé par l'investigateur coordonnateur, qui soumet le projet dans les conditions définies au premier alinéa du premier article.
Le comité rend son avis sur les conditions de validité de la recherche au regard de la protection des personnes, notamment la protection des participants, leur information avant et pendant la durée de la recherche et les modalités de recueil de leur consentement, les indemnités éventuellement dues, la pertinence générale du projet et l'adéquation entre les objectifs poursuivis et les moyens mis en œuvre ainsi que la qualification du ou des investigateurs. Dans un délai de cinq semaines, il fait connaître par écrit son avis à l'investigateur. Il communique à l'autorité compétente tout avis défavorable donné à un projet de recherche.
Avant sa mise en œuvre, le promoteur transmet à l'autorité compétente une lettre d'intention décrivant les données essentielles de la recherche accompagnée de l'avis du comité consulté.
Cet avis ne le dégage pas de sa responsabilité. Les projets ayant fait l'objet d'un avis défavorable ne peuvent être mis en œuvre avant un délai de deux mois à compter de leur réception par l'autorité compétente.

Lorsque la recherche doit se dérouler dans un ou plusieurs établissements publics ou privés, le promoteur en informe le ou les directeurs de ces établissements avant que cette recherche ne soit mise en œuvre.

Le promoteur informe, dès qu'il en a connaissance, l'autorité compétente de tout effet ayant pu contribuer à la survenue d'un décès, provoquer une hospitalisation ou entraîner des séquelles organiques ou fonctionnelles durables et susceptibles d'être dû à la recherche. Le promoteur transmet également à l'autorité administrative compétente toute information relative à un fait nouveau concernant le déroulement de la recherche ou le développement du produit ou du dispositif faisant l'objet de la recherche lorsque ce fait nouveau est susceptible de porter atteinte à la sécurité des personnes qui se prêtent à la recherche. Il l'informe enfin de tout arrêt prématuré de la recherche en indiquant le motif de cet arrêt.
L'autorité administrative compétente peut, à tout moment, demander au promoteur des informations complémentaires sur la recherche. En cas d'absence de réponse du promoteur, de risque pour la santé publique ou de non-respect des dispositions du présent livre, elle peut également à tout moment suspendre ou interdire une recherche biomédicale.

Article L.209-12-1
Le comité consultatif de protection des personnes peut émettre dans les conditions prévues à l'article L.209-12 un avis favorable à la réalisation d'une recherche sous réserve de la transmission d'informations complémentaires par l'investigateur pendant le déroulement de celle-ci.
A la suite de cette transmission, le comité peut maintenir ou modifier son avis. Cette décision est transmise par écrit à l'investigateur dans un délai de cinq semaines ; elle est adressée par le promoteur à l'autorité administrative compétente dans un délai d'une semaine après sa réception.

Article L.209-13
Les médecins inspecteurs de la santé et dans les limites de leurs attributions, les inspecteurs de l'Agence du médicament, ont qualité pour veiller au respect des dispositions du présent livre et des textes réglementaires pris pour son application.

Article L.209-13-1
Les modalités de consultation des comités consultatifs de protection des personnes dans la recherche biomédicale en ce qui concerne les recherches à caractère militaire sont fixées par décret au Conseil d'Etat.

TITRE IV DISPOSITIONS PARTICULIERES AUX RECHERCHES SANS BENEFICE INDIVIDUEL DIRECT

Article L.209-14
Les recherches biomédicales sans bénéfice individuel direct ne doivent comporter aucun risque prévisible sérieux pour la santé des personnes qui s'y prêtent.
Elles doivent être précédées d'un examen médical des personnes concernées. Les résultats de cet examen leur sont communiqués préalablement à l'expression de leur consentement par l'intermédiaire du médecin de leur choix.

Article L.209-15
Dans le cas d'une recherche sans bénéfice individuel direct à l'égard des personnes qui s'y prêtent, le promoteur peut verser à ces personnes une indemnité en compensation des contraintes subies. Le montant total des indemnités qu'une personne peut percevoir au cours d'une même année est limité à un maximum fixé par le ministre chargé de la santé.
Les recherches effectuées sur des mineurs, des majeurs protégés par la loi ou des personnes admises dans un établissement sanitaire ou social à d'autres fins que celle de la recherche ne peuvent en aucun cas donner lieu au versement de l'indemnité prévue au premier alinéa du présent article.

Article L.209-16
Toute recherche biomédicale sans bénéfice individuel direct sur une personne qui n'est pas affiliée à un régime de sécurité sociale ou bénéficiaire d'un tel régime est interdite.
L'organisme de sécurité sociale dispose contre le promoteur d'une action en paiement des prestations versées ou fournies.

Article L.209-17
Nul ne peut se prêter simultanément à plusieurs recherches biomédicales sans bénéfice individuel direct.
Pour chaque recherche sans bénéfice individuel direct, le protocole soumis à l'avis consultatif du comité consultatif de protection des personnes dans la recherche biomédicale détermine une période d'exclusion au cours de laquelle la personne qui s'y prête ne peut participer à une autre recherche sans finalité thérapeutique directe. La durée de cette période varie en fonction de la nature de la recherche.
En vue de l'application des dispositions ci-dessus, le ministre chargé de la santé établit et gère un fichier national.

Article L.209-18
Les recherches biomédicales sans bénéfice individuel direct ne peuvent être réalisées que dans un lieu équipé des moyens matériels et techniques adaptés à la recherche et compatibles avec les impératifs de sécurité des personnes qui s'y prêtent, autorisé, à ce titre, par le ministre chargé de la santé.

Article L.209-18-1
Aucune recherche biomédicale ne peut être effectuée sur une personne en état de mort cérébrale sans son consentement exprimé directement ou par le témoignage de sa famille.
Les dispositions de l'article 225-17 du code pénal ne sont pas applicables à ces recherches.

TITRE V SANCTIONS PENALES

Article L.209-19
Ainsi qu'il est dit à l'article 223-8 du code pénal, le fait de pratiquer ou de faire pratiquer sur une personne une recherche biomédicale sans avoir recueilli le consentement libre, éclairé et exprès de l'intéressé, des titulaires de l'autorité parentale ou du tuteur dans les cas prévus par les dispositions du présent code sont punis de trois ans d'emprisonnement et de 300 000 F d'amende.
Les mêmes peines sont applicables lorsque le consentement a été retiré avant qu'il ne soit procédé à la recherche biomédicale.
Ainsi qu'il est dit à l'article 223-9 du code pénal, les personnes morales peuvent être déclarées responsable pénalement, dans les conditions prévues par l'article 121-2 du code pénal, de cette infraction.
Les peines encourues par les personnes morales sont :
L'amende, suivant les modalités prévues par l'article 131-38 du code pénal
Les peines mentionnées à l'article 131-39 du code pénal. L'interdiction mentionnée au 2° de l'article 131-39 du code pénal porte sur l'activité dans l'exercice de laquelle ou à l'occasion de laquelle l'infraction a été commise.

Article L.209-19-1
Le fait de pratiquer ou de faire pratiquer une recherche biomédicale en infraction aux dispositions des articles L.209-4 à L.209-6 et du dernier alinéa de l'article L.209-9 est puni de trois ans d'emprisonnement et de 300 000 F d'amende.
Les personnes physiques coupables de l'infraction prévue à l'alinéa précédent encourent également les peines suivantes :
L'interdiction des droits physiques, civils et de famille, suivant les modalités prévues par l'article 131-26 du code pénal.

L'interdiction pour une durée de cinq ans au plus d'exercer l'activité professionnelle ou sociale à l'occasion de laquelle ou dans l'exercice de laquelle l'infraction a été commise
La confiscation définie à l'article 131-21 du code pénal
L'exclusion des marchés publics à titre définitif ou pour une durée de cinq ans au plus.
Les personnes morales peuvent être déclarées responsables pénalement, dans les conditions prévues par l'article 121-2 du code pénal, de l'infraction définie à l'alinéa premier.
Les peines encourues par les personnes morales sont :
L'amende, suivant les modalités prévues par l'article 131-38 du code pénal ;
Les peines mentionnées à l'article 131-39 du code pénal.
L'interdiction mentionnée au 2° de l'article 131-39 du code pénal porte sur l'activité dans l'exercice de laquelle ou à l'occasion de laquelle l'infraction a été commise.

Article L.209-20
Est puni d'un an d'emprisonnement et de 100 000 F d'amende :
- quiconque aura pratiqué ou fait pratiquer une recherche biomédicale sans avoir obtenu l'avis préalable prévu par l'article L.209-12 du présent code ;
- quiconque aura pratiqué ou fait pratiquer une recherche biomédicale dans des conditions contraires aux dispositions des deux premiers alinéas de l'article L.209-17 du présent code ;
- quiconque aura pratiqué ou fait pratiquer, continué de pratiquer ou de faire pratiquer une recherche biomédicale dont la réalisation a été interdite ou suspendue par le ministre chargé de la santé.
L'investigateur qui réalise une telle recherche en infraction aux dispositions de l'article L.209-18 est puni des mêmes peines.

Article L.209-21
Le promoteur dont la responsabilité civile n'est pas garantie par l'assurance prévue à l'article L.209-7 du présent code est puni d'un an d'emprisonnement et de 100 000 F d'amende.
Le promoteur qui réalise ou fait réaliser une recherche biomédicale sans avoir transmis au ministre chargé de la santé la lettre d'intention prévue à l'article L.209-12 est puni des mêmes peines.

TITRE VI DISPOSITIONS DIVERSES

Article L.209-22
Par dérogation à l'article 13 de la loi des 16 et 24 août 1790 sur l'organisation judiciaire, le tribunal de grande instance est seul compétent pour statuer sur toute action en indemnisation des dommages résultant d'une recherche biomédicale ; cette action se prescrit dans les conditions prévues à l'article 2270-1 du code civil.

Article L.209-23
Les dispositions du présent livre sont applicables dans les collectivités territoriales de Saint-Pierre et Miquelon et de Mayotte.

ANNEXE 2.3
CIRCULAIRE D'APPLICATION SUR LES DMOS

JOURNAL OFFICIEL – 6 AOUT 1993

MINISTERE DES AFFAIRES SOCIALES
DE LA SANTE ET DE LA VILLE

SANTE

Circulaire du 9 juillet 1993 relative à l'application de l'article L 365-1 du code de la santé publique.

NOR : SANP3301911C

Paris, le 9 juillet 1993

Le Ministre d'Etat, ministre des affaires sociales de la santé et de la ville, le ministre de l'économie et le ministre délégué à la santé à Mesdames et Messieurs les préfets de départements (directions départementales des affaires sanitaires et sociales à l'attention de Mesdames et Messieurs les médecins inspecteurs départementaux de la santé, directions départementales de la concurrence, de la consommation et de la répression des fraudes).

L'article 47 de la loi du 27 janvier 1993 portant diverses mesures d'ordre social introduit notamment un article L 365-1 dans le code de la santé publique. Ce texte a pour objet d'assurer une plus grande transparence dans les relations entre les professionnels du secteur de la santé et les entreprises de ce secteur assurant des prestations, produisant ou commercialisant des produits pris en charge par les régimes obligatoires de sécurité sociale. Il n'a pas pour objet d'empêcher les activités de recherche et d'évaluation scientifique dont font partie notamment les actions de formation médicale continue, ces activités devant toutefois faire l'objet d'une procédure conventionnelle. Les interrogations que suscite ce texte nous amènent à apporter un certain nombre de précisions sur son sens et sa portée afin notamment d'orienter l'activité des médecins inspecteurs de la santé publique et des agents de la direction générale de la concurrence et de la répression des fraudes chargées de veiller au respect des dispositions légales.

I – Le principe de l'interdiction des avantages en nature ou espèces

a) Le sens de l'interdiction :
Les professionnels, dans le choix qu'ils font d'un médicament, d'un matériel ou d'une prestation remboursés par les régimes obligatoires d'assurance maladie, ne doivent être guidés que par des considérations d'ordre exclusivement médical. Ce principe éthique est exprimé dans les codes de déontologie (art. 9, 10 et 23 du code de déontologie médicale, art. 6 dernier alinéa, du code de déontologie des chirurgiens dentistes, art. 7 du code de déontologie des sages-femmes).
Avant même la promulgation de l'article L 365-1 des dispositions du même type existaient dans notre droit interne (art. L 549 et R 5046-1 du code de la santé publique).
A l'échelon européen, la directive n° 92-28 du 31 mars 1992 concernant la publicité faite à l'égard des médicaments à usage humain a également introduit une régulation de cette activité.

C'est à la lumière de ces principes qu'il convient d'interpréter l'article L 365-1 du code de la santé publique.

b) Le champ d'application de l'interdiction :

1 – Les professionnels concernés

Sont concernés les professions médicales visées au titre 1er du Livre IV du code de la santé publique (médecins, chirurgiens-dentistes, sages-femmes) ainsi que les professions visées au titre II au chapitre 1er du titre III et au titre III-1 du Livre IV dudit code (infirmiers, masseurs kinésithérapeutes, orthophonistes, orthoptistes).

2 – Les entreprises concernées

Le champ d'application de l'article L 365-1 est à cet égard très vaste puisque est concernée « toutes entreprises assurant les prestations produisant ou commercialisant des produits pris en charge par les régimes obligatoires de sécurité sociale ». Sont donc visées non seulement les entreprises fabriquant ou commercialisant des produits pharmaceutiques, mais aussi notamment celles fabriquant et commercialisant des matériels médico-chirurgicaux tels que les prothèses internes inertes, les stimulateurs cardiaques et les implants intraoculaires… Entrent aussi dans le champ d'application de l'article L 365-1 les sociétés de communication ou toute autre structure agissant pour le compte d'entreprises elles-mêmes concernées par son application.

3 – Les organismes non concernés par l'interdiction de recevoir des avantages

Ne sont pas concerné par l'interdiction de recevoir des avantages les organismes constitués de professionnels de la santé tels que, notamment, les associations, les organisations syndicales, les fonds d'assurance formation ou les sociétés savantes qui reçoivent des entreprises visées au point 2 ci-dessus des avantages en nature ou en espèces.

Il en est de même dans le secteur hospitalier public ou privé à but ou non lucratif des associations constituées en vue de recueillir des fonds ou des dons en nature destinés à permettre d'apporter une aide aux activités d'un ou plusieurs services hospitaliers.

Dans tous les cas, les avantages en nature ou en espèces reçus par les groupements sus énumérés doivent, pour échapper à l'interdiction prévue à l'article L 365-1 être exclusivement utilisés à des fins collectives.

4 – Champ d'application dans le temps de la disposition légale

Les dispositions de l'article 47 de la loi du 27 janvier 1993 s'appliquent à compter du 1er février 1993. L'interdiction posée par le premier alinéa de l'article L 365-1 vaut donc pour tous les avantages procurés à compter de cette date. Les avantages considérés comme licites en application du deuxième alinéa de l'article L 365-1 procurés après le 1er février 1993, doivent faire l'objet d'une convention soumise pour avis au conseil départemental de l'ordre compétent avant sa mise en application.

Toutefois, bien évidemment, les agents chargés de vérifier les conditions d'application de l'article L 365-1 tiendront compte des délais nécessaires à l'information des intéressés sur ce mécanisme législatif. Les vérifications porteront sur les pratiques mises en œuvre après la publication de la présente circulaire.

c) Les avantages dont la fourniture est interdite :

L'interdiction de recevoir des avantages est, sous réserve des exceptions indiquées au II ci-dessous générale.

Il est indifférent que ces avantages aient un rapport avec des produits ou des prestations non prises en charge dès lors que l'entreprise qui les versent commercialise des produits ou assure des prestations prise en charge par un régime obligatoire de sécurité sociale.

Outre la perception d'avantage en espèces (commissions, ristournes, remboursement de frais), est également prohibée celle d'avantages en nature (cadeaux, invitations, prise en charge de voyages d'agrément…).

Peu importe que ces voyages soient directs ou indirects, c'est-à-dire bénéficient au professionnel lui-même ou soient accordés à ses proches ou à des groupements dont il ferait partie. La constitution d'une association ne saurait être un moyen d'échapper aux dispositions de l'article L 365-1 : il est interdit de percevoir par l'intermédiaire d'une association des avantages qu'il serait interdit de percevoir directement.

Toutefois, compte tenu tant de l'esprit de la loi que des termes de la directive du 31 mars 1992, ne doivent pas être considérés comme entrant dans le champ de l'interdiction les avantages de valeur intrinsèque négligeable, puisque, par nature, ceux-ci ne constituent pas réellement des avantages.
Ainsi, peuvent être acceptés de menus présents dès lors qu'ils sont de valeur négligeable et qu'ils ont trait à l'exercice de la médecine ou de la pharmacie. De même, l'acceptation d'une invitation au restaurant ou à une manifestation telle que cocktail, buffet n'est pas par elle-même illégale. Toutefois, leur répétition, l'importance des frais exposés ou leur extension aux membres de la famille de l'invité ou à ses proches changeraient le caractère de telles invitations.
Par ailleurs, la délivrance d'échantillons pharmaceutiques n'est pas concernée par l'interdiction dès lors qu'elle correspond aux règles définies par l'article R 5046-2 du code de la santé publique.
Aux termes de ce texte, il ne peut être délivré d'échantillons qu'aux personnes habilitées à prescrire des médicaments à condition qu'elles en aient fait la demande et dans les limites fixées par les dispositions relatives à leur droit de prescription. Les échantillons doivent être identiques aux spécialités pharmaceutiques et porter la mention « échantillon médical gratuit ».
Enfin, la délivrance de ces échantillons est interdite dans les enceintes accessibles au public à l'occasion de congrès médicaux et pharmaceutiques.

II – Les exceptions à l'interdiction des avantages en nature ou en espèces

1 – Les activités de recherche ou d'évaluation scientifique ne rentrent pas dans le champ de l'interdiction à condition de respecter la procédure qui sera décrite au (2) ci-dessous
Si le législateur a entendu interdire l'octroi d'avantages matériels aux professionnels par les entreprises qui assurent des prestations, fabriquent ou commercialisent des médicaments ou produits faisant l'objet d'une prise en charge par les régimes obligatoires d'assurance maladie, il n'a nullement entendu priver les entreprises concernées de la possibilité de contribuer au financement d'activités de recherche ou d'évaluation scientifique entendue au sens large, c'est-à-dire comprenant aussi bien l'accomplissement des recherches ou des évaluations que la diffusion de leur résultat.
Doivent être considérés comme tels :
Les activités de recherche stricto sensu, y compris les recherches biomédicales régies par la loi n° 88-1138 du 20 décembre 1998.
Les colloques, séminaires, journées d'études ayant pour objet de faire le point des connaissances, des recherches ou des pratiques dans un domaine déterminé.
Les actions de formation médicale continue lorsqu'elles sont financées dans des conditions autres que celles prévues au 1.b.3. Encore faut-il que les actions énumérées ci-dessus visent effectivement les objectifs qu'elles affichent.
Dans ce cadre, conformément aux dispositions de l'article 10 de la directive précitée, l'hospitalité offerte aux participants demeure licite, dès lors qu'elle se situe à un niveau raisonnable, reste accessoire par rapport à l'objet scientifique et professionnel et n'est pas étendue à des personnes autres que les professionnels directement concernés.
Une précision doit être apportée s'agissant, d'une part, de la rémunération des recherches biomédicales et, d'autre part, de celle des brevets.
S'agissant de la rémunération des activités de recherche biomédicale, celle-ci, si elle ne peut être proportionnelle au nombre de produits prescrits, doit correspondre à l'importance du travail accompli dans le cadre du protocole d'étude et peut par exemple tenir compte du nombre d'observations demandées aux praticiens.
S'agissant du régime des brevets, ceux-ci sont par nature le résultat d'une recherche et la loi ne modifie rien à leurs conditions de rémunération, sous la seule réserve qu'elles fassent l'objet d'une convention.
En particulier la loi ne s'oppose pas au versement d'une rétribution proportionnelle au chiffre d'affaire total du produit couvert par le brevet.
L'interdiction de la proportionnalité s'applique à la valeur et au nombre des produits prescrits par le praticien et non à l'activité de l'entreprise ayant acquis le droit d'exploiter le brevet.

2 – Une procédure conventionnelle doit être impérativement respectée
Les avantages consentis par les entreprises aux professionnels de santé doivent faire l'objet d'une convention écrite. Les professionnels organisés en ordres doivent, avant la mise en application de la convention, la soumettre pour avis au conseil départemental de l'ordre auquel ils sont rattachés.
Si l'article L 365-1, alinéa 2, du code de la santé publique ne vise expressément que le seul ordre des médecins, les professions de chirurgien-dentiste et de sage-femme étant également organisées en ordres, les compétences reconnues au conseil de l'ordre des médecins à l'égard de ceux-ci doivent leur être également reconnues à l'égard de leurs ressortissants respectifs. En revanche les professions paramédicales pour lesquelles aucun ordre n'a été institué ne sont assujetties à cette formalité.
Par ailleurs, lorsque les activités de recherche ou d'évaluation sont réalisées même partiellement dans un établissement de santé, elles doivent être soumises pour avis au responsable de l'établissement.
Le souci de respecter la volonté de transparence qui sous-tend la disposition légale, sans pour autant tomber dans un formalisme paralysant, nous conduit à donner sur ses modalités concrètes d'application un certain nombre de précisions.

a) Forme et contenu de la convention
Afin notamment de permettre aux ordres concernés de donner un avis en connaissance de cause, la convention doit revêtir un caractère écrit et comporter un certain nombre d'indications (objet de l'activité en cause, leur, durée, nature et montant des avantages consentis et, s'agissant des manifestations ou actions de formation, programme faisant ressortir le temps consacré aux activités scientifiques par rapport à la durée totale).
Cela étant, la nature de l'écrit variera bien entendu selon l'objet de la convention en cause.
S'il prend parfois la forme d'un contrat en bonne et due forme, signé par les parties concernées, il n'en sera bien évidemment pas ainsi lorsqu'il s'agira d'une invitation à un congrès ou d'une action de formation continue. En ce cas la simple invitation adressée au professionnel vaut convention dès lors qu'elle contient des indications sur le contenu et le déroulement de la manifestation et qu'elle précise les prestations prises en charge par l'entreprise.

b) Convention transmise au conseil de l'ordre concerné sur le fondement d'un autre texte :
L'article L 462 du code de la santé publique fait notamment obligation aux médecins et aux chirurgiens-dentistes de communiquer au conseil départemental de l'ordre dont ils relèvent « les contrats et avenants ayant pour objet l'exercice de leur profession ». Dès lors qu'une convention aura été communiquée en application de cet article ou d'une autre disposition de même nature, il conviendra de considérer que la formalité prévue à l'article L 365-1 du code de la santé publique aura par-là même été remplie.
Il en sera notamment ainsi pour les expérimentations réalisées en application de la loi n° 88-1138 du 20 décembre 1988 (loi Huriet).
Pour les essais en phase I, II, III ayant fait l'objet d'un avis favorable du comité régional de protection des personnes dans la recherche biomédicale, il ne sera pas nécessaire de réétudier le contenu de la convention saut rémunération manifestement injustifiée du praticien et inadéquation de la prestation avec l'objectif recherché.
En revanche, pour les essais de phase IV, l'examen de la convention devra porter sur tous les aspects de celle-ci.

c) Date à laquelle la convention doit être soumise au conseil départemental de l'ordre concerné :
Aux termes du deuxième alinéa de l'article L 365-1, les conventions doivent être « soumises pour avis » avant leur mise en application. Il n'est pas en revanche nécessaire que l'avis ait lui-même été rendu avant l'exécution de la convention, ce qui poserait d'ailleurs souvent des problèmes pratiques.
Cela étant les agents chargés de veiller au respect de la loi devront bien entendu, toutes les fois que cela sera possible, vérifier que cet avis a bien été sollicité.

d) Recherches, congrès, actions de formation s'adressant à des professionnels sur l'ensemble du territoire ou sur des départements différents
Afin de simplifier le travail d'instruction des demandes par les conseils départementaux et d'assurer une certaine harmonisation de leurs interprétations, nous ne verrons que des avantages à ce que les conventions relatives à des recherches, congrès, séminaires, actions de formation continue ayant vocation à s'adresser à des professionnels sur l'ensemble du territoire ou même dans plusieurs départements différents soient adressées au conseil national de l'ordre concerné qui pourrait émettre sur le principe même de la convention un avis ne devrait pas porter sur la valeur scientifique des activités concernées, mais sur leur conformité aux règles de déontologie et sur le caractère scientifique des sujets abordés.
Les conseils départementaux, saisis de demandes individuelles, conserveraient bien évidemment leur liberté d'appréciation quant à l'avis à formuler sur chaque demande individuelle, compte tenu notamment de la qualification du praticien.

e) Le cas particulier des professionnels exerçant dans un établissement de santé :
L'article L 365-1 dispose que les conventions doivent être notifiées au responsable de l'établissement lorsque les activités de recherche ou d'évaluation sont effectuées même partiellement dans un établissement de santé.
S'agissant des établissements publics de santé, cette disposition ne fait que rappeler les règles générales applicables aux personnes travaillant dans ces établissements.
Il s'agit en l'occurrence d'une simple obligation d'information du responsable, celui-ci n'étant pas investi d'un pouvoir d'appréciation de la validité de l'activité de recherche ou d'évaluation.

III – Les prestations autres que celles relatives à des activités de recherche ou d'évaluation scientifiques réalisées par les praticiens

L'interdiction générale posée par la loi de versement par les entreprises de tout avantage en nature ou espèces ne vise évidemment pas à interdire les relations normales de travail, qu'il s'agisse de missions, de contrats de travail ou de participation à des conseils scientifiques.
Mais si la rémunération de telles activités était sans rapport avec les services effectivement rendus, elles tomberaient sous le coup de l'interdiction prévue à l'article L 365-1.
En tout état de cause, il appartient aux praticiens, conformément à l'article L 462 de communiquer aux conseils départementaux de l'ordre l'ensemble des contrats ayant pour objet l'exercice de leur profession.
Dans l'application des présentes instructions, nous vous demandons de vous attacher à rechercher les abus éventuels, en tenant compte du délai qui a été nécessaire pour prendre en compte les dispositions légales, et d'en faire connaître le sens et la portée aux professionnels avec lesquels vous serez en relation. La nécessité de sanctionner des comportements manifestement répréhensibles ne doit évidemment par être perçue comme traduisant une suspicion systématique à l'encontre de professionnels qui dans leur grande majorité sont soucieux d'exercer leurs activités en toute indépendance.

Le ministre délégué à la santé
Philippe DOUSTE-BLAZY

ANNEXE 2.4
BONNES PRATIQUES CLINIQUES

Ministère des Affaires Sociales
et de l'Emploi
Ministère chargé de la Santé
et de la Famille

Direction de la Pharmacie
et du Médicament

BONNES PRATIQUES CLINIQUES

Avis aux promoteurs et aux investigateurs pour des essais cliniques des médicaments

(Paru au Journal Officiel)

1987

Préambule

Les Bonnes Pratiques Cliniques (B.P.C.) sont définies par l'ensemble des dispositions à mettre en place pour assurer à des essais la qualité et l'authenticité de leurs données scientifiques d'une part, et le respect de l'éthique d'autre part.

Ce sont des recommandations de nature évolutive. Elles précisent les responsabilités respectives du promoteur et de l'investigateur, et supposent la mise en place d'un ensemble de contrôles adaptés.

04/01/89

Les Bonnes Pratiques Cliniques s'intègrent dans le système d'assurance de la qualité du médicament, système qui recouvre les phases de développement, de production et de dispensation. Elles visent à renforcer la maîtrise de la qualité des essais cliniques réalisés en France sur le médicament. Elles ne visent pas à apprécier la valeur scientifique intrinsèque d'une étude.

1. DEFINITIONS DE TERMES

1.1. Système d'assurance de la qualité
L'assurance de la qualité est obtenue par la «mise en œuvre d'un ensemble approprié de dispositions préétablies et systématiques destinées à donner confiance en l'obtention de la qualité requise».
Cet ensemble de dispositions est appelé système d'assurance de la qualité.

1.2. Contrôle de la qualité
Tout contrôle est une «vérification de la conformité à des données préétablies, suivie d'un jugement».
La vérification de la conformité d'un essai clinique aux B.P.C. est appelée contrôle de la qualité.

1.3. Promoteur
Le promoteur est un individu ou un organisme qui prend l'initiative de la réalisation d'un essai clinique.

1.4. Investigateur
L'investigateur est le responsable de la réalisation pratique de l'essai proposé par le promoteur, et de la synthèse des données recueillies sous forme d'un rapport.

1.5. Moniteur
Le moniteur est une personne choisie et mandatée par le promoteur, chargée d'assurer pour ce dernier le suivi de l'essai. Il sert de lien entre le promoteur et l'investigateur et rend compte de son activité au promoteur.

1.6. Lieu d'expérimentation
Le lieu de l'expérimentation est un établissement de soins public ou privé, un cabinet médical, ou, un centre disposant de l'équipement nécessaire à la réalisation d'essais cliniques, compatible avec les impératifs de rigueur scientifique et de sécurité.

1.7. Pré-requis
Les pré-requis sont constitués par les éléments d'information dont la connaissance est jugée nécessaire préalablement à la mise en œuvre de l'essai : données analytiques et galéniques, toxicologiques, pharmacocinétiques, pharmacologiques et cliniques antérieures.
Ils peuvent être présentés sous forme d'un document de synthèse confidentiel, appelé par exemple brochure d'investigation. L'investigateur a toujours la possibilité, outre ce document, d'accéder à l'intégralité des données disponibles.
Ces éléments d'information sont actualisés au fur et à mesure de la progression des travaux.

1.8. Protocole
Document qui définit l'objectif, les conditions de réalisation et le déroulement de l'essai.

1.9. Cahier d'observation (annexe au protocole)
Document destiné à recueillir au fur et à mesure de l'essai, pour chaque sujet les informations définies par le protocole.
Les informations peuvent être recueillies par tout moyen en garantissant l'édition et la conservation, et permettant le contrôle de la qualité.

1.10. Documents de levée d'anonymat (annexe au protocole)
Pour les essais réalisés en double insu, une procédure de levée d'anonymat doit être prévue afin de connaître, dans certains cas d'urgence, la nature du traitement attribué à l'un des sujets. Les documents de levée d'anonymat sont conservés dans des enveloppes opaques scellées ou par tout autre moyen approprié permettant de préserver le secret. Ces documents doivent être restitués au promoteur en fin d'expérimentation.

1.11. Procédures opératoires (annexe au protocole)
Instructions écrites décrivant les opérations à effectuer, les précautions à prendre et les mesures à appliquer, non détaillées dans le protocole.

1.12. Dossier du malade
Un dossier spécifique (par exemple dossier hospitalier, fiche de consultation...) doit exister pour chaque sujet inclus dans l'essai. Il permet notamment, dans le respect des dispositions réglementant la consultation de ce type de document, de vérifier l'authenticité des informations figurant dans le cahier d'observation et au besoin de les compléter ou de les corriger.

1.13. Produits étudiés
Il s'agit de tout produit étudié dans le cadre d'un essai. Dans les essais comparatifs, le produit pour lequel l'essai est entrepris est comparé à un ou des produits de référence ou à un placebo.

1.14. Lot
Quantité définie d'un produit, obtenue au cours d'un cycle donné de fabrication : la qualité essentielle d'un lot de fabrication est son homogénéité.

1.15. Contrat entre promoteur et investigateur
Ce contrat définit les relations entre le promoteur et l'investigateur, il comporte :
- des dispositions générales : répartition précise des tâches entre les deux parties, durée prévue pour la réalisation de l'essai, conditions de publication des travaux et dispositions couvrant l'essai sur le plan de la responsabilité civile et de la confidentialité
- éventuellement des dispositions autres, notamment financières, qui sortent du cadre des B.P.C.
  NB : La réalisation d'un essai peut également donner lieu à la conclusion d'autres contrats entre le promoteur et l'établissement de soins, entre le promoteur et d'autres prestataires impliqués dans la mise en œuvre et le suivi de l'essai.

2. RESPONSABILITES DU PROMOTEUR

2.1. Choix de l'investigateur
Le promoteur choisit le (ou les) investigateur(s) chargé(s) de la réalisation de l'essai.

2.2. Choix du moniteur
Le promoteur choisit et mandate le (ou les) moniteur(s) chargé(s) d'assurer le suivi de l'essai.

2.3. Relations promoteur - investigateur
Elles s'effectuent par l'intermédiaire du moniteur.

2.3.1 Visites initiales
Préalablement à la mise en place de l'essai, le moniteur rend visite à l'investigateur choisi et, à cette occasion, s'assure de :
- La qualification de l'investigateur
- Des moyens de l'investigateur pour mener à bien l'essai demandé en terme de :
    - disponibilité
    - recrutement des sujets
    - plateau technique et environnement
- La compatibilité de l'essai proposé avec les autres projets de recherche de l'investigateur sur les mêmes types de sujets
- Remet les pré-requis à l'investigateur
- Reçoit de l'investigateur un document décrivant l'équipe qui participera de prés ou de loin à cette étude avec noms et fonctions de l'essai
- Communique à chaque investigateur le nom des autres investigateurs qui effectuent des essais sur le même médicament

- Informe l'investigateur des procédures prévues en cas d'événements critiques et des possibilités de joindre en urgence la (ou les) personne(s) compétente(s) désignée(s) par le promoteur
- Procède à la discussion détaillée du protocole et de ses annexes avant acceptation écrite par le promoteur et l'investigateur
- Recueille toutes les normes et méthodes des examens qui seront pratiqués durant l'essai
- Vérifie les conditions de stockage des produits étudiés
- Vérifie l'acceptation écrite des dispositions du contrat par les deux parties
- S'assure de l'engagement écrit du promoteur et de l'investigateur à se conformer aux Bonnes Pratiques Cliniques en ce qui les concerne, et à accepter les contrôles prévus.

2.3.2 Visites périodiques

Le moniteur maintient un contact personnel avec l'investigateur en se rendant sur place périodiquement et en réunissant si nécessaire l'équipe participant à l'essai, et s'assure de :

- L'adhérence au protocole et à ses annexes : il cherche notamment à obtenir le maximum d'informations sur les données manquantes
- La conformité des données du cahier d'observation avec le dossier du malade
- L'utilisation des produits étudiés selon les dispositions prévues par le protocole
- La conservation en accord avec la réglementation, des données brutes permettant de vérifier les informations portées sur le cahier d'observation (examens biologiques, radiographies...)
- L'acceptation écrite par le promoteur et l'investigateur des éventuelles modifications du protocole et de ses annexes.

2.4. Consultation d'un « comité d'éthique »

Le promoteur prend connaissance, avant de débuter l'essai, de l'avis obtenu auprès d'un «comité d'éthique» par l'investigateur.

Le promoteur et l'investigateur s'engagent à n'accepter aucune modification significative du protocole sans notification au «comité d'éthique».

2.5. Responsabilités relatives aux effets indésirables

Le promoteur notifie au Système National de Pharmacovigilance les effets indésirables jugés graves dont il a connaissance dans le cadre de l'essai.

Le promoteur informe l'investigateur et le Système National de Pharmacovigilance des effets graves constatés lors d'autres essais simultanés, même effectués à l'étranger.

2.6. Responsabilités relatives aux produits étudiés

Le promoteur est responsable de l'adéquation de la présentation des produits étudiés à l'usage prévu par le protocole.

Un échantillon représentatif de chacun des lots utilisés dans l'essai est conservé pour permettre tout contrôle éventuel.

Le promoteur tient une comptabilité des quantités de produits adressées pour l'essai à l'investigateur avec les numéros de lots correspondants.

Il s'assure du devenir de ces produits :

- réception selon les modalités d'approvisionnement établies
- Conservation dans les conditions prévues par le protocole
- Utilisation exclusivement dans les limites prévues par le protocole
- Devenir des produits non utilisés, qui sont soit récupérés, soit détruits par une personne habilitée.

Lorsque le pharmacien de l'établissement de soins est associé à la mise en place de l'essai, il doit disposer d'informations suffisantes.

2.7. Archivage
Le promoteur organise le stockage dans des locaux appropriés des documents suivants :
- Pré-requis,
- Protocole et annexes,
- Données individuelles,
- Documents de suivi,
- Rapport final,
- Analyses statistiques.

Le matériel archivé est indexé de manière à faciliter un stockage ordonné et une recherche rapide. La durée de conservation est de dix ans après la fin de l'essai.

2.8. Obligations réglementaires
Le promoteur vérifie que les dispositions réglementaires sont respectées.

2.9. Cas particulier : l'organisme prestataire de service
Le promoteur peut déléguer tout ou partie de ses droits et devoirs à un organisme prestataire de service. Cette délégation n'exempte pas le promoteur de sa propre responsabilité.

3. RESPONSABILITES DE L'INVESTIGATEUR

3.1 Avant le début de l'essai

3.1.1. Qualification
L'investigateur doit avoir une qualification lui permettant d'assurer la responsabilité de l'essai. Dans le cas particulier où l'investigateur n'est pas réglementairement habilité à recruter des patients, à prescrire une thérapeutique, à dispenser des soins et à assurer une surveillance clinique, il doit recourir à la participation d'une personne habilitée.
Il est tenu compte dans la définition de l'aptitude à pratiquer des essais cliniques de la formation initiale de l'investigateur, de son expérience professionnelle, de sa participation à des essais cliniques antérieurs. Un curriculum vitae reprenant ces différents éléments est demandé par le promoteur.

3.1.2. Connaissance des produits étudiés
L'investigateur prend connaissance des pré-requis et demande au promoteur toute information ou précision complémentaire qu'il juge utile.
Il vérifie par ailleurs l'adéquation des produits étudiés à l'usage prévu par le protocole (conditionnement, date de péremption) et les conditions de stockage.

3.1.3. Connaissance du protocole et de ses annexes
L'investigateur concourt à l'élaboration, avec le promoteur, du protocole et de ses annexes (cahiers d'observation, documents de levée d'anonymat,...) avant de les accepter par écrit.

3.1.4. Disponibilité
La disponibilité de l'investigateur et de son équipe doit être compatible avec la réalisation de l'essai.
L'investigateur s'assure notamment que l'essai proposé ne sera pas perturbé par d'autres essais éventuels.

3.1.5. Adéquation des locaux et des équipements
L'investigateur organise les structures techniques dont il dispose pour assurer, en fonction de la nature de l'essai :
- La mise en place de circuits spécifiques à l'essai (pour les sujets, les prélèvements...)
- La sécurité des sujets, en particulier dans les situations d'urgence
- Le stockage des lots de produits dans un lieu bien défini, garantissant leur bonne conservation et leur inaccessibilité à des tiers
- L'archivage des documents pendant la durée de l'essai, et après l'essai.

3.1.6. Recrutement des sujets

Préalablement à la signature du protocole, l'investigateur s'assure qu'il pourra disposer d'un recrutement de sujets suffisant pour garantir le bon déroulement de l'essai.

Lors du recrutement, l'investigateur doit être particulièrement vigilant sur l'existence d'éléments de nature à perturber l'essai, par exemple :

- Impossibilité de suivi des sujets (sujet résidant à grande distance du lieu de l'essai)
- Inaptitude de certains sujets à suivre les contraintes du protocole (barrière linguistique, intellectuelle...)
- Interférences possibles (médecins traitants, autres centres hospitaliers...).

3.1.7. Consentement des sujets

L'investigateur s'engage à recueillir le consentement des sujets. Il précise clairement le contenu de l'information donnée aux sujets et les modalités du recueil de leur consentement.

En cas d'essai sans objectif thérapeutique direct pour le sujet, le consentement est normalement recueilli par écrit.

3.1.8. Avis d'un « comité d'éthique »

L'investigateur saisit le « comité d'éthique » et en communique l'avis au promoteur.

3.1.9. Acceptation du contrat

Après discussion du projet de contrat avec le promoteur, l'investigateur exprime son accord par écrit.

3.1.10. Engagement de l'investigateur

L'investigateur s'engage par écrit à se conformer aux Bonnes Pratiques Cliniques en ce qui le concerne ; il accepte notamment les visites périodiques du moniteur et ce qu'elles impliquent. Il accepte également le principe d'un contrôle par les autorités de tutelle.

3.1.11. Constitution de l'équipe

Pour l'ensemble du personnel assurant la continuité des services de soins (jour, nuit, garde) l'investigateur :

- Définit par écrit les rôles respectifs des différents membres de l'équipe impliquée dans l'essai en fonction de leur qualification
- S'assure que le personnel est informé du protocole en cours, et a bien compris les fonctions qu'il doit remplir dans le cadre de l'essai
- Prend en charge la formation nécessaire à ces fonctions ;
- Désigne la (ou les) personne(s) spécifiquement chargée(s) de la gestion administrative de l'essai
- S'assure que les autres départements ou services impliqués dans la réalisation pratique de l'essai sont informés de la mise en œuvre de l'étude et détermine avec eux des procédures opératoires spécifiques.

3.2. Pendant l'essai

3.2.1. Respect du protocole et de ses annexes

L'investigateur s'assure que le protocole et ses annexes sont scrupuleusement suivis, notamment lorsque d'autres services sont impliqués dans l'essai. Un répertoire des procédures opératoires est conservé.

L'investigateur discute des éventuelles modifications de l'ensemble de ces documents avec le promoteur avant de les accepter par écrit. Lorsque ces modifications sont jugées significatives, l'investigateur les notifie au « comité d'éthique ».

3.2.2. Recueil des données

L'investigateur veille à la qualité du recueil des données dans les cahiers d'observation.

Les données obtenues au cours de l'essai sont enregistrées directement selon la chronologie prévue, de façon lisible et indélébile. Toute modification des données brutes est datée, signée et motivée. L'enregistrement original ne doit pas être masqué.
Les données entrées sur support magnétique doivent être convenablement identifiées. Leurs éventuelles modifications répondent aux procédures définies pour un support papier.
L'investigateur transmet les bordereaux éventuellement requis selon les modalités prévues par le protocole.

3.2.3. Gestion des produits étudiés
L'investigateur gère le stock de produits, éventuellement en relation avec le pharmacien de l'établissement si l'essai se déroule dans un établissement de soins.

3.2.4. Evénements critiques
L'investigateur informe le plus rapidement possible le promoteur de tout événement critique survenant au cours de l'essai. Les décisions cliniques individuelles concernant les événements critiques non prévus par le protocole relèvent de la seule responsabilité de l'investigateur. Si ces événements entraînent une modification significative du protocole, cette modification est notifiée au « comité d'éthique ».
Toute levée d'anonymat est signalée et motivée par l'investigateur. Elle doit rester une procédure exceptionnelle.

3.2.5. Disponibilité
L'investigateur ménage le temps nécessaire pour les visites de contrôle de qualité effectuées au cours de l'essai, et les réunions préalables à la rédaction du rapport final.

3.3. Après l'essai
L'investigateur s'assure du devenir de l'ensemble du matériel (documents, produits, équipements...) relatif à l'essai, en accord avec le promoteur. Les données brutes sont conservées pendant dix ans par l'investigateur.
Chaque investigateur date et signe le rapport final ce qui implique qu'il accepte la responsabilité de la validité des données et confirme qu'elles ont été obtenues conformément aux principes des Bonnes Pratiques Cliniques.
Les responsables de l'analyse des résultats signent également le rapport final.
Les corrections et additions à un rapport final doivent être apportées sous forme d'amendement motivé, signé et daté par le ou les investigateurs.
L'analyse statistique des données peut être assurée ou commanditée par le promoteur ou l'investigateur.

4. ASPECTS SPECIFIQUES DE CERTAINS ESSAIS

4.1. Essai multicentrique
Un essai multicentrique est un essai réalisé simultanément par plusieurs investigateurs travaillant dans des centres différents avec des méthodes identiques et selon un protocole commun, dans le but de réunir plus rapidement des données pour une analyse globale aboutissant à la rédaction d'un rapport commun et unique.

Un essai multicentrique nécessite de mettre en place un système de coordination pour animer et contrôler la mise en œuvre et le déroulement de l'essai. En effet, certaines étapes sont rendues plus complexes :
élaboration, discussion et acceptation écrite du protocole et de ses annexes par tous les investigateurs (lorsque des investigateurs sont recrutés à la suite de la défection de certains, centres l'élaboration conjointe du protocole n'est pas toujours réalisable) :
- organisation des réunions préalables et intermédiaires
- mise en place de l'étude

- préparation ou contrôle de la randomisation, du conditionnement des produits destinés à l'essai
- formation des investigateurs au même protocole
- standardisation des méthodes d'évaluation
- centralisation des données
- contrôle de l'adhérence au protocole
- rédaction du rapport final

Il peut être utile par ailleurs de disposer d'un comité de surveillance, interlocuteur du comité d'éthique auquel il notifie ses décisions. Ce comité de surveillance discute d'éventuelles modifications du protocole, rend des arbitrages concernant le suivi, la poursuite de l'essai, l'interprétation des résultats. Ses membres sont habituellement extérieurs à la structure de coordination.

4.2 Essai en cabinet médical
Effectué en médecine générale ou en médecine spécialisée, cet essai est habituellement multicentrique.
Il peut être entrepris pour tout type d'essai réalisable en médecine ambulatoire ; s'agissant d'un essai en cabinet médical, l'investigateur doit cependant être particulièrement vigilant sur certaines de ses obligations :
- disposer des moyens nécessaires à la réalisation de l'essai
- l'engager à accepter les contraintes de l'essai
- présenter une disponibilité et des possibilités de recrutement compatibles avec la réalisation de l'essai.

Toutes les dispositions décrites dans les chapitres généraux sont compatibles avec la réalisation d'un essai en cabinet médical.

4.3 Essai sans objectif thérapeutique
Il s'agit de tout essai destiné à améliorer la connaissance du produit, sans qu'un objet thérapeutique direct soit recherché pour le sujet participant.
Une attention particulière doit être portée sur les points suivants :
- Nécessité d'une compétence spécifique de l'investigateur
- Conditions nécessaires et suffisantes d'installation des locaux permettant d'assurer la sécurité des sujets participants
- Modalités et procédures de recrutement des sujets participants et notamment évaluation de la qualité de « volontaire sain »
- Condition d'indemnisation des sujets
- Consentement des sujets, normalement par écrit.

5. RESPONSABILITES DU COMITE D'ETHIQUE

5.1 Consultation du comité d'éthique
Les comités d'éthique sont saisis par les investigateurs. Ils ont un rôle consultatif.

5.1.1 Au cours d'un examen initial
L'essai ne peut débuter qu'après avis du dit comité dans les délais précis, prévus dans son règlement intérieur ; cet avis porte :
- L'appréciation de l'investigateur pour l'essai projeté, et l'adéquation des moyens dont il dispose
- La pertinence générale du protocole soumis par l'investigateur
- L'adéquation entre l'objectif de l'essai, ses risques potentiels et les désagréments liés aux modalités prévues par le protocole
- L'information destinée aux sujets ou à leurs parents ou tuteurs ainsi que les modalités de recueil du consentement. Dans les cas exceptionnels où le consentement ne peut être obtenu, les raisons doivent en être fournies l'existence d'une assurance couvrant la responsabilité civile de l'investigateur et du promoteur.

5.1.2 En cours d'essai, en cas d'éléments nouveaux significatifs
Le comité d'éthique est saisi lorsque des éléments nouveaux entraînent une modification significative du protocole ou sont susceptibles de remettre en cause l'avis donné initialement.

5.2 Echanges entre comité d'éthique et investigateur
Le comité d'éthique transmet par écrit les avis émis à l'investigateur. La correspondance avec l'investigateur et les documents de base soumis sont conservés pendant une période de 10 ans suivant la date du rapport final.

6. CONTROLE DE LA QUALITE

Le contrôle de la qualité mis en place permet de s'assurer que l'essai clinique est bien réalisé conformément aux principes de B.P.C.
Il se situe à trois niveaux.

6.1 Vérification de la qualité par l'investigateur
L'investigateur, responsable de la réalisation pratique de l'essai, s'assure de la qualité du travail de l'équipe, notamment en ce qui concerne le respect du protocole et de ses annexes, le recueil des données, la gestion des produits étudiés.

6.2 Vérification de la qualité par le promoteur
Ce contrôle est effectué par une ou plusieurs personnes (éventuellement par le moniteur), ou par un organisme prestataire de services, ou par toute autre structure désignée par le promoteur, à condition qu'en soient exclus les investigateurs de l'essai. Il est important que la nature et la fréquence des contrôles fassent l'objet d'un consensus entre l'investigateur et le promoteur.
Ce contrôle a pour objet de vérifier, notamment par des visites régulières, les moyens et conditions de réalisation de l'essai, et d'évaluer en conséquence la qualité et la fiabilité des données et informations transmises au promoteur. Les comptes rendus de ces visites doivent être conservés.
Le promoteur et l'investigateur doivent être informés, périodiquement et en tant que de besoin, des conclusions de ces visites et des actions correctives éventuelles à effectuer.
Une attestation de conformité aux B.P.C. est incluse dans le rapport final.

ANNEXE 2.5
AVIS DU CPP

COMITE DE PROTECTION DES PERSONNES ILE DE FRANCE X
HOPITAL ROBERT BALLANGER
Boulevard Robert Ballanger - Bâtiment n° 8 (3ème étage) - 93602 Aulnay-sous-bois cedex
Tél. : 01 49 36 73 57 - E-mail : cpp.iledefrance10@ch-aulnay.fr
Président : Pr. Philippe CASASSUS

Recommandée avec AR
N° 1A 052 185 8259 1

Monsieur Marc PIOCHAUD
SPRIT
7, rue Lallier
75009 PARIS

Aulnay-sous-Bois, le 09 juin 2011

Vos réf. : OSTP-EUR-10-01
Nos réf : PC/AP 19-2011
N° ID RCB : 2011-A00258-33

Cher Monsieur,

Le Comité de Protection des Personnes Ile-de-France X a été saisi par vous-même de la demande d'avis sur le projet de recherche biomédicale portant sur un dispositif médical ou sur un dispositif médical de diagnostic in vitro intitulé :

« Etude clinique d'évaluation de l'efficacité d'osténil® plus (acide hyaluronique) versus synvisc-one™ dans le traitement de la gonarthrose fémoro-tibiale. Etude en double aveugle randomisée contrôlée d'osténil® plus versus synvisc-one™ de 2 groupes parallèles de patients suivis pendant 6 mois. »
dont TRB Chemedica est le promoteur.

Au reçu des modifications apportées selon ses recommandations, le Comité émet un **AVIS FAVORABLE à la mise en œuvre de cette étude**, au regard des documents suivants :

- Courrier de réponse .... daté du 27 mai 2011
- Formulaire de demande d'avis initial au CPP sur une recherche biomédicale (DM) .... daté du 08 avril 2011
- Document additionnel à la demande d'avis au CPP (DM) .... daté du 08 avril 2011
- Reçu de confirmation du n° d'enregistrement de la recherche .... daté du 18 février 2011
- Autorisation écrite du promoteur pour délégation .... daté du 07 avril 2011
- Attestation d'assurance : Chubb Insurance Company Europe SE .... version 2 du 07 avril 2011
- Protocole de recherche .... version 3 du 16 mai 2011
- Résumé de l'étude ....
- Note d'information au patient .... version du 16 mai 2011
- Formulaire de consentement de participation .... version du 16 mai 2011
- Liste des investigateurs participants à l'étude ....
- CV de l'investigateur coordonnateur et des investigateurs participants ....

COMITE DE PROTECTION DES PERSONNES ILE DE FRANCE X

HOPITAL ROBERT BALLANGER

Boulevard Robert Ballanger - Bâtiment n° 8 (3ème étage) - 93602 Aulnay-sous-bois cedex

Tél. : 01 49 36 73 57 - E-mail : cpp.iledefrance10@ch-aulnay.fr

Président : Pr. Philippe CASASSUS

- Cahier d'observation du médecin évaluateur .... version 4 du 16 mai 2011
- Cahier d'observation du médecin injecteur .... version 4 du 16 mai 2011
- Cahier patient ....
- Brochure investigateurs du Ostenil® Plus *en anglais* .... version du 23 mars 2011
- Notices d'information des dispositifs médicaux :
  - Ostenil® Plus (DM de classe III) ....
  - Synvisc® (DM de classe III) ....

Avaient participé à la délibération les membres du Comité suivants :

<u>Recherche biomédicale</u>	:	Monsieur Philippe CASASSUS (*)........................ titulaire<br>Monsieur Jean-Luc GAILLARD .... titulaire<br>Madame Ilham MOUMNA............................... suppléante
<u>Médecin généraliste</u>	:	Madame Elisabeth HENON................................. titulaire
<u>Pharmacien hospitalier</u>	:	Madame Patricia LEROUX.................................. titulaire
<u>Associations agréées de malades ou d'usagers du système de santé</u>	:	Madame Marie-Claude FEINSTEIN .... titulaire<br>Monsieur Philippe MAUGIS.............................. suppléant

Avaient assisté au débat sans participation les suppléants suivants :

<u>Médecin généraliste</u>	:	Monsieur Daniel FAUCHER .... suppléant

(*) : épidémiologiste / biostatisticien

Je vous prie de croire, Cher Monsieur, en l'expression de mes sentiments les meilleurs.

Le Président,
Professeur Philippe CASASSUS

[CET AVIS COMPORTE DEUX PAGES]

Au cas où il apparaîtrait nécessaire de présenter ultérieurement à notre Comité ***<u>une modification substantielle au protocole</u>****, nous vous prions de nous adresser la* ***nouvelle version du protocole, ou de la notice d'information, ou de la feuille de consentement, en indiquant de façon facilement visible*** *(<u>en gras, ou surligné en couleur, ou marqué à la marge, ou sur des feuilles spécifiques indiquant l'ancienne et la nouvelle version</u>)* ***les modifications proposées.***

ANNEXE 2.6
AVIS DE L'AFSSAPS

RÉPUBLIQUE FRANÇAISE

afssaps

Agence française de sécurité sanitaire des produits de santé

Direction de l'évaluation des dispositifs médicaux
Unité essais cliniques (UEC)
Dossier suivi par Lynda ARNAUD-BOISSEL
Tél. : +33 (0)1 55 87 37 53
Fax. : +33 (0)1 55 87 37 17
E-mail : dedim.dm@afssaps.sante.fr
N/Réf. : UEC/LynAB/DA/2011-

Saint-Denis, le **20 MAI 2011**

Objet : ESSAI CLINIQUE D'EVALUATION DE L'EFFICACITE D'OSTENIL®PLUS (ACIDE HYALURONIQUE) VERSUS SYNVISC-ONE™ DANS LE TRAITEMENT DE LA GONARTHROSE FEMORO-TIBIALE
N° d'enregistrement : 2011-A00258-33

Monsieur,

Vous avez adressé à mes services une demande d'autorisation pour la recherche biomédicale référencée ci-dessus.

Au vu des réponses apportées le 17 mai 2011 (protocole de version 3 du 16 mai 2011) aux objections émises par l'Afssaps par courrier en date du 10 mai 2011, j'autorise cette recherche biomédicale en France. En vertu de l'article L. 1121-4 du code de la santé publique, je vous précise toutefois que cette recherche biomédicale doit bénéficier d'un avis favorable du CPP pour pouvoir être mise en place en France.

Je vous rappelle notamment que, pendant le déroulement de la recherche et pour ce qui concerne l'Afssaps, toute modification substantielle du dossier initialement soumis doit faire l'objet d'une demande d'autorisation en vertu des articles L. 1123-9 et R. 1123-35 du code de la santé publique. Les effets/évènements indésirables graves ainsi que les faits nouveaux susceptibles de porter atteinte à la sécurité des personnes sont à déclarer en vertu de l'article L. 1123-10 du code de la santé publique, la fin de l'essai est également à déclarer en vertu de l'article L. 1123-11 du code de la santé publique.

Je vous prie d'agréer, Monsieur, l'expression de mes salutations distinguées.

Le chef de Département surveillance de marché

Nicolas THEVENET

SPRIT
7, rue Lallier
75009 Paris

A l'attention de Monsieur Marc PIOCHAUD

Copie : CPP Ile de France X

ANNEXE 2.7
NOTICE D'INFORMATION
AU PATIENT

NOTICE D'INFORMATION AU PATIENT

ESSAI CLINIQUE D'EVALUATION DE L'EFFICACITE D'OSTENIL® PLUS (ACIDE HYALURONIQUE) VERSUS SYNVISC-ONE™ DANS LE TRAITEMENT DE LA GONARTHROSE FEMORO-TIBIALE
ETUDE EN DOUBLE AVEUGLE RANDOMISEE CONTROLEE D'OSTENIL® PLUS VERSUS SYNVISC-ONE™ DE DEUX GROUPES PARALLELES DE PATIENTS SUIVIS PENDANT SIX MOIS

Nous vous demandons de bien vouloir lire attentivement ce document constitué en application de la loi du 20 décembre 1988 relative à la protection des personnes se prêtant à la recherche biomédicale.

Madame, Monsieur,

Vous souffrez d'une gonarthrose fémoro-tibiale qui se manifeste par des douleurs et une gêne fonctionnelle.
La gonarthrose est l'arthrose touchant le genou. En général, il s'agit d'une arthrose entre fémur et tibia, dite fémoro-tibiale.

L'arthrose est une maladie de l'articulation qui touche en premier lieu le cartilage, mais peut aussi affecter l'os sous le cartilage et les structures qui constituent et entourent l'articulation. Le cartilage articulaire perd peu à peu ses propriétés de résistance au choc et d'élasticité. Les chocs, traumatismes ou pressions s'exerçant sur l'articulation sont moins bien amortis. Le liquide articulaire qui a aussi une fonction d'amortisseur et de lubrifiant perd ses qualités. L'articulation est moins lubrifiée, moins élastique et moins résistante aux pressions. L'os sous le cartilage est donc plus sollicité et peut réagir, douloureusement notamment.
La plupart du temps l'évolution de l'arthrose est lente et se traduit par des douleurs mécaniques : par exemple en marchant ou en descendant les escaliers dans le cas d'une arthrose fémoro-tibiale, comme la vôtre. Ces douleurs s'accompagnent d'une gêne fonctionnelle plus ou moins invalidante dans la vie quotidienne et de phénomènes d'enraidissement, notamment au démarrage après une position immobile prolongée (le matin, ou en se relevant d'une position assise, par exemple). Parfois, l'évolution de cette arthrose est entrecoupée d'une poussée nettement plus douloureuse et inflammatoire avec gonflement de l'articulation (à cause de la présence d'un épanchement articulaire, particulièrement visible au genou, et que l'on peut ponctionner).

Le traitement de la gonarthrose fait appel à différentes mesures physiques et médicamenteuses. Les récentes recommandations européennes pour le traitement de la gonarthrose ont recensé de très nombreuses possibilités thérapeutiques :
- médicamenteuses : antalgiques, anti-inflammatoires, produits symptomatiques d'action lente et prolongée par voie orale, injections intra-articulaires de corticoïdes ou injections intra-articulaires d'acide hyaluronique.
- mais aussi non médicamenteuses tels que la réduction du poids, le port de semelles adaptées, la pratique d'exercices physiques de renforcement musculaire des quadriceps, la kinésithérapie, la physiothérapie, les conseils de réduction du surmenage articulaire, l'utilisation d'une canne pour marcher, le port d'une genouillère, les cures thermales, l'éducation et l'information du patient sur son arthrose, etc.

Parmi tous ces traitements, les injections d'acide hyaluronique intra-articulaire ont vu leur efficacité reconnue. Elles agissent sur les symptômes tels que la douleur et la gêne fonctionnelle. L'acide hyaluronique est un constituant naturel important du cartilage et du liquide articulaire. Sa fonction est probablement de donner à ceux-ci une meilleure résistance aux chocs.
Sa concentration et sa masse moléculaire diminuent dans le liquide articulaire en cas d'arthrose. L'idée d'injecter de l'acide hyaluronique exogène (extérieur) pour restaurer une meilleure concentration et promouvoir la synthèse (fabrication) d'un acide hyaluronique de qualité meilleure a paru séduisante et conduit à l'essayer dans la gonarthrose.

Depuis le début des années 1980, plus d'une centaine d'études ont été réalisées chez des patients gonarthrosiques montrant l'efficacité de ces injections intra-articulaires et leur bonne tolérance : il y a eu en effet peu d'effets secondaires; il s'agissait généralement de réactions locales peu intenses, de courte durée et guérissant spontanément.

L'acide hyaluronique intra-articulaire est donc indiqué chez les patients gonarthrosiques souffrant malgré la prise d'antalgiques ou supportant mal les antalgiques ou les anti-inflammatoires.

Les modalités d'administration sont de trois injections consécutives à raison d'une injection intra-articulaire par semaine.
Cependant récemment les laboratoires TRB CHEMEDICA ont mis au point une nouvelle forme permettant une seule et unique injection.

Le but de cet essai clinique multicentrique et randomisé, dont le promoteur est le laboratoire TRB CHEMEDICA, auquel nous vous proposons de participer est d'apprécier l'efficacité et la tolérance d'OSTENIL® PLUS par comparaison à un autre acide hyaluronique qui existe déjà sous la forme d'une injection : le SYNVISC-ONE™ de poids moléculaire différent.

Cette étude durera six mois durant lesquels vous bénéficierez d'une surveillance très attentive de l'évolution de votre arthrose, même si vous arrêtez l'essai. Un seul genou fera l'objet de l'évaluation et sera traité. Votre médecin vous donnera toutes les informations sur le calendrier des visites ainsi que sur le déroulement de l'étude.

Lors de la première consultation, après que votre médecin vous ait expliqué le but de l'essai et que vous ayez accepté d'y participer, vous irez chez un autre praticien soit un rhumatologue, soit un médecin de rééducation physique, soit un chirurgien orthopédique, qui procédera le jour même (si possible) ou dès le lendemain à l'injection intra-articulaire de l'un ou l'autre des produits qui vous sera attribué de façon aléatoire: OSTENIL® PLUS ou SYNVISC-ONE™.
Cette procédure est nécessaire afin que ni votre médecin, ni vous-même ne sachiez quel acide hyaluronique vous a été injecté, ceci afin d'éviter de favoriser l'un ou l'autre produit.

Vous viendrez consulter à nouveau trois fois votre médecin, les 30ème 90ème et 180ème jours après votre entrée dans l'étude, afin qu'il évalue avec vous votre gonarthrose. Il y aura donc au total quatre consultations, plus une visite pour l'injection. Il vous sera demandé de noter dans un carnet personnel fourni au début de l'étude tous les traitements que vous prenez pour soigner votre gonarthrose.

L'injection de l'acide hyaluronique étant strictement intra-articulaire, les précautions générales liées à ce type d'injection doivent être respectées. Il faut éviter l'injection dans les vaisseaux sanguins et dans les tissus environnants. Une attention particulière doit être portée aux patients ayant une hypersensibilité connue aux médicaments.
Les contre-indications à l'utilisation d'OSTENIL® PLUS sont les antécédents d'hypersensibilité à l'un des constituants.

Les risques potentiels de ce traitement sont les suivants :

1. Réaction douloureuse pendant l'infiltration, comme avec toutes les autres infiltrations, ou réaction pendant l'injection du produit, comme avec n'importe quel produit à usage intra-articulaire. Cette réaction est de courte durée et ne nécessite aucun traitement.
2. Réaction douloureuse au point d'injection après l'infiltration. Elle est peu fréquente et de courte durée, ne nécessitant pas de traitement particulier en général.
3. Dans quelques rares cas, il peut y avoir 24 à 48 heures après l'injection une réaction très douloureuse et inflammatoire avec parfois un épanchement et une sensation de chaleur. Celle-ci nécessite un traitement par le repos, l'application de glace localement et éventuellement la prise d'un anti-inflammatoire pendant quelques jours. Cette « arthrite » régresse et n'empêche pas la poursuite du traitement la plupart du temps.
4. Enfin, il faut signaler la possibilité très rare mais grave de survenue d'une infection dans l'articulation (arthrite septique). Cette arthrite septique se traduit par une augmentation importante de vos douleurs, avec gonflement du genou, rougeur et chaleur locale, plus éventuellement de la fièvre. L'apparition d'un ou de plusieurs de ces symptômes doit vous faire immédiatement contacter votre rhumatologue. Un tel accident nécessite en effet un traitement et parfois une hospitalisation en urgence.
5. Il n'y a pas d'effets généraux avec ce traitement.

Votre praticien connaît bien toutes ces réactions, car il utilise souvent le traitement par injection d'acide hyaluronique dans le genou. Vous devez lui signaler tout problème qui surviendrait pendant ou juste après l'injection, ou pendant toute la durée de l'étude.

Durant toute la durée de cette étude, vous ne pouvez participer à un autre essai clinique. La période d'exclusion pour la participation à un autre essai s'élève à 3 mois après la fin de la présente étude.

Les données médicales et informations vous concernant, feront l'objet d'un traitement informatique.
Ces informations vous concernant seront codées et identifiées seulement par un numéro de façon à préserver votre anonymat.
Elles ne seront transmises au promoteur et le cas échéant aux autorités sanitaires habilitées que dans des conditions garantissant leur confidentialité.
Le fichier informatique utilisé pour réaliser la présente recherche a fait l'objet d'une autorisation de la CNIL en application des articles 40-1 et suivant la loi "Informatique & Libertés".
Ces informations pourront être contrôlées selon la réglementation en vigueur.

Les résultats de cette étude pourront être publiés dans une revue médicale ou présentés aux autorités administratives sans que votre identité ne soit révélée.
Conformément à l'article L-1122-1 du Code de la Santé Publique, vous pourrez, si vous le désirez, être informé des résultats globaux de la recherche à laquelle vous acceptez de participer.
Vous serez informé des résultats globaux de cette étude par votre praticien.

Conformément à la loi Huriet du 20 décembre 1988 et ses décrets, le laboratoire TRB CHEMEDICA promoteur de cet essai a souscrit une assurance responsabilité civile pour la protection des participants. Cette assurance couvre les risques liés à l'étude pouvant survenir pendant son déroulement.

Selon la loi française, cette recherche a reçu l'avis favorable du Comité de Protection des Personnes Ile de France X.

Conformément à la loi du 20 décembre 1988, la confirmation par écrit de votre accord est préalablement nécessaire.
Cependant, vous avez le droit de refuser de participer à la recherche ou de retirer votre consentement à tout moment pendant le déroulement de l'étude sans encourir aucune responsabilité, sans que ceci ne modifie vos soins et votre bonne relation avec votre médecin. Il en est de même en cas de suspension ou d'interruption anticipée de l'étude.

Date :
Nom du médecin :
Signature :

Date :
Signature du patient :
(précédée de la mention " Lu et approuvé ")

ANNEXE 2.8
CONSENTEMENT ECLAIRE
DU PATIENT

CONSENTEMENT ECLAIRE DU PATIENT

ESSAI CLINIQUE D'EVALUATION DE L'EFFICACITE D'OSTENIL® PLUS (ACIDE HYALURONIQUE) VERSUS SYNVISC- ONE™ DANS LE TRAITEMENT DE LA GONARTHROSE FEMORO-TIBIALE
ETUDE EN DOUBLE AVEUGLE RANDOMISEE CONTROLEE D'OSTENIL® PLUS VERSUS SYNVISC-ONE™ DE DEUX GROUPES PARALLELES DE PATIENTS SUIVIS PENDANT SIX MOIS

Praticien (nom & prénom) ______________________________

Identification du patient (nom & prénom) ______________________________

Adresse : ______________________________

- J'ai reçu et lu la notice d'information de l'étude OSTENIL®PLUS concernant la solution viscoélastique 2% de hyaluronate de sodium dans le traitement de l'arthrose du genou versus SYNVISC-ONE™.
- J'ai noté que le produit est destiné à améliorer les symptômes de l'arthrose à moyen et long terme. J'ai aussi noté que l'étude à laquelle il m'est proposé de participer comporte une évaluation de l'efficacité des traitements.
- J'ai discuté librement avec mon praticien des effets favorables espérés et des risques potentiels.
- J'accepte les conditions de suivi, en particulier le principe de consulter à quatre reprises et de faire une visite pour l'injection.
- Je consens à participer à cette étude, sachant que je reste libre de m'en retirer à tout moment même après ma signature.
- J'ai bien noté que mon consentement ne décharge en rien le médecin et le promoteur de l'ensemble de leurs responsabilités et je conserve tous mes droits garantis par la loi.
- J'ai noté que toute information me concernant ne serait recueillie durant cette étude que dans le respect strict du secret professionnel.
- J'accepte que les données enregistrées à l'occasion de cette étude puissent faire l'objet d'un traitement informatisé par le promoteur ou pour son compte.
- J'ai noté que le fichier informatique utilisé pour réaliser la présente recherche a fait l'objet d'une autorisation de la CNIL en application des articles 40-1 et suivants de la loi "Informatique & Libertés". Ces informations pourront être contrôlées selon la réglementation en vigueur.
- J'ai bien noté que le droit d'accès prévu par la loi "Informatique et Libertés" (Art. 40) s'exerce à tout moment directement ou auprès de mon praticien.
- J'ai reçu l'information et copie du présent document, ai été informé qu'une copie serait également conservée par les organisateurs de la recherche dans des conditions garantissant la confidentialité et j'y consens.
- J'ai bien noté que je ne pourrai participer durant toute la durée de cette étude à un autre essai clinique et, que la période d'exclusion pour la participation à un autre essai s'élève à 3 mois après la fin de la présente étude. Je déclare n'avoir participé à aucun autre essai de recherche biomédicale depuis 3 mois.
- Je consens à l'utilisation par le médecin investigateur, à des fins licites, de tous les résultats et informations recueillis pendant cette étude à condition que mon anonymat demeure scrupuleusement respecté.
- En fait de quoi j'accorde mon consentement librement.

Date |__|__| |__|__| |__|__|__|__|
jour mois année

Signature du patient : Signature de l'investigateur :

Feuillet original blanc destiné au promoteur TRB CHEMEDICA, feuillet jaune destiné à SPRIT
Feuillet rose à conserver par l'investigateur, feuillet vert à remettre au patient

ANNEXE 2.9
CERTIFICAT D'ASSURANCE

PERSONAL INFORMATION

PERSONAL INFORMATION

ANNEXE 2.10
REFERENCES

REFERENCES

1. Reginster JY, Henrotin Y. Conception actuelle du traitement de l'arthrose. Revue de la Médecine Générale, 2000; 177: 382-391.

2. Creamer P, Hochberg MC. Osteoarthritis. Lancet 1997; 350: 503-9.

3. Altman RD. Pain relief in osteoarthritis: the rationale for combination therapy. J Rheumatol 2004; 31: 5-7.

4. Felson DT. The course of osteoarthritis and factors that affect it. Rheum Dis Clin North Am 1993; 19: 607-15.

5. Pouchot J, Coste J, Guillemin F. Impact of osteoarthritis on quality of life. In: Reginster JY, Pelletier JP, Martel-Pelletier J, Henrotin Y, Eds. Osteoarthritis Clinical and experimental aspects. Berlin Heidelberg: Springer 1999. p 331-55.

6. IRDES, Enquête Santé Protection Sociale. www.irdes.fr. 2006.

7. Jordan KM, Arden NK, Doherty M, Bannwarth B, Bijlsma JWJ, Dieppe P, et al. EULAR recommendations 2003: an evidence-based approach to the management of knee osteoarthritis: Report of a Task Force of the Standing Committee for International Clinical Studies Including Therapeutic Trials (ESCISIT). Ann Rheum Dis 2003: 62: 1145-55.

8. Maheu E. Principes thérapeutiques généraux des arthroses. Rev Prat (Paris) 1996; 46: 2218-24.

9.Zhang W, Nuki G, Moskowitz RW, Abramson S, Altman RD and al. OARSI recommendations for the management of hip and knee osteoarthritis: Part III: changes in evidence following systematic cumulative update of research published through January 2009. Osteoarthritis and Cartilage, 2010: 18 : 476-499.

10. Maheu E, Lamotte J, Lequesne M. Sel sodique de l'acide hyaluronique (hyaluronan) et gonarthrose. In : MF Kahn, D Kuntz, A Dryll, O Meyer, Th Bardin, Cl Guérin, eds, L'Actualité Rhumatologique 1994, Paris, Expansion Scientifique Française, 1994, p. 324-39.

11. Maheu E, Ayral X, Dougados M. A hyaluronan preparation (500-730 KDA) in the treatment of osteoarthritis : A review of clinical trials with Hyalgan®. Int J Clin Pract 2002; 56: 804-13.

12. Bellamy et al. Viscosupplementation for the treatment of osteoarthritis of the knee. Cochrane Database Syst Rev. 2006 April 19 (2): CD 005321.

13. Chen-Ti Wang, Jinn Lin, Chee-Jen Chang, Yu-Tsan Lin and Sheng-Mou Hou. Therapeutic effects of hyaluronic acid ou osteoarthritis of the knee. A meta-analysis of randomized controlled trials. J. Bone Joint Surg. Am. 86: 538-545, 2004.

14. Lo GH, LaValley M, McAlindon T, Felson DT. Intra-articular hyaluronic acid in treatment of knee osteoarthritis. A meta-analysis. Jama December 17, 2003 vol 290, N°23, 3115-3121.

15. Arrich J, Piribauer F, Mad P et al. Intra-articular hyaluronic acid for the treatment of osteoarthritis of the knee: systematic review and meta-analysis. CMAJ 2005 ; 172: 1039-43.

16. Chevalier X, Jerosch J, Goupille P, van Dijk N, Luyten FP, Scott DL, Bailleul F and Pavelka K. Single, intra-articular treatment with 6 ml hylan G-F 20 in patients with symptomatic primary osteoarthritis of the knee: a randomised, multicentre, double-blind, placebo controlled trial. Ann Rheum Dis, 2010; 69: 113-119.

17. Bellamy N, Buchan WW, Goldsmith CH, Campbell J, Stit LWJ. Validation of WOMAC: a health status instrument for measuring clinically important patient relevant outcomes to antirheumatic drug therapy in patients with osteoarthritis of the hip or knee. J Rheumatol 1995; 15: 1833-40

18. Altman RD, Asch E, Bloch D, Bole G, Borenstein D, Brandt K, et al - Development of criteria for the classification and reporting of osteoarthritis. Classification of osteoarthritis of the knee. *Arthritis Rheum* 1986;29:1039-49.

19. Kellgren JH, Lawrence DM - Radiological assessment of osteoarthritis. *Ann Rheum Dis* 1957;16:494-502.

20. Lequesne M, Méry C, Samson M, Gérard P - Indexes of severity for osteoarthritis of the hip and knee. *Scand J Rheumatol* 1987;65(Suppl):85-9.

21. Huskisson EC - Measurement of pain. *Lancet* 1974;II:1127-31.

22. Lellouche H, Bloch JG, Bellony R. Acide hyaluronique: Ponction et injection intra-articulaire dans la gonarthrose. Rhumatos septembre 2006 vol.3, N°20, 308-309.

23. Pham T, van der Heijde D, Altman RD, Anderson JJ, Bellamy N, Hochberg M, Simon L, Strand V, Woodworth T, Dougados M. OMERACT-OARSI Initiative: Osteoarthritis research society international set of responder criteria for osteoarthritis clinical trials revisited. Osteoarthritis cartilage 2004; 12: 389-399

24. Little, R.J.A. & Rubin, D.B. (1987). Statistical analysis with missing data. New York: Wiley.

25. http://www.ema.europa.eu/docs/en_GB/document_library/Scientific_guidelines /2009/09/wc500002928.pdf

26. Dictionnaire Vidal OCP éd, Paris 2011

27. Dictionnaire Vidal OCP éd, Paris 2010
