# Supplementary material for: A single intra-articular injection of 2.0% non-chemically modified sodium hyaluronate vs 0.8% hylan G-F 20 in the treatment of symptomatic knee osteoarthritis: A 6-month, multicenter, randomized, controlled non-inferiority trial
Source: PLoS One. 2019 Dec 10;14(12):e0226007. doi: 10.1371/journal.pone.0226007 (PMC6903764; doi:10.1371/journal.pone.0226007)
Supplement: S2 Protocol — (PDF) [file pone.0226007.s003.pdf]

S2 Protocol. Translated version of the study protocol (in English).

EFFICACY OF OSTENIL PLUS (HYALURONIC ACID) VERSUS SYNVISC-ONE IN PATIENTS WITH TIBIOFEMORAL OSTEOARTHRITIS

A RANDOMISED, CONTROLLED, DOUBLE-BLIND, PARALLEL-GROUP STUDY WITH A 6-MONTH FOLLOW-UP.

PROTOCOL

NATIONAL CO-ORDINATOR	Professor THOMAS BARDIN (APHP LARIBOISIERE)<br>2, RUE AMBROISE PARE<br>75010 PARIS<br>Tel. 01.49.95.62.90
SPONSOR AND HEAD OF PROJECT	TRB CHEMEDICA<br>Dr CHARLES JOLLES<br>IMMEUBLE ABC 1<br>SITE D'ARCHAMPS P.O. BOX 218<br>74162 ARCHAMPS CEDEX<br>Tel. 04.50.95.09.00 Fax 04.50.95.09.01
SCIENTIFIC ADVISOR	Dr RENEE LILIANE DREISER<br>APHP BICHAT-CLAUDE BERNARD<br>46, RUE HENRI HUCHARD<br>75018 PARIS<br>Tel. 01.42.80.95.73
SCIENTIFIC COMMITTEE	Dr RENEE LILIANE DREISER (APHP BICHAT-CLAUDE BERNARD)<br>Dr BERNARD AVOUAC (APHP HENRI MONDOR)
STUDY MANAGEMENT	SPRIT<br>7, RUE LALLIER<br>75009 PARIS<br>Tel. 01.48.78.87.78 Fax 01.48.78.87.82
STATISTICS	CEMKA<br>Dr ANTOINE LAFUMA<br>43, BOULEVARD DU MARECHAL JOFFRE<br>92340 BOURG LA REINE<br>Tel. 01.40.91.30.30 Fax 01.40.91.30.31

1. TITLE PAGE

**Title**	Efficacy of OSTENIL PLUS (hyaluronic acid) versus SYNVISC-ONE in patients with tibiofemoral osteoarthritis. A randomised, controlled, double-blind parallel-group study with a 6-month follow-up.
**Study location**	FRANCE
**Study aim**	To demonstrate the non-inferiority of the efficacy of OSTENIL PLUS compared with SYNVISC-ONE in symptomatic tibiofemoral osteoarthritis.
**Investigational plan**	Controlled, randomised study in parallel groups comparing two groups of patients receiving either 1 intra-articular injection of OSTENIL PLUS or 1 injection of SYNVISC-ONE.<br>Double-blind study: patient and evaluating investigator-blinded.
**Protocol**<br>**ID-RCB No.**	OSTP-EUR-10-01<br>2011-A00258-33
**Phase**	IV
**Sponsor**<br>**National co-ordinator**	TRB CHEMEDICA<br>Professor THOMAS BARDIN (APHP LARIBOISIERE)
**Start of trial**	May 2011
**End of trial**	June 2013
**Study management**	SPRIT
**Protocol date**	16 May 2011

2. SYNOPSIS

**Sponsor**: TRB CHEMEDICA	
**Name of investigational product**: OSTENIL PLUS	
**Name of active substance**: Sodium hyaluronate 2.0%	
**Study title**: Efficacy of OSTENIL PLUS (hyaluronic acid) versus SYNVISC-ONE in patients with tibiofemoral osteoarthritis. A randomised, controlled, double-blind parallel-group study with a 6-month follow-up.	
**Co-ordinator**: Professor THOMAS BARDIN (APHP LARIBOISIERE)	
**Number of investigators and sites**: 75 sites with two study investigators, one as evaluator and one as injector.	
**Planned study period**:<br>May 2011<br>End of June 2013	**Development phase of study**:<br>Phase IV
**Study aims**: To demonstrate the non-inferiority of the efficacy of OSTENIL PLUS compared with SYNVISC-ONE in symptomatic tibiofemoral osteoarthritis	
**Investigational plan**: Controlled, randomised study in parallel groups comparing two groups of patients receiving either 1 intra-articular injection of OSTENIL PLUS or 1 injection of SYNVISC-ONE. Double-blind study: patient and evaluator-blinded.	
**Number of patients**: Two groups of 130, i.e. a total of 260 patients.	
**Primary inclusion criteria**: Male or female patients aged 40 to 85 years, with unilateral or bilateral knee osteoarthritis according to the ACR criteria and radiologically confirmed (KELLGREN-LAWRENCE grades Ib to III) with pain assessed according to the mean WOMAC pain scale score (section A) of 40 mm or more.	
**Investigational product**: OSTENIL PLUS	
**Pharmacological form**: Viscoelastic solution for injection.	

**Sponsor**: TRB CHEMEDICA
**Name of investigational product**: OSTENIL PLUS
**Name of active substance**: Sodium hyaluronate 2.0%
**Route of administration**: Intra-articular
**Dosage**: One intra-articular injection
**Batch numbers**:
**Treatment duration**: 1 injection, 6 months' follow-up per patient, 5 consultations.
**Comparison product**: SYNVISC-ONE
**Pharmacological form**: Viscoelastic solution for injection
**Route of administration**: Intra-articular
**Dosage**: One intra-articular injection
**Batch numbers**:
**Efficacy endpoints**:<br>The primary endpoint is:<br>The course of the mean WOMAC pain scale score (section A) from D0 to D180<br>The secondary endpoints are:<br>The LEQUESNE index,<br>The course of the mean WOMAC stiffness and physical function scale score (section B and C),<br>Assessment of overall efficacy and safety by the patient and the investigator,<br>The patient's overall status score in relation to his knee osteoarthritis on a VAS,<br>Use of analgesics and NSAIDs,<br>The percentage of responder patients according to the OMERACT-OARSI criteria.<br><br>**Safety endpoints**:<br>The proportions of the subjects who present with one or more adverse events or with none will be studied.

PART 1: CONDUCT OF THE STUDY

3. SUMMARY

1. TITLE PAGE

2. SYNOPSIS

3. SUMMARY

4. LIST OF ABBREVIATIONS

5. REGULATORY AND ETHICAL ASPECTS

6. INVESTIGATORS, ORGANISATION AND STUDY PARTICIPANTS

7. INTRODUCTION

7.1. Importance of osteoarthritic disease
7.2. Management of osteoarthritis
7.3. Role of hyaluronic acid in the treatment of knee osteoarthritis
7.4. Hyaluronic acid in France
7.4.1. General aspects
7.4.2. OSTENIL PLUS
7.4.2.1. Clinical trials of OSTENIL PLUS
7.4.2.2. Marketing of OSTENIL PLUS
7.4.3. SYNVISC-ONE·
7.4.3.1. Clinical trials of SYNVISC-ONE·

8. OBJECTIVE
9. INVESTIGATIONAL PLAN
9.1. Experimental study design
9.2. Discussion of investigational plan
9.3. Study population
9.3.1. Selection criteria
9.3.1.1. Inclusion criteria
9.3.1.2. Non-inclusion criteria
9.3.2. Patient excluded during study and early withdrawal of treatment
9.4. Treatments
9.4.1. Products administered
9.4.1.1. Pharmaceutical form and dosage
9.4.2. Therapeutic regimen
9.4.2.1. Presentation and labelling
9.4.2.2. Assigning treatments
9.4.2.3. Blinding
9.4.2.4. Storage and handling of treatments
9.4.3. Choice of study dose
9.4.4. Prior and concomitant treatments
9.4.4.1. Prior treatments

9.4.4.2. Concomitant treatments
9.4.4.3. Forbidden treatments
9.4.5. Patient compliance
9.5. Evaluation of efficacy and safety
9.5.1. Endpoints
9.5.1.1. Primary endpoint
9.5.1.1.1 Efficacy
9.5.1.1.2 Safety
9.5.1.2. Secondary endpoints
9.5.1.2.1 Efficacy
9.5.1.2.2 Safety
9.5.2. Relevance of the endpoints
9.5.2.1. Primary endpoint for efficacy
9.5.3. Endpoints for clinical safety
9.5.3.1. Adverse events
9.5.3.2. Safety study
9.6. Final report
9.7. Planned statistical methods and calculation of the number of subjects
9.7.1. Chosen population size
9.7.2. Parameters used to calculate the number of subjects
9.7.3. Statistical evaluation
9.7.3.1. Statistical analysis method
9.7.3.1.1 Blind review
9.7.3.1.2 Sample description
9.7.3.1.3 Comparison of groups before treatment
9.7.3.2. Endpoints
9.7.3.2.1 Primary endpoint for efficacy
9.7.3.2.2 Secondary criteria
9.7.3.2.3 Endpoint for safety
9.7.3.3. Statistical analyses
9.7.3.3.1 Definition of populations analysed
9.7.3.3.1.1 Intent-to-treat population (ITT)
9.7.3.3.1.2 Per Protocol population (PP)

10. CALENDAR

PART II: GENERAL PRINCIPLES

1. ETHICS
1.1 Declaration of Helsinki
1.2 Good Clinical Practice

2. INVESTIGATORS' RESPONSIBILITIES
2.1 Ethics
2.1.1 Declaration of Helsinki
2.1.2 CPP (Ethics Committee)
2.1.3 Informing the patient
2.1.4 Patient informed consent
2.1.5 Withdrawal of a subject
2.1.6 Patient anonymity
2.1.7 Fees
2.2 Amendments to the protocol
2.3 Case report form
2.4 Checking source documents
2.5 Adverse events
2.5.1 Definition
2.5.1.1 Serious adverse events
2.5.1.2 Serious adverse event report
2.5.1.3 Death
2.5.1.4 Dropouts
2.5.1.5 Non-serious adverse events
2.5.1.6 Treatment and monitoring of adverse events
2.6 Publications
2.7 Archiving
2.8 Audit

3. RESPONSIBILITIES OF THE SPONSOR AND SERVICE PROVIDERS
3.1 General responsibilities
3.2 Insurance
3.3 Monitoring and responsibilities of service providers
3.4 Quality assurance and quality control
3.5 Archives

4. SIGNATURES

PART III: ANNEXES

1 ANNEXES RELATED TO THE STUDY

1. ACR criteria for knee osteoarthritis
2. Kellgren-Lawrence grading scale
3. Lequesne algofunctional index for knee osteoarthritis
4. WOMAC – Western Ontario McMaster's Arthritis Index
5. OMERACT-OARSI criteria
6. List of NSAID treatments
7. NSAID wash-out periods
8. Visual analogue scale (VAS)
9. Analysis certificate
10. Co-ordinator's curriculum vitae
11. Investigators participating in the trial
12. Case report forms
13. Patient form
14. Information sheet OSTENIL PLUS & SYNVISC-ONE
15. Pharmacovigilance form and serious adverse event (SAE) report
16. Acknowledgement of receipt
17. Confidentiality undertaking and protocol approval

2. ANNEXES RELATED TO LEGAL OBLIGATIONS

18. Declaration of Helsinki
19. Huriet law
20. Implementing circular on DMOS (*Diverses Mesures d'Ordre Social* – French Anti-Gift Law or French DMOS Law)
21. Good clinical practice
22. Opinion of the CPP (Ethics Committee)
23. Opinion of the AFSSAPS (French health products safety agency)
24. Patient information leaflet
25. Patient informed consent
26. Insurance certificate
27. References

4. LIST OF ABBREVIATIONS

ACR	American College of Rheumatology
AFSSAPS	French Agency for the Safety of Health Products
HA	Hyaluronic acid
NSAID	Non-steroidal anti-inflammatory drug
ANOVA	Analysis of variance
CRA	Clinical Research Associate
SYSADOA	Symptomatic slow-acting drugs for osteoarthritis
GCP	Good Clinical Practice
C	Consultation
CPP	Ethics Committee
CM	Centimetre
CNIL	National Commission for Data Processing and Civil Liberties
CRF	Case report form
CRO	Contract Research Organisation
CV	Curriculum vitae
MD	Missing data
AE	Adverse event
SAE	Serious adverse event
SE	Side effect
EULAR	European League Against Rheumatism
VAS	Visual analogue scale
F	Female
G	Gram
M	Male
h	hour
IA	Intra-articular
ICH	International Conference on Harmonisation
ITT	Intent to treat
D	Day
KG	Kilogram
LOCF	Last observation carried forward
LPPR	List of reimbursable products and services
M	Month
MAR	Missing at random
MCAR	Missing completely at random
MedDRA	Medical Dictionary for Regulatory Activities
MG	Milligram
MW	Molecular weight
PP	Per Protocol
SD	Standard deviation
WOMAC	Western Ontario McMaster’s Arthritis Index

5. REGULATORY AND ETHICAL ASPECTS

See PART II

6. INVESTIGATORS, ORGANISATION AND STUDY PARTICIPANTS

**NATIONAL CO-ORDINATOR**	Professor THOMAS BARDIN (APHP LARIBOISIERE)<br>2, RUE AMBROISE PARE<br>75010 PARIS<br>Tel. 01.49.95.62.90
**SPONSOR AND HEAD OF PROJECT**	TRB CHEMEDICA<br>Dr CHARLES JOLLES<br>IMMEUBLE ABC 1<br>SITE D'ARCHAMPS P.O. BOX 218<br>74162 ARCHAMPS CEDEX<br>Tel. 04.50.95.09.00 Fax 04.50.95.09.01
**SCIENTIFIC ADVISOR**	Dr RENEE LILIANE DREISER<br>APHP BICHAT-CLAUDE BERNARD<br>46, RUE HENRI HUCHARD<br>75018 PARIS<br>Tel. 01.42.80.95.73
**SCIENTIFIC COMMITTEE**	Dr RENEE LILIANE DREISER (APHP BICHAT-CLAUDE BERNARD)<br>Dr BERNARD AVOUAC (APHP HENRI MONDOR)
**STUDY MANAGEMENT**	SPRIT<br>7, RUE LALLIER<br>75009 PARIS<br>Tel. 01.48.78.87.78 Fax 01.48.78.87.82
**INVESTIGATORS**	SEE ANNEX
**STATISTICS**	CEMKA<br>Dr ANTOINE LAFUMA<br>43, BOULEVARD DU MARECHAL JOFFRE<br>92340 BOURG LA REINE<br>Tel. 01.40.91.30.30 Fax 01.40.91.30.31
**CLINICAL REPORT**	Professor THOMAS BARDIN (APHP LARIBOISIERE)<br>2, RUE AMBROISE PARE<br>75010 PARIS<br>Tel. 01.49.95.62.90

7. INTRODUCTION

7.1 IMPORTANCE OF OSTEOARTHRITIC DISEASE

Osteoarthritis is the most widespread joint disease in the population. It affects more than half of all those over the age of 65 and 80% of those over 75 (1). Women and those over the age of 45 are more frequently affected. The prevalence of osteoarthritis is expected to increase considerably due to the increase in mean life expectancy, the ageing population and continued involvement in occupational and sports activities to an advanced age (2). It is therefore a major public health problem and, according to ALTMAN, its economic impact in the USA "exceeds the gross national product of several countries" (3).

The main aetiological factors or risk factors for knee osteoarthritis are age, family history, obesity, knee injury (sprains, ruptured ligaments, meniscopathy), meniscectomy and some arthrogenic occupational or sports activities. There is a close correlation between obesity and knee osteoarthritis (4).

Symptomatic knee osteoarthritis is a chronic incapacitating disorder responsible for a considerable degree of disability. It has a high impact on quality of life (5).

In 2006 the number of knee osteoarthritis patients in France was estimated at between 1.8 and 2.3 million (6).

The main symptoms of osteoarthritis are pain, the resulting functional impairment and joint stiffness. Most current treatments aim to improve these symptoms, in particular pain. They succeed only partially (4) and do not prevent a number of people needing surgery (generally prosthetic) to improve their condition. The number of prosthetic knees fitted annually in France is estimated to be 50,000 which also represents a very high social cost.

None of the medical treatments currently available on the market can halt the progression of osteoarthritis or permanently reduce its symptoms reliably.

7.2 MANAGEMENT OF OSTEOARTHRITIS

All the recent recommendations stress the importance, both medically and financially, of the early therapeutic management of knee osteoarthritis to delay and even limit the use of aggressive treatments and particularly surgery.

A study group consisting of delegates from thirteen European countries under the auspices of the European League Against Rheumatism (EULAR) began to draw up its recommendations for the treatment of knee osteoarthritis in September 1998.

These recommendations were published in late 2000 and revised in 2003 (7). The methodology was devised in co-operation with the Department of Public Health at St Georges Hospital Medical School in London, to meet the criteria of evidence-based medicine, but many of the therapeutic measures in the field of osteoarthritis have not yet been scientifically studied. A joint decision-making process in several rounds (Delphi method) among the 24 European experts also examined 23 methods of treating knee osteoarthritis and 10 recommendations were adopted and published.

The treatment should be adjusted to each patient (7,8). Symptomatic slow-acting drugs for osteoarthritis (SYSADOA) can be used in patients whose symptoms are poorly controlled. Recommendation No. 8 concerns hyaluronic acid which is one of the recommended symptomatic slow-acting drugs for osteoarthritis.

More recently in 2010 the OARSI drew up 25 recommendations for the methods of managing osteoarthritis (9). Intra-articular injections of hyaluronic acid continue to appear in these recommendations with an effect size judged to be moderate (0.60).

7.3 ROLE OF HA IN THE TREATMENT OF KNEE OSTEOARTHRITIS

Hyaluronic acid (HA) is a macropolysaccharide produced by the polymerisation of thousands of basic disaccharide units composed of D-N-acetylglucosamine and D-glucuronic acid. Its molecular weight in the normal state in cartilage or synovial fluid is approximately 4 to 5 million daltons (Da).

The observations that have led to the use of hyaluronic acid in the treatment of knee osteoarthritis are as follows:

In osteoarthritis the hyaluronic acid concentration in synovial fluid is greatly reduced and viscosity is lower as a result. The increase in low molecular weight hyaluronic acid also contributes to the reduction in viscosity. This deterioration in rheological properties causes severe impairment of the intercellular matrix and cell functions. The disappearance of the lubricant and protective effects of hyaluronic acid allows shear forces, caused by the movement of the joints, to destroy the joint cartilage by friction.

Hyaluronic acid or hyaluronan (the international name now recommended) was trialled in the treatment of knee osteoarthritis in the early 1980s (10). More than sixty clinical trials have been published since then, with different hyaluronans, versus placebo, versus intra-articular corticosteroids and versus NSAIDs. Most of these therapeutic trials were conducted in knee osteoarthritis and provide evidence of the symptomatic clinical efficacy of hyaluronic acids in its treatment (10,11). A meta-analysis concluded that hyaluronic acids were effective in the treatment of knee osteoarthritis (beneficial effects as regards pain, function and the patient's overall assessment) (12). However some meta-analyses (13,14,15) report moderate efficacy of hyaluronic acids and this is why the AFSSAPS has requested new trials in this disorder.

7.4 HA IN FRANCE

7.4.1 General aspects

In France there are fifteen or so different hyaluronic acid products for the treatment of knee osteoarthritis, registered as medical devices, one of which has medicinal product marketing authorisation (MA).
They differ in their method of extraction (rooster comb or biofermentation), their crosslinked structure with a molecular weight of more than 6 million daltons (SYNVISC, DUROLANE) or their non-crosslinked structure (ADANT, SINOVIAL, ARTHRUM H) with a molecular weight ranging from 600,000 to 1.2 million daltons, their concentration (0.8 to 2%) and finally their unit dose (2 to 3 ml).

Intra-articular injections of hyaluronic acid have been reimbursed to 65% in France since 1999 in the treatment of knee osteoarthritis, provided they are given by a rheumatologist, a physical rehabilitation physician or an orthopaedic specialist. For almost all the products treatment consists of three weekly injections. The treatment is indicated and reimbursable only in symptomatic knee osteoarthritis, and after failure or intolerance of first-line analgesics and NSAIDs, at the rate of one treatment of 3 injections per knee and per year at most. However two products are used with a single injection only (DUROLANE and SYNVISC-ONE™.).

7.4.2 OSTENIL PLUS

OSTENIL PLUS belongs to the non-crosslinked hyaluronic acids, obtained by bacterial fermentation and free from animal proteins.
OSTENIL PLUS is a transparent viscoelastic solution of natural hyaluronic acid highly purified to 2% sodium hyaluronate, the sodium salt of hyaluronic acid, reserved strictly for intra-articular injections.
OSTENIL PLUS also contains mannitol, a free radical scavenger, which helps to stabilise hyaluronic acid chains.
The mean molecular weight is between 1.5 and 2 million daltons.

7.4.2.1 Clinical trials of OSTENIL PLUS

In an open, uncontrolled, multicentre pilot study conducted in Spain, 80 patients with osteoarthritis of the knee received one injection of OSTENIL PLUS (D0) and were monitored for 6 months, with evaluations at 0, 15, 30, 60, 90, 120,150 and 180 days.
A clinical evaluation of pain and joint function was carried out using a visual analogue scale (VAS) and the WOMAC index.
A significant reduction in joint pain, stiffness and functional impairment compared with baseline was observed at each follow-up visit ($p < 0.001$) from the 15th day onwards.
Joint pain was improved by 40.7% (VAS) and 38.7% (WOMAC) on D30, reaching 46.5% and 47.5% respectively on D180.
The evaluation of efficacy and safety by the investigator and the patient were considered excellent throughout the study.
Mild side effects (local pain and swelling at the injection site) were reported in 4 patients on D15.
No serious adverse effects were observed.

7.4.2.2 Marketing of OSTENIL PLUS

OSTENIL PLUS was first marketed in Germany in February 2009.
Since then the product has been marketed in several European countries (Belgium, Bulgaria, Spain, France, Greece, Hungary, Luxembourg, Netherlands, Romania and the UK) and in Switzerland.
By the end of December 2010 nearly 87,900 syringes of OSTENIL PLUS had been sold worldwide.
No incidents had been reported to the competent authorities.

7.4.3 SYNVISC-ONE

SYNVISC-ONE contains a type of hylan polymer technically known as hylan G-F20, a sterile, non-pyrogenic viscoelastic fluid.
hylans are derivatives of hyaluronan (sodium hyaluronate) and consist of repeating disaccharide units of N-acetylglucosamine and sodium glucuronate.
Hylan G-F20 contains hylan A and hylan B (8.0 mg ± 2.0 mg per ml).
Hylan A has a mean molecular weight of 6 million daltons and hylan B is a hydrated gel.

7.4.3.1 Clinical trials of SYNVISC-ONE

SYNVISC-ONE has recently been the subject of a comparative double-blind placebo-controlled trial (injector and evaluator-blinded). This randomised study was carried out on two parallel groups of 253 patients with painful knee osteoarthritis treated with one injection of SYNVISC-ONE or placebo by randomisation, then monitored for 26 weeks.
The results demonstrated the superiority of SYNVISC-ONE one single injection of Hylan G-F20 of 6 ml over placebo as regards the primary endpoint; WOMAC pain (-0.15 (SE), p=0.047) and also the secondary endpoints (16).
In this trial the safety of SYNVISC-ONE was satisfactory and not statistically different from that of the placebo.
No serious adverse effects were reported.

To conclude the majority of the hyaluronic acid products currently available are 1.0% solutions and require 3 to 5 injections to restore the viscoelastic properties of the synovial fluid in osteoarthritis of the knee.

A higher concentration (2% hyaluronic acid) should allow a more rapid, more sustained improvement in the viscoelastic properties of synovial fluid.

To confirm the pilot study results it was considered appropriate to conduct a new trial in a larger number of patients with OSTENIL PLUS in comparison with a reference product, SYNVISC-ONE·.

8. OBJECTIVE

This is a non-inferiority study of the efficacy of OSTENIL PLUS compared with SYNVISC-ONE in symptomatic tibiofemoral osteoarthritis.
The primary endpoint is the course of the mean WOMAC pain scale score (section A) (17) (Annex 1.4) from D0 to D180 in patients receiving treatment with one intra-articular injection of OSTENIL PLUS versus one intra-articular injection of SYNVISC-ONE·
Secondly this will enable data on the safety of the two treatments to be obtained.

9. INVESTIGATIONAL PLAN

9.1 Experimental study design

Phase IV, prospective, randomised, controlled, double-blind, multicentre study of two parallel groups comparing the administration of one intra-articular injection of OSTENIL PLUS with the administration of one intra-articular injection of SYNVISC-ONE in patients with symptomatic knee osteoarthritis.
260 patients are planned in total.

The study will take place in all the regions of France.
It is planned to recruit 75 evaluating investigators and 75 injecting investigators who will study 4 to 8 cases per centre.
The recruitment period will be eighteen months after the first appointment. The patients will be treated once and monitored for six months.
The treatment will be a single injection of hyaluronic acid.
5 visits are planned per patient.

C 0 (D-7) Pre-selection visit by evaluating investigator
At the end of the visit, if the patient is taking NSAIDs, he will undergo a wash-out of 2 to 5 days with a minimum of 2 days (Annex 1.7).
Paracetamol can be used subject to a wash-out of at least 24 hours before C 1.
If a wash-out is unnecessary C 0 and C1 can be combined.
The investigator will make sure that the patient has X-ray less than 1 year old.

C 1 (D 0) Visit for inclusion by evaluating investigator
Evaluation of patient as regards pain.
The patient must have a mean pain of at least 40 mm (VAS scored from 0 to 100 mm) on the WOMAC pain scales (section A) (Annex 1.4) for the knee to be injected, with a difference of at least 20 mm from the pain in the opposite knee if knee osteoarthritis is bilateral. For bilateral osteoarthritis the most painful knee will be used.
Demographic data: age, height, weight, BMI, general health.
Knee osteoarthritis characteristics.
ACR criteria (18) (Annex 1.1)
Radiological criteria (19) (Annex 1.2)
Descriptive data: history, prior and concomitant therapy.
Check on inclusion and non-inclusion criteria.
LEQUESNE algofunctional index (20) (Annex 1.3).
Patient's overall status score in relation to his knee osteoarthritis on VAS (21) (Annex 1.8).
Patient's signed informed consent.
Declaration of inclusion.

The patient will then be referred to an "injecting" rheumatologist or a physical medicine specialist or an orthopaedic surgeon to administer the product.

After this visit and throughout the study the patient will record in a diary any analgesics used (paracetamol, number of tablets and dose).
Any treatment with paracetamol should be stopped at least 12 hours before each visit (preferably 24 hours).

C 2 (D 2) Injection visit (± 2 days) by injecting investigator
Use of paracetamol (check patient diary).
Description of adverse events.
Changes in current treatments.
Patient's overall status score in relation to his knee osteoarthritis on VAS.
Injection of the trial product.
Confirmation of injection.

C 3 (D 30) Follow-up visit (± 15 days) by evaluating investigator
Use of paracetamol (check patient diary).
Changes in current treatments.
Assessment of the overall treatment efficacy by patient and investigator.
Assessment of the overall treatment tolerance by patient and investigator.
Assessment of the local treatment tolerance by patient and investigator.
Description of adverse events.
Total WOMAC (Annex 1.4).
LEQUESNE algofunctional index (Annex 1.3).
Patient's overall status score in relation to his knee osteoarthritis on VAS (Annex 1.8).

C 4 (D 90) Follow-up visit (± 15 days) by evaluating investigator
Use of paracetamol (check patient diary).
Changes in current treatments.
Assessment of the overall treatment efficacy by patient and investigator.
Assessment of the overall treatment tolerance by patient and investigator.
Description of adverse events.
Total WOMAC (Annex 1.4).
LEQUESNE algofunctional index (Annex 1.3).
Patient's overall status score in relation to his knee osteoarthritis on VAS.

C 5 (D 180) End of trial visit (± 15 days) by evaluating investigator
Use of paracetamol (check patient diary).
Changes in current treatments.
Assessment of the overall treatment efficacy by patient and investigator.
Assessment of the overall treatment tolerance by patient and investigator.
Description of adverse events.
Total WOMAC (Annex 1.4).
LEQUESNE algofunctional index (Annex 1.3).
Patient’s overall status score in relation to his knee osteoarthritis on VAS (Annex 1.8).
End of trial sheet.

In the event of early termination of the study or a missed intermediate consultation, a final visit must be carried out and the reasons for termination must be described in detail.

STUDY OUTLINE

	D 0<br>C 1	D 2<br>C 2<br>IA	D 30<br>C 3<br>Follow-up	D 90<br>C 4<br>Follow-up	D 180<br>C 5<br>End
Demographic data	X				
Surgical and medical history	X				
Current treatments	X				
Features of knee osteoarthritis	X				
ACR criteria	X				
ALTMAN classification	X				
Inclusion criteria	X				
Non-inclusion criteria	X				
OSTENIL PLUS or SYNVISC-ONE injection group		X			
Informed consent obtained	X				
Declaration of inclusion	X				
**Assessment of efficacy**					
WOMAC total	X		X	X	X
LEQUESNE	X		X	X	X
Patient's overall status score VAS	X	X	X	X	X
Investigator's overall assessment			X	X	X
Patient's overall assessment			X	X	X
**Assessment of compliance**					
Use of paracetamol		X	X	X	X
Concomitant treatments		X	X	X	X
**Assessment of safety**					
Investigator's overall assessment			X	X	X
Patient's overall assessment			X	X	X
Investigator's local assessment			X		
Patient's local assessment			X		
Adverse events		X	X	X	X

9.2 Discussion of investigational plan

The study objective is to demonstrate the non-inferiority of the efficacy of OSTENIL PLUS compared with SYNVISC-ONE in the treatment of symptomatic tibiofemoral osteoarthritis.

9.3 Study population

9.3.1 Selection criteria
9.3.1.1 Inclusion criteria

- Male or female patient aged 40 to 85 years.
- Patient with primary knee osteoarthritis meeting ACR criteria (Annex 1.1):
  Mechanical knee pain
  and at least one of the following 3 criteria:
  1. Age > 50 years
  2. Morning stiffness < 30 minutes
  3. Popping in the knee on active movement
  and Osteophytes on anteroposterior X-ray of both knees.
- Patient whose osteoarthritis has been defined radiologically: narrowed joint spaces and osteophyte on images taken less than 1 year previously and modified KELLGREN-LAWRENCE grades Ib to III (19) (Annex 1.2) (i.e. narrowed joint spaces of between 25 and 75% and confirmed osteophyte).
- Patient with symptoms, on one side only, with a mean score on the WOMAC pain scale (section A) of ≥ 40 mm, or for bilateral knee osteoarthritis, with a difference of at least 20 mm in the same score between the opposite knee and the studied knee (after wash-out for analgesics or NSAIDs) (Annex 1.7).
- Patient with pain present at least 15 days in the month before inclusion.
- Patient needing treatment with a hyaluronic acid injection after failure or intolerance of first-line analgesics and NSAIDs.
- Patient with health insurance.
- Patient able to understand and follow the study instructions.
- Patient who has signed the informed consent.

9.3.1.2 Non-inclusion criteria

In addition to patients who do not meet all the inclusion criteria mentioned above, patients presenting with at least one of the following criteria will be excluded:

- Non-symptomatic or insufficiently symptomatic knee osteoarthritis.
- Symptomatic bilateral knee osteoarthritis with the same severity on both sides.
- Secondary post-traumatic knee osteoarthritis.
- Radiological grade I, Ia or IV knee osteoarthritis.
- Knee osteoarthritis that is exclusively patellofemoral or with symptoms mainly of patellofemoral origin (patellar syndrome).
- Patient with symptomatic homolateral hip osteoarthritis.
- Varus or valgus deformity of the selected knee (deformity axis ≥ 15° on X-ray).
- Major hydrarthrosis (requiring puncture) at the time of inclusion.
- Inflammatory rheumatism (RA, psoriatic arthritis, articular chondrocalcinosis, gout, Paget’s disease, ankylosing spondylitis, lupus, etc.).
- History of injury to the selected knee in the six months before inclusion.
- Wound or skin disorder of the selected knee.
- Venous or lymphatic stenosis of the lower limb.
- Femoral or sciatic nerve root pain of the lower limb under study.
- Tendinopathy (hip periarthritis).
- Subject who has been treated with intra-articular hyaluronic acid in the selected knee in the 6 months before inclusion.
- Subject who has received an intra-articular corticosteroid injection in the selected knee in the 2 months before inclusion.
- Subject treated with a symptomatic slow-acting drugs for osteoarthritis and/or dietary supplements for osteoarthritis (chondroitin sulphate, diacerein, avocado and soybean unsaponifiables, oxaceprol, copper granions, glucosamine) started in the last 3 months, or the dose of which has been changed in the last 3 months, before inclusion.
- Anticoagulant treatment with heparin or warfarin, however antiplatelet agents such as ASPIRIN ≤ 325 mg/day, ticlopidine (TICLID) or clopidogrel (PLAVIX) are possible.
- Insertion of a total prosthesis in the selected knee.
- Surgery of the other knee or hip or any other surgery scheduled during the trial.
- History of any surgical procedure, arthroscopy, osteotomy, etc. in the year before inclusion.
- Patient who is obese (BMI ≥ 30 kg/m^2).
- Subject with a history of autoimmune diseases.
- Subject with known hypersensitivity to hyaluronic acid and/or avian proteins and/or paracetamol.
- Subject with known hypersensitivity to mannitol.
- Patient with a severe disorder likely to interfere with evaluation, such as: cancer, malignant blood disorder, kidney disease, liver disease or severe infection.
- Subject having taken part in a clinical research study in the previous 3 months.
- Woman who is pregnant, or likely to become pregnant during the trial, or breast-feeding.

9.3.2 Patient excluded during study and early withdrawal of treatment
The evaluating and/or injecting-investigator can decide at any time, for each patient, not to continue the trial. Similarly each patient can withdraw consent (Annex 2.8) and leave the study at any time.
Reasons for leaving the trial can be:
- Symptomatic (lack of efficacy, decision to have surgery on the target knee, prosthesis or other reason, etc.);
- Therapeutic (adverse event(s), etc.);
- Medical (intercurrent disorder incompatible with the continuation of the trial, etc.);
- Personal (availability, lack of motivation, moving house, etc.).

Dropouts from the study will be recorded and documented.

In all cases a final clinical evaluation must be carried out as close as possible to the date the subject dropped out.

Early dropouts will be counted, identified and described per visit and the percentage in the two treatment groups will be compared using a chi^2 test.

Subjects who drop out before the injection will be replaced and not included in the intent-to-treat analysis.

Other subjects who drop out early from the study will not be replaced. The last value, known as the last observation carried forward (LOCF), will be taken into account at subsequent visits not made.

Dropouts due to serious adverse events (SAE) will be the subject of a special notification.

9.4 Treatments

9.4.1 Products administered
Batches of treatment for the study will be packaged by TRB CHEMEDICA.
The treatments allocated will be the following: OSTENIL PLUS or Synvisc-One after randomisation.

9.4.1.1 Pharmaceutical form and dosage
Characteristics of OSTENIL PLUS
Brand name: OSTENIL PLUS
Form and presentation: Viscoelastic solution for injection into the joint cavity: Prefilled syringe of 40 mg/2 ml, in a sterile pack, unit box. Steam sterilised.
Composition: Isotonic solution (pH = 7.3): per ml
Sodium hyaluronate of fermentation origin 20 mg
Excipients: sodium chloride, monosodium phosphate, disodium phosphate, mannitol, WFI.
Manufacturer: TRB CHEMEDICA
Dosage: Inject 1 syringe of OSTENIL PLUS into the affected joint.
Route of administration: intra-articular
Storage conditions: Store at room temperature not exceeding 25ºC.

Characteristics of SYNVISC-ONE
Brand name: SYNVISC-ONE
Form and presentation: 6 ml: glass syringe of 10 ml
Composition per syringe
Per 1 ml (hylan G-F20): hylan 8.0 mg, sodium chloride 8.5 mg, disodium phosphate 0.16 mg, monosodium phosphate hydrate 0.04 mg, WFI qs
SYNVISC-ONE contains 6 ml of hylan G-F20, hyaluronic acid extracted from rooster comb and crosslinked.
Manufacturer: GENZYME
Dosage: knee osteoarthritis, one injection
Route of administration: exclusively intra-articular
Storage conditions: temperature between 2ºC and 30ºC.

9.4.2 Therapeutic regimen

9.4.2.1 Presentation and labelling
Each therapeutic unit is identified by a number (from 001 to 400) and labelled according to current regulations.
The treatments OSTENIL PLUS and SYNVISC-ONE will be packaged in an identical neutral pack.

9.4.2.2 Assigning treatments
The numbered treatment batches will be allocated to each investigator after a randomisation procedure.
Once the final list of investigators has been closed, one of the investigators on the list will be randomised to receive treatment batches No. 001 to No. 004.
The investigator next on the list will receive the following batches, i.e. No. 005 to No. 008 and so on to the last investigator.
The necessary treatments will be given to the injecting-investigators when the trial is set up.
Each injecting-investigator will receive 4 to 8 batches.
The treatment batches will be used in increasing numerical order.

9.4.2.3 Blinding
The randomisation list will be drawn up by the study sponsor.
The patients will be randomised to one of the two treatment groups, OSTENIL PLUS or SYNVISC-ONE according to a computerised randomisation procedure in blocks of four treatments.
The treatments will be packaged in an identical neutral pack.
To preserve the balance between the groups each investigator will undertake to recruit 4 patients.
Each evaluating-investigator will include patients in order of randomisation beginning with the lowest number for the first patient and so on.
Each injecting investigator will perform one injection of the product according to the same treatment number beginning with the lowest number for the first patient, checking that it corresponds to the number given by the evaluating investigator.
The procedure will be the same for the subsequent patients in increasing numerical order.
Each investigator will receive a "tamper-evident" envelope for each batch, marked with the patient number.
Each envelope will contain the patient number and the group the patient belongs to.
If necessary the investigator can, with justification, open the appropriate envelope. All the randomisation envelopes will be collected at the end of the trial.
As the viscosity, volume and size of syringe of OSTENIL PLUS are different from those of the SYNVISC-ONE solution and thus easily identifiable, the double-blind procedure will also require the investigator assessing the patients clinically to be different from the injecting investigator, so that neither the patient nor the evaluating investigator know which product has been administered.

9.4.2.4 Storage and handling of treatments
The treatments should be stored in a place clearly defined by the investigator to ensure their correct storage and inaccessibility to third parties.
The treatments must be used only for this study.
Throughout the trial the Clinical Research Associate of the company in charge of monitoring may have access to the treatments for checking purposes.
At the end of the trial all used or unused treatments will be returned to the Clinical Research Associate of the company in charge of monitoring.

9.4.3 Choice of study dose
That recommended namely 1 injection.

The intra-articular injection of OSTENIL PLUS or of SYNVISC-ONE solution according to the randomisation group will be carried out in the injecting investigator's surgery, according to the usual rules of asepsis for intra-articular injections, as stated in the GRRIF (French Interventional Rheumatology Research Group) consensus:
"The physician does not need to routinely wear gloves and a mask unless he has an ENT infection;
It is essential to wash hands and nails carefully and meticulously with an antiseptic solution;
The patient's skin is cleaned but not shaved (clipped or depilated with cream if necessary), disinfected with sterile pads soaked in an iodine derivative such as betadine alcohol which should be allowed to dry for 3 minutes on average;
For patients who are allergic to iodine, a different antiseptic should be used, but not quaternary ammonium compounds which can interact with hyaluronic acid (HA).
After injection a clean or even sterile dressing is applied" (22).

The injection should preferably be given externally, after clinically identifying the injection site.

9.4.4 Prior and concomitant treatments
9.4.4.1 Prior treatments
Any treatment taken by the patient on inclusion in the trial will be recorded in the CRF at the initial visit.
If the patient is not taking any NSAIDs or analgesics he can be included in the study subject to meeting the other inclusion and non-inclusion criteria.
If the patient is on NSAIDs and/or analgesics a wash-out will be necessary (Annex 1.7).
For oxicam derivatives the wash-out will be 10 to 15 days.
For all other anti-inflammatories it will be 4 days.
For treatment with paracetamol a 24-hour wash-out should be observed before inclusion.

9.4.4.2 Concomitant treatments
Treatments prescribed during the trial for disorders other than knee osteoarthritis will be authorised if necessary.
They should be described and recorded in the CRF at follow-up visits.

The following are authorised in particular:
Short-term courses of NSAIDs (3 to 5 days per month), these should be withdrawn at least 5 half-lives before evaluation visits (Annex 1.7), preference should be given to NSAIDs with a short half-life;
Topical NSAIDs (wash-out 2 days);
Oral corticosteroids (wash-out 2 days);
Antiplatelet agents: ASPIRIN (with a daily maximum of 325 mg), ticlopidine (TICLID) and clopidogrel (PLAVIX);
Physiotherapy treatments (ultrasound, electrotherapy, ionisation);
Alternative medicine (acupuncture, homeotherapy, mesotherapy);
Mineral water treatments;
Kinesitherapy.

The following intercurrent treatments will not be permitted at any time during the trial:
symptomatic slow-acting drugs for osteoarthritis and/or dietary supplements for osteoarthritis (chondroitin sulphate, diacerein, avocado and soybean unsaponifiables, oxaceprol, copper granions, glucosamine);
Intra-articular injections of hyaluronic acid;
Intra-articular injections of corticosteroids;
Any treatment which, in the investigator's opinion, could interfere with the efficacy of the test treatment or with the interpretation of the endpoints.
If there is any doubt about whether a treatment can be taken, the scientific committee should be consulted.

9.4.4.3 Forbidden treatments
The following are forbidden for the treatment of knee osteoarthritis up to the end of study visit at 6 months:
Symptomatic slow-acting drugs for osteoarthritis and/or dietary supplements for osteoarthritis (chondroitin sulphate, diacerein, avocado and soybean unsaponifiables, oxaceprol, copper granions, glucosamine), unless they have been used regularly for at least 3 months and their doses remain constant and they must be continued until the end of the trial;
NSAIDs including topical forms;
Intra-articular injections of hyaluronic acid or corticosteroid;
Oral corticosteroids;
Local treatments: intra-articular lavage, puncture (except if required for severe hydrarthrosis);
Physiotherapy treatments (however if these have been followed for at least 1 year and are regular they can be continued);
Alternative medicine.

If necessary paracetamol can be taken up to 4 g/day and this should be noted in the patient diary and recorded in the CRF at each visit (it will be used as an additional endpoint). A wash-out of 12 hours will be observed before the follow-up visits.

If any Forbidden treatment is taken the patient will be withdrawn from the trial.

9.4.5 Patient compliance
This will be assessed by the injection being given.

9.5 Evaluation of efficacy and safety

9.5.1 Endpoints
9.5.1.1 Primary endpoint
9.5.1.1.1 Efficacy
The primary endpoint is the change in the mean WOMAC pain scale score (section A) from D0 to D180 (Annex 1.4).

9.5.1.1.2 Safety
The proportions of subjects who present with one or more adverse events or with none will be studied during the trial.

9.5.1.2 Secondary endpoints
9.5.1.2.1 Efficacy
The secondary endpoints for efficacy will be the clinical criteria and their course during the 6-month follow-up:

- Mean WOMAC stiffness and physical function scale scores (section B and C);
- LEQUESNE index (Annex 1.3);
- Assessment of overall treatment efficacy by patient and investigator;
- Patient’s overall status score in relation to his knee osteoarthritis on a VAS;
- Use of analgesics and NSAIDs;
- Percentage of patients who did not need to use local treatment;
- Percentage of responder patients according to the OMERACT-OARSI criteria (23) (Annex 1.5).

9.5.1.2.2 Safety
Overall safety is evaluated at C3, C4 and C5:
- By assessment by patient and investigator according to a verbal scale;
- By recording adverse events.
Local safety is evaluated at consultation C3 by patient and investigator according to a verbal scale and by recording manifestations such as: post-injection pain, inflammatory reaction, presence or absence of hydrarthrosis or acute pseudoseptic or septic arthritis.
All side effects occurring during this study will be recorded in the CRF.

9.5.2 Relevance of the endpoints
9.5.2.1 Primary endpoint for efficacy
WOMAC: index of the symptomatic severity of lower limb osteoarthritis.
WOMAC is the validated index for the evaluation of lower limb osteoarthritis. There are two systems for scoring responses to the questions: either the LIKERT scale with 5 possible responses (none = 0; slight = 1; moderate = 2; severe = 3; extreme = 4) or a visual analogue scale of 100 mm. The scores can be calculated in each domain or for WOMAC overall. The mean sub-scores on the WOMAC will be calculated by adding the 5 items relating to pain, the 2 items relating to stiffness and the 17 items relating to physical function, divided by the number of related questions to obtain a value between 0 and 100 for each sub-score.

- Mild to moderate pain is defined by a mean score of 25 mm to 60 mm
- Moderate to severe pain is defined by a mean score > 60 mm

WOMAC Section A Pain domain: what is the extent of the pain?
1. When you walk on a flat surface?
2. When you go up or down stairs?
3. At night, when you are in bed?
4. When you get up from a chair or sit down?
5. When you are standing up?

WOMAC Section B Stiffness domain: what is the extent of the stiffness?
1. When you get up in the morning?
2. When you move after sitting, lying down or resting during the day?

WOMAC Section C Function domain: how difficult do you find the following:
1. Going downstairs?
2. Going upstairs?
3. Getting up from a chair?
4. Standing?
5. Leaning forwards?
6. Walking on flat ground?
7. Getting in and out of a car?
8. Doing shopping?
9. Putting on tights or socks?
10. Getting out of bed?
11. Taking off tights or socks?
12. Lying down on the bed?
13. Getting in and out of a bath?
14. Sitting down?
15. Sitting on and standing up from toilets?
16. Doing thorough housework at home?
17. Doing the daily housework?

9.5.3 Endpoints for clinical safety
9.5.3.1 Adverse events
The term “adverse event” refers to any harmful unwanted event experienced by a person taking part in a biomedical study, regardless of its cause. Any event discovered by the investigator or spontaneously reported by the patient will be described in the CRF on the forms provided for this purpose.
If any serious, unexpected or life-threatening medical event affects the patient, regardless of whether it is considered related to the treatment, TRB CHEMEDICA and the CRO SPRIT must be contacted immediately.

Any event with a fatal outcome or capable of being life-threatening, or resulting in disability or incapacity, or causing or prolonging hospitalisation is defined as serious.
Serious adverse events must be reported within 24 hours by telephone or fax to the sponsor. This notification will be confirmed by immediately sending the duly completed “Pharmacovigilance form”.
Any death must be reported by telephone or fax to the sponsor, as soon as the investigator learns of it.
A detailed written report must be sent to the sponsor within the next three days (Annex 1.16).

A patient can leave the study due to an adverse event at his own discretion or that of the investigator.
In all cases the reason why a patient leaves the study must be clearly stated.
As far as possible a final evaluation must be carried out on the date the patient leaves the trial or as soon as the patient can be seen again.

9.5.3.2 Safety study
This will be purely descriptive.
Side effects will be categorised according to the MedDRA classification which is a list of medical terms developed by the international conference on harmonisation or ICH.
It will be carried out for all the patients who have received an injection as treatment.
It will be based on a study of adverse events which have occurred (name, nature, duration, course) and on dropouts due to intolerance.
All adverse events will be monitored until they have resolved.

9.6 Final report

At the end of the statistical analysis, a full study report will be drafted and signed after approval by the co-ordinator, sponsor, head of statistics, scientific advisor and manager of the trial.

The sponsor, national co-ordinator and investigators are not permitted to disclose in any manner whatsoever the full results of the study without the official authorisation of each party.

9.7 Planned statistical methods and calculation of the number of subjects

9.7.1 Chosen population size
To demonstrate a reduction in overall spontaneous pain in the last 24 hours on a VAS of 100 mm between the inclusion visit and the follow-up visit at 6 months between the OSTENIL PLUS group and the SYNVISC-ONE group, at least 111 subjects must be recruited to each treatment group.

9.7.2 Parameters used to calculate the number of subjects
This number of subjects is based on the following hypotheses:

Type I error	$\alpha = 0.05$
Type II error	$\beta = 0.20$
Clinically significant difference	$\Delta = 8$ mm
Standard deviation	$\sigma = 24$ mm

$$n = \frac{2\sigma^2}{\Delta^2}(z_{1-\alpha} + z_{1-\beta})^2$$

The study aim is to demonstrate the non-inferiority of OSTENIL PLUS compared with the reference product (according to EMEA recommendations:
http://www.emea.eu.int/pdfs/human/ewp/048299en.pdf).

The number of subjects to be included will therefore be estimated by a unilateral approach.
The theoretical number calculated from these data is 111 subjects per group.
A rate of loss of ± 15% is anticipated at M6.
Taking into account patients who may be "lost to follow-up" the total number of patients is 260.

9.7.3 Statistical evaluation

9.7.3.1 Statistical analysis method
A detailed statistical analysis plan will be prepared and validated by the scientific committee.

9.7.3.1.1 Blind review
Description of sample (according to the parameter type), deviations from the protocol, aberrant or missing data.
Decision requests from the co-ordinator and scientific committee of the trial, in particular for classifying deviations from the protocol as "major" and "minor".
After this blind review the following populations will be defined: ITT population and PP population.
Any proposals concerning the results of this analysis, changes to the plan and/or analysis methods, or definition of criteria, forming the subject of an amendment to the protocol.

9.7.3.1.2 Sample description
According to the parameters, means and standard deviations, medians and ranges, numbers in classes.

9.7.3.1.3 Comparison of groups before treatment
This will be done on D 0 using conventional statistical tests:
Chi^2 test for qualitative variables.
Student's test and analysis of variance for quantitative variables.

9.7.3.2 Endpoints

9.7.3.2.1 Primary endpoint for efficacy
The primary analysis will relate to the Per Protocol (PP) population, the secondary analysis to the Intent-to-Treat (ITT) population (to ensure the stability of the results obtained).
The mean differences in the WOMAC score between inclusion and the 6-month visit will be calculated for each group.
For OSTENIL PLUS to be non-inferior to SYNVISC-ONE, the lower limit of the 95% confidence interval of the difference (between OSTENIL PLUS and SYNVISC-ONE) between the WOMAC scale means must be greater than -8 mm (see diagram below).
The zone of equivalence will therefore consist of the 95% confidence interval of the difference (assumed to be zero) from -8 mm to +8 mm.

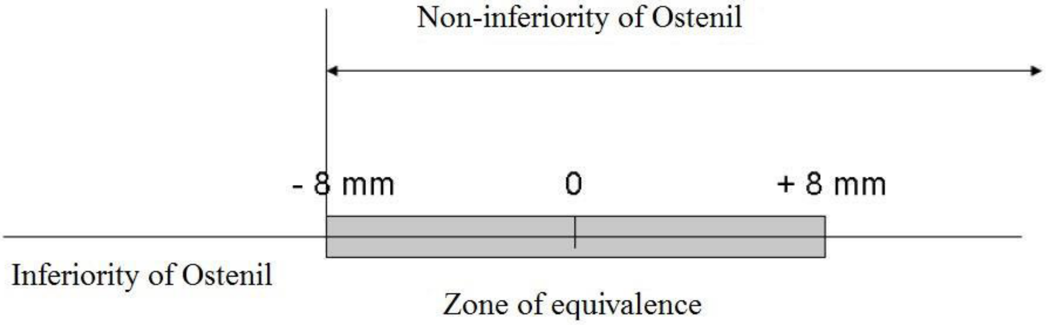

To limit any risk of bias during the analyses, missing data will undergo specific treatment.
A comparative analysis of patients lost to follow-up will initially be carried out to measure potential biases and to identify any covariables on which major differences have been noted.
Then, assuming that missing data are of the type MAR (24), imputation of missing data will secondly be carried out using PROC MI (Multiple Imputation in SAS) or with STATA software.
Analysis according to LOCF, even assuming that missing data are MCAR (24), is probably not correct in this study.
Results involving imputation will be compared with those obtained without imputation to determine the bias caused by these missing data.

9.7.3.2.2 Secondary criteria
The area under the curve of the LEQUESNE algofunctional index will also be compared between groups using STUDENT's t-test for independent data or a WILCOXON test (according to the normality of the distribution of the variable observed).

The course of the LEQUESNE algofunctional index between inclusion and the end of the follow-up period (or the last observation recorded) will be tested in each of the two groups using STUDENT's t-test for paired data, then compared between the two groups using STUDENT's t-test for independent data or a WILCOXON test (according to the normality of the distribution of the variable observed).

The overall assessment by the patient of his condition in relation to his knee osteoarthritis rated on the VAS from 0 to 100 mm will be compared between the groups using STUDENT's t-test for independent data or a WILCOXON test (according to the normality of the distribution of the variable observed).
Assessment of overall efficacy by patient and investigator.
Variations in qualitative criteria (OMERACT-OARSI) will be analysed.

9.7.3.2.3 Endpoint for safety
This will be based on an assessment of overall and local safety by patient and investigator.
Adverse events will be studied in the randomised population.
Adverse events will be classified according to the WHO classification by system/organ and will be counted and described per visit.
Patients will be asked about adverse events appearing during the trial, regardless of whether they caused an early dropout and regardless of their causality or severity.
The number of adverse events will be calculated.

The groups will be compared using a Chi^2 test for the following variables:

<u>Overall incidence</u>
Number of subjects presenting with at least one adverse event / Number of subjects included.

<u>Incidence per event</u>
Number of subjects presenting with the adverse event in question / Number of subjects included. The severity of the events will also be described in each group.

9.7.3.3 Statistical analyses
The primary analysis is the analysis Per Protocol (PP), the secondary analysis is the ITT analysis.
The items in the CRF will be double-entered in the study database by two different operators. These two data entry bases will then be compared and the differences will be systematically checked. Furthermore 10% of these forms will be checked in full against the corrected data entry base.
The data will then be checked by inconsistency check programming and individual data listings will be published, evident corrections will be made and other errors or missing data will be the subject of clarification requests to the investigators.
When the database has been declared complete and valid, it will be locked. Any change in this database after this point must be the subject of a written request by authorised personnel. The data will be anonymised.

All the analyses will be carried out using SAS software and if necessary STATA software for the multiple imputation of missing data.
All data will be listed.
All the statistical tables will be presented per treatment group after the start of the treatment, and by treatment group for the total population at inclusion. If appropriate, the data will also be presented by site.
The statistical analyses will be carried out using descriptive statistics, hypothesis tests and confidence intervals.

The groups will initially be compared for demography, endpoints for efficacy, use of symptomatic treatments for osteoarthritis or none and safety parameters as follows:
For quantitative variables a normality test will first be performed on the total population, and depending on normality, STUDENT's t-test or a non-parametric test (WILCOXON test) will be used to compare the two groups.
For ordinal variables a MANN-WHITNEY test will be performed.
For qualitative variables the comparison will be carried out using either a Chi^2 test or FISHER's exact test.
The quantitative data will be characterised by at least: the number of non-missing data, the number of missing data, the mean, the median, the standard deviation, the minimum and the maximum.
The qualitative data will be characterised by the frequency and percentage by modality, and by the number of missing data.
For all the tests performed, the approach will be bilateral and the risk of the first kind set at 5%.
The robustness of the study conclusions will be analysed by testing the influence of the parameters for which the difference between the two groups will be statistically significant at inclusion (analysis of covariance and multiple regressions and/or adjustment, according to the nature of the parameters).

9.7.3.3.1 Definition of populations analysed
The populations will be defined according to the ICH standard Topic E 9 Statistical Principles for Clinical Trials EMEA September 1998, chapter 5.2 (25).

9.7.3.3.1.1 Intent-to-treat population (ITT)
The patients selected for the intent-to-treat population will be characterised by the following criteria:

For initial comparability and safety: Any patient included (i.e. randomised) in the trial and having received the intra-articular injection of the investigational product will belong to the intent-to-treat population for safety and initial comparability between the two groups.

For efficacy:
- Any patient randomised in the trial
- having received the injection of the investigational product
- having at least one measurement of the primary endpoint for efficacy (WOMAC A) in the follow-up
- having no severe deviations from the selection criteria (severe deviations will be defined specifically by the scientific committee during the blind review).

The exclusion from this group of patients who do not receive the injection is justified because the reason why a patient would not receive the injection does not depend on knowledge of the treatment allocated (as the two treatments are of the same type).

Patients who receive an injection of the product in the group other than the one they were assigned to will be analysed in this latter group (and not according to the product actually received).

9.7.3.3.1.2 Per Protocol population (PP)
The Per Protocol population will include patients in the ITT group who additionally:
- have no major deviations from the protocol (major deviations will be defined specifically by the scientific committee during the blind review).
- have had WOMAC A measured at D 180.

10. CALENDAR

Trial set up	May 2011
End of inclusions	December 2012
End of trial	June 2013
Statistical analyses and results	September 2013
Clinical report	December 2013

PART II: GENERAL PRINCIPLES

1. ETHICS

1.1 Declaration of Helsinki

The Declaration of Helsinki (Annex 2.1), in its most recent revision, is accepted as the ethical basis for clinical studies and must be followed in full and observed by all those involved in a study in humans.

Any exceptions must be justified and mentioned in the same protocol.

1.2 Good Clinical Practice

Clinical studies must be conducted according to Good Clinical Practice (Annex 2.4). A clinical study is understood to include the design, conduct, monitoring, closure, audit, analyses, reports and documentation of studies.

Good Clinical Practice ensures that studies are ethically and scientifically justified, and that the clinical properties of diagnostic, therapeutic or prophylactic investigational products are correctly documented.

2. INVESTIGATORS' RESPONSIBILITIES

It is the responsibility of the investigators to conduct the trial according to the protocol and to ensure that they have the necessary recruitment to carry out the study within the time stated in the study protocol.

2.1 Ethics

2.1.1 Declaration of Helsinki
The study will be conducted according to the principles of medical ethics as defined by the Declaration of Helsinki, revised in 2008.

2.1.2 CPP (Ethics Committee)
The favourable opinion of the CPP must be obtained before the start of the study (Annex 2.5).
It must be sent in writing to the co-ordinator.
A copy must be sent to the sponsor.
A copy appears in the annex to the protocol.

2.1.3 Informing the patient
Each patient must be informed fully and unambiguously that he is free to refuse to take part in the study or that he can withdraw his consent at any time, for any reason whatsoever, without incurring any disadvantage as regards the care he should receive from the investigator for his state of health.

The patient will be given an information sheet (Annex 2.7).

2.1.4 Patient informed consent
It is the responsibility of the investigators to obtain the written informed consent of each patient taking part in the study (Annex 2.8) after having correctly explained to him/her the aims, methods, benefits and potential risks. The consent must be obtained before any procedure specific to the study is undertaken.
A copy of the consent is given to the patient.
For patients who are unable to give their written informed consent for any reason whatsoever, it must be obtained from their legal guardian.
The signed informed consent must be filed by the investigator and documented in the CRF and the patient's medical notes.

2.1.5 Withdrawal of a subject
The investigator has the right to withdraw a subject from the study for any reason whatsoever, if it is in the patient's interest (including in the event of an intercurrent disease, adverse events or treatment failure).
Whenever a patient is withdrawn from the trial for any reason whatsoever, an end of trial evaluation must be carried out, mentioning the reason for the withdrawal.
Withdrawals for non-participation must be monitored to identify the reasons for this.
Withdrawals for an intercurrent disease or adverse events must be fully documented in the CRF, with all additional available appropriate information.

2.1.6 Patient anonymity
The investigators must ensure that patient anonymity is preserved.
The patient must be identified by an identification code only on all the documents given to the sponsor, not by name or hospital record number.
The investigators must keep a confidential inclusion form which links the identification code and the patients' names/addresses.
Documents which will not be given to the sponsor will be kept strictly confidential by the investigators.

2.1.7 Fees
The following will be covered by TRB CHEMEDICA, the sponsor of this study:

- The fees of the investigating physicians for study consultations
- Treatments associated with the study.

2.2 Amendments to the protocol

In accordance with articles L 1123-9 and R 1123-35 of the public health code, any substantial amendment to the study by the sponsor must obtain the favourable opinion of the ethics committee and/or authorisation by the AFSSAPS before it is implemented, depending on the aspects of the study amended.

Each amendment to the protocol will be signed by the co-ordinator and the sponsor.

The favourable opinion of the ethics committee and/or authorisation by the AFSSAPS will be sent in writing to the co-ordinator, to TRB CHEMEDICA and to each investigator.

2.3 Case report form

The triplicate CRF (Annex 1.12) is designed to record all data necessary for analysis of the study, including the patients' medical histories and any adverse events.
The data will be entered in the form by hand, preferably in blue ballpoint pen.
For any alteration or addition made to the CRF, the investigator should cross out the initial data with a single line, write the new data alongside and then initial and date the change.
The following annotations should be used: MD missing data, NA not applicable, NK not known, ND not done.

2.4 Checking source documents

All data obtained during a clinical trial must be treated in confidence to ensure patients' rights to privacy.

The investigators undertake to agree that the monitor, auditor and inspector may have access to all documents necessary for checking the source documents and the appropriate monitoring of the progress of the study.
If direct checking of source documents is not permitted by law, the investigator then undertakes to assist the monitor, auditor or inspector in the process of validating data quality.

2.5 Adverse events

2.5.1 Definition
An adverse event is any harmful unwanted manifestation experienced by a person taking part in a biomedical study, regardless of its cause.

An adverse event is serious when:

- It is life-threatening for the patient or has a fatal outcome and/or causes a serious permanent disability.
- It causes or prolongs hospitalisation of the patient.
- A congenital malformation or malignancy appears.
- It is related to an accidental or intentional overdose.
- It seems to suggest an imminent overdose, a contraindication, a special precaution or a serious adverse event.

Causal link:
Excluded:
This should be reserved for events occurring before the administration of the medicinal product (e.g. wash-out period) or for events which cannot even vaguely be related to participation in the study (e.g. injuries sustained in a car accident).

Unlikely:
Due to the chronological relationship between the start of the treatment and the date the associated adverse manifestation occurred (potentially an adverse event).

Possible:
Reasonable chronological relationship in relation to the start of the treatment.
Possible relationship with the patient's clinical condition or with another treatment.

Probable:
Reasonable chronological relationship in relation to the start of the treatment.
Disappearance of the associated adverse manifestation after the end of treatment with the suspect product.
No evident reasonable explanation, given the patient’s known clinical condition.

Certain:
Reasonable chronological relationship in relation to the start of the treatment.
Disappearance of the associated adverse manifestation after the end of treatment with the suspect product.
After rechallenge with the suspect product, reappearance of the associated adverse manifestation.

Any event discovered by the investigator or spontaneously reported by the patient will be described in the CRF on the forms provided for this purpose.
If any serious, unexpected or life-threatening medical event affects the patient, regardless of whether it is considered related to the treatment, TRB CHEMEDICA and SPRIT must be contacted immediately.

2.5.1.1 Serious adverse events
Any event with a fatal outcome or which is likely to be life-threatening or which results in disability or incapacity or which causes or prolongs hospitalisation is defined as serious.

2.5.1.2 Serious adverse event report
Serious adverse events must be reported within 24 hours by telephone or fax to the sponsor TRB CHEMEDICA and to SPRIT.

This notification should be confirmed by immediately sending the duly completed “Serious adverse event (SAE) report”.

In accordance with article R 1123-48 of the public health code, suspected serious unexpected adverse effects and serious adverse events that can be related to the use of the test device must be reported by the sponsor as soon as he learns of it, and at the latest within 15 calendar days of the date when he was informed of it, to AFSSAPS and the ethics committee.

Regardless of how long after the end of the trial it occurs, any SAE likely to be due to the study must be reported when it comes to the knowledge of the sponsor and when no cause other than the study can reasonably be blamed.

2.5.1.3 Death
Any death must be reported by telephone or fax to the sponsor TRB CHEMEDICA and to SPRIT as soon as the investigator learns of it. A detailed written report must be sent to the medical directorate of TRB CHEMEDICA within the next three days.
Reports of deaths or life-threatening events will be made by the sponsor as soon as he learns of them and, at the latest, within 7 calendar days of the date when he was informed of them.
An additional detailed written report will be sent to the competent authorities within 15 days of the date of the first information.

2.5.1.4 Dropouts
A patient can leave the study due to an adverse event at any time at his own discretion or that of the investigator.

In all cases, the reason why a patient leaves the study must be clearly stated on the end of study page and, as far as possible, a final evaluation should be carried out on the date the patient leaves the trial or as soon as the patient can be seen again.

2.5.1.5 Non-serious adverse events
Adverse events not mentioned in the above definitions are not serious and can be classed as:
Mild:
Appearance of symptoms readily tolerated not requiring any treatment.

Moderate:
Sufficient discomfort to disturb usual activity (at least one day of corrective treatment is necessary).

Severe:
Appearance of a significant risk resulting in:

- Contraindications to the treatment;
- Or requiring special precautions;
- Or resulting in an inability to carry out usual activities;
- Or requiring major countermeasures;
- Or requiring the withdrawal of the test treatment.

2.5.1.6 Treatment and monitoring of adverse events
Adverse events must be documented and monitored until the event has either resolved or been adequately explained, even after the subject has completed the test treatment.

2.6 Publications

TRB CHEMEDICA reserves the right to examine all manuscripts and/or summaries of trial results before their submission for publication or presentation.
This measure is not intended to restrict or prevent publication or presentation but to allow TRB CHEMEDICA to protect patented information and/or to make comments based on information which might not be available to the investigators.

2.7 Archiving

The investigators are required to keep all data relating to the study, including the informed consent form for each patient, for a minimum of 15 years after its end.

2.8 Audit

The investigators undertake to comply with the requests of TRB CHEMEDICA and the Health Authorities as regards the study audit (also see checking source documents).

3. RESPONSIBILITIES OF THE SPONSOR AND SERVICE PROVIDERS

3.1 General responsibilities

TRB CHEMEDICA provides an updated scientific information brochure (Annex 1.14) or all relevant information for each of the products in a clinical trial, whether or not it is registered.
For marketed products, the updated product leaflet and/or monograph is provided.
TRB CHEMEDICA, the scientific advisor, the statistician and the monitoring company produce a final protocol version.
SPRIT provides the investigators with a sufficient number of CRFs.
TRB CHEMEDICA reserves the right to stop the trial early due to repeated persistent deviations from the protocol or for any other valid ethically justified reason.
In this eventuality the various participants will, after examination and consultation, implement the necessary procedures to ensure the protection of patients' interests.

3.2 Insurance

If the trial has been conducted in accordance with the protocol, the civil liability of the investigators is covered by the civil liability insurance policy taken out by TRB CHEMEDICA (Annex 2.9).

3.3 Monitoring and responsibilities of service providers

The study is conducted in accordance with Good Clinical Practice.
The study will be regularly monitored by a Clinical Research Associate (CRA) to check correct compliance with the protocol and transcription of the data in the CRFs.
The investigators undertake in accordance with Good Clinical Practice to keep medical archives containing reliable data.
The Clinical Research Associate must have access to these data during monitoring visits.
Telephone contacts with the investigators will take place.

3.4 Quality assurance and quality control

Any document used in clinical studies is subject to quality control.
Quality assurance audits can be carried out by TRB CHEMEDICA or any Health Authority during the conduct of the study or after its completion.

3.5 Archives

TRB CHEMEDICA and the service providers (SPRIT, CEMKA) must store the documents relating to this study (protocol, documentation, protocol approval, audit and inspection report, and any data relating to the biomedical study) for a period of 15 years after the end of the study or its early termination (order of 11 August 2008).
All the documents must be archived in a secure place and treated in strictest confidence.

4. SIGNATURES

EFFICACY OF OSTENIL PLUS (HYALURONIC ACID) VERSUS SYNVISC-ONE IN PATIENTS WITH TIBIOFEMORAL OSTEOARTHRITIS

Done in six originals on 16 MAY 2011

National Co-ordinator
Professor THOMAS BARDIN

[Signature]

Sponsor
TRB CHEMEDICA
Dr CHARLES JOLLES

[Signature]

Scientific Advisor
Dr RENEE LILIANE DREISER

[Signature]

Statistician
CEMKA
Dr ANTOINE LAFUMA

[Signature]

Trial Manager
SPRIT
MARC PIOCHAUD

[Signature]

PART III: ANNEXES

1. ANNEXES RELATED TO THE STUDY
1.1 ACR criteria for tibiofemoral osteoarthritis
1.2 Kellgren-Lawrence grading scale
1.3 Lequesne algofunctional index knee osteoarthritis
1.4 WOMAC – Western Ontario McMaster's Arthritis Index
1.5 OMERACT-OARSI criteria
1.6 List of NSAID treatments
1.7 NSAID wash-out periods
1.8 Visual analogue scale (VAS)
1.9 Analysis certificate
1.10 Co-ordinator's curriculum vitae
1.11 Investigators participating in the trial
1.12 Case report forms
1.13 Patient form
1.14 Information sheet OSTENIL PLUS & SYNVISC-ONE
1.15 Pharmacovigilance form and serious adverse event (SAE) report
1.16 Acknowledgement of receipt
1.17 Confidentiality undertaking and protocol approval

2. ANNEXES RELATED TO LEGAL OBLIGATIONS
2.1 Declaration of Helsinki
2.2 Huriet law
2.3 Implementing circular on DMOS (*Diverses Mesures d'Ordre Social* – French Anti-Gift Law or French DMOS Law)
2.4 Good clinical practice
2.5 Opinion of the CPP (Ethics Committee)
2.6 Opinion of the AFSSAPS (French health products safety agency)
2.7 Patient information leaflet
2.8 Patient informed consent
2.9 Insurance certificate
2.10 References

ANNEX 1.1
ACR CRITERIA FOR TIBIOFEMORAL OSTEOARTHRITIS

AMERICAN COLLEGE OF RHEUMATOLOGY CLASSIFICATION CRITERIA FOR OSTEOARTHRITIS OF THE KNEE

Definition criteria for knee osteoarthritis

A patient is considered to be suffering from knee osteoarthritis if they suffer from

1. Knee pain

AND

If they meet 1 of the 3 following criteria:

1. Age > 50
2. Morning stiffness < 30 minutes
3. Cracking joints

AND

A knee X-ray has revealed osteophytes

The sensitivity and specificity of the criteria are 91% and 86% respectively.

(18) Reference: Altman and Al., Arthritis Rheum., 1986, 29, pp. 1039-1049

ANNEX 1.2
KELLGREN-LAWRENCE
GRADING SCALE

KELLGREN-LAWRENCE GRADING SCALE

Grade I Mild joint space narrowing

Grade I_A < 25% joint space narrowing
Negligible osteophytes

Grade I_B 25–50% joint space narrowing
Negligible osteophytes

Grade II Definite joint space narrowing – approximately 50%
Definite osteophytes
Mild osteosclerosis

Grade III Pronounced joint space narrowing > 50%
Large osteophytes
Some sclerosis and possible deformity of bone ends

Grade IV Marked joint space narrowing (narrowing > 75%)
Large osteophytes
Severe sclerosis and cysts
Deformity of bone ends

(19) Reference: Kellgren JH, Lawrence DM - Radiological assessment of osteoarthritis. Ann Rheum Dis 1957;16:494-502

ANNEX 1.3
LEQUESNE
ALGOFUNCTIONAL INDEX
FOR TIBIOFEMORAL OSTEOARTHRITIS

LEQUESNE ALGOFUNCTIONAL INDEX FOR KNEE OSTEOARTHRITIS

**NOCTURNAL PAIN OR DISCOMFORT?**		I__I pt
None	0 pt	
Only on movement and in certain positions	1 pt	
Even without moving	2 pt	
**REGRESSION OF MORNING STIFFNESS?**		I__I pt
Less than 1 minute	0 pt	
Between 1-15 minutes	1 pt	
More than 15 minutes	2 pt	
**DOES REMAINING STANDING OR WAITING AROUND FOR ½ HOUR INCREASE THE PAIN?**		I__I pt
No	0 pt	
Yes	1 pt	
**PAIN ON WALKING?**		I__I pt
None	0 pt	
After a certain distance	1 pt	
Or from the beginning and increasingly	2 pt	
**CAN YOU GET UP WITHOUT USING YOUR ARMS?**		I__I pt
Yes	0 pt	
No	1 pt	
**MAXIMUM WALKING DISTANCE** (may walk with pain)		I__I pt
Unlimited	0 pt	
Limited but over 1 km but (about 15 minutes)	1 pt	
About 1 km	2 pt	
500–900 m (about 8–15 min)	3 pt	
300–500 m	4 pt	
100–300 m	5 pt	
Less than 100 m	6 pt	
A walking stick or crutch needed	+1 pt	
Two walking sticks or crutches needed	+2 pt	
**OTHER DIFFICULTIES***		
Can you go up one floor?	0–2 pt	I__I.I__I pt
Can you go down one floor?	0–2 pt	I__I.I__I pt
Can you squat or stay on your knees?	0–2 pt	I__I.I__I pt
Can you walk on uneven ground?	0–2 pt	I__I.I__I pt

*** Score: 0 = without difficulty; 1 = possible, with difficulty (or 0.5 or 1.5 depending on the degree of difficulty); 2 = impossible**

TOTAL I__I__I,I__I pts

(20) Reference: Lequesne M, Méry C, Samson M, Gérard P - Indexes of severity for osteoarthritis of the hip and knee. Scand J Rheumatol 1987;65(Suppl):85-9.

ANNEX 1.4
WOMAC

WOMAC: a symptomatic severity index for lower limb osteoarthritis
The WOMAC is widely used to evaluate osteoarthritis of the hip or knee. There are two formats available for answering the questions: the LIKERT scale which has 5 possible responses (none = 0; mild = 1; moderate = 2; severe = 3; extreme = 4) and the 100 mm visual analogue scale. The scores can be calculated for each domain or for the entire WOMAC.

WOMAC sub-score means are calculated by adding the 5 pain items, the 2 stiffness items and the 17 physical function items, divided by the number of related questions in order to obtain a value between 0 and 100 for each sub-score.

SECTION A

WOMAC Pain sub-scale: Indicate the severity of the pain:

1. Walking on a flat surface?
   No pain____Maximum pain

2. Going up/downstairs?
   No pain____Maximum pain

3. At night, in bed?
   No pain____Maximum pain

4. Getting in/out of a chair?
   No pain____Maximum pain

5. Standing upright?
   No pain____Maximum pain

SECTION B

WOMAC Stiffness sub-scale: Indicate how severe the stiffness is:

1. After first waking?
   No stiffness____Extreme stiffness

2. Later in the day, when you move after sitting, lying down or resting?
   No stiffness____Extreme stiffness

SECTION C

WOMAC Physical function sub-scale: Indicate the degree of difficulty you experience when:

1. Descending stairs?

   No difficulty____Extreme difficulty

2. Going upstairs?

   No difficulty____Extreme difficulty

3. Rising from sitting?

   No difficulty____Extreme difficulty

4. Standing?

   No difficulty____Extreme difficulty

5. Bending forward?

   No difficulty____Extreme difficulty

6. Walking on a flat surface?

   No difficulty____Extreme difficulty

7. Getting in/out of a car?

   No difficulty____Extreme difficulty

8. Going shopping?

   No difficulty____Extreme difficulty

9. Putting on socks/stockings?

   No difficulty____Extreme difficulty

10. Rising from bed?

    No difficulty____Extreme difficulty

11. Taking off socks/stockings?

    No difficulty____Extreme difficulty

12. Lying in bed?

    No difficulty____Extreme difficulty

13. Getting in/out of the bath?

    No difficulty____Extreme difficulty

14. Siting?

    No difficulty____Extreme difficulty

15. Getting on/off the toilet?

    No difficulty____Extreme difficulty

16. Doing thorough housework at home?

    No difficulty____Extreme difficulty

17. Doing the daily housework?

    No difficulty____Extreme difficulty

(17) Reference: Bellamy N, Buchan WW, Goldsmith CH, Campbell J, Stit LWJ. Validation of WOMAC: a health status instrument for measuring clinically important patient relevant outcomes to antirheumatic drug therapy in patients with osteoarthritis of the hip or knee. J Rheumatol 1995; 15: 1833-40

ANNEX 1.5
OMERACT-OARSI CRITERIA

OMERACT-OARSI set of responder criteria

The responders are defined by:

A) An improvement of at least 50% (≥ 50%) in pain or function and an absolute change of at least 20 mm (≥ 20 mm) on the 100 mm VAS

B) Or an improvement in at least 2 of the 3 following criteria:

1. Pain improved by at least 20% (≥ 20%) and absolute change of at least 10 mm (≥ 10 mm)
2. Function improved by at least 20% (≥ 20%) and absolute change of at least 10 mm (≥ 10 mm)
3. Patient’s overall assessment improved by at least 20% (≥ 20%) and absolute change of at least 10 mm (≥ 10 mm)

(23) Reference: Pham T, van der Heijde D, Altman RD, Anderson JJ, Bellamy N, Hochberg M, Simon L, Strand V, Woodworth T, Dougados M. OMERACT-OARSI Initiative: Osteoarthritis research society international set of responder criteria for osteoarthritis clinical trials revisited. Osteoarthritis cartilage 2004; 12: 389-399

ANNEX 1.6
LIST OF NSAID TREATMENTS

ANNEXE 1.6

SHORT HALF-LIFE NSAIDS

**ACECLOFENAC**	Cartrex	100 mg		
**ALMINOPROFEN**	Minalfen	300 mg		
**DICLOFENAC**	Artotec	50 mg	75 mg	
	Voltaren	25 mg	50 mg	
	Xenid	50 mg		
**ETODOLAC**	Lodine	200 mg	300 mg	
**FLURBIPROFEN**	Antadys	100 mg		
	Cebutid	50 mg	100 mg	200 mg
**IBUPROFEN**	Advil	200 mg	400 mg	
	Antarene	100 mg	200 mg	400 mg
	Brufen	400 mg		
	Nurofen	200 mg		
**INDOMETHACIN**	Indocid	25 mg		
**KETOPROFEN**	Ketum	100 mg		
	Profenid	50 mg	100 mg	
	Topfena	50 mg		
**NIFLUMIC ACID**	Nifluril	250 mg		
**NIMESULIDE**	Nexen	100 mg		
**SULINDAC**	Arthrocine	100 mg	200 mg	
**TIAPROFENIC ACID**	Flanid	100 mg	200 mg	
	Surgam	100 mg	200 mg	

ANNEXE 1.6

LONG HALF-LIFE NSAIDS

**CELECOXIB**	Celebrex	100 mg	200 mg	
**DICLOFENAC**	Voltaren	75 mg ER	100 mg ER	
**ETORICOXIB**	Arcoxia	30 mg	60 mg	
**IBUPROFEN**	Nureflex	300 mg ER		
**INDOMETHACIN**	Chrono-indocid	75 mg		
**KETOPROFEN**	Bi-Profenid	100 mg ER		
	Ketum	200 mg ER		
	Profenid	200 mg ER		
	Topfena	200 mg ER		
**MELOXICAM**	Mobic	7.5 mg	15 mg	
**NABUMETONE**	Nabucox	500 mg		
**NAPROXEN**	Apranax	275 mg	550 mg	750 mg
	Naprosyn	250 mg	500 mg	
	Naprosyn	750 mg	1000 mg	
**PIROXICAM**	Brexin	20 mg		
	Cycladol	20 mg		
	Feldene	10 mg	20 mg	
	Proxalyoc	20 mg		
**TENOXICAM**	Tilcotil	20 mg		

ANNEX 1.7
NSAID WASH-OUT PERIODS

**SHORT HALF-LIFE NSAID**	Brand name	½ life	Wash out in days
**ACECLOFENAC**	Cartrex	4 hours	2 days
**ALMINOPROFEN**	Minalfen	4–6 hours	2 days
**DICLOFENAC**	Artotec	3–6 hours	2 days
	Voltaren	2 hours	2 days
	Xenid	2 hours	2 days
**ETODOLAC**	Lodine	7 hours	2 days
**FLURBIPROFEN**	Antadys	3.5–4 hours	2 days
	Cebutid	3.5–4 hours	2 days
**IBUPROFEN**	Advil	2 hours	2 days
	Antarene	2 hours	2 days
	Brufen	2 hours	2 days
	Nurofen	2 hours	2 days
**INDOMETHACIN**	Indocid	2 hours	2 days
**KETOPROFEN**	Ketum	2–3 hours	2 days
	Profenid	2 hours	2 days
	Topfena	1.5–2 hours	2 days
**NIFLUMIC ACID**	Nifluril	4–6 hours	2 days
**NIMESULIDE**	Nexen	3–6 hours	2 days
**SULINDAC**	Arthrocine	8 hours	2 days
**TIAPROFENIC ACID**	Flanid	1.5–2.5 hours	2 days
	Surgam	1.5–2.5 hours	2 days

SHORT HALF-LIFE NSAID	Brand name	½ life	Wash out in days
**CELECOXIB**	Celebrex	8–12 hours	3 days
**DICLOFENAC**	Voltaren ER	2 hours	2 days
**ETORICOXIB**	Arcoxia	22 hours	5 days
**IBUPROFEN**	Nureflex ER	7 hours	2 days
**INDOMETHACIN**	Chrono-indocid	2.6–11.2 hours	3 days
**KETOPROFEN**	Ketum ER	6 hours	2 days
	Profenid ER	2 hours	2 days
	Topfena ER	6 hours	2 days
**MELOXICAM**	Mobic	20 hours	4 days
**NABUMETONE**	Nabucox	20–24 hours	5 days
**NAPROXEN**	Apranax	13–15 hours	3 days
	Naprosyn	13–15 hours	3 days
**PIROXICAM**	Brexin	50 hours	10 days
	Cycladol	50 hours	10 days
	Feldene	50 hours	10 days
	Proxalyoc	50 hours	10 days
**TENOXICAM**	Tilcotil	70 hours	15 days

ANNEX 1.8
VISUAL ANALOGUE SCALE (VAS)

VISUAL ANALOGUE SCALE (VAS) FOR SPONTANEOUS PAIN

SERIOUSNESS OF THE PAIN
**No pain**____**Unbearable pain**
VAS |_|_|_|_| mm

21) Reference: Huskisson EC. Measurement of pain. Lancet 1974; II: 1127-31

ANNEX 1.9
ANALYSIS CERTIFICATE
(NOT APPLICABLE)

ANNEX 1.10

CO-ORDINATOR'S CURRICULUM VITAE

CURRICULUM VITAE

Surname: Bardin
First names: Thomas Pierre Jean

PERSONAL INFORMATION

POSITIONS HELD IN HOSPITALS

PERSONAL INFORMATION

TEACHING POSITIONS

PERSONAL INFORMATION

LEARNED SOCIETIES

PERSONAL INFORMATION

READING COMMITTEES

PERSONAL INFORMATION

WORK PERFORMED WITH THE FRENCH NATIONAL HEALTH PRODUCTS SAFETY AGENCY

PERSONAL INFORMATION

MAIN RESEARCH ACTIVITIES

PERSONAL INFORMATION

ANNEX 1.11

INVESTIGATORS PARTICIPATING IN THE TRIAL

INVESTIGATORS – RHEUMATOLOGISTS

Title	Surname	First name	Address	Code	Town	Telephone	CNOM No.
PERSONAL INFORMATION							

INVESTIGATORS – GENERAL PRACTIONERS

Title	Surname	First name	Address	Code	Town	Telephone	CNOM No.

PERSONAL INFORMATION

INVESTIGATORS – GENERAL PRACTIONERS (CONT'D)

Title	Surname	First name	Address	Code	Town	Telephone	CNOM No.
PERSONAL INFORMATION							

INVESTIGATORS – GENERAL PRACTIONERS (CONT'D)

Title	Surname	First name	Address	Code	Town	Telephone	CNOM No.

PERSONAL INFORMATION

INVESTIGATORS – RHEUMATOLOGISTS

Title	Surname	First name	Address	Code	Town	Telephone	CNOM No.

PERSONAL INFORMATION

INVESTIGATORS – RHEUMATOLOGISTS (CONT'D)

Title	Surname	First name	Address	Code	Town	Telephone	CNOM No.

PERSONAL INFORMATION

INVESTIGATORS – RHEUMATOLOGISTS (CONT'D)

Title	Surname	First name	Address	Code	Town	Telephone	CNOM No.

PERSONAL INFORMATION

INVESTIGATORS – RHEUMATOLOGISTS (CONT'D)

Title	Surname	First name	Address	Code	Town	Telephone	CNOM No.

PERSONAL INFORMATION

INVESTIGATORS – RHEUMATOLOGISTS (CONT'D)

Title	Surname	First name	Address	Code	Town	Telephone	CNOM No.

PERSONAL INFORMATION

INVESTIGATORS – RHEUMATOLOGISTS (CONT'D)

Title	Surname	First name	Address	Code	Town	Telephone	CNOM No.

PERSONAL INFORMATION

INVESTIGATOR – PHYSICAL MEDICINE AND REHABILITATION SPECIALIST

Title	Surname	First name	Address	Code	Town	Telephone	CNOM No.

PERSONAL INFORMATION

ANNEX 1.12
CASE REPORT FORMS
(SEE SEPARATE DOCUMENTS)

ANNEX 1.13
PATIENT FORM
(SEE SEPARATE DOCUMENT)

ANNEX 1.14
INFORMATION SHEET
OSTENIL PLUS & SYNVISC-ONE

OSTENIL PLUS medical device

HOW SUPPLIED:
Viscoelastic solution for injection into the joint cavity: Pre-filled syringes of 40 mg/2 ml, in a sterile pack, individually packaged. Sterilised by moist heat.

COMPOSITION:
Isotonic solution*: per ml
Sodium hyaluronate** 20 mg
Excipients: sodium chloride, monosodium phosphate, disodium phosphate, mannitol, water for injections.
*pH = 7.3
** from fermentation

PROPERTIES:
Synovial fluid, which is viscoelastic due to the presence of hyaluronic acid, is found in all synovial joints, particularly the large weight-bearing joints, where it ensures normal painless movement due to its lubricating and shock-absorbing properties. It is also responsible for the nutrition of the cartilage.
In degenerative joint disorders such as osteoarthritis, the viscoelasticity of the synovial fluid is markedly reduced, thereby decreasing its lubricating and shock-absorbing functions. This increases mechanical loading of the joint and cartilage destruction, which ultimately results in restricted mobility of the affected joint and the onset of pain. Supplementing the synovial fluid with intra-articular injections of highly purified hyaluronic acid can improve the viscoelastic properties of synovial fluid.
An improvement in its lubricating and shock-absorbing properties and a reduction in mechanical overload of the joint are observed. As a rule, this results in a decrease in pain and an improvement in joint mobility which may last for several months after treatment.
OSTENIL PLUS is a transparent solution of natural and highly purified hyaluronic acid obtained by bacterial fermentation and is devoid of animal protein.
OSTENIL PLUS also contains mannitol, a free radical scavenger, which helps to stabilise the chains of hyaluronic acid. In biocompatibility studies OSTENIL PLUS was found to be well tolerated.

USE:
Pain and restricted mobility in degenerative and traumatic changes of the knee joint and other synovial joints.

METHOD OF ADMINISTRATION:

Inject 1 syringe of OSTENIL PLUS into the affected joint.
The injections can be repeated with up to 3 consecutive injections in total, with an interval between the injections of at least one week.
Several joints may be treated at the same time.
Repeat treatment cycles may be administered as required.
In case of joint effusion, it is advisable to reduce the effusion by aspiration, rest, application of an ice pack and/or intra-articular corticosteroid injection. Treatment with OSTENIL PLUS can be started two to three days later.
The content and the outer surface of the OSTENIL PLUS pre-filled syringe are sterile as long as the sterile packaging is intact. Take the pre-filled syringe out of the sterile pack. Before use the security seal of the pre-filled syringe should be broken. Break the cross-piece of the security seal. The end-piece can be pulled out with the cap. Attach a suitable needle (for example 18 to 25 G) and secure it by turning slightly. Remove any air bubbles from the syringe, if present, before injection.

CONTRAINDICATIONS:
Previous hypersensitivity to any of the ingredients.

PRECAUTIONS FOR USE:
Particular caution should be exercised in patients with known hypersensitivity to medicinal products.
The general precautions for intra-articular injections should be observed, including measures to avoid joint infections.
OSTENIL PLUS should be injected specifically into the joint cavity, if necessary under imaging guidance. Avoid injections into blood vessels or surrounding tissues. As no clinical data are available on the use of hyaluronic acid in children, pregnant or breast-feeding women, or in inflammatory joint diseases such as rheumatoid arthritis or ankylosing spondylitis, treatment with OSTENIL PLUS is not recommended in these cases.
Do not use if the pre-filled syringe or sterile packaging are damaged.

Interactions:
No information on the incompatibility of OSTENIL PLUS with other solutions for intra-articular use is available to date. The concomitant use of an oral analgesic or anti-inflammatory drug during the first few days of treatment may be helpful for the patient.

Undesirable effects:
Local side effects such as pain, sensation of heat, redness and swelling may occur in the joint treated with OSTENIL PLUS. Application of an ice pack for 5 to 10 minutes onto the treated area will reduce the incidence of these effects.

STORAGE CONDITIONS:
Store at room temperature not exceeding 25°C.
Do not use after the expiry date stated on the box.
Keep out of the reach of children.

Recommended price 76.00 Euros (1 syringe)
ACL 3401096650204
Class III CE marking.

TRB CHEMEDICA
Immeuble ABC 1 Site d'Archamps
P.O. Box 40218.
74162 Archamps cedex
Tel.: 04 50 95 09 00 Fax: 04 50 95 09 01

(26) Reference: Vidal Compendium OCP [pharmacy wholesaler] ed, Paris 2011

SYNVISC-ONE· HYLAN G-F 20

HOW SUPPLIED:
SYNVISC-ONE, 6 ml: 10 ml glass syringe (ACL 486 402.2).
Hylan G-F 20 is an elastoviscous, sterile and non-pyrogenic fluid containing hylans. Hylans are derivatives of hyaluronan (sodium hyaluronate) which contain repeating disaccharide units of Nacetylglucosamine and sodium glucaronate.
Hylan A has an mean molecular weight of 6,000,000 daltons and hylan B is a hydrated gel. Hylan G-F 20 contains hylan A and hylan B (8.0 mg ± 2.0 mg/ml) in a buffered physiological sodium chloride solution (pH 7.2 ± 0.3).

COMPOSITION: per syringe
Per 1 ml (hylan G-F 20): hylan 8.0 mg, sodium chloride 8.5 mg, disodium phosphate 0.16 mg, monosodium phosphate hydrate 0.04 mg, water for injections qs.
SYNVISC-ONE contains 6 ml hylan G-F 20.

PROPERTIES
Hylan G-F 20 is biologically similar to hyaluronan. Hyaluronan is a component of synovial fluid which is responsible for its viscoelasticity. The mechanical (viscoelastic) properties of hylan G-F 20 are however superior to those of synovial fluid and hyaluronan solutions of comparable concentration. Hylan G-F 20 has an elasticity (storage modulus G′) at 2.5 Hz of 111 ± 13 Pascals (Pa) and a viscosity (loss modulus G″) of 25 ± 2 Pa. The elasticity and viscosity of knee synovial fluid of subjects aged 18 to 27 years old measured with a comparable method at 2.5 Hz are G′ = 117 ± 13 Pa and G″ = 45 ± 8 Pa. Hylans are metabolised in the body by the same pathway as hyaluronan and the breakdown products are non-toxic.

INDICATIONS:
Hylan G-F 20:
is a temporary replacement and supplement for synovial fluid;
has proven to be effective when administered in all stages of joint pathology;
has proven to be most effective when administered to patients whose regular physical activity mobilises the affected joint;
achieves its therapeutic effect through viscosupplementation, a process whereby the physiological and rheological properties of the arthritic joint tissues are restored.
Viscosupplementation with hylan G-F 20 is a treatment to decrease pain and discomfort, allowing more extensive movement of the joint. In vitro studies have shown that hylan G-F 20 protects cartilage cells against certain physical and chemical damage.

POSOLOGY AND METHOD OF ADMINISTRATION:

SYNVISC-ONE is only intended for intra-articular use by a physician to treat pain associated with osteoarthritis of the knee.

Remove synovial fluid or any effusion before each hylan G-F 20 injection.
Do not use hylan G-F 20 if the package is opened or damaged.
Inject at room temperature.
To remove the syringe from the blister (or tray) take hold of it by the body, without touching the plunger rod.
Inject using strict aseptic procedures taking particular care when removing the tip cap.
Twist the grey tip cap before pulling it off as this will minimise product leakage.

Use a needle with an appropriate diameter: 18 to 20 gauge SYNVISC-ONE syringe.
Use a needle with an appropriate length depending on joint to be treated.
To ensure a tight seal and prevent leakage during administration secure the needle firmly to the neck of the Luer lock syringe.
Do not tighten or apply excessive leverage when attaching the needle or removing the needle guard as this may break the tip of the syringe.
Do not resterilise hylan G-F 20.
Inject into the synovial space only, using fluoroscopic guidance if necessary, especially when treating the hip and shoulder joints.
The syringe contents are for single use only.
When using radioscopic guidance an ionic contrast agent may be used.
No more than 1 ml of contrast agent should be used for 2 ml of hylan G-F 20.

Posology:

The posology for hylan G-F 20 is dependent on the joint being treated.

Osteoarthritis of the knee:

The recommended treatment regimen for SYNVISC-ONE is one injection in the knee. A second injection may be carried out 6 months after the first, if justified by the patient's symptoms.

Duration of effect:

Hylan G-F 20 treatment affects only the treated joint; it does not produce a systemic effect.
SYNVISC-ONE: prospective clinical data show an effect of treatment up to 26 weeks after a single injection of SYNVISC-ONE.

CONTRAINDICATIONS:

If venous or lymphatic stasis is present in the relevant limb hylan G-F 20 should not be injected into the joint.

Hylan G-F 20 should not be used in infected or severely inflamed joints or in patients with skin diseases or infections at the injection site.

PRECAUTIONS FOR USE:

Warnings:

Do not inject intravascularly.

Do not inject outside of the joint cavity or into the synovial tissue or the capsule.
Do not concomitantly use disinfectants containing quaternary ammonium salts for skin preparation because hyaluronate can precipitate in their presence.

Precautions:

Hylan G-F 20 should not be used if there is a large intra-articular effusion prior to the injection.

As following any invasive joint procedure, it is recommended that the patient avoid any strenuous physical activity after the intra-articular injection and resume normal activities after a few days.

Hylan G-F 20 has not been evaluated in pregnant women or children under 18 years of age.

Hylan G-F 20 contains small amounts of avian protein and should not be used in patients with hypersensitivities to these proteins.

Undesirable effects:

Intra-articular injections of hylan G-F 20 may result in transient pain, oedema and/or effusion. Synvisc has shown that in some cases the effusion can be greater and lead to more marked pain. Therefore, it is essential to drain and analyse this liquid to rule out an infectious or microcrystalline cause. These reactions usually resolve within a few days. Therapeutic benefit is still possible after such reactions. No cases of intra-articular infection occurred in the clinical trials of SYNVISC/SYNVISC-ONE; rare cases have been reported during post-marketing use of SYNVISC.

The following rare systemic reactions have been reported after the administration of SYNVISC: erythema, urticaria, itching, fever, nausea, headache, dizziness, chills, muscle cramps, paraesthesia, peripheral oedema, malaise, respiratory difficulties, hot flushes and facial oedema. Controlled clinical trials with SYNVISC found no significant statistical difference in the number or types of undesirable systemic effects between the group of patients treated with SYNVISC and the control group.

In the controlled trial with SYNVISC-ONE the frequency and the types of undesirable effects were similar in the group that received SYNVISC-ONE and the group that received a placebo.

STORAGE CONDITIONS:

Store between +2°C and +30°C.

Do not freeze.

Do not use if the package is opened or damaged.

Tariff in the LPPR [French list of products and services that can be reimbursed]

76.00 Euros code: LPPR 1130495 (Synvisc-one box of 1 syringe)

Social security reimbursement only for patients with osteoarthritis of the knee who have failed to respond to analgesics and who have failed to respond to or are intolerant to non-steroidal anti-inflammatory drugs (NSAIDs) within a maximum limit of:

One treatment consisting of a single injection per year and per knee (Synvisc-one, Official Journal of 22.01.09)

Coverage is subject to the intra-articular injection being prescribed and performed either by a rheumatologist, an orthopaedic surgeon or a physician specialising in physical and rehabilitation medicine.

CE marking 0088.

GENZYME SAS

33-35, boulevard de la Paix. 78105 Saint-Germain-en-Laye cedex

Information and ordering: higher rate tel. No.: 0825825861 Devices vigilance: higher rate tel. no: 0825801051 Medical information: higher rate tel. no: 08 25 80 14 03

(27) Reference: Vidal Compendium OCP [pharmacy wholesaler] ed, Paris 2010

ANNEX 1.15

PHARMACOVIGILANCE FORM AND SERIOUS ADVERSE EVENT (SAE) REPORT

**Date**<br>Day Month Year	**Descriptio n**	**Seriousness**<br><br>0 = Non-serious<br>1= Serious (a)	**Intensity**<br><br>0 = Mild<br>1 = Moderate<br>2 = Severe	**Start**<br><br>0 = Before the injection<br>1 = During or just after the injection<br>2 = After the injection (b)	**Duration**<br><br>Days Hours Minutes	**Causality**<br><br>0 = Excluded<br>1 = Unlikely<br>3 = Probable<br>4 = Certain<br>5 = Unknown/ not assessable	**Medical intervention**<br>0 = None<br>1 = Treatment (c)<br>2 = Possible	**Development**<br><br>0 = Recovery<br>1 = Ongoing<br>2= Hospitalisatio n	**Comments**<br><br>1= Trial continued<br>2 = Trial ended
Start date<br>|_|_| |_|_| |_|_| <br>End date<br>|_|_| |_|_| |_|_| <br>or ☐ ongoing					|_|_| |_|_| |_|_| (unless ongoing)				
Start date<br>|_|_| |_|_| |_|_| <br>End date<br>|_|_| |_|_| |_|_| <br>or ☐ ongoing					|_|_| |_|_| |_|_| (unless ongoing)				
Start date<br>|_|_| |_|_| |_|_| <br>End date<br>|_|_| |_|_| |_|_| <br>or ☐ ongoing					|_|_| |_|_| |_|_| (unless ongoing)				

(a) If serious, complete the SAE form (see protocol part II paragraph 2.5 for the definition of serious adverse event terms)

(b) Specify the number of days and hours if possible

(c) Description of treatment: ………………………………………………………………………………………

Serious Adverse Event (SAE) Report

Serious Adverse Event (SAE) Report **Page 1/3**

Fax this report __immediately__ or within 24 hours at the latest. Telephone the number below to confirm it has been received: TRB Chemedica International SA, Rue Michel-Servet 12, Case postale 352, 1211 Geneva 12, Switzerland.
Fax: +41 22 703 49 01. Tel.: +41 22 703 49 21 during office hours. Fax: +41 22 703 49 01 outside of office hours.
To: Dr My-Lam Nguyen

This box is for TRB Chemedica International SA staff	Case No.: …………………………..	Acknowledgement of receipt |_|_| |_|_| |_|_|_|_| day month year

Patient data

Patient No.:	Patient initials: ………………. surname/first name	Date of birth: ..../……../…… day/month/year	Pregnancy: ☐ ☐ ☐ yes no N/A	Sex: ☐ ☐ male female

Study data

Protocol No.:	Centre No.: …………………………………………………..	Country where the SAE occurred: ……………………………………………..

O	S	T	P	-	E	U	R	-	1	0	-	0	1

SAE report

Reporter	Name: ………………………………………………………………………………………........<br>Address: ………………………………………………………………………………………...<br>Fax No.: ………………………………………………………………………………………<br>Tel. No.: ………………………………………………………………………………………

SAE criteria

Select all boxes that apply	☐ death<br>☐ in-patient hospitalisation or prolongation of existing hospitalisation<br>☐ persistent or significant disability<br>☐ life threatening<br>☐ congenital anomaly/birth defect<br>☐ other medically important event<br>Please specify: ………………………………………………………………………………

SAE details

Diagnosis/diagnoses (if no diagnosis is available, specify the main symptoms)	*__Please complete in order of importance__*		
	1<br>……….................................<br>……….................................<br>………..................................	2<br>……….................................<br>……….................................<br>………..................................	3<br>……….................................<br>……….................................<br>………..................................
Onset date and time	|_|_| |_|_| |_|_|_|_| day month year<br>|_|_| : |_|_| hours min.	|_|_| |_|_| |_|_|_|_| day month year<br>|_|_| : |_|_| hours min.	|_|_| |_|_| |_|_|_|_| day month year<br>|_|_| : |_|_| hours min.
End date and time (select the "ongoing" box where appropriate)	|_|_| |_|_| |_|_|_|_| day month year<br>|_|_| : |_|_| hours min.<br>☐ ongoing	|_|_| |_|_| |_|_|_|_| day month year<br>|_|_| : |_|_| hours min.<br>☐ ongoing	|_|_| |_|_| |_|_|_|_| day month year<br>|_|_| : |_|_| hours min.<br>☐ ongoing
Maximum intensity	☐ mild<br>☐ moderate<br>☐ severe	☐ mild<br>☐ moderate<br>☐ severe	☐ mild<br>☐ moderate<br>☐ severe

Serious Adverse Event (SAE) Report Page 2/3

<table>
<tr><td colspan="4">Protocol No.</td><td colspan="4">Site No.</td><td colspan="2">Patient No.</td><td colspan="5">Patient initials:
……………….
Initials: surname/first name</td></tr>
<tr><td>O</td><td>S</td><td>T</td><td>P</td><td>-</td><td>E</td><td>U</td><td>R</td><td>-</td><td>1</td><td>0</td><td>-</td><td>0</td><td>1</td><td></td></tr>
</table>

<table>
<tr><td>Causality</td><td>☐ Excluded
☐ Unlikely
☐ Possible
☐ Probable
☐ Certain
☐ Unknown/not assessable</td><td>☐ Excluded
☐ Unlikely
☐ Possible
☐ Probable
☐ Certain
☐ Unknown/not assessable</td><td>☐ Excluded
☐ Unlikely
☐ Possible
☐ Probable
☐ Certain
☐ Unknown/not assessable</td></tr>
<tr><td colspan="4">Description of the SAE, including the conduct of the event, and comments</td></tr>
<tr><td colspan="4">Specify relevant information about the diagnosis, causality assessment, dosage of the drug studied, relevant laboratory results, etc.
………..….
……..…………</td></tr>
</table>

<table>
<tr><td colspan="3">Administration of the study drug</td></tr>
<tr><td>Study drug</td><td colspan="2">Indication: ………………………………………………………………………………….……….</td></tr>
<tr><td>“Code broken” (sealed envelope opened)</td><td colspan="2">☐ Yes -> |_|_| |_|_| |_|_|_|_|
☐ No day month year
☐ N/A</td></tr>
<tr><td>Study drug</td><td colspan="2">If the study is open or if the code has been broken, please specify: ………..……………....
Route: …………………………….….…. Daily dose: ……………..……………..………</td></tr>
<tr><td>First administration of the study drug</td><td colspan="2">|_|_| |_|_| |_|_|_|_| |_|_| : |_|_|
day month year hours min.</td></tr>
<tr><td>Last administration of the study drug before the SAE</td><td colspan="2">|_|_| |_|_| |_|_|_|_| |_|_| : |_|_|
day month year hours min.</td></tr>
<tr><td>Action taken regarding the SAE of the study drug</td><td>☐ None
☐ Treatment temporarily discontinued then resumed
☐ Dose reduced
☐ Dose increased
☐ Treatment stopped</td><td>Comments (durations, dosage changes etc.)
………………………………………….
…………………………………..……..
…………………………………..……..
……………………………………..…..</td></tr>
<tr><td>Has this event occurred previously?</td><td colspan="2">☐ Yes ☐ No</td></tr>
<tr><td>Did the SAE disappear when the drug was stopped?</td><td colspan="2">☐ Yes ☐ No ☐ N/A</td></tr>
<tr><td>Did the SAE reappear following re-introduction of the product?</td><td colspan="2">☐ Yes ☐ No ☐ N/A</td></tr>
</table>

<table>
<tr><td colspan="6">Concomitant treatments</td></tr>
<tr><td>Generic name (brand name for combined drugs)</td><td>Indication</td><td>Total daily dose</td><td>Route</td><td>First administration</td><td>Last administration</td></tr>
<tr><td></td><td></td><td></td><td></td><td>|_|_| |_|_| |_|_|_|_|
day month year</td><td>|_|_| |_|_| |_|_|_|_|
day month year
☐ ongoing</td></tr>
<tr><td></td><td></td><td></td><td></td><td>|_|_| |_|_| |_|_|_|_|
day month year</td><td>|_|_| |_|_| |_|_|_|_|
day month year
☐ ongoing</td></tr>
</table>

**Serious Adverse Event (SAE) Report**			**Page 3/3**
Protocol No.	Site No.	Patient No.	Patient initials: ………………<br>Initials: surname/first name
**O S T P - E U R - 1 0 - 0 1**			

**SAE specific treatment (drugs, procedures)**	
Specific treatment for this SAE	☐ None<br>☐ Drug therapy ⌦ specify below<br>☐ Non-drug therapy ⌦ specify below<br>…………………………………………………………………………………..……………<br>…………………………………………………………………………………..……………

**Outcome**	
Select the appropriate box	☐ Recovered<br>☐ Recovery ongoing<br>☐ No recovery/ongoing ⌦ **further information should be provided as soon as possible**<br>☐ Recovered with sequelae ⌦ please specify<br>……………………………………………………………………………………….…<br>……………………………………………………………………………………..………<br>☐ Patient died/⌦ date of death |_|_| |_|_| |_|_|_|_| day month year<br>☐ Unknown
In the event of death	Cause of death<br>☐ Progression of the disease being studied<br>☐ SAE<br>☐ Other<br><br>Details on the cause of death<br>……………………………………………………………………………………….<br>……………………………………………………………………………………….<br><br>Yes No<br>Autopsy ☐ ☐<br>Autopsy report available ☐ ☐

**Patient history**			
History, concomitant diseases, allergies, major risk factors, laboratory findings, surgical procedures, biopsies, unusual circumstances, differential diagnosis, etc.)<br>………<br>………<br>………			
Type of report:	initial ☐	follow-up ☐	No. ………………
………………….<br>Date	………………………………………..……………………………..…………………..<br>Signature of the investigator (attesting that the information provided is accurate)		

ANNEX 1.16

ACKNOWLEDGEMENT OF RECEIPT

ACKNOWLEDGMENT OF RECEIPT BY EVALUATING DOCTOR

CLINICAL TRIAL EVALUATING THE EFFICACY OF OSTENIL PLUS (HYALURONIC ACID) VERSUS SYNVISC-ONE IN PATIENTS WITH TIBIOFEMORAL OSTEOARTHRITIS

I, the undersigned Doctor ______________________________, herby certify that I have received today:

☑ 1 Scientific information brochure
☑ 1 Protocol
☑ 4 CRFs
☑ 4 Patient diaries
☑ 4 Informed consent forms
☑ 4 Patient information leaflet
☑ 2 Investigator financial agreements
☑ 1 Confidentiality undertaking and protocol approval signature sheet
☑ 4 Randomisation envelopes |_|_|_| |_|_|_| |_|_|_| |_|_|_|

<u>The aforementioned are:</u>
☑ In good condition
☐ Damaged
Comments: ____

____

Date |_|_| |_|_| |_|_|_|_| Investigator's signature

ACKNOWLEDGMENT OF RECEIPT BY INJECTING INVESTIGATOR

CLINICAL TRIAL EVALUATING THE EFFICACY OF OSTENIL PLUS (HYALURONIC ACID) VERSUS SYNVISC-ONE IN PATIENTS WITH TIBIOFEMORAL OSTEOARTHRITIS

I, the undersigned Doctor ______________________________, herby certify that I have received today:

☑ 1 Scientific information brochure
☑ 1 Protocol
☑ 4 CRFs
☑ 2 Investigator financial agreements
☑ 1 Confidentiality undertaking and protocol approval signature sheet
☑ 4 Drug batches |_|_|_| |_|_|_| |_|_|_| |_|_|_|
☑ 4 Randomisation envelopes |_|_|_| |_|_|_| |_|_|_| |_|_|_|

<u>The aforementioned are:</u>
☑ In good condition
☐ Damaged
Comments: ____

____

Date |_|_| |_|_| |_|_|_|_| Investigator's signature

ANNEX 1.17

CONFIDENTIALITY UNDERTAKING AND PROTOCOL APPROVAL

CONFIDENTIALITY UNDERTAKING AND PROTOCOL APPROVAL

CLINICAL TRIAL EVALUATING THE EFFICACY OF OSTENIL PLUS (HYALURONIC ACID) VERSUS SYNVISC-ONE IN PATIENTS WITH TIBIOFEMORAL OSTEOARTHRITIS

I, the undersigned Doctor ____ herby confirm that I have read the scientific protocol and have taken note of the directives it contains for the conduct of the above-mentioned study.

I hereby undertake to respect the protocol and the confidentiality of the study.

Date |_|_| |_|_| |_|_|_|_| Investigator's signature

ANNEX 2.1
DECLARATION OF HELSINKI

WORLD MEDICAL ASSOCIATION DECLARATION OF HELSINKI

Ethical Principles for Medical Research Involving Human Subjects

Adopted by the 18th WMA General Assembly,
Helsinki, Finland, June 1964,
and amended by the:

29th WMA General Assembly,
Tokyo, Japan, October 1975

35th WMA General Assembly,
Venice, Italy, October 1983

41st WMA General Assembly,
Hong Kong, September 1989

48th WMA General Assembly,
Somerset West, Republic of South Africa, October 1996

52nd General Assembly, Edinburgh, Scotland, October 2000

WMA General Assembly, Washington 2002
(Note of Clarification on paragraph 29 added)

WMA General Assembly, Tokyo 2004
(Note of Clarification on Paragraph 30 added)

59th WMA General Assembly, Seoul, Korea, October 2008

Introduction

The World Medical Association has developed the **Declaration of Helsinki** as a statement of ethical principles to provide guidance to physicians and other participants in medical research involving human subjects. Medical research involving human subjects includes research on identifiable human material or identifiable data.

It is the duty of the physician to promote and safeguard the health of the people. The physician's knowledge and conscience are dedicated to the fulfilment of this duty.

The Declaration of Geneva of the World Medical Association binds the physician with the words, *'The health of my patient will be my first consideration'* and the International Code of Medical Ethics declares that *'A physician shall act only in the patient's interest when providing medical care which might have the effect of weakening the physical and mental condition of the patient.'*

Medical progress is based on research which ultimately must rest in part on experimentation involving human subjects.

In medical research on human subjects, considerations related to the well-being of the human subject should take precedence over the interests of science and society.

The primary purpose of medical research involving human subjects is to improve prophylactic, diagnostic and therapeutic procedures and the understanding of the aetiology and pathogenesis of disease. Even the best proven prophylactic, diagnostic and therapeutic methods must continuously be challenged through research for their effectiveness, efficiency and accessibility.

In current medical practice and in medical research, most prophylactic, diagnostic and therapeutic procedures involve risks and burdens.

Medical research is subject to ethical standards that promote respect for all human beings and protect their health and rights. Some research populations are vulnerable and need special protection. The particular needs of the economically and medically disadvantaged must be recognised. Special attention is also required for those who cannot give or refuse consent for themselves, for those who may be subject to giving consent under duress, for those who will not benefit personally from the research and for those for whom the research is combined with care.

Research investigators should be aware of the ethical, legal and regulatory requirements for research on human subjects in their own countries as well as applicable international requirements. No national ethical, legal or regulatory requirement should be allowed to reduce or eliminate any of the protections for human subjects set forth in this Declaration.

Basic principles for all medical research

It is the duty of the physician in medical research to protect the life, health, privacy and dignity of the human subject.

Medical research involving human subjects must conform to generally accepted scientific principles, be based on a thorough knowledge of the scientific literature, other relevant sources of information and on adequate laboratory and, where appropriate, animal experimentation.

Appropriate caution must be exercised in the conduct of research which may affect the environment and the welfare of animals used for research must be respected.

The design and conduct of each experimental procedure involving human subjects should be clearly formulated in an experimental protocol. This protocol should be submitted for consideration, comment, guidance, and where appropriate, approval to a specially appointed ethical review committee, which must be independent of the investigator, the sponsor or any other kind of undue influence. This independent committee should be in conformity with the laws and regulations of the country in which the research experiment is performed. The committee has the right to monitor ongoing trials. The researcher has the obligation to provide monitoring information to the committee, especially any serious adverse events. The researcher should also submit to the committee, for review, information regarding funding, sponsors, institutional affiliations, other potential conflicts of interest and incentives for subjects.

The research protocol should always contain a statement of the ethical considerations involved and should indicate that there is compliance with the principles enunciated in this Declaration.

Medical research involving human subjects should be conducted only by scientifically qualified persons and under the supervision of a clinically competent medical person. The responsibility for the human subject must always rest with a medically qualified person and never rest on the subject of the research, even though the subject has given consent.

Every medical research project involving human subjects should be preceded by careful assessment of predictable risks and burdens in comparison with foreseeable benefits to the subject or to others. This does not preclude the participation of healthy volunteers in medical research. The design of all studies should be publicly available.

Physicians should abstain from engaging in research projects involving human subjects unless they are confident that the risks involved have been adequately assessed and can be satisfactorily managed. Physicians should cease any investigation if the risks are found to outweigh the potential benefits or if there is conclusive evidence of positive and beneficial results.

Medical research involving human subjects should only be conducted if the importance of the objective outweighs the inherent risks and burdens to the subject. This is especially important when the human subjects are healthy volunteers.

Medical research is only justified if there is a reasonable likelihood that the populations in which the research is carried out stand to benefit from the results of the research.

The subjects must be volunteers and informed participants in the research project.

The right of research subjects to safeguard their integrity must always be respected. Every precaution should be taken to respect the privacy of the subject, the confidentiality of the patient's information and to minimise the impact of the study on the subject's physical and mental integrity.

In any research on human beings, each potential subject must be adequately informed of the aims, methods, sources of funding, any possible conflicts of interest, institutional affiliations of the researcher, the anticipated benefits and potential risks of the study and the discomfort it may entail. The subject should be informed of the right to abstain from participation in the study or to withdraw consent to participate at any time without reprisal. After ensuring that the subject has understood the information, the physician should then obtain the subject's freely-given informed consent, preferably in writing. If the consent cannot be obtained in writing, the non-written consent must be formally documented and witnessed.

When obtaining informed consent for the research project the physician should be particularly cautious if the subject is in a dependent relationship with the physician or may consent under duress. In that case the informed consent should be obtained by a well-informed physician who is not engaged in the investigation and who is completely independent of this relationship.

For a research subject who is legally incompetent, physically or mentally incapable of giving consent or is a legally incompetent minor, the investigator must obtain informed consent from the legally authorised representative in accordance with applicable law. These groups should not be included in research unless the research is necessary to promote the health of the population represented and this research cannot instead be performed on legally competent persons.

When a subject deemed legally incompetent, such as a minor child, is able to give assent to decisions about participation in research, the investigator must obtain that assent in addition to the consent of the legally authorised representative.

Research on individuals from whom it is not possible to obtain consent, including proxy or advance consent, should be done only if the physical/mental condition that prevents obtaining informed consent is a necessary characteristic of the research population. The specific reasons for involving research subjects with a condition that renders them unable to give informed consent should be stated in the experimental protocol for consideration and approval of the review committee. The protocol should state that consent to remain in the research should be obtained as soon as possible from the individual or a legally authorised surrogate.

Both authors and publishers have ethical obligations. In publication of the results of research, the investigators are obliged to preserve the accuracy of the results. Negative as well as positive results should be published or otherwise publicly available. Sources of funding, institutional affiliations and any possible conflicts of interest should be declared in the publication. Reports of experimentation not in accordance with the principles laid down in this Declaration should not be accepted for publication.

Additional principles for medical research combined with medical care

The physician may combine medical research with medical care, only to the extent that the research is justified by its potential prophylactic, diagnostic or therapeutic value. When medical research is combined with medical care, additional standards apply to protect the patients who are research subjects.

The benefits, risks, burdens and effectiveness of a new method should be tested against those of the best current prophylactic, diagnostic and therapeutic methods. This does not exclude the use of placebo, or no treatment, in studies where no proven prophylactic, diagnostic or therapeutic method exists.

To clarify the WMA position on the use of placebo-controlled trials, the WMA Council issued, during October 2001, a note of clarification which can be found on this page.

At the conclusion of the study, every patient entered into the study should be assured of access to the best proven prophylactic, diagnostic and therapeutic methods identified by the study. (Note of clarification).

The investigator should fully inform the patient which aspects of the care are related to the research. The refusal of a patient to participate in a study must never interfere with the patient–physician relationship.

In the treatment of a patient, where proven prophylactic, diagnostic and therapeutic methods do not exist or have been ineffective, the physician, with informed consent from the patient, must be free to use unproven or new prophylactic, diagnostic and therapeutic measures, if in the physician’s judgement it offers hope of saving life, re-establishing health or alleviating suffering. Where possible, these measures should be made the object of research, designed to evaluate their safety and efficacy. In all cases new information should be recorded and, where appropriate, published. The other relevant guidelines of this Declaration should be followed.

Note of clarification on paragraph 29

The WMA is concerned that paragraph 29 of the Declaration of Helsinki (October 2000) has led to diverse interpretations and possible confusion. It hereby affirms its position that extreme care must be taken in making use of a placebo-controlled trial and that in general this methodology should only be used in the absence of existing proven therapy. However, a placebo-controlled trial may be ethically acceptable, even if proven therapy is available, under the following circumstances:

- where for compelling and scientifically sound methodological reasons its use is necessary to determine the efficacy or safety of a prophylactic, diagnostic or therapeutic method; or
- where a prophylactic, diagnostic or therapeutic method is being investigated for a minor condition and the patients who receive placebo will not be subject to any additional risk of significant or irreversible harm.

All other provisions of the Declaration of Helsinki must be adhered to, especially the need for appropriate ethical and scientific review.

Note of clarification on paragraph 30

The WMA hereby reaffirms its position that it is necessary during the study planning process to identify post-trial access by study participants to prophylactic, diagnostic and therapeutic procedures identified as beneficial in the study or access to other appropriate care. Post-trial access arrangements or other care must be described in the study protocol so the ethical review committee may consider such arrangements during its review.

ANNEX 2.2
HURIET LAW

Ministry of Solidarity, Health
and Social Protection

HURIET LAW

Legal provisions

(Law No. 88-1138 of 20 December 1988, published in the French Official Gazette, amended by Law No. 90-86 of 23 January 1990, amended by Law No. 94-630 of 25 July 1994)

PROTECTION OF PERSONS PARTICIPATING
IN BIOMEDICAL RESEARCH

Article L.209-1
Clinical trials or experiments organised and conducted on human subjects with the objective of developing medical or biological knowledge are hereby authorised under the conditions prescribed in this book and are referred to herein by the term: ‘biomedical research’.
Biomedical research where the person participating in the research is expected to directly benefit is referred to as biomedical research ‘with direct individual benefit’. All other research, whether concerning people who are ill or not, is referred to as ‘without direct individual benefit’. The natural or legal person responsible for initiating the biomedical research on human subjects is hereinafter referred to as the sponsor. The individual(s) who direct(s) and supervise(s) the conduct of the research are hereinafter referred to as the investigators.
When several people are responsible for initiating the same research, they may designate a natural or legal person to act as the sponsor and they shall undertake the corresponding obligations pursuant to this book.
If the research sponsor chooses several investigators, it must designate a co-ordinating investigator from among them.

I - GENERAL PROVISIONS

Article L.209-2
Biomedical research cannot be carried out on human subjects:
- If it is not based on the latest state of scientific knowledge and sufficient preclinical testing
- If the foreseeable risk to the persons participating in the research is ‘out of proportion’ to the expected benefit for these persons or the interest of this research
- If it does not aim to broaden scientific knowledge and the methods likely to improve their condition

Article L.209-3
Biomedical research can only be conducted:
- Under the direction and supervision of a physician with appropriate experience
- Within a physical and technical setting which is suitable for the trial and compatible with proper scientific rigour and safety for the research participants.

Dentistry-related biomedical research may only be conducted under the direction and supervision of a dental surgeon and physician with appropriate experience.
Where human behavioural sciences are concerned, a qualified person, together with the investigator, may lead the research.

Article L.209-4
Research without direct individual benefit on pregnant women, women in labour and nursing mothers shall only be permitted if it does not present any predictable risks to the health of the woman or child, if it is relevant to knowledge of phenomena associated with pregnancy or breast-feeding and if it cannot be conducted otherwise.

Article L.209-5
Persons deprived of their liberty by judicial or administrative decision, patients in emergency situations and patients hospitalised without their consent under articles L.333 and L.342 who are not protected by the law can only be asked to participate in biomedical research if a direct and significant benefit to their health is expected.

Article L.209-6
Minors, adults subject to guardianship and persons staying in a health or welfare establishment for reasons other than research, may not be asked to participate in biomedical research unless direct benefit to their health is expected.
However, research without direct individual benefit is permitted if the following three conditions are met:
- There is no serious predictable risk to their health
- It is of benefit to persons displaying the same characteristics of age, disease or disability
- It cannot be conducted otherwise.

Article L.209-7
For biomedical research without direct individual benefit, the sponsor is liable, even without fault, for the compensation of any harmful effects of the research on the subject and their beneficiaries; this shall not be binding upon any third party or in the case of voluntary withdrawal of a person who initially consented to participating in the research.
For biomedical research with direct individual benefit, the sponsor is liable for the compensation of any harmful effects of the research on the subject and their beneficiaries, unless it can be proven that the sponsor is not responsible for the harm caused, and nor is the subject; this shall not be binding upon any third party or in the case of voluntary withdrawal of a person who initially consented to participating in the research.
Biomedical research requires the sponsor to take out insurance guaranteeing its civil liability as set out in this article and that of all participants, irrespective of the nature of the relationship between the participants and the sponsor. The provisions of this article are mandatory.

Article L.209-8
Biomedical research participants should not receive any direct or indirect financial gain over and above the reimbursement of expenses incurred, subject to the specific conditions provided for in article L.209-15 of this Code regarding research without direct individual benefit.

II - CONSENT

Article L.209-9

Prior to biomedical research being conducted on any person, free, informed and expressed consent must be obtained from that person after the investigator or physician designated to represent him/her has informed the person of the following:

- The research objective, methodology and duration
- The anticipated benefits, the limitations and risks associated, including in the case of early termination of the research
- The opinion of the Committee mentioned in article L.209-12 of this Code
- Where appropriate, its entry in the national register specified in article L.209-17.

The investigator or physician shall inform the person from whom the consent is sought of their right to refuse to participate in the research or to withdraw their consent at any time without reprisal.

The objectives of psychological research, as well as its methodology and duration may be provided as brief preliminary information provided that the research involves only healthy volunteers and there is no serious predictable risk. Comprehensive information on this research is provided to participants at the end of the research.

The project referred to in the first paragraph of article L.209-12 specifies the nature of the prior information provided to the participants.

In exceptional circumstances, when in the interests of a patient, the diagnosis of their illness has not been made known to them, the investigator may, in accordance the patient's beliefs, withhold some of the information regarding this diagnosis. In this case, the research protocol must specify this eventuality. The information provided is summarised in a written document given to the person whose consent is requested.

Consent is provided in writing or, where this is impossible, attested by a third party. The latter must be fully independent of the investigator and the sponsor.

However, in the event of biomedical research implemented in an emergency, thereby preventing the participant from giving prior consent, the protocol submitted to the committee established under article L.209-11 of this code may provide that the person's consent will not be sought and that the consent of family members if they are present will suffice, under the conditions specified above. The person concerned will be informed as soon as possible and consent requested for possible continuation of the research.

Article L.209-10

If biomedical research is conducted on minors or adults under guardianship:

- Consent shall be given in accordance with the provisions of article L.209-9 of this Code by the persons entitled to exercise parental authority on behalf of unemancipated minors. In the case of minors or adults under guardianship, consent may be given by the guardian for research with direct individual benefit not involving any serious predictable risk and in other cases, by the guardian authorised by the Family Council or the judge supervising guardianship.
- The consent of a minor or an adult under guardianship shall also be sought if they are capable of exercising judgment. Their refusal or revocation of consent may not be disregarded.

III - ADMINISTRATIVE PROVISIONS

Article L.209-11

In each region, the Minister of Health approves one, or depending on requirements, several CCPPRB (*Comité consultatif de protection des personnes dans la recherche biomédicale* – Consultative Committee for the Protection of Persons in Biomedical Research).
The Minister sets out the number of committees in each region by ministerial decree. The territorial jurisdiction of a committee may extend to several regions.
The Committees are totally independent in the exercise of their mission. They are legal entities in their own right.

The committees have jurisdiction within the region where they have their headquarters. A Council of State decree sets out the minimum conditions of activity below which the territorial jurisdiction of a committee may be extended to several regions.
The Committees are formed in such a way as to ensure their independence and the diversity of competencies in the biomedical domain and with regard to ethical, social, psychological and legal questions.
Their members are nominated by the State representative in the region where the committee has its headquarters. They are chosen from the persons on the list drawn up based on a proposal from the organisations or authorities which are authorised to do so under the conditions set out in the decree.
Members of the Committees, persons called upon to collaborate in their work, agents coming under the general status of public servants and hospital public service agents coming under law 86-33 of 9 January 1986 containing statutory provisions relating to hospital public service are legally bound, under the conditions and the penalties provided for in article 378 of the French Penal Code, to keep secret any information which comes to their knowledge by reason of their function and which is related to the nature of the research, to the persons who organise it or who participate in it, or to products, objects or experimental methods.
Persons who are not independent of the sponsor and the investigator in the research project under examination may not legitimately take part in the deliberations.
The operating costs of the Committees are financed by the proceeds from a fixed fee paid by the sponsors for each biomedical research project that is the subject of a request for an opinion. The amount of this fee is fixed by the Minister of Health.
The Minister of Health may withdraw the registration of the Committee if the conditions of independence, of formation or of operation required for fulfilling its mission are no longer satisfied.

Article L.209-12

Before conducting research on human subjects, all investigators must submit the research project to the CCPPRB responsible for the region where they are working. Only one opinion can be requested per research project.
In the case of research performed by several investigators, the co-ordinating investigator shall request the opinion and submit the project under the conditions set out in the first paragraph of the first article.
The committee delivers its opinion on the conditions required to ensure the validity of the research with regards to the protection of persons, notably the protection of the participants, their information before and during the research, the methods of obtaining consent, any possible compensation, the general relevance of the project and whether the available means and the qualifications of the investigators involved are appropriate to meet the aims. The committee must make its opinion known in writing to the investigator within five weeks. The competent authority must be informed if a research project receives an unfavourable opinion.

Before commencing the research, the sponsor shall submit a letter of intent to the competent authority describing the key research data along with the opinion of the committee.
This opinion does not relieve the sponsor of its responsibility. Projects which have received an unfavourable opinion cannot be implemented until two months have elapsed from the date of receipt by the competent authority
When the research is to be conducted in one or more public or private establishments, the sponsor shall inform the director(s) of the establishments prior to the implementation of the research.

The sponsor shall inform the competent authority as soon as it becomes aware of any effect which may have contributed to the occurrence of a death, caused hospitalisation or lead to any lasting organic or functional effects which are likely to be due to the research. The sponsor shall also provide the competent administrative authority with any information relating to the occurrence of any new event concerning the conduct of the research or the development of the product or device undergoing research where the new event is likely to affect the safety of the participants. Finally, the sponsor shall inform the committee if the research is terminated early, explaining the reason why.
The competent administrative authority can, at any time, request additional information on the research from the sponsor. If the sponsor does not respond, there is a risk to public health or the provisions of this book are not complied with, the competent administrative authority may also, at any time, suspend or prohibit the biomedical research.

Article 209-12-1
The CCP (*Comité consultatif de protection des personnes* – Consultative Committee for the Protection of Persons) may issue a favourable opinion under the conditions provided for in article L.209-12 subject to the investigator submitting additional information during the conduct of the research.
Following this submission, the committee may maintain or amend its opinion. This decision is sent in writing to the investigator within five weeks; it is then sent by the sponsor to the competent administrative authority within one week from receipt.

Article L.209-13
Medical inspectors and, within the scope of their own remit, inspectors from the medicines agency, have the authority to ensure compliance with the provisions of this book and its enabling legislation.

Article L.209-13-1
The consultation procedures for the CCPPRBs regarding research projects of a military nature are set out by decree of the Council of State.

IV – SPECIFIC PROVISIONS REGARDING RESEARCH WITHOUT DIRECT INDIVIDUAL BENEFIT

Article L.209-14
Biomedical research without direct individual benefit should not carry any serious predictable risk for the health of the participants.
Potential candidates must undergo medical examination. The results of this examination shall be communicated to them by the physician of their choice, prior to them giving their consent.

Article L.209-15
In the case of biomedical research without direct individual benefit for the participant, the sponsor may pay compensation to such persons for the constraints they have faced. The total sum of compensation an individual can receive in any given year is limited to a maximum set by the Minister of Health.
Payment of the compensation provided for in the first paragraph of this article is prohibited if the research involves minors, adults under guardianship or persons staying in a health or welfare establishment for purposes other than research.

Article L.209-16
Any biomedical research without direct individual benefit on a person who is not registered with a social security scheme or is not a beneficiary of such a scheme is prohibited.
The social security body shall bring an action against the sponsor for the payment of services paid or provided.

Article L.209-17
No person may simultaneously volunteer for several biomedical research projects without direct individual benefit.
For each research project without direct individual benefit, the protocol submitted for the advisory opinion of the CCPPRB establishes an exclusion period during which a person who volunteers for research may not participate in other research without direct therapeutic aims. The length of this period varies depending on the nature of the research.
In order to apply the above provisions, the Minister of Health shall establish and maintain a national register.

Article L.209-18
Biomedical research without direct individual benefit may only take place in a location equipped with the physical and technical means which are suitable for the research and compatible with the safety requirements for the participants, as authorised by the Minister of Health.

Article L.209-18-1
No biomedical research can be carried out on a person who is brain dead without their directly expressed consent or the testimony of their family.
The provisions of article 225-17 of the Penal Code do not apply to such research.

V - CRIMINAL SANCTIONS

Article L.209-19
As stated in article 223-8 of the French Penal Code, conducting or causing biomedical research to be conducted on a person without having obtained the free, informed and explicit consent of the person concerned, or of the persons entitled to exercise parental authority on their behalf, or of their guardian in the cases provided for under the provisions of the Code of Public Health, is punishable by three years' imprisonment and a fine of 300,000 French francs.
The same penalties apply if consent is withdrawn before the biomedical research is conducted.
As stated in article 223-9 of the French Penal Code, legal persons may incur criminal liability for this offence pursuant to the conditions set out under article 121-2 of the French Penal Code.
The penalties incurred by legal persons are:
A fine, pursuant to the conditions set out under article 131-38 of the French Penal Code
The penalties enumerated under article 131-39 of the French Penal Code. The prohibition mentioned under 2° of article 131-39 of the French Penal Code applies to the activity in the exercise of which, or on the occasion of which, the offence was committed.

Article L.209-19-1
Conducting or causing biomedical research to be conducted in breach of the provisions of articles L.209-4 to L.209-6 and the last paragraph of article L.209-9 is punishable by 3 years' imprisonment and a fine of 300,000 French francs.
Natural persons convicted of the offence mentioned in the preceding paragraph shall also incur the following penalties:
Forfeiture of civic, civil and family rights, pursuant to the conditions set out under article 131-26 of the French Penal Code

Prohibition to discharge the social or professional activity in the exercise of which, or on the occasion of which, the offence was committed
Confiscation as defined under article 131-21 of the French Penal Code
Exclusion from public tenders, either permanently or for a period of up to five years
Legal persons may incur criminal liability for the offence defined in the first paragraph, pursuant to the conditions set out under article 121-2 of the French Penal Code.
The penalties incurred by legal persons are:
A fine, pursuant to the conditions set out under article 131-38 of the French Penal Code
The penalties enumerated under article 131-39 of the French Penal Code
The prohibition mentioned under 2° of article 131-39 of the French Penal Code applies to the activity in the exercise of which, or on the occasion of which, the offence was committed.

Article L.209-20
One year's imprisonment and a fine of 100,000 French francs is the punishment given to the following:
- Anyone who has conducted or caused biomedical research to be conducted without having obtained the prior opinion provided for in article L.209-12 of this Code
- Anyone who has conducted or caused biomedical research to be conducted in contravention of the provisions of the first two paragraphs of article L.209-17 of this code
- Anyone who has conducted or caused biomedical research to be conducted, or who has continued to conduct or to cause biomedical research to be conducted which has been prohibited or suspended by the Minister of Health.

An investigator conducting such research in breach of the provisions of article L.209-18 shall be subject to the same penalties.

Article L.209-21
Any sponsor whose third-party liability is not covered by the insurance set out in article L.209-7 of this Code shall be sentenced to one year's imprisonment and a fine of 100,000 French francs.
Any sponsor conducting or causing biomedical research to be conducted without having submitted the letter of intent provided for under article L.209-12 to the Health Minister shall be subject to the same penalties.

VI - MISCELLANEOUS PROVISIONS

Article L.209-22
By way of derogation from article 13 of the Judicature Act of 16-24 August 1790, the high court is the only competent court which can adjudicate on any action for compensation for damages arising from biomedical research; this action is set out in the conditions provided for in article 2270-1 of the French Civil Code.

Article L.209-23
The provisions of this book apply in the territorial collectivities of Saint-Pierre and Miquelon and Mayotte.

ANNEX 2.3
IMPLEMENTING CIRCULAR ON DMOS
(*Diverses Mesures d'Ordre Social* – French Anti-Gift Law or French DMOS Law)

FRENCH OFFICIAL GAZETTE – 6 AUGUST 1993

MINISTRY OF SOCIAL, HEALTH AND URBAN AFFAIRS

HEALTH

Circular dated 9 July 1993 on the application of article L.365-1 of the French Public Health Code.

NOR: SANP3301911C

Paris, 9 July 1993

The Minister of State, the Minister of Social, Health and Urban Affairs, the Minister of Economy and the Minister of Health to the regional prefects (Regional Directorate for Health and Social Affairs for the attention of the Regional Public Health Medical Inspectors and the Regional Directorates for Competition Policy, Consumer Affairs and Fraud Control).

Article 47 of the French Law dated 27 January 1993 containing various social measures introduced article L.365-1 to the French Public Health Code. The purpose of this text is to ensure greater transparency in relationships between healthcare professionals and companies in this sector supplying services or manufacturing or marketing products, where the cost of the services or products is reimbursed by compulsory social insurance schemes. It is not intended to prevent research or scientific assessment activities, such as continuing medical education, as these activities should always be subject to standard procedure. The questions raised by this text lead us to provide several clarifications on its meaning and scope in order to guide the activities of public health inspectors and officials of the Directorate General for Competition Policy and Fraud Control responsible for ensuring compliance with legal requirements.

I. The principle of prohibiting benefits in kind or pecuniary benefits

a) The meaning of the prohibition
Professionals, when choosing medicinal products, equipment or services where the cost is reimbursed by compulsory social insurance schemes, must only be guided by exclusively medical considerations. This ethical principle is expressed in the ethics codes (articles 9, 10 and 23 of the Code of Medical Ethics, article 6, final paragraph of the Code of Medical Ethics for Dental Surgeons and article 7 of the Code of Medical Ethics for Midwives).
Even before the enactment of article L.365-1, similar provisions existed in our internal law (articles L.549 and R.5064-1 of the French Public Health Code).
At European level, Directive No 92-28 of 31 March 1992 on the promotion of medicinal products for human use also introduced regulation of this activity.

It is in light of these principles that article L.365-1 of the French Public Health Code should be interpreted.

b) Scope of the prohibition

1 – Healthcare professionals targeted by the prohibition

The prohibition covers the medical professions identified in Title 1 of Book IV of the French Public Health Code (physicians, dental surgeons, midwives) and the professions identified in Title II, Chapter 1 of Title III and Title III-1 of Book IV of the Code (nurses, masseur-physiotherapists, speech therapists, orthoptists).

2 – Companies targeted by the prohibition

The scope of article L.365-1 is, in this respect, very broad since it deals with 'all companies supplying services or manufacturing or marketing products, where the cost of the services or products is reimbursed by compulsory social insurance schemes'. This includes not only companies manufacturing or marketing pharmaceutical products, but also those manufacturing or marketing medical and surgical equipment such as inert internal prostheses, pacemakers and intraocular implants. Also falling within the scope of article L.365-1 are communications companies or any other organisation acting on behalf of the companies which themselves fall under its remit.

3 – Companies not targeted by the prohibition on receiving benefits

Organisations made up of healthcare professionals, such as associations, trade unions, training insurance funds or learned societies, which receive benefits in kind or pecuniary benefits from the companies specified in point 2 above are not affected by the prohibition on receiving benefits.

The same applies to associations in the public or private not-for profit hospital sector formed for the purpose of raising funds or donations in kind aimed at providing support for the activities of one or more hospital services.

In order to circumvent the prohibition provided for in article L.365-1, the benefits in kind or pecuniary benefits received by the groups listed above must, in all cases, be used exclusively for collective purposes.

4 – Temporal application of the legal provision

The provisions of article 47 of the law of 27 January 1993 shall apply from 1 February 1993. The prohibition provided for in the first paragraph of article L.365-1 therefore applies to all benefits obtained from this date. Benefits considered to be lawful under the second paragraph of article L.365-1 obtained after 1 February 1993 shall be the subject of an agreement submitted for opinion to the departmental board of the relevant professional association before being implemented.

However, of course, the officials responsible for verifying the conditions for applying article L.365-1 shall take into account the time required to inform the parties concerned about this legislative mechanism. Audits shall focus on practices implemented after this circular is released.

c) Prohibited benefits

Receiving benefits is generally prohibited, subject to the exceptions listed in II below.

It is irrelevant whether these benefits relate to products or services which are not reimbursed since the company paying them markets products or provides services which are reimbursed by compulsory social insurance schemes.

In addition to receiving pecuniary benefits (commissions, kickbacks, reimbursement of expenses), benefits in kind are also prohibited (gifts, invitations, bearing the cost of trips for leisure etc.).

Whether these trips are direct or indirect, i.e. they benefit the professional or are granted to their relatives or groups of which they are a part, is irrelevant. Establishing an association cannot be used as a means of avoiding the provisions of article L.365-1: it is forbidden to obtain benefits through an association which are forbidden to be obtained directly.

However, taking into account both the spirit of the law and the terms of the directive of 31 March 1992, benefits of negligible intrinsic value must not be considered as falling within the scope of the prohibition since, by their nature, they are not really benefits.
Therefore, small gifts are acceptable if they are inexpensive and relate to the practice of medicine or pharmacy. Likewise, accepting a dinner invitation or an invitation to an event such as a cocktail party or buffet is not in itself illegal. However, if there are repeated invitations, the expenses incurred are significant or family members or close relatives of the guest are also invited then the nature of such invitations is changed.
Moreover, the supply of pharmaceutical samples is not covered by the prohibition since this complies with the regulations specified in article R.5046 of the French Public Health Code.
Pursuant to that text, samples can only be provided to persons authorised to prescribe medicinal products, provided that they have requested them and within the limits set by the provisions relating to their right to prescribe. Samples must be identical to the proprietary medicinal products and must be labelled ‘free medical sample’.
Finally, supply of these samples is prohibited at venues which are accessible to the public during medical or pharmaceutical conferences.

II – Exceptions to prohibiting benefits in kind or pecuniary benefits

1 – Research or scientific assessment activities do not fall within the scope of the prohibition provided that they comply with the procedure described in (2) below.
If the legislature intended to prohibit material advantages being granted to professionals by companies providing services or manufacturing or marketing medicinal products or products reimbursed by compulsory social security schemes, it in no way intended to deprive the companies concerned of the opportunity to contribute to the financing of research or scientific assessment activities in the broadest sense, i.e. including both conducting research or assessments and disseminating results.
The following should be regarded as such:

- Research activities *sensu stricto*, including biomedical research governed by Law No 88-1138 of 20 December 1998.
- Conferences, seminars and study days aimed at updating knowledge, research or practices in a specific domain.
- Continuing medical education activities when they are financed under conditions other than those provided for in 1.b.3. The activities outlined above must be geared to the objectives they set out.

In this context, in accordance with the provisions of article 10 of the aforementioned directive, hospitality offered to participants remains lawful when it is of a reasonable level, remains of secondary importance in comparison to the scientific and professional objective, and is not extended to persons other than the professionals directly concerned.
Clarification is required regarding the remuneration of biomedical research and the remuneration of patents.
As regards the remuneration of biomedical research activities, remuneration must, if it cannot be proportional to the number of products prescribed, correspond to the significance of the work accomplished within the study protocol and may, for example, take account of the number of observations requested from the practitioners
As regards the patent system, patents are inherently the result of research and the law does not change their remuneration conditions, provided that that they are subject to an agreement.
In particular, the law does not preclude a payment being made which is proportional to the total turnover of the product covered by the patent.
The prohibition of proportionality applies to the value and number of products prescribed by the practitioner and not to the activity of the company which has acquired the right to use the patent.

2 – Conventional procedure must be adhered to
Benefits granted to healthcare professionals by companies must be the subject of a written agreement. Before the agreement is implemented, professionals who are members of associations must submit said agreement to the departmental board of the relevant professional association for its opinion.
Although article L.365-1, paragraph 2, of the French Public Health Code expressly refers only to the medical association, as dental surgeons and midwives also have their own associations, the powers conferred to the departmental board of the medical association with regard to physicians must also be conferred to these other associations with regard to their respective workers. On the other hand, paramedics do not have a professional association and are not subject to this formality.
Moreover, when research or assessment activities are conducted, even partially, in a healthcare facility, they must be submitted to the person in charge for their opinion.
The need to respect the transparency that underlies the legal provision, without imposing undue formalism, leads us to clarify several things regarding its application.

a) Form and content of the agreement
In order for the professional associations to provide an informed opinion, the agreement must be in writing and contain certain information (purpose of the activity in question, location, duration, nature and sum of the benefits granted and, where events or education activities are concerned, a program revealing the time dedicated to scientific activities in relation to the total duration).
However, the nature of the document will obviously vary depending on the purpose of the agreement in question.
While it will sometimes take the form of a fully-fledged contract, signed by the parties involved, this will not be the case when it is an invitation to a conference or a continuing education activity. In this case, the invitation itself addressed to the professional constitutes an agreement since it contains information on the content and conduct of the event and specifies the services provided by the company.

b) Agreement communicated to the board of the professional association in question pursuant to another legislative text:
Article L.462 of the French Public Health Code requires physicians and dental surgeons to communicate to the departmental board of the professional association of which they are a member ‘a complete set of their contracts concerning the exercise of their profession’. As soon as an agreement has been submitted under this article or another provision of the same kind, the formality provided for under article L.365-1 of the French Public Health Code shall be considered as having been met.
This will apply in particular to trials conducted under Law No 88-1138 of 20 December 1988 (Huriet Law).
For phase I, II and III trials which have received a favourable opinion from the *Comité régional de protection des personnes dans la recherche biomédicale* (Regional Committee for the Protection of Persons in Biomedical Research), it shall not be necessary to re-examine the content of the agreement unless the practitioner’s remuneration is clearly unjustified or the service provided is not consistent with the intended objective.
However, for phase IV trials, the review of the agreement must include all aspects of the agreement.

c) Date when the agreement must be submitted to the departmental board of the professional association
According to the second paragraph of article L.365-1, agreements must be ‘submitted for opinion’ before they are implemented. However, it is not necessary for the opinion itself to have been given before the agreement is implemented, which often causes practical problems.
It is up to the law enforcement officers, of course, to check, whenever possible, that this opinion has been requested.

d) Research, conferences and education activities for professionals throughout France or in different departments
In order to simplify the task of examining applications by departmental boards and to ensure a degree of harmonisation of their interpretations, we feel that there is everything to gain from agreements relating to research, conferences, seminars and continuing education activities, which by their nature are directed at professionals throughout the country or even in several different departments, being referred to the national board of the association concerned, which could provide an opinion on the principle of the agreement itself; it would not consider the scientific value of the activities concerned but rather their compliance with ethical guidelines and the scientific nature of the subjects covered.
The departmental boards to which individual applications are referred would of course retain their freedom of judgment regarding the opinion formulated for each individual application, in particular where the qualifications of the practitioner are concerned.

e) The specific case of professionals working in a healthcare facility
Article L.365-1 stipulates that the agreements shall be notified to the head of any healthcare facility where the research or assessment activities are carried out, even in part.
In the case of public healthcare facilities, this provision is a reminder of the general regulations which apply to persons working in these facilities.
In this case, there is simply a duty to inform the person responsible, as the latter does not have the discretionary power to assess the validity of the research or assessment activity.

III – Services other than those related to research or scientific assessment activities conducted by practitioners

The general prohibition established by the law on the payment of any benefit in kind or pecuniary benefit by companies is obviously not intended to prohibit normal working relations, such as assignments, contracts of employment or participation in scientific boards.
However, if the remuneration of such activities is not commensurate with the services actually rendered, they shall fall within the scope of the prohibition referred to in article L.365-1.
Nevertheless, in accordance with article L.462, the practitioners shall be responsible for communicating to the departmental board of the relevant professional association a complete set of their contracts concerning the exercise of their profession.
In complying with these instructions, we ask that you endeavour to look out for any potential abuse, bearing in mind the time required to take into account legal provisions, and to make their meaning and scope known to those professionals you deal with. The need to sanction behaviour which is clearly wrong should obviously not be seen as a systematic mistrust of professionals, the vast majority of whom are committed to independently carrying out their activities.

Minister of Health
Philippe DOUSTE-BLAZY

ANNEX 2.4
GOOD CLINICAL PRACTICE

Ministry of Social Affairs
and Employment
Ministry of Health
and Family Affairs

Department of Pharmacy
and Medicines

GOOD CLINICAL PRACTICE

Recommendations for sponsors and investigators for clinical trials on medicinal products

(Published in the French Official Gazette)

1987

Preamble

Good Clinical Practice (GCP) is defined by a set of provisions to be implemented to ensure the quality and authenticity of scientific data and ethical compliance in clinical trials.

These recommendations are constantly evolving. They specify the respective responsibilities of the sponsor and investigator and entail the implementation of an appropriate set of controls.

04/01/89

Good Clinical Practice is incorporated in the medicinal product quality assurance system which includes the development phase, production and dispensing. The aim of Good Clinical Practice is to reinforce quality control for clinical trials on medicinal products conducted in France. Good clinical practice is not intended to assess the intrinsic scientific value of a study.

1. GLOSSARY

1.1. Quality assurance system
Quality assurance is achieved through ‘implementing a suitable set of pre-established and systematic provisions aimed at building confidence in achieving the required quality’. This set of provisions is referred to as a quality assurance system.

1.2 Quality control
Any control is a ‘verification of compliance with pre-determined data, followed by an appraisal’. Verifying clinical trial compliance with GCP is referred to as quality control.

1.3 Sponsor
The sponsor is an individual or organisation which initiates the conduct of a clinical trial.

1.4 Investigator
The investigator is responsible for the practical conduct of the clinical trial proposed by the sponsor and for summarising the data collected in the form of a report.

1.5 Monitor
The monitor is the person appointed by, and responsible to, the sponsor for the monitoring of the trial. The monitor is a link between the sponsor and the investigator and reports to the sponsor.

1.6 Trial site
The trial site is a public or private healthcare centre, medical practice, or centre which has the equipment required for the conduct of clinical trials, compatible with proper scientific rigour and safety.

1.7 Pre-requisites
The pre-requisites include essential information which must be known prior to the trial being implemented: available analytical, pharmaceutical, toxicological, pharmacokinetic, pharmacological and clinical data.
The data can be presented as a confidential summary document, called for example ‘investigation brochure’. In addition to this document, the investigator has access to all of the data available.
This information is updated as the work progresses.

1.8 Protocol
Document defining the objective of the trial and the conditions under which it should be conducted.

1.9 Case report form (annex to the protocol)
Document used to record data on each trial subject during the course of the trial, as defined by the protocol.
The data should be collected by procedures which guarantee editing and preservation and allow quality control.

1.10 Subject identification documents (annex to the protocol)
A procedure for revealing the identity of subjects must be in place for double-blind trials, so that, in emergency cases, it is possible to find out the nature of treatment assigned to a specific subject. Subject identification documents shall be kept in sealed opaque envelopes or by any other appropriate means to preserve secrecy. These documents must be returned to the sponsor at the end of the trial.

1.11 Operating procedures (annex to the protocol)
Written instructions describing the activities to be carried out and the precautions and measures which need to be taken which are not specified in the protocol.

1.12 Patient file
A specific file (e.g. hospital file, consultation records) must exist for each subject included in the trial. In particular, in accordance with the provisions regulating the consultation of these types of documents, it permits the authenticity of the information presented in CRFs to be verified and, where necessary, completed or corrected.

1.13 Investigational products
Any product studied within the framework of a trial. In comparative trials the investigational product is compared with one or more reference products or a placebo.

1.14 Batch
Defined quantity of a product obtained from a specific manufacturing cycle: the essential quality of a manufactured batch is its homogeneity.

1.15 Contract between the sponsor and the investigator
This contract defines the relationship between the sponsor and the investigator and includes:
- The general provisions: specific task allocation for the two parties, anticipated timescale for conducting the trial, conditions for publication of the works and provisions covering the trial in terms of civil liability and confidentiality
- Other provisions, if any, including financial, which are not included in the GCP framework.
  NB The conduct of a trial can also lead to the signing of other contracts between the sponsor and the healthcare centre and between the sponsor and other service providers involved in the implementation and monitoring of the trial.

2. RESPONSIBILITIES OF THE SPONSOR

2.1 Selection of the investigator
The sponsor shall choose the investigator(s) in charge of conducting the trial.

2.2 Selection of the monitor
The sponsor shall choose and instruct the monitor(s) in charge of monitoring the trial.

2.3 Sponsor-investigator relationship
This monitor is the intermediary for this relationship.

2.3.1 First visit
Before the trial commences the monitor shall visit the chosen investigator and, on this occasion, shall:
- Check the investigator's qualifications
- Check the investigator's capability to successfully carry out the requested trial in terms of:
    - availability
    - subject recruitment
    - technical equipment and environment
- Check the compatibility of the proposed trial with the investigator's other research projects on similar types of subjects
- Provide the pre-requisites to the investigator

- Receive a document from the investigator describing the team which shall participate directly or indirectly in this study, with their names and the responsibilities allocated to them for the study
- Communicate to each investigator the names of the other investigators conducting trials on the same medicinal product
- Inform the investigator of the procedures to be followed in the case of critical events and the ways to contact the competent person(s) designated by the sponsor in an emergency
- Discuss the protocol and its annexes in detail prior to written acceptance by the sponsor and investigator
- Collect all the examination standards and methods which shall be used during the trial
- Check that the storage conditions of the investigational products are adequate
- Verify that the two parties have confirmed their acceptance of the contract provisions in writing
- Ensure that, for their part, the sponsor and the investigator have agreed in writing to comply with Good Clinical Practice and to accept the scheduled inspections.

2.3.2 Periodic visits

The monitor shall maintain personal contact with the investigator by making periodic visits and by bringing together, if necessary, the team participating in the trial, and shall ensure that:

- The protocol and its annexes are adhered to: they shall try to obtain as much information as possible on missing data
- The data in the CRF comply with the patient files
- The investigational products are used in accordance with the provisions of the protocol
- Raw data are retained in accordance with regulations, thereby making it possible to check the information entered in the CRF (e.g. biological examinations, X-ray etc.)
- The sponsor and investigator accept in writing any amendments to the protocol and its annexes.

2.4 ‘Ethics committee’ consultation

Prior to commencing the trial, the sponsor shall become acquainted with the opinion of the ethics committee obtained by the investigator.

The sponsor and investigator undertake to not accept any substantial amendment to the protocol without notifying the ‘ethics committee’.

2.5 Responsibilities concerning adverse events

The sponsor shall notify the National Pharmacovigilance System of any serious adverse events which they become aware of during the trial.

The sponsor shall notify the investigator and the National Pharmacovigilance System of any serious adverse events recorded during other simultaneous trials, even if they are carried out abroad.

2.6 Responsibilities relating to the investigational products

The sponsor is responsible for ensuring the suitability of the packaging of investigational products for the use specified in the protocol.

A representative sample for each of the batches used in the trial shall be kept for audit purposes.

The sponsor shall keep a record of the quantities of products sent to the investigator for the trial along with the corresponding batch numbers.

They shall ensure the future of the products as follows:

- Delivery of the products shall be taken in accordance with established supply arrangements
- They shall be stored in accordance with the conditions specified in the protocol
- They shall be used exclusively within the limits specified in the protocol
- Unused products shall either be returned or destroyed by an authorised person

If the pharmacist of the healthcare centre is involved in the conduct of the trial, they must be provided with sufficient information.

2.7 Record-keeping
The sponsor shall organise the storage of the following documents in appropriate facilities:
- Pre-requisites
- Protocol and annexes
- Personal data
- Monitoring documents
- Final report
- Statistical analyses

Archived data shall be indexed in order to facilitate orderly storage and fast searching. The retention period is ten years after the end of the trial.

2.8 Regulatory obligations
The sponsor shall ensure compliance with the regulatory requirements.

2.9 Special case: the service provider
The sponsor may delegate all or part of its rights and duties to a service provider. This does not exempt the sponsor from its own responsibility.

3. RESPONSABILITIES OF THE INVESTIGATOR

3.1 Before the trial commences

3.1.1 Qualifications
The investigator must have qualifications which allow him to accept accountability for the trial. In the specific case where the investigator is not legally authorised, from a statutory point of view, to recruit patients, prescribe therapy, provide care and ensure clinical monitoring, they must employ an authorised person.
The initial training of the investigator, their professional experience and participation in previous clinical trials shall be taken into account in the definition of proficiency to perform clinical trials. A curriculum vitae which reflects this is requested by the sponsor.

3.1.2 Familiarity with the investigational products
The investigator shall be acquainted with the pre-requisites and if they feel additional information or clarification is required they shall ask the sponsor to provide this.
In addition, the investigator shall ensure that the investigational products are suitable for the intended use specified in the protocol (packaging, expiry date) and that they are stored correctly.

3.1.3 Familiarity with the protocol and its annexes
Together with the sponsor, the investigator shall contribute to the development of the protocol and its annexes (CRFs, subject identification documents).

3.1.4 Availability
The investigator and their team must be available to conduct the trial.
Specifically, the investigator shall ensure that the proposed trial shall not be disrupted by any other trials.

3.1.5 Suitability of the facilities and equipment
The investigator shall organise the technical facilities which they have available to ensure, depending on the nature of the trial:
- The implementation of systems specific to the trial (for the subjects, samples etc.)
- The safety of the subjects, especially in emergency situations
- The storage of product batches in a clearly-defined place, ensuring that they are properly stored and not accessible to third parties
- The archiving of documents during and after the trial.

3.1.6 Subject recruitment

Prior to the signing of the protocol, the investigator shall ensure that they can recruit enough subjects to guarantee that the trial is conducted successfully.

During the recruitment the investigator must be especially vigilant about any factors which could disrupt the study, for example:

- Inability to monitor the subjects (subject living a long distance from the trial location)
- Inability of some subjects to comply with the requirements of the trial (e.g. linguistic or intellectual barrier)
- Possible interference (attending physicians, other healthcare facilities).

3.1.7 Subject's consent

The investigator shall obtain the consent of the subjects. The contents of the information given to the subjects and the means of obtaining consent shall be clearly indicated by the investigator.

In the case of trials without direct therapeutic aim for the subjects, consent is usually obtained in writing.

3.1.8 Ethics committee opinion

The investigator shall bring matters before the 'ethics committee' and communicate its opinion to the sponsor.

3.1.9 Contract acceptance

After discussing the draft contract with the sponsor, the investigator shall provide their agreement in writing.

3.1.10 Investigator commitment

The investigator shall undertake in writing to confirm to Good Clinical Practice; they accept the sponsor's periodic visits and what these involve. The investigator also accepts that the supervisory authorities shall carry out inspections.

3.1.11 Formation of the team

For all personnel ensuring the continuity of healthcare services (day, night, care) the investigator shall:

- Define in writing the respective roles of different team members involved in the trial according to their skills
- Ensure that each member of staff is informed of the current protocol and has fully understood the duties they are expected to fulfil as part of the trial
- Take responsibility for the training required for these duties
- Designate the person(s) who is/are specifically responsible for the administrative management of the trial
- Ensure that the other departments or services involved in the practical conduct of the trial are informed about the execution of the trial and determine with them the specific operating procedures.

3.2 During the trial

3.2.1 Compliance with the protocol and its annexes

The investigator shall ensure that the protocol and its annexes are scrupulously followed, especially when other services are involved in the trial. A directory of operating procedures shall be kept.

The investigator shall discuss any amendments to all the documents with the sponsor before accepting these in writing. When these amendments are deemed to be significant, the investigator shall notify the 'ethics committee'.

3.2.2 Data collection

The investigator shall ensure quality of the data collected in the CRFs.

The data obtained during the trial shall be legibly and indelibly directly recorded according to the projected timeline. Any amendment to raw data must be dated, signed and explained.
The original record must not be obscured.
Data recorded on magnetic media must be suitably identified. Amendments, if any, shall comply with the procedures defined for hard copy.
The investigator shall provide the documents which may be required in accordance with protocol procedures.

3.2.3 Management of investigational products
The investigator shall manage the product stocks; this may be in connection with the healthcare centre's pharmacist if the trial takes place in a healthcare centre.

3.2.4 Critical events
The investigator shall inform the sponsor as soon as possible of any critical event which arises during the trial. Individual clinical decisions regarding critical events not anticipated in the protocol are the sole responsibility of the investigator. If these events involve a significant amendment to the protocol, the 'ethics committee' shall be notified thereof.
The investigator shall record and explain any instances where a subject's identity is revealed. This must remain an exceptional procedure.

3.2.5 Availability
The investigator shall allow time for the quality control visits performed during the trial and the meetings prior to drafting the final report.

3.3 After the trial
The investigator shall ensure the future of all the materials (documents, products, equipment etc.) relating to the trial, in agreement with the sponsor. Raw data shall be kept by the investigator for ten years.
Each investigator shall date and sign the final report which shall imply that they accept responsibility for the validity of the data and shall confirm that the data has been obtained in accordance with Good Clinical Practice principles.
The persons responsible for analysing the results shall all sign the final report.
Corrections and additions to the final report must be carried out by way of an amendment, which is justified, signed and dated by the investigator(s).
The statistical analysis of the data may be provided or financed by the sponsor or the investigator.

4. SPECIFIC ASPECTS OF CERTAIN TRIALS

4.1 Multicentre trials
A multicentre trial is conducted simultaneously by several investigators at different sites using identical methods and following the same protocol, with the aim of collecting the data more quickly for a comprehensive analysis, resulting in the drafting of a single joint report.

A multicentre study requires the implementation of an administration system for facilitating and overseeing the implementation and conduct of the trial. Several aspects are rendered more complex in multi-centre trials, such as:
The elaboration, discussion and written acceptance of the protocol and its annexes by all investigators (when investigators are recruited following the withdrawal of certain centres the joint preparation of the protocol is not always feasible)

- The organisation of initial and intermediary meetings
- The implementation of the study

- The preparation or verification of the randomisation and the packaging of products intended for the trial
- The training of investigators to follow the same protocol
- The standardisation of evaluation methods
- Data centralisation
- Control of adherence to the protocol
- Drafting of the final report

In addition, it can also be useful to have a monitoring committee, which maintains contact with the ethics committee to which it notifies its decisions. Where appropriate, this monitoring committee shall discuss protocol amendments, settle disputes concerning the monitoring, the conduct of the trial and the interpretation of the results. Its members are usually external to the co-ordinating body.

4.2 Medical practice trial
Conducted in general practice or specialty medicine, this type of trial is usually a multicentre trial.
It may be undertaken for any type of trial which can be conducted as an outpatient trial; however, being a medical practice trial, the investigator must be especially vigilant about some obligations:
- Having the necessary facilities to conduct the trial
- Undertaking to accept the constraints of the trial
- Offering availability and recruitment opportunities which are compatible with the conduct of the trial.

All the provisions described in the general chapters are compatible with the conduct of a medical practice trial.

4.3 Trial without therapeutic aim
This applies to any trial intended to improve knowledge of a product, without a specific therapeutic aim being sought for the participating subjects.
The following points are particularly important:
- The investigator must have a specific area of expertise
- Necessary and sufficient facilities to ensure the safety of trial subjects
- Terms and procedures for the recruitment of trial subjects and especially the quality assessment of ‘healthy volunteers’
- Conditions of compensation for subjects
- Consent of the subjects, usually in writing.

5. RESPONSABILITES OF THE ETHICS COMMITTEE

5.1 Ethics committee consultations
The ethics committees deal with matters referred to them by the investigators. They have an advisory role.

5.1.1 During an initial examination
The trial can only commence once the opinion of this committee has been given, within the specified period, as provided for in its Rules of Procedure. The following should be considered:
- The acceptability of the investigator for the proposed trial and the suitability of the facilities available to them
- The general suitability of the protocol submitted by the investigator
- The balance between the objective of the trial, its predictable risks and any inconveniences associated with the procedures provided for in the protocol
- The information for the subjects or their parents or guardians, as well as the methods of obtaining consent. In exceptional circumstances where consent cannot be obtained, the reasons why must be provided
- The provision of insurance covering the civil liability of the investigator and sponsor.

5.1.2 During the trial, in the case of significant new information
The ethics committee is referred to when new information leads to a significant amendment to the protocol or is likely to call into question the initial opinion.

5.2 Exchanges between the ethics committee and the investigator
The ethics committee sends its opinions in writing to the investigator. The correspondence with the investigator and the submitted core documents shall be retained for a period of 10 years following the date of the final report.

6. QUALITY CONTROL

The implemented quality control ensures that the clinical trial is properly conducted in accordance with GCP principles.
There are three different levels.

6.1 Quality control by the investigator
The investigator, who is responsible for the practical conduct of the trial, shall ensure the quality of the work carried out by the team, especially regarding compliance with the protocol and its annexes, data collection and management of the investigational products.

6.2 Quality control by the sponsor
This control is carried out by one or more persons (possibly the monitor) or by a service provider, or by another organisation designated by the sponsor, provided that the investigators involved in the trial are excluded. The investigator and the sponsor must agree on the nature and frequency of these controls.
The purpose of quality control is to verify, in particular through regular visits, the facilities and conditions under which the trial is being conducted and to assess quality and reliability of the data and information provided to the sponsor. Records of these visits should be retained.
The sponsor and the investigator must be informed periodically, and where necessary, of the conclusions of such visits and any corrective action to be taken.
A certificate of compliance with GCP is included in the final report.

ANNEX 2.5
OPINION OF THE CPP
(ETHICS COMMITTEE)

ILE DE FRANCE ETHICS COMMITTEE X
ROBERT BALLANGER HOSPITAL
Boulevard Robert Ballanger – Building No. 8 (3rd floor) – 93602 Aulnay-sous-bois cedex
Tel.: 01 49 36 73 57 – Email: cpp.iledefrance10@ch-aulnay.fr
Chairman: Prof. Philippe CASASSUS

Registered letter with acknowledgement of receipt
No. 1A 052 185 8259 1

Mr Marc PIOCHAUD
SPRIT
7, rue Lallier
75009 PARIS

Aulnay-sous-Bois, 9 June 2011

Your ref.: **OSTP-EUR-10-01**
Our ref.: **PC/AP 19-2011**
RCB ID No.: **2011-A00258-33**

Dear Mr Piochaud,

The Ile-de-France Ethics Committee X received an application from you for an opinion on the biomedical research project relating to a medical device or an in vitro diagnostic medical device entitled:
"Efficacy of OSTENIL PLUS (hyaluronic acid) versus SYNVISC-ONE in patients with tibiofemoral osteoarthritis. A randomised, controlled, double-blind, parallel-group study with a 6-month follow-up" the sponsor of which is TRB Chemedica.

On receipt of the amendments made in compliance with its recommendations, the Committee issues a **FAVOURABLE OPINION on the implementation of this study,** with regard to the following documents:

- Reply letter .... dated 27 May 2011
- Initial application form to the EC for an opinion on biomedical research (DM)........ dated 8 April 2011
- Additional document for application to the EC for an opinion (DM) .... dated 8 April 2011
- Receipt confirming research registration number. .... dated 18 February 2011
- Sponsor's written authorisation for delegation of powers........ dated 7 April 2011
- Certificate of insurance: Chubb Insurance Company Europe SE........ version 2 dated 7 April 2011
- Research protocol .... version 3 dated 16 May 2011
- Summary of the study ....
- Patient information sheet .... version dated 16 May 2011
- Informed consent form for participation .... version dated 16 May 2011
- List of Investigators participating in the study ....
- CV of the co-ordinating investigator and the participating investigators ....

ILE DE FRANCE ETHICS COMMITTEE X

ROBERT BALLANGER HOSPITAL
Boulevard Robert Ballanger – Building No. 8 (3rd floor) – 93602 Aulnay-sous-bois cedex
Tel.: 01 49 36 73 57 – Email: cpp.iledefrance10@ch-aulnay.fr
Chairman: Prof. Philippe CASASSUS

- Evaluating investigator's CRF .... version 4 dated 16 May 2011
- Injecting investigator's CRF .... version 4 dated 16 May 2011
- Patient diary ....
- Investigator's Brochure for Ostenil Plus *in English* .... version dated 23 March 2011
- Information sheets on the medical devices:
  - Ostenil Plus (DM class III) ....
  - Synvisc (DM class III) ....

The following Committee members took part in the discussions:

Biomedical research:	Mr Philippe CASASSUS (*)	full member
	Mr Jean-Luc GAILLARD	full member
	Ms Ilham MOUMNA	deputy member
General practitioner:	Ms Elisabeth HENON	full member
Hospital pharmacist	Ms Patricia LEROUX	full member
Approved associations of patients or health system users:	Ms Marie-Claude FEINSTEIN	full member
	Ms Philippe MAUGIS	deputy member

The following deputy members were present for the discussions but did not take part:

General practitioner:	Mr Daniel FAUCHER	deputy member

(*): epidemiologist/biostatistician

Yours sincerely,

[Signed]

The Chairman,
Professor Philippe Casassus

[THIS OPINION CONSISTS OF TWO PAGES]

*Should it become necessary to submit **a substantial amendment to the protocol** to our Committee at a later date, please send us the **new version of the protocol, or the information sheet, or the consent form, clearly indicating the proposed changes** (in bold, or highlighted, or noted in the margin, or on specific pages showing the former and the new version).*

ANNEX 2.6

OPINION OF THE AFSSAPS (FRENCH HEALTH PRODUCTS SAFETY AGENCY)

FRENCH REPUBLIC

afssaps
Agence française de sécurité sanitaire des produits de santé

(French national agency for health products safety)

Department for the assessment of medical devices
Clinical trials unit
File monitored by Lynda Arnaud-Boissel
Tel.: +33 (0)1 55 87 37 53
Fax: +33 (0)1 55 87 37 17
E-mail: dedim.dm@afssaps.samte/fr
Ref. No.: UEC/LynAB/DA/2001-

Saint-Denis, **20 MAY 2011**

RE: CLINICAL TRIAL ASSESSING THE EFFICACY OF OSTENIL PLUS (HYALURONIC ACID) VERSUS SYNVISC-ONE IN PATIENTS WITH TIBIOFEMORAL OSTEOARTHRITIS
Registration No.: 2011-A00258-33

Dear Sir,

My department has received an authorisation application from you for the above-mentioned biomedical research.

In light of the answers provided on 17 May 2011 (protocol version 3 of 16 May 2011) to the objections raised in the letter dated 10 May 2011 from Afssaps (*Agence française de sécurité sanitaire des produits de santé* - French national agency for health products safety), I hereby authorise this biomedical research in France. In accordance with article L.1121-4 of the French Public Health Code, I must point out to you however that this biomedical research must obtain a favourable opinion from the REC (Regional Ethics Committee) for it to take place in France.

I would also like to remind you that, while the research is being conducted, and as regards Afssaps, any substantial modification to the file originally submitted must obtain authorisation in accordance with articles L.1123-9 and R.1123-35 of the French Public Health Code. Serious adverse events/effects as well as new events likely to affect the safety of individuals must be declared in accordance with article L.1123-10 of the French Public Health Code and the end of the trial must also be declared in accordance with Article L.1123-11 of the French Public Health Code.

Yours faithfully,

Head of Market Surveillance Department

[signature]

Nicolas THEVENET

SPRIT
7, rue Lallier
75009 Paris

For the attention of Mr Marc PIOCHAUD

Copy to: Ile de France X REC

ANNEX 2.7

PATIENT INFORMATION LEAFLET

PATIENT INFORMATION LEAFLET

CLINICAL TRIAL ASSESSING THE EFFICACY OF OSTENIL PLUS (HYALURONIC ACID) VERSUS SYNVISC-ONE IN PATIENTS WITH TIBIOFEMORAL OSTEOARTHRITIS
A RANDOMISED, CONTROLLED, DOUBLE-BLIND, PARALLEL-GROUP STUDY WITH A 6-MONTH FOLLOW-UP

Please read this document carefully. It is based on the French law of 20 December 1988 on the protection of persons participating in biomedical research.

Dear Sir/Madam,

You suffer knee osteoarthritis which manifests itself as pain and functional impairment.

In general knee osteoarthritis is an osteoarthritis affecting the joint between the femur and the tibia, also known as tibiofemoral osteoarthritis.

Osteoarthritis is a joint disease which primarily affects the cartilage but can also affect the bone under the cartilage and the structures which make up and surround the joint. Joint cartilage gradually loses its impact resistance and elasticity properties. Impact, trauma or pressure to the joint are less well absorbed. The joint fluid, which also functions as a shock absorber and lubricant, loses its qualities. The joint is less lubricated, less elastic and less resistant to pressure. There is therefore more stress on the bone under the cartilage and it can react painfully.
Most of the time osteoarthritis develops slowly and results in mechanical pain: for example, when walking or going downstairs in the case of knee osteoarthritis like yours. The pain is accompanied by functional impairment in daily living which is debilitating to a greater or lesser extent and stiffening phenomenon, specifically when starting to move after maintaining a prolonged sedentary position (e.g. in the morning or when standing up from a sitting position). Sometimes the development of this osteoarthritis is interspersed with a much more painful and inflammatory flare up with swelling of the joint (due to excess fluid accumulating which is most clearly visible in or around your knee joint which can be punctured).

Knee osteoarthritis treatment requires various physical and medicinal measures. Recent European recommendations on the treatment of knee osteoarthritis have identified numerous therapeutic options:

- Medicinal: analgesics, anti-inflammatories, symptomatic slow and long-acting oral drugs, corticosteroid joint injections or hyaluronic acid joint injections.
- Non-medicinal: weight loss, shoe insoles, quadricep muscle strengthening exercises, kinesiotherapy, physiotherapy, advise on reducing joint overuse, using a walking stick, wearing a knee support, hydrotherapy, providing education and information to the patient on their osteoarthritis etc.

Out of all these treatments, hyaluronic acid joint injections have been recognised for their efficacy. They act on symptoms such as pain and functional impairment. Hyaluronic acid is an important natural component of the cartilage and joint fluid. Its function is probably to provide the cartilage and joint fluid with better shock resistance.
In the case of osteoarthritis, the concentration and molecular mass of hyaluronic acid reduces in the joint fluid. The idea of injecting exogenous (external) hyaluronic acid to restore better concentration and promote the synthesis (production) of better quality hyaluronic acid appeared attractive and led to it being tested with knee osteoarthritis.

Since the early 1980s over 100 studies have been carried out on patients with knee osteoarthritis, demonstrating the efficacy and good safety of these joint injections: in fact, there have been few side effects; mainly local reactions which were not very intense, short lived and self-limiting.

Intra-articular hyaluronic acid is therefore recommended in knee osteoarthritis patients who are suffering despite taking analgesics or who are having trouble with analgesics or anti-inflammatories.

The method of administration is three consecutive injections at the rate of one joint injection per week. However the laboratory TRB CHEMEDICA has recently developed a new form allowing one single injection.

The aim of this randomised, multi-centre clinical trial, sponsored by the laboratory TRB CHEMEDICA, which we are inviting you to participate in, is to assess the efficacy and safety of OSTENIL PLUS versus another hyaluronic acid which already exists in the form of an injection: SYNVISC-ONE of different molecular weight.

This study will last for six months during which the development of your osteoarthritis will be monitored very carefully, even if you withdraw from the trial. Only one knee will be assessed and treated. Your physician will give you all of the information regarding the visit schedule and the conduct of the study.

During the first consultation, once your physician has explained the purpose of the trial to you and you have agreed to participate in the trial, you will go to see another physician, either a rheumatologist, a physical rehabilitation physician or an orthopaedic surgeon, who will administer on the same day (where possible) or the next day, a joint injection of either of the products randomly assigned to you: OSTENIL PLUS or SYNVISC-ONE.
This procedure is necessary so that neither your physician nor you know which hyaluronic acid you have been injected with in order to avoid favouring one or the other product.

You will see your physician three more times, on the 30th, 90th and 180th day after your enrolment to the study, so that s/he can assess your knee osteoarthritis with you. Therefore, in total there will be four consultations, plus one visit for the injection. You will be asked to record all treatments you take to treat your knee osteoarthritis in your patient diary which will be provided at the beginning of the study.

As the hyaluronic acid is injected into the joint, general precautions relating to this type of injection must be observed. Injection into blood vessels and surrounding tissues should be avoided. Special attention should be paid to patients with known hypersensitivity to drugs.
OSTENIL PLUS is not advised if there is a history of hypersensitivity to one of its components.

The potential risks of this treatment are as follows:

1. Painful reaction during infiltration, as with all other infiltrations, or reaction during injection of the product, as with any product for use within the joint. This reaction is short lived and does not require any treatment.
2. Painful reaction at the injection site after infiltration. This is infrequent and short lived, and usually does not require any specific treatment.
3. In some rare cases a very painful inflammatory reaction may occur 24-48 hours after the injection, sometimes with excess fluid in or around your knee joint and a sensation of heat. This can be treated by resting, applying an ice pack and using an anti-inflammatory for a few days. This "arthritis" regresses and most of the time does not prevent the treatment from being continued.
4. Finally it should be noted that there is a very rare but serious risk of infection of the joint (septic arthritis). This septic arthritis results in increased pain, swelling of the knee, redness and heat around the injection site and possibly fever. If you experience any of these symptoms you must contact your rheumatologist immediately. You will require treatment and possibly hospitalisation.
5. There are no general effects with this treatment.

Your physician is familiar with all of these reactions as s/he often uses hyaluronic acid knee injections. You must make him aware of any problems that arise during or just after the injection, or throughout the study period.

You must not participate in any other clinical trial throughout the duration of this study. The exclusion period for participation in another trial is 3 months after the end of this study.

Medical data and information about you will be processed electronically.
Information about you will be coded and identified only by a number in order to protect your anonymity.
This information will only be forwarded to the sponsor and where appropriate the competent health authorities under conditions which ensure confidentiality is maintained.
The computerised file used in this research has been approved by the CNIL (*Commission Nationale de l'Informatique et des Libertés* – French Data Protection Authority) in accordance with articles 40-1 et seq. of the French law *"Informatique et Libertés"* on information technology, data files and civil liberties.
These data will be controlled in accordance with current regulations.

The results of this study may be published in a medical journal or presented to the administrative authorities without your identity being revealed.
In accordance with article L-1122-1 of the French Public Health Code you may, if you wish, be informed of the overall findings of the research in which you have agreed to participate.
Your physician will inform you of the overall findings of this study.

In accordance with the Huriet law of 20 December 1988 and its decrees, the laboratory TRB CHEMEDICA, which is the sponsor of this trial, has taken out civil liability insurance for the protection of participants. This insurance covers the risks associated with the study that may occur during its conduct.

In accordance with French law this research has received a favourable opinion from the Ile de France X Research Ethics Committee.

In accordance with the law of 20 December 1988 prior written confirmation of your agreement is required.
However you have the right to refuse to participate in the research or to withdraw your consent at any time during the conduct of the study with no liability, without altering your care or good relationship with your physician. The same shall apply in the case of suspension or early termination of the study.

Date:
Name of physician:
Signature:

Date:
Signature of patient:
(preceded by the text “read and approved”)

ANNEX 2.8

PATIENT INFORMED CONSENT

PATIENT INFORMED CONSENT

CLINICAL TRIAL ASSESSING THE EFFICACY OF OSTENIL PLUS (HYALURONIC ACID) VERSUS SYNVISC-ONE IN PATIENTS WITH TIBIOFEMORAL OSTEOARTHRITIS
A RANDOMISED, CONTROLLED, DOUBLE-BLIND, PARALLEL-GROUP STUDY WITH A 6-MONTH FOLLOW-UP

Doctor (surname & first name) ____________________

Patient identification (surname & first name) ____________________

Address: ____________________

- I have received and read the patient information leaflet for the OSTENIL PLUS study regarding the sodium hyaluronate 2% viscoelastic solution in the treatment of osteoarthritis of the knee versus SYNVISC-ONE.
- I have noted that the product is intended to improve the symptoms of osteoarthritis in the medium and long term. I have also noted that the study I am invited to participate in involves assessing the efficacy of the treatments.
- I have freely discussed the expected positive benefits and the potential risks with my physician.
- I accept the monitoring conditions, specifically the four consultations and the visit for the injection.
- I consent to participate in this study and I understand that I may withdraw from the study at any time, even after signing the documents.
- I acknowledge that my consent does not in any way relieve the investigator and the sponsor of their responsibilities and I retain all of my rights guaranteed by the law.
- I acknowledge that compliance with professional secrecy will be strictly adhered to when collecting information concerning me during this study.
- I accept that the data recorded for this study may be processed electronically by the sponsor or on his behalf.
- I acknowledge that the computerised file used in this research has been approved by the CNIL (*Commission Nationale de l'Informatique et des Libertés* – French Data Protection Authority) in accordance with articles 40-1 et seq. of the French law *"Informatique et Libertés"* on information technology, data files and civil liberties. These data will be controlled in accordance with current regulations.
- I acknowledge that the right of access provided for under article 40 of the abovementioned law on information technology, data files and civil liberties can be exercised at any time, either directly or via my physician.
- I have received the information and a copy of this document. I have been informed that a copy will also be kept by the research organisers under conditions which guarantee confidentiality and I give my consent for this.
- I also acknowledge that for the duration of this study I cannot participate in another clinical trial and that the exclusion period for participation in another clinical trial is 3 months from the end of this study. I declare that I have not participated in any other biomedical research trial in the last 3 months.
- I consent to the use by the study investigator for legal purposes of all the results and information collected during this study, provided that my anonymity is scrupulously protected.
- In witness whereof, I freely consent.

Date |_|_| |_|_| |_|_|
day month year
Signature of the patient: Signature of the investigator:

Original white copy to be kept by the sponsor TRB CHEMEDICA, yellow copy to be given to SPRIT
Pink copy to be kept by the investigator, green copy to be given to the patient

ANNEX 2.9
INSURANCE CERTIFICATE

PERSONAL INFORMATION

PERSONAL INFORMATION

Date issued: 7 April 2011
Version 2

ANNEX 2.10
REFERENCES

REFERENCES

1. Reginster JY, Henrotin Y. Conception actuelle du traitement de l'arthrose. Revue de la Médecine Générale, 2000; 177: 382-391.

2. Creamer P, Hochberg MC. Osteoarthritis. Lancet 1997; 350: 503-9.

3. Altman RD. Pain relief in osteoarthritis: the rationale for combination therapy. J Rheumatol 2004; 31: 5-7.

4. Felson DT. The course of osteoarthritis and factors that affect it. Rheum Dis Clin North Am 1993; 19: 607-15.

5. Pouchot J, Coste J, Guillemin F. Impact of osteoarthritis on quality of life. In: Reginster JY, Pelletier JP, Martel-Pelletier J, Henrotin Y, Eds. Osteoarthritis Clinical and experimental aspects. Berlin Heidelberg: Springer 1999. p 331-55.

6. IRDES, Enquête Santé Protection Sociale. www.irdes.fr. 2006.

7. Jordan KM, Arden NK, Doherty M, Bannwarth B, Bijlsma JWJ, Dieppe P, et al. EULAR recommendations 2003: an evidence-based approach to the management of knee osteoarthritis: Report of a Task Force of the Standing Committee for International Clinical Studies Including Therapeutic Trials (ESCISIT). Ann Rheum Dis 2003: 62: 1145-55.

8. Maheu E. Principes thérapeutiques généraux des arthroses. Rev Prat (Paris) 1996; 46: 2218-24.

9.Zhang W, Nuki G, Moskowitz RW, Abramson S, Altman RD and al. OARSI recommendations for the management of hip and knee osteoarthritis: Part III: changes in evidence following systematic cumulative update of research published through January 2009. Osteoarthritis and Cartilage, 2010: 18 : 476-499.

10. Maheu E, Lamotte J, Lequesne M. Sel sodique de l'acide hyaluronique (hyaluronan) et gonarthrose. In : MF Kahn, D Kuntz, A Dryll, O Meyer, Th Bardin, Cl Guérin, eds, L'Actualité Rhumatologique 1994, Paris, Expansion Scientifique Française, 1994, p. 324-39.

11. Maheu E, Ayral X, Dougados M. A hyaluronan preparation (500-730 KDA) in the treatment of osteoarthritis : A review of clinical trials with Hyalgan®. Int J Clin Pract 2002; 56: 804-13.

12. Bellamy et al. Viscosupplementation for the treatment of osteoarthritis of the knee. Cochrane Database Syst Rev. 2006 April 19 (2): CD 005321.

13. Chen-Ti Wang, Jinn Lin, Chee-Jen Chang, Yu-Tsan Lin and Sheng-Mou Hou. Therapeutic effects of hyaluronic acid ou osteoarthritis of the knee. A meta-analysis of randomized controlled trials. J. Bone Joint Surg. Am. 86: 538-545, 2004.

14. Lo GH, LaValley M, McAlindon T, Felson DT. Intra-articular hyaluronic acid in treatment of knee osteoarthritis. A meta-analysis. Jama December 17, 2003 vol 290, N°23, 3115-3121.

15. Arrich J, Piribauer F, Mad P et al. Intra-articular hyaluronic acid for the treatment of osteoarthritis of the knee: systematic review and meta-analysis. CMAJ 2005 ; 172: 1039-43.

16. Chevalier X, Jerosch J, Goupille P, van Dijk N, Luyten FP, Scott DL, Bailleul F and Pavelka K. Single, intra-articular treatment with 6 ml hylan G-F 20 in patients with symptomatic primary osteoarthritis of the knee: a randomised, multicentre, double-blind, placebo controlled trial. Ann Rheum Dis, 2010; 69: 113-119.

17. Bellamy N, Buchan WW, Goldsmith CH, Campbell J, Stit LWJ. Validation of WOMAC: a health status instrument for measuring clinically important patient relevant outcomes to antirheumatic drug therapy in patients with osteoarthritis of the hip or knee. J Rheumatol 1995; 15: 1833-40

18. Altman RD, Asch E, Bloch D, Bole G, Borenstein D, Brandt K, et al - Development of criteria for the classification and reporting of osteoarthritis. Classification of osteoarthritis of the knee. *Arthritis Rheum* 1986;29:1039-49.

19. Kellgren JH, Lawrence DM - Radiological assessment of osteoarthritis. *Ann Rheum Dis* 1957;16:494-502.

20. Lequesne M, Méry C, Samson M, Gérard P - Indexes of severity for osteoarthritis of the hip and knee. *Scand J Rheumatol* 1987;65(Suppl):85-9.

21. Huskisson EC - Measurement of pain. *Lancet* 1974;II:1127-31.

22. Lellouche H, Bloch JG, Bellony R. Acide hyaluronique: Ponction et injection intra-articulaire dans la gonarthrose. Rhumatos septembre 2006 vol.3, N°20, 308-309.

23. Pham T, van der Heijde D, Altman RD, Anderson JJ, Bellamy N, Hochberg M, Simon L, Strand V, Woodworth T, Dougados M. OMERACT-OARSI Initiative: Osteoarthritis research society international set of responder criteria for osteoarthritis clinical trials revisited. Osteoarthritis cartilage 2004; 12: 389-399

24. Little, R.J.A. & Rubin, D.B. (1987). Statistical analysis with missing data. New York: Wiley.

25. http://www.ema.europa.eu/docs/en_GB/document_library/Scientific_guidelines /2009/09/wc500002928.pdf

26. Dictionnaire Vidal OCP éd, Paris 2011

27. Dictionnaire Vidal OCP éd, Paris 2010
