# Supplementary material for: A single intra-articular injection of 2.0% non-chemically modified sodium hyaluronate vs 0.8% hylan G-F 20 in the treatment of symptomatic knee osteoarthritis: A 6-month, multicenter, randomized, controlled non-inferiority trial
Source: PLoS One. 2019 Dec 10;14(12):e0226007. doi: 10.1371/journal.pone.0226007 (PMC6903764; doi:10.1371/journal.pone.0226007)
Supplement: S1 Appendix — (DOCX) [file pone.0226007.s004.docx]

**S1 Appendix. Changes to the database.**

Some corrections had to be made to the frozen database on one occasion after ***unblinding*** the study. The procedure was duly recorded and approved by the study coordinator and the scientific committee (Scientific Board) and occurred in the following manner:

**First freezing of the database.** The database was first frozen on 26 February 2013. The freeze was immediately followed (i.e., on the same day) by lifting of the blinding.

**Detection of errors in the database.** After the sponsor had received the complete database, data which had not been presented during the blind review were supplied:

- Presence of 11 serious adverse reactions (i.e., septic arthritis) introduced ***in error*** into the database due to poor programming of automatic controls. Indeed, in the section “Assessment of local treatment tolerance”) in the Case Report Forms concerned, the investigator had clearly mentioned that there was no septic arthritis (“Septic arthritis: No”);
- Concomitant treatments and their influence on the primary efficacy endpoint not studied during the blind review;
- Missing data and discrepancies observed in the database.

The errors and discrepancies were studied by the sponsor, the Contract Research Organization and the scientific committee of the study.

**Request for modifications of the database.** Following the discussions held the following modifications were proposed (see table below for details):

- Removal of the septic arthritis cases appearing by error in the database;
- Corrections following data clarification requests to the investigators (*e.g.,* missing data, discrepancies);
- Change of patient numbers in the various populations of analysis.

These changes were documented and approved by the Scientific Board. A request for correction of the database was sent in writing to the data management and analysis office on 14 July 2014.

**Second freezing of the database.** After thawing and correction the final database was frozen on 5 September 2014.

Changes made to the database (by theme).

| **Patient** | | **Theme** | **Old database locked 26 February 2013** | **New database locked 5 September 2014** |
| --- | --- | --- | --- | --- |
| 067 | | C1, item 2  (Pre-inclusion) | NSAID wash-out observed: NA; analgesics wash-out observed: NA | NSAID wash-out observed: **Yes;** analgesics wash-out observed: **Yes** |
| 317 | | C1, item 2  (Pre-inclusion) | NSAID wash-out observed: NA; analgesics wash-out observed: NA | NSAID wash-out observed: **Yes;** analgesics wash-out observed: **Yes** |
| 320 | | C1, item 2  (Pre-inclusion) | NSAID wash-out observed: NA; analgesics wash-out observed: NA | NSAID wash-out observed: **Yes;** analgesics wash-out observed: **Yes** |
| 358 | | C1, item 2  (Pre-inclusion) | NSAID wash-out observed: Yes; analgesics wash-out observed: NA | NSAID wash-out observed: **Yes;** analgesics wash-out observed: **Yes** |
| 359 | | C1, item 2  (Pre-inclusion) | NSAID wash-out observed: Yes; analgesics wash-out observed: NA | NSAID wash-out observed: **Yes;** analgesics wash-out observed: **Yes** |
| 076 | | C1, item 5  (Altman crit. check) | Age >50 years = Yes | Age >50 yes = **No** |
| 366 | | C1, item 6  (X-ray diagnosis) | X-ray date = NA/XX/XXXX | X-ray date = **XX/XX/XXXX** |
| 392 | | C1, item 7.2  (Cortico. injection) | End date corticosteroid injection = NA/NA/XXXX | End date corticosteroid injection = **XX/XX/XXXX** |
| 068 | | C1, item 7.3  (HA injection) | End date HA injection = NA/NA/NA | End date HA injection = **XX/XX/XXXX** |
| 016 | | C1, item 7.5  (Current Tt) | Tramadol + acetaminophen (IXPRIM): start date = NA/NA/XXXX; end date = NA/NA/NA; ongoing = Yes | Tramadol + acetaminophen (IXPRIM): start date = **XX/XX/XXXX**; end date = **XX/XX/XXXX**; ongoing = **NA** |
| 017 | | C1, item 7.5  (Current Tt) | Ketoprofen (BI PROFENID): start date = NA/XX/XXXX | Ketoprofen (BI PROFENID): start date = **XX/XX/XXXX** |
| 018 | | C1, item 7.5  (Current Tt) | Tramadol (TOPALGIC): start date = NA/NA/XXXX | Tramadol (TOPALGIC): start date = **XX/XX/XXXX** |
| 019 | | C1, item 7.5  (Current Tt) | Diclofenac (VOLTARENE): start date = NA/NA/XXXX; end date = NA/NA/NA; ongoing = Yes | Diclofenac (VOLTARENE): start date = **XX/XX/XXXX**; end date = **XX/XX/XXXX**; ongoing = **NA** |
| 019 | | C1, item 7.5  (Current Tt) | Glucosamine (FLEXEA): start date = NA/XX/XXXX | Glucosamine (FLEXEA): start date = **XX/XX/XXXX** |
| 019 | | C1, item 7.5  (Current Tt) | Tramadol (TOPALGIC): start date = NA/NA/XXXX | Tramadol (TOPALGIC): start date = **XX/XX/XXXX** |
| 020 | | C1, item 7.5  (Current Tt) | Acetaminophen (DAFALGAN): start date = NA/NA/XXXX | Acetaminophen (DAFALGAN): start date = **XX/XX/XXXX** |
| 037 | | C1, item 7.5  (Current Tt) | Acetaminophen (DAFALGAN): start date = NA/XX/XXXX | Acetaminophen (DAFALGAN): start date = **XX/XX/XXXX** |
| 038 | | C1, item 7.5  (Current Tt) | Acetaminophen + codeine (KLIPAL CODEINE): start date = NA/XX/XXXX | Acetaminophen + codeine (KLIPAL CODEINE): start date = **XX/XX/XXXX** |
| 039 | | C1, item 7.5  (Current Tt) | Diclofenac (VOLTARENE): start date = NA/XX/XXXX | Diclofenac (VOLTARENE): start date = **XX/XX/XXXX** |
| 040 | | C1, item 7.5  (Current Tt) | Ketoprofen (BIPROFENID): start date = NA/XX/XXXX | Ketoprofen (BIPROFENID): start date = **XX/XX/XXXX** |
| 041 | | C1, item 7.5  (Current Tt) | Aceclofenac (CARTREX): start date = NA/XX/XXXX; end date = XX/XX/XXXX | Aceclofenac (CARTREX): start date = **XX/XX/XXXX**; end date = **XX/XX/XXXX** |
| 044 | C1, item 7.5  (Current Tt) | | Acetaminophen + codeine (KLIPAL): start date = NA/XX/XXXX | Acetaminophen + codeine (KLIPAL): start date = **XX/XX/XXXX** |
| 050 | C1, item 7.5  (Current Tt) | | Piascledine: start date = NA/XX/XXXX | Piascledine: start date = **XX/XX/XXXX** |
| 050 | C1, item 7.5  (Current Tt) | | Acetaminophen (GELUPRANE): start date = NA/XX/XXXX | Acetaminophen (GELUPRANE): start date = **XX/XX/XXXX** |
| 064 | C1, item 7.5  (Current Tt) | | Prednisone (CORTANCYL): start date = NA/NA/XXXX | Prednisone (CORTANCYL): start date = **XX/XX/XXXX** |
| 067 | C1, item 7.5  (Current Tt) | | Acetaminophen: start date = NA/NA/XXXX | Acetaminophen: start date = **XX/XX/XXXX** |
| 068 | C1, item 7.5  (Current Tt) | | Acetaminophen (DOLIPRANE): start date = NA/NA/XXXX | Acetaminophen (DOLIPRANE): start date = **XX/XX/XXXX** |
| 074 | C1, item 7.5  (Current Tt) | | Diclofenac (VOLTARENE): start date = NA/NA/XXXX; end date = NA/NA/NA (C1 = **XX/XX/XXXX**) | Diclofenac (VOLTARENE): start date = **XX/XX/XXXX**; end date = **XX/XX/XXXX** |
| 074 | C1, item 7.5  (Current Tt) | | Tramadol + acetaminophen (IXPRIM): start date = NA/NA/XXXX; end date = NA/NA/NA (C1 = XX/XX/XXXX) | Tramadol + acetaminophen (IXPRIM): start date = **XX/XX/XXXX**; end date = **XX/XX/XXXX** |
| 076 | C1, item 7.5  (Current Tt) | | Diclofenac (VOLTARENE): start date = NA/XX/XXXX; end date = NA/XX/XXXX | Diclofenac (VOLTARENE): start date = **XX/XX/XXXX**; end date = **XX/XX/XXXX** |
| 076 | C1, item 7.5  (Current Tt) | | Piascledine: start date = NA/XX/XXXX | Piascledine: start date = **XX/XX/XXXX** |
| 081 | C1, item 7.5  (Current Tt) | | Acetaminophen (DOLIPRANE): start date = NA/XX/XXXX | Acetaminophen (DOLIPRANE): start date = **XX/XX/XXXX** |
| 101 | C1, item 7.5  (Current Tt) | | Diacerein (ART 50): start date = NA/NA/XXXX | Diacerein (ART 50): start date = **XX/XX/XXXX** |
| 103 | C1, item 7.5  (Current Tt) | | Glucosamine (VOLTAFLEX): start date = NA/XX/XXXX | Glucosamine (VOLTAFLEX): start date = **XX/XX/XXXX** |
| 114 | C1, item 7.5  (Current Tt) | | Chondroitin (STRUCTUM): start date = NA/NA/XXXX | Chondroitin (STRUCTUM): start date = **XX/XX/XXXX** |
| 114 | C1, item 7.5  (Current Tt) | | Piascledine: start date = NA/NA/XXXX | Piascledine: start date = **XX/XX/XXXX** |
| 117 | C1, item 7.5  (Current Tt) | | Acetaminophen + opium + caffeine (LAMALINE): start date = NA/NA/XXXX | Acetaminophen + opium + caffeine (LAMALINE): start date = **XX/XX/XXXX** |
| 127 | C1, item 7.5  (Current Tt) | | Chondroitin (CHONDROSULF): start date = NA/NA/XXXX | Chondroitin (CHONDROSULF): start date = **XX/XX/XXXX** |
| 142 | C1, item 7.5  (Current Tt) | | Acetaminophen (DOLIPRANE): start date = NA/XX/XXXX | Acetaminophen (DOLIPRANE): start date = **XX/XX/XXXX** |
| 174 | C1, item 7.5  (Current Tt) | | Acetaminophen (DOLIPRANE): start date = NA/NA/XXXX | Acetaminophen (DOLIPRANE): start date = **XX/XX/XXXX** |
| 176 | C1, item 7.5  (Current Tt) | | Diclofenac (VOLTARENE): start date = NA/NA/XXXX; end date = //; ongoing = Yes | Diclofenac (VOLTARENE): start date = **XX/XX/XXXX**; end date = **XX/XX/XXXX**; ongoing = **NA** |
| 177 | C1, item 7.5  (Current Tt) | | Harpagophytum (ARTHROFEN): start date = NA/NA/XXXX | Harpagophytum (ARTHROFEN): start date = **XX/XX/XXXX** |
| 178 | C1, item 7.5  (Current Tt) | | Ketoprofen: start date = NA/NA/XXXX | Ketoprofen: start date = **XX/XX/XXXX** |
| 178 | C1, item 7.5  (Current Tt) | | Acetaminophen (DAFALGAN): start date = NA/NA/XXXX | Acetaminophen (DAFALGAN): start date = **XX/XX/XXXX** |
| 178 | C1, item 7.5  (Current Tt) | | Chondroitin (STRUCTUM): start date = NA/NA/XXXX | Chondroitin (STRUCTUM): start date = **XX/XX/XXXX** |
| 210 | | C1, item 7.5  (Current Tt) | Chondroitin (STRUCTUM): start date = NA/NA/XXXX | Chondroitin (STRUCTUM): start date = **XX/XX/XXXX** |
| 213 | | C1, item 7.5  (Current Tt) | Piascledine: start date = NA/XX/XXX | Piascledine: start date = **XX/XX/XXXX** |
| 217 | | C1, item 7.5  (Current Tt) | Piascledine: start date = NA/NA/XXXX | Piascledine: start date = **XX/XX/XXXX** |
| 229 | | C1, item 7.5  (Current Tt) | Acetaminophen (DOLIPRANE): start date = NA/NA/XXXX | Acetaminophen (DOLIPRANE): start date = **XX/XX/XXXX** |
| 233 | | C1, item 7.5  (Current Tt) | Chondroitin (STRUCTUM): start date = NA/XX/XXXX | Chondroitin (STRUCTUM): start date = **XX/XX/XXXX** |
| 239 | | C1, item 7.5  (Current Tt) | Diacerein (ART 50): start date = NA/NA/XXXX | Diacerein (ART 50): start date = **XX/XX/XXXX** |
| 239 | | C1, item 7.5  (Current Tt) | Piascledine: start date = NA/NA/XXXX | Piascledine: start date = **XX/XX/XXXX** |
| 247 | | C1, item 7.5  (Current Tt) | Chondroitin (CHONDROSULF): start date = NA/XX/XXXX | Chondroitin (CHONDROSULF): start date = **XX/XX/XXXX** |
| 250 | | C1, item 7.5  (Current Tt) | Chondroitin (CHONDROSULF): start date = NA/NA/XXXX | Chondroitin (CHONDROSULF): start date = **XX/XX/XXXX** |
| 257 | | C1, item 7.5  (Current Tt) | Piascledine: start date = NA/XX/XXXX | Piascledine: start date = **XX/XX/XXXX** |
| 261 | | C1, item 7.5  (Current Tt) | Acetaminophen (DAFALGAN): start date = NA/NA/XXXX | Acetaminophen (DAFALGAN): start date = **XX/XX/XXXX** |
| 277 | | C1, item 7.5  (Current Tt) | Piascledine: start date = NA/NA/XXXX | Piascledine: start date = **XX/XX/XXXX** |
| 279 | | C1, item 7.5  (Current Tt) | Chondroitin (CHONDROSULF): start date = NA/NA/XXXX | Chondroitin (CHONDROSULF): start date = **XX/XX/XXXX** |
| 284 | | C1, item 7.5  (Current Tt) | Acetaminophen (DAFALGAN): start date = NA/NA/XXXX | Acetaminophen (DAFALGAN): start date = **XX/XX/XXXX** |
| 301 | | C1, item 7.5  (Current Tt) | Acetaminophen (DOLIPRANE): start date = NA/XX/XXXX; end date = NA/XX/XXXX | Acetaminophen (DOLIPRANE): start date = **XX/XX/XXXX**; end date = **XX/XX/XXXX** |
| 305 | | C1, item 7.5  (Current Tt) | Acetaminophen (DAFALGAN): start date = NA/NA/XXXX | Acetaminophen (DAFALGAN): start date = **XX/XX/XXXX** |
| 305 | | C1, item 7.5  (Current Tt) | Chondroitin (CHONDROSULF): start date = NA/NA/XXXX | Chondroitin (CHONDROSULF): start date = **XX/XX/XXXX** |
| 310 | | C1, item 7.5  (Current Tt) | Acetaminophen + opium + caffeine (LAMALINE): start date = NA/NA/XXXX | Acetaminophen + opium + caffeine (LAMALINE): start date = **XX/XX/XXXX** |
| 311 | | C1, item 7.5  (Current Tt) | Tramadol + acetaminophen (IXPRIM): start date = NA/NA/XXXX | Tramadol + acetaminophen (IXPRIM): start date = **XX/XX/XXXX** |
| 311 | | C1, item 7.5  (Current Tt) | Acetaminophen + opium + caffeine (LAMALINE): start date = NA/NA/XXXX | Acetaminophen + opium + caffeine (LAMALINE): start date = **XX/XX/XXXX** |
| 317 | | C1, item 7.5  (Current Tt) | Piroxicam (PROXALYOC): end date = NA/NA/NA; ongoing = Yes | Piroxicam (PROXALYOC): end date = **XX/XX/XXXX**; ongoing = **NA** |
| 318 | | C1, item 7.5  (Current Tt) | Chondroitin (CHONDROSULF): start date = NA/NA/XXXX | Chondroitin (CHONDROSULF):start date = **XX/XX/XXXX** |
| 321 | | C1, item 7.5  (Current Tt) | Acetaminophen (DOLIPRANE): start date = NA/XX/XXXX | Acetaminophen (DOLIPRANE): start date = **XX/XX/XXXX** |
| 322 | | C1, item 7.5  (Current Tt) | Acetaminophen (EFFERALGAN): start date = NA/NA/XXXX | Acetaminophen (EFFERALGAN): start date = **XX/XX/XXXX** |
| 324 | | C1, item 7.5  (Current Tt) | Chondroitin (STRUCTUM): start date = NA/XX/XXXX | Chondroitin (STRUCTUM): start date = **XX/XX/XXXX** |
| 342 | | C1, item 7.5  (Current Tt) | Tramadol + acetaminophen (IXPRIM): start date = NA/XX/XXXX | Tramadol + acetaminophen (IXPRIM): start date = **XX/XX/XXXX** |
| 345 | | C1, item 7.5  (Current Tt) | Acetaminophen (DOLIPRANE): start date = NA/NA/XXXX | Acetaminophen (DOLIPRANE): start date = **XX/XX/XXXX** |
| 345 | | C1, item 7.5  (Current Tt) | Chondroitin (CHONDROSULF): start date = NA/NA/XXXX | Chondroitin (CHONDROSULF): start date = **XX/XX/XXXX** |
| 346 | | C1, item 7.5  (Current Tt) | Acetaminophen (DOLIPRANE): start date = NA/NA/XXXX | Acetaminophen (DOLIPRANE): start date = **XX/XX/XXXX** |
| 347 | | C1, item 7.5  (Current Tt) | Tramadol: start date = NA/NA/XXXX | Tramadol: start date = **XX/XX/XXXX** |
| 347 | | C1, item 7.5  (Current Tt) | Cuivramine: start date = NA/NA/XXXX | Cuivramine: start date = **XX/XX/XXXX** |
| 347 | | C1, item 7.5  (Current Tt) | Chondroitin (CHONDROSULF): start date = NA/NA/XXXX | Chondroitin (CHONDROSULF): start date = **XX/XX/XXXX** |
| 348 | | C1, item 7.5  (Current Tt) | Chondroitin (STRUCTUM): start date = NA/NA/XXXX | Chondroitin (STRUCTUM): start date = **XX/XX/XXXX** |
| 351 | | C1, item 7.5  (Current Tt) | Diacerein (ART 50): start date = NA/NA/XXXX | Diacerein (ART 50): start date = **XX/XX/XXXX** |
| 358 | | C1, item 7.5  (Current Tt) | Acetaminophen (DOLIPRANE): start date = NA/XX/XXXX | Acetaminophen (DOLIPRANE): start date = **XX/XX/XXXX** |
| 358 | | C1, item 7.5  (Current Tt) | Diacerein (ART 50): start date = NA/XX/XXXX | Diacerein (ART 50): start date = **XX/XX/XXXX** |
| 361 | | C1, item 7.5  (Current Tt) | Chondroitin (CHONDROSULF): start date = NA/XX/XXXX | Chondroitin (CHONDROSULF): start date = **XX/XX/XXXX** |
| 362 | | C1, item 7.5  (Current Tt) | Glucosamine (DOLENIO): start date = NA/XX/XXXX | Glucosamine (DOLENIO): start date = **XX/XX/XXXX** |
| 365 | | C1, item 7.5  (Current Tt) | Acetaminophen (DOLIPRANE): start date = NA/XX/XXXX | Acetaminophen (DOLIPRANE): start date = **XX/XX/XXXX** |
| 383 | | C1, item 7.5  (Current Tt) | Acetaminophen: start date = NA/NA/XXXX | Acetaminophen: start date = **XX/XX/XXXX** |
| 386 | | C1, item 7.5  (Current Tt) | Chondroitin (CHONDROSULF): start date = NA/NA/XXXX | Chondroitin (CHONDROSULF): start date = **XX/XX/XXXX** |
| 387 | | C1, item 7.5  (Current Tt) | Piascledine: start date = NA/NA/XXXX | Piascledine: start date = **XX/XX/XXXX** |
| 394 | | C1, item 7.5  (Current Tt) | Acetaminophen (DAFALGAN): start date = NA/NA/XXXX | Acetaminophen (DAFALGAN): start date = **XX/XX/XXXX** |
| 395 | | C1, item 7.5  (Current Tt) | Dextropropoxyphene + acetaminophen+caffeine (PROPOFAN): start date = NA/NA/XXXX; end date = NA/NA/NA; ongoing = Yes | Dextropropoxyphene+acetaminophen + caffeine (PROPOFAN): start date = **01/01/2000**; end date = **XX/XX/XXXX**; ongoing = **NA** |
| 396 | | C1, item 7.5  (Current Tt) | Piascledine: start date = NA/NA/XXXX | Piascledine: start date = **XX/XX/XXXX** |
| 397 | | C1, item 7.5  (Current Tt) | Glucosamine (FLEXEA): start date = NA/XX/XXXX | Glucosamine (FLEXEA): start date = **XX/XX/XXXX** |
| 399 | | C1, item 7.5  (Current Tt) | Diacerein (ART 50): start date = NA/NA/XXXX | Diacerein (ART 50): start date = **XX/XX/XXXX** |
| 400 | | C1, item 7.5  (Current Tt) | Acetaminophen (EFFERALGAN): start date = NA/NA/XXXX | Acetaminophen (EFFERALGAN): start date = **XX/XX/XXXX** |
| 400 | | C1, item 7.5  (Current Tt) | Chondroitin (STRUCTUM): start date = NA/NA/XXXX | Chondroitin (STRUCTUM): start date = **XX/XX/XXXX** |
| 019 | | C3, item 16  (Change Concomitant Tt) | Tramadol (TOPALGIC): start date = NA/NA/XXXX | Tramadol (TOPALGIC): start date = **XX/XX/XXXX** |
| 344 | | C3, item 16  (Change Concomitant Tt) | Acetaminophen + caffeine (CLARADOL CAFEINE): start date = NA/XX/XXXX | Acetaminophen + caffeine (CLARADOL CAFEINE): start date = **XX/XX/XXXX** |
| 030 | | C3, item 18.3  (Local safety) | Septic arthritis = Yes | Septic arthritis = **No** |
| 051 | | C3, item 18.3  (Local safety) | Septic arthritis = Yes | Septic arthritis = **No** |
| 068 | | C3, item 18.3  (Local safety) | Septic arthritis = Yes | Septic arthritis = **No** |
| 153 | | C3, item 18.3  (Local safety) | Septic arthritis = Yes | Septic arthritis = **No** |
| 174 | | C3, item 18.3  (Local safety) | Septic arthritis = Yes | Septic arthritis = **No** |
| 201 | | C3, item 18.3  (Local safety) | Septic arthritis = Yes | Septic arthritis = **No** |
| 317 | | C3, item 18.3  (Local safety) | Septic arthritis = Yes | Septic arthritis = **No** |
| 331 | | C3, item 18.3  (Local safety) | Septic arthritis = Yes | Septic arthritis = **No** |
| 362 | | C3, item 18.3  (Local safety) | Septic arthritis = Yes | Septic arthritis = **No** |
| 395 | | C3, item 18.3  (Local safety) | Septic arthritis = Yes | Septic arthritis = **No** |
| 398 | | C3, item 18.3  (Local safety) | Septic arthritis = Yes | Septic arthritis = **No** |
| 018 | | C4, item 26  (Change Concomitant Tt) | Piroxicam (FELDENE): start date = XX/XX/XXXX | Piroxicam (FELDENE): start date = XX/XX/XXXX |
| 365 | | C4, item 26  (Change Concomitant Tt) | Prednisone: start date = XX/XX/XXXX | Prednisone: start date = XX/XX/XXXX |
| 166 | | C5, item 35  (Change Concomitant Tt) | Diclofenac (VOLTARENE): start date = XX/XX/XXXX | Diclofenac (VOLTARENE): start date = XX/XX/XXXX |
| 390 | | AE, item 43  (Date) | Start date = XX/XX/XXXX | Start date = XX/XX/XXXX |
| 096 | | AE, item 43  (Tt) | Treatment: Puncture | Treatment: **Puncture on** XX/XX/XXXX |
| 097 | | AE, item 43  (Tt) | Treatment: cortivazol (ALTIM) | Treatment: cortivazol (ALTIM) **injected on XX/XX/XXXX** |
| 108 | | AE, item 43  (Tt) | Major acute inflammation in the studied knee: not recorded | Major acute inflammation in the studied knee: date adverse event = **XX/XX/XXXX**; ongoing = **Yes;** start = **2** (after injection); medical intervention = **1** (treatment); comments = **2** (stop the trial); treatment: **cortivazol (ALTIM) injected on XX/XX/XXXX** |
| 019 | | Study population | PP | **FAS** |
| 028 | | Study population | FAS | **PP** |
| 064 | | Study population | PP | **FAS** |
| 068 | | Study population | PP | **FAS** |
| 073 | | Study population | PP | **FAS** |
| 075 | | Study population | PP | **FAS** |
| 094 | | Study population | PP | **FAS** |
| 108 | | Study population | ITT | **FAS** |
| 128 | | Study population | PP | **FAS** |
| 142 | | Study population | FAS | **ITT** |
| 178 | | Study population | PP | **FAS** |
| 191 | | Study population | FAS | **PP** |
| 246 | | Study population | FAS | **PP** |
| 300 | | Study population | FAS | **PP** |
| 320 | | Study population | PP | **FAS** |
| 323 | | Study population | ITT | **FAS** |
| 341 | | Study population | PP | **FAS** |
| 342 | | Study population | PP | **FAS** |
| 344 | | Study population | PP | **FAS** |
| 347 | | Study population | PP | **FAS** |
| 358 | | Study population | ITT | **FAS** |
| 360 | | Study population | FAS | **PP** |

AE = adverse event; C = Consultation; cortico. = corticosteroids; crit. = criteria; D = Day; FAS = Full Analysis Set; HA = hyaluronic acid; ITT = Intention-to-Treat; NA = not available; NSAID = non-steroidal anti-inflammatory drug; PP = Per Protocol; Tt = treatment, X = confidential information.
