# Supplementary material for: A single intra-articular injection of 2.0% non-chemically modified sodium hyaluronate vs 0.8% hylan G-F 20 in the treatment of symptomatic knee osteoarthritis: A 6-month, multicenter, randomized, controlled non-inferiority trial
Source: PLoS One. 2019 Dec 10;14(12):e0226007. doi: 10.1371/journal.pone.0226007 (PMC6903764; doi:10.1371/journal.pone.0226007)
Supplement: S2 Appendix — (DOCX) [file pone.0226007.s005.docx]

**S2 Appendix. Adjustments for covariates.**

An imbalance in the male/female ratio between the two treatment groups was revealed (*P* = 0.03). Its influence on the primary efficacy outcome (difference between the two treatment groups regarding the change from baseline in WOMAC A at D180) was analyzed (Per Protocol dataset only).

The following two hypotheses were envisaged to explore whether the imbalance in sex ratio influenced the observed result:

- Difference between men and women in the quantity of effects observed;
- Sex-treatment interaction, i.e., difference of effects per product as a function of sex.

The table below shows the differences of efficacy between the two treatment groups in men, in women and regardless of sex.

Differences of efficacy between the two treatment groups according to sex (Per Protocol dataset).

| **Sex** | **Difference** **(absolute values)** | **95% confidence interval** | ***P*** |
| --- | --- | --- | --- |
| Male | -1.6 | (-10.8, 7.6) | 0.7 |
| Female | -1.7 | (-8.6, 5.2) | 0.6 |
| All | -1.9 | (-7.3, 3.5) | 0.5 |

The table below presents the analysis of the interaction between sex and difference of efficacy between the two treatment groups.

Analysis of the interaction between sex and difference of efficacy between the two treatment groups.

| **Variable** | **Difference** **(absolute values)** | **Standard deviation** | **T** | ***P*>\|t\|** | **95% confidence interval** |
| --- | --- | --- | --- | --- | --- |
| Sodium hyaluronate minus hylan G-F 20 | -1.6 | 4.6 | 0.45 | 0.7 | (-10.7, 7.5) |
| Sex | -1.6 | 3.9 | 0.40 | 0.7 | (-9.3, 6.2) |
| Interaction | 0.1 | 5.8 | 0.02 | >0.9 | (-11.3, 11.5) |
| Constant | -37.0 | 2.9 | -12.58 | 0.0 | (-42.8, -31.2) |

The results of the analysis show that the interaction between sex and difference of efficacy between the two treatment groups was not significant (*P* >0.9).

Finally the comparison of efficacy when adjusted for sex was not significant:

- Unadjusted difference: -1.9 (95% CI : -7.3, 3.5), *P* = 0.5
- Difference adjusted for sex: -1.7 (95% CI: -7.1, 3.8), *P* = 0.6

In conclusion the imbalance in the male/female ratio observed between the treatment groups had no significant effect on the primary efficacy outcome of the study.
