# Supplementary material for: A single intra-articular injection of 2.0% non-chemically modified sodium hyaluronate vs 0.8% hylan G-F 20 in the treatment of symptomatic knee osteoarthritis: A 6-month, multicenter, randomized, controlled non-inferiority trial
Source: PLoS One. 2019 Dec 10;14(12):e0226007. doi: 10.1371/journal.pone.0226007 (PMC6903764; doi:10.1371/journal.pone.0226007)
Supplement: S1 Table — (DOCX) [file pone.0226007.s006.docx]

**S1 Table. Patients who discontinued the trial or were lost to follow-up (Intention-to-Treat population).**

| **Patient** | **Group** | **Dataset** | **Age** | **Sex** | **Description** | **Injection** | | **Left trial** | **Last visit** | | **Reason(s) given** |
| --- | --- | --- | --- | --- | --- | --- | --- | --- | --- | --- | --- |
| 025 | SH | FAS | 74 | Male | Patient chose to leave the study without giving a reason 92 days post injection, i.e., 61 days post C3. | Yes | D0 | D92 | C3 | D31 | Consent withdrawal |
| 026 | SH | FAS | 82 | Female | Patient chose to leave the study without giving a reason 122 days post injection, i.e., 30 days post C4. | Yes | D0 | D122 | C4 | D92 | Consent withdrawal |
| 034 | SH | ITT | 62 | Male | Patient did not attend the injection visit C2 and therefore did not receive an injection. He left the trial 3 days post C1. | No | NA | D3 | C1 | D0 | Consent withdrawal |
| 095 | SH | ITT | 55 | Female | After the last visit completed (C2) the evaluating investigator heard nothing further from the patient. She was therefore withdrawn from the study 250 days post injection. | Yes | D0 | D250 | C2 | D0 | Lost to follow-up |
| 097 | SH | ITT | 60 | Female | Patient developed an effusion in the studied knee (AE appeared outside the treatment) 2 days before C2, which was treated by puncture of knee and infiltration of cortivazol on the same day. The injecting investigator then decided not to proceed to injection with the trial drug. | No | NA | D6 | C2 | D6 | Adverse event  (Withdrawal by injector) |
| 117 | SH | FAS | 64 | Female | Patient received an infiltration of cortivazol (ALTIM) in the studied knee (arthralgia) 90 days post injection, i.e., at C4. | Yes | D0 | D90 | C4 | D90 | Forbidden treatment |
| 311 | SH | FAS | 53 | Female | Patient chose to leave the study without giving a reason 90 days post injection, i.e., at C3. | Yes | D1 | D91 | C3 | D91 | Consent withdrawal |
| 358 | SH | FAS | 62 | Female | Patient took nabumetone (NABUCOX) as continuous treatment (arthralgia) from 19 days post injection until 4 days before C3 (87 days before C4) (10 days’ treatment). She then received 2 x 10-day courses of diclofenac (VOLTAREN) (arthralgia) from 36 days post injection until 30 days before C4. Patient also received an infiltration of cortivazol (ALTIM) in the studied knee (arthralgia) 81 days post injection, i.e., 25 days before C4. The trial treatment was considered to be ineffective. | Yes | D0 | D106 | C4 | D106 | Lack of efficacy  (Forbidden treatment) |
| 362 | SH | FAS | 60 | Female | Patient lost to follow-up 98 days post injection, i.e., 63 days post C3. | Yes | D0 | D98 | C3 | D35 | Lost to follow-up |
| 391 | SH | ITT | 73 | Female | Patient chose to leave the study without giving a reason 24 days post injection. | Yes | D0 | D24 | C2 | D0 | Consent withdrawal |
| 018 | Control | FAS | 54 | Male | Patient received an infiltration of cortivazol (ALTIM) in the studied knee (arthralgia) 85 days post injection, i.e., 7 days before C4. | Yes | D0 | D92 | C4 | D92 | Forbidden treatment |
| 108 | Control | FAS | 53 | Female | Patient began treatment with ibuprofen 29 days post injection, i.e., 6 days before C3. She then suffered an osteoarthritis flare up (osteoarthritis, AE) of the studied knee 35 days post injection requiring puncture of the knee and infiltration of cortivazol (ALTIM) 4 days later. | Yes | D0 | D35 | C3 | D35 | Adverse event  (Forbidden treatment) |
| 111 | Control | ITT | 86 | Female | Patient moved address temporarily and therefore chose to leave the study 90 days post injection. | Yes | D1 | D91 | C2 | D1 | Consent withdrawal |
| 142 | Control | ITT | 80 | Female | Evaluating investigator was admitted to hospital after C3. | Yes | D5 | NA | C3 | D30 | Evaluator withdrawal |
| 173 | Control | FAS | 71 | Female | Patient took diclofenac (VOLTAREN) as continuous treatment (arthralgia) from 67 days post injection until 7 days before C4 (19 days of treatment). The trial treatment was considered to be ineffective. | Yes | D0 | D92 | C4 | D92 | Lack of efficacy (Forbidden treatment) |
| 187 | Control | FAS | 65 | Male | Patient chose to leave the study without giving a reason 124 days post injection, i.e., 92 days post C3. | Yes | D0 | D124 | C3 | D32 | Consent withdrawal |
| 221 | Control | ITT | 56 | Female | Patient was doing well and then chose to leave the study 41 days post injection. | Yes | D4 | D45 | C2 | D4 | Consent withdrawal |
| 227 | Control | FAS | 84 | Female | Last information about the patient was 90 days post injection, i.e., 63 days post C3. Since then, the evaluating investigator has heard no more. He therefore withdrew the patient from the study 224 days post injection, i.e., 197 days post C3. | Yes | D5 | D95 | C3 | D32 | Lost to follow-up |
| 230 | Control | FAS | 74 | Female | Patient suffered an arthrosis flare up (osteoarthritis, AE) of the studied knee 54 days post injection, i.e., 27 days post C3 which was treated by puncture of the knee and infiltration of steroids on the same day. | Yes | D2 | D56 | C3 | D29 | Adverse event (Forbidden treatment) (Withdrawal by evaluator) |
| 237 | Control | FAS | 51 | Female | Patient began treatment with NSAIDs 68 days post injection, i.e., 36 days post C3. | Yes | D0 | D68 | C3 | D32 | Forbidden treatment (Withdrawal by evaluator) |
| 261 | Control | ITT | 69 | Female | Patient refused injection at C2. | No | NA | D0 | C2 | D0 | Consent withdrawal |
| 269 | Control | ITT | 79 | Female | Patient was on oral anticoagulants (fluindione, PREVISCAN) and developed an effusion in the studied knee (AE appeared outside the treatment) at C2. The patient did not therefore receive the injection. | No | NA | D5 | C2 | D5 | Adverse event |
| 312 | Control | FAS | 68 | Female | After the last visit completed (C4 at D91) the evaluating investigator heard nothing further from the patient. He withdrew her from the study 257 days post injection, i.e., 167 days post C4. | Yes | D1 | D258 | C4 | D91 | Lost to follow-up |
| 323 | Control | FAS | 78 | Female | Patient received an infiltration of cortivazol (ALTIM) in the studied knee (arthralgia) 9 days post injection, i.e., 16 days before C3. Patient also took diclofenac (VOLTAREN) for 2 days (arthralgia) 2 days before this infiltration, i.e., 7 days post injection. | Yes | D0 | D25 | C3 | D25 | Forbidden treatment |
| 324 | Control | FAS | 55 | Female | Patient took diclofenac (VOLTAREN) for 2 days (arthralgia) 42 days post injection, i.e., 45 days before C4. She also received 13 days’ continuous treatment (arthralgia) with ketoprofen (PROFENID) from 57 days post injection until 18 days before C4. | Yes | D0 | D87 | C4 | D87 | Forbidden treatment |
| 350 | Control | ITT | 78 | Male | Development of fever, requiring admission to hospital 22 days post injection (SAE). | Yes | D1 | D35 | C2 | D1 | Adverse event |

AE = adverse event; C = Consultation; control = hylan G-F 20; D = Day; FAS = Full Analysis Set; ITT = Intention-to-Treat; NA = not applicable, SAE = serious adverse event; SH = sodium hyaluronate.
