# Supplementary material for: A single intra-articular injection of 2.0% non-chemically modified sodium hyaluronate vs 0.8% hylan G-F 20 in the treatment of symptomatic knee osteoarthritis: A 6-month, multicenter, randomized, controlled non-inferiority trial
Source: PLoS One. 2019 Dec 10;14(12):e0226007. doi: 10.1371/journal.pone.0226007 (PMC6903764; doi:10.1371/journal.pone.0226007)
Supplement: S2 Table — (DOCX) [file pone.0226007.s007.docx]

**S2 Table. Patients with protocol deviations (Intention-to-Treat population).**

| **Patient** | **Group** | **Sex** | **Age** | **Description** | **Deviation** | **Severity** | **Dataset** |
| --- | --- | --- | --- | --- | --- | --- | --- |
| 006 | SH | Male | 69 | C2 out of time (D5) | Follow-up visit out of time | Minor | PP |
| 008 | SH | Female | 71 | C2 out of time (D5) | Follow-up visit out of time | Minor | PP |
| 019 | SH | Female | 69 | Duration of NSAID treatment >5 days (diclofenac 25 days) Cortisone wash-out not respected at C5 | Duration of NSAID treatment not respected Wash-out not respected | Minor Major | FAS |
| 025 | SH | Male | 74 | C4 missing C5 missing | Follow-up visit not performed D180 follow-up visit not performed | Minor Major | FAS |
| 026 | SH | Female | 82 | C5 missing | D180 follow-up visit not performed | Major | FAS |
| 029 | SH | Male | 53 | BMI >30 kg/m^2^ (34.5) C2 out of time (D5) | Inclusion criterion not respected Follow-up visit out of time | Major Minor | FAS |
| 034 | SH | Male | 62 | WOMAC A post C1 absent No injection C2 missing C3 missing C4 missing C5 missing Acetaminophen wash-out not respected at C1 | No post injection primary criterion No study injection Follow-up visit not performed Follow-up visit not performed Follow-up visit not performed D180 follow-up visit not performed Wash-out not respected | Major+ Major+ Minor Minor Minor Major Major | ITT |
| 041 | SH | Female | 75 | C2 out of time (D5) | Follow-up visit out of time | Minor | PP |
| 052 | SH | Female | 78 | Forbidden treatment (naproxen for the studied knee) Duration of NSAID treatment >5 days (naproxen 27 days) Naproxen wash-out not respected at C4 | Forbidden treatment Duration of NSAID treatment not respected Wash-out not respected | Minor Minor Minor | PP |
| 055 | SH | Male | 69 | C2 out of time (D6) | Follow-up visit out of time | Minor | PP |
| 064 | SH | Female | 84 | Forbidden concomitant disease at inclusion (rhizomelic pseudopolyarthritis) Prednisone wash-out not respected at C3 Prednisone wash-out not respected at C4 Prednisone wash-out not respected at C5 | Inclusion criterion not respected Wash-out not respected Wash-out not respected Wash-out not respected | Major Minor Minor Major | FAS |
| 068 | SH | Female | 72 | Time since last injection of HA <180 days (date of last injection not recorded) | Inclusion criterion not respected | Major | FAS |
| 075 | SH | Female | 65 | C5 out of time (D197) Tramadol+acetaminophen wash-out not respected at C3 Tramadol+acetaminophen wash-out not respected at C4 Tramadol+acetaminophen wash-out not respected at C5 | Follow-up visit out of time Wash-out not respected Wash-out not respected Wash-out not respected | Minor Minor Minor Major | FAS |
| 076 | SH | Female | 50 | ACR criteria not respected (age = 50 years, morning stiffness ≥30 min, no crepitation) C5 out of time (D197) | Inclusion criterion not respected  Follow-up visit out of time | Minor  Minor | PP |
| 089 | SH | Male | 62 | Forbidden treatment (diclofenac for the studied knee) Duration of NSAID treatment >5 days (diclofenac 14 days) Prednisolone wash-out not respected at C4 | Forbidden treatment Duration of NSAID treatment not respected Wash-out not respected | Minor Minor Minor | PP |
| 094 | SH | Female | 69 | Piroxicam wash-out not respected at C1 | Wash-out not respected | Major | FAS |
| 095 | SH | Female | 55 | WOMAC A post C1 absent C3 missing C4 missing C5 missing | No post injection primary criterion Follow-up visit not performed Follow-up visit not performed D180 follow-up visit not performed | Major+ Minor Minor Major | ITT |
| 097 | SH | Female | 60 | BMI >30 kg/m^2^ (30.9) WOMAC A post C1 absent No injection C3 missing C4 missing C5 missing C2 out of time (D6) Forbidden treatment (cortivazol for the studied knee 2 days before C2) | Inclusion criterion not respected No post injection primary criterion No study injection Follow-up visit not performed Follow-up visit not performed D180 follow-up visit not performed Follow-up visit out of time Forbidden treatment | Minor Major+ Major+ Minor Minor Major Minor Major | ITT |
| 110 | SH | Female | 75 | WOMAC A <40 mm (37) | Inclusion criterion not respected | Minor | PP |
| 112 | SH | Female | 80 | WOMAC A <40 mm (31) C4 missing C5 out of time (D206) | Inclusion criterion not respected Follow-up visit not performed Follow-up visit out of time | Major Minor Minor | FAS |
| 115 | SH | Female | 62 | C3 missing C4 missing C5 out of time (D271) | Follow-up visit not performed Follow-up visit not performed Follow-up visit out of time | Minor Minor Major | FAS |
| 117 | SH | Female | 64 | C5 missing | D180 follow-up visit not performed | Major | FAS |
| 136 | SH | Female | 57 | C2 out of time (D5) | Follow-up visit out of time | Minor | PP |
| 139 | SH | Female | 75 | WOMAC A difference between two knees <20 mm (18) | Inclusion criterion not respected | Minor | PP |
| 141 | SH | Male | 61 | C4 missing | Follow-up visit not performed | Minor | PP |
| 143 | SH | Female | 43 | C2 out of time (D6) | Follow-up visit out of time | Minor | PP |
| 151 | SH | Female | 75 | C5 out of time (D201) Acetaminophen wash-out not respected at C3 Acetaminophen wash-out not respected at C4 | Follow-up visit out of time Wash-out not respected Wash-out not respected | Minor Minor Minor | PP |
| 170 | SH | Female | 80 | C5 out of time (D204) | Follow-up visit out of time | Minor | PP |
| 179 | SH | Female | 62 | C5 out of time (D196) Forbidden treatment (etoricoxib for the studied knee) Duration of NSAID treatment >5 days (etoricoxib 6-10 days) Etoricoxib wash-out not respected at C3 Etoricoxib wash-out not respected at C4 | Follow-up visit out of time Forbidden treatment Duration of NSAID treatment not respected Wash-out not respected Wash-out not respected | Minor Minor Minor Minor Minor | PP |
| 191 | SH | Male | 70 | C4 out of time (D122) C5 out of time (D214) | Follow-up visit out of time Follow-up visit out of time | Minor Minor | PP |
| 196 | SH | Female | 60 | C4 out of time (D119) C5 out of time (D208) | Follow-up visit out of time Follow-up visit out of time | Minor Minor | PP |
| 198 | SH | Female | 52 | C2 out of time (D6) C5 out of time (D196) | Follow-up visit out of time Follow-up visit out of time | Minor Minor | PP |
| 200 | SH | Female | 71 | BMI >30 kg/m^2^ (30.8) Time since X-ray >365 days (381) C2 out of time (D21) C3 out of time (D48) C4 out of time (D109) C5 out of time (D200) | Inclusion criterion not respected Inclusion criterion not respected Follow-up visit out of time Follow-up visit out of time Follow-up visit out of time Follow-up visit out of time | Minor Minor Major Minor Minor Minor | FAS |
| 202 | SH | Male | 66 | Forbidden treatment (cortivazol for the studied knee 28 days before C5) | Forbidden treatment | Major | FAS |
| 204 | SH | Female | 71 | C5 out of time (D196) | Follow-up visits out of time | Minor | PP |
| 210 | SH | Female | 74 | Forbidden treatment (cortivazol for scapulohumeral periarthritis 60 days before C5) Duration of NSAID treatment >5 days (etoricoxib 10-11 days) | Forbidden treatment  Duration of NSAID treatment not respected | Minor  Minor | PP |
| 217 | SH | Female | 63 | C4 out of time (D112) | Follow-up visit out of time | Minor | PP |
| 226 | SH | Female | 54 | WOMAC A <40 mm (20) Forbidden treatment (diclofenac for the studied knee) Duration of NSAID treatment >5 days (diclofenac) Diclofenac wash-out not respected at C5 | Inclusion criterion not respected Forbidden treatment Duration of NSAID treatment not respected Wash-out not respected | Major Minor Minor Major | FAS |
| 228 | SH | Female | 72 | WOMAC A <40 mm (34) C2 out of time (D7) Duration of NSAID treatment >5 days (celecoxib 17-97 days) Celecoxib wash-out not respected at C4 Celecoxib wash-out not respected at C5 | Inclusion criterion not respected Follow-up visit out of time Duration of NSAID treatment not respected Wash-out not respected Wash-out not respected | Major Minor Minor Minor Major | FAS |
| 233 | SH | Female | 56 | Acetaminophen wash-out not respected at C4 | Wash-out not respected | Minor | PP |
| 245 | SH | Male | 72 | C5 out of time (D202) | Follow-up visit out of time | Minor | PP |
| 248 | SH | Female | 62 | C4 out of time (D115) C5 out of time (D206) | Follow-up visit out of time Follow-up visit out of time | Minor Minor | PP |
| 249 | SH | Female | 60 | WOMAC A difference between two knees <20 mm (12) C4 out of time (D108) C5 out of time (D197) | Inclusion criterion not respected Follow-up visit out of time Follow-up visit out of time | Major Minor Minor | FAS |
| 279 | SH | Female | 41 | WOMAC A difference between two knees <20 mm (9) | Inclusion criterion not respected | Major | FAS |
| 281 | SH | Male | 67 | C4 out of time (D112) C5 out of time (D201) | Follow-up visit out of time Follow-up visit out of time | Minor Minor | PP |
| 282 | SH | Female | 79 | Forbidden concomitant disease at inclusion (congestive episode) C4 out of time (D119) C5 out of time (D210) Forbidden treatment (ketoprofen for the studied knee) Forbidden treatment (tramadol+acetaminophen for the studied knee) Duration of NSAID treatment >5 days (ketoprofen 7-19 days) Tramadol+acetaminophen wash-out not respected at C3 | Inclusion criterion not respected Follow-up visit out of time Follow-up visit out of time Forbidden treatment Forbidden treatment Duration of NSAID treatment not respected Wash-out not respected | Major Minor Minor Minor Minor Minor Minor | FAS |
| 290 | SH | Male | 66 | C4 out of time (D140) C5 out of time (D227) Tramadol wash-out not respected at C3 Tramadol wash-out not respected at C4 | Follow-up visit out of time Follow-up visit out of time Wash-out not respected Wash-out not respected | Minor Major Minor Minor | FAS |
| 292 | SH | Female | 60 | C5 out of time (D199) | Follow-up visit out of time | Minor | PP |
| 303 | SH | Female | 81 | Forbidden treatment (cortivazol for scapular pain 56 days before C4) Duration of NSAID treatment >5 days (naproxen 29 days) | Forbidden treatment Duration of NSAID treatment not respected | Minor Minor | PP |
| 307 | SH | Female | 68 | Time since X-ray >365 days (615) | Inclusion criterion not respected | Minor | PP |
| 311 | SH | Female | 53 | C4 missing C5 missing C3 out of time (D91) Tramadol+acetaminophen wash-out not respected at C3 | Follow-up visit not performed D180 follow-up visit not performed Follow-up visit out of time Wash-out not respected | Minor Major Minor Minor | FAS |
| 321 | SH | Male | 56 | Time since last HA injection <180 days (168) Acetaminophen wash-out not respected at C3 | Inclusion criterion not respected Wash-out not respected | Minor Minor | PP |
| 327 | SH | Male | 39 | Age <40 or >85 years (39) C5 out of time (D204) | Inclusion criterion not respected Follow-up visit out of time | Minor Minor | PP |
| 329 | SH | F | 75 | C2 out of time (D7) | Follow-up visit out of time | Minor | PP |
| 334 | SH | Male | 84 | C5 out of time (D208) | Follow-up visit out of time | Minor | PP |
| 335 | SH | Female | 61 | BMI >30 kg/m^2^ (31.8) C5 out of time (D204) | Inclusion criterion not respected Follow-up visit out of time | Minor Minor | PP |
| 341 | SH | Female | 68 | Acetaminophen wash-out not respected at C5 | Wash-out not respected | Major | FAS |
| 342 | SH | Female | 81 | C5 out of time (D199) Tramadol+acetaminophen wash-out not respected at C3 Tramadol+acetaminophen wash-out not respected at C4 Tramadol+acetaminophen wash-out not respected at C5 Acetaminophen wash-out not respected at C5 | Follow-up visit out of time Wash-out not respected Wash-out not respected Wash-out not respected Wash-out not respected | Minor Minor Minor Major Major | FAS |
| 347 | SH | Female | 64 | Tramadol wash-out not respected at C3 Tramadol wash-out not respected at C4 Tramadol wash-out not respected at C5 | Wash-out not respected Wash-out not respected Wash-out not respected | Minor Minor Major | FAS |
| 358 | SH | Female | 62 | C5 missing C4 out of time (106) Forbidden treatment (cortivazol 25 days before C4 for the studied knee) Forbidden treatment (diclofenac for the studied knee) Forbidden treatment (nabumetone for knee in question) Duration of NSAID treatment >5 days (diclofenac 10 days) Duration of NSAID treatment >5 days (nabumetone 10 days) Nabumetone wash-out not respected at C3 | D180 follow-up visit not performed Follow-up visit out of time Forbidden treatment Forbidden treatment Forbidden treatment Duration of NSAID treatment not respected Duration of NSAID treatment not respected Wash-out not respected | Major Minor Major Minor Minor Minor Minor Minor | FAS |
| 360 | SH | Female | 72 | C5 out of time (D214) Duration of NSAID treatment >5 days (celecoxib 10-1041 days) | Follow-up visit out of time Duration of NSAID treatment not respected | Minor Minor | PP |
| 362 | SH | Female | 60 | C4 missing C5 missing | Follow-up visit not performed D180 follow-up visit not performed | Minor Major | FAS |
| 366 | SH | Male | 70 | Baseline WOMAC A absent Time since X-ray >365 days (1173) C2 out of time (D7) C5 out of time (D225) | No baseline primary criterion Inclusion criterion not respected Follow-up visit out of time Follow-up visit out of time | Major Major Minor Major | ITT |
| 369 | SH | Female | 57 | Duration of NSAID treatment >5 days (aceclofenac 18-46 days) Aceclofenac wash-out not respected at C3 | Duration of NSAID treatment not respected Wash-out not respected | Minor Minor | PP |
| 373 | SH | Male | 46 | WOMAC A difference between two knees <20 mm (18), X-ray after C1 (+12 days) | Inclusion criterion not respected | Minor | PP |
| 375 | SH | Female | 53 | C5 out of time (D208) | Follow-up visit out of time | Minor | PP |
| 382 | SH | Female | 65 | C5 out of time (D257) | Follow-up visit out of time | Major | FAS |
| 383 | SH | Female | 70 | C5 out of time (D203) Forbidden treatment (ketoprofen for the studied knee) Duration of NSAID treatment >5 days (ketoprofen 14 days) | Follow-up visit out of time Forbidden treatment Duration of NSAID treatment not respected | Minor Minor Minor | PP |
| 385 | SH | Female | 63 | C5 out of time (D197) | Follow-up visit out of time | Minor | PP |
| 391 | SH | Female | 73 | WOMAC A post C1 absent C3 missing C4 missing C5 missing | No post injection primary criterion Follow-up visit not performed Follow-up visit not performed D180 follow-up visit not performed | Major+ Minor Minor Major | ITT |
| 392 | SH | Female | 64 | C5 out of time (D152) | Follow-up visit out of time | Minor | PP |
| 396 | SH | Female | 61 | Ibuprofen wash-out not respected at C3 | Wash-out not respected | Minor | PP |
| 397 | SH | Female | 63 | C5 out of time (D209) | Follow-up visit out of time | Minor | PP |
| 007 | Control | Female | 77 | BMI >30 kg/m^2^ (31.3) C2 out of time (D5) | Inclusion criterion not respected Follow-up visit out of time | Minor Minor | PP |
| 016 | Control | Female | 81 | Tramadol+acetaminophen wash-out not respected at C3 Tramadol+acetaminophen wash-out not respected at C4 Acetaminophen wash-out not respected at C4 | Wash-out not respected Wash-out not respected Wash-out not respected | Minor Minor Minor | PP |
| 017 | Control | Male | 69 | Forbidden treatment (ketoprofen for the studied knee) Duration of NSAID treatment >5 days (ketoprofen 8-50 days) Ketoprofen wash-out not respected at C3 | Forbidden treatment Duration of NSAID treatment not respected Wash-out not respected | Minor Minor Minor | PP |
| 018 | Control | Male | 54 | C5 missing Forbidden treatment (cortivazol for the studied knee 7 days before C4) Duration of NSAID treatment >5 days (piroxicam 6-26 days) Tramadol wash-out not respected at C3 Tramadol wash-out not respected at C4 Piroxicam wash-out not respected at C3 Piroxicam wash-out not respected at C4 Acetaminophen wash-out not respected at C3 | D180 follow-up visit not performed Forbidden treatment Duration of NSAID treatment not respected Wash-out not respected Wash-out not respected Wash-out not respected Wash-out not respected Wash-out not respected | Major Major Minor Minor Minor Minor Minor Minor | FAS |
| 021 | Control | Female | 79 | C5 out of time (D210) Duration of NSAID treatment >5 days (ketoprofen 8 days) | Follow-up visit out of time Duration of NSAID treatment not respected | Minor Minor | PP |
| 027 | Control | Female | 79 | C5 out of time (D209) | Follow-up visit out of time | Minor | PP |
| 028 | Control | Male | 74 | C4 missing C5 out of time (D211) | Follow-up visit not performed Follow-up visit out of time | Minor Minor | PP |
| 033 | Control | Female | 60 | C4 out of time (D132) | Follow-up visit out of time | Minor | PP |
| 043 | Control | Male | 55 | Time to last corticosteroid injection <60 days (59) | Inclusion criterion not respected | Minor | PP |
| 045 | Control | Male | 79 | WOMAC A <40 mm (39) WOMAC A difference <20 mm (16) | Inclusion criterion not respected Inclusion criterion not respected | Minor  Minor | PP |
| 049 | Control | Female | 65 | Acetaminophen wash-out not respected at C4 | Wash-out not respected | Minor | PP |
| 056 | Control | Female | 72 | C2 out of time (D7) | Follow-up visit out of time | Minor | PP |
| 061 | Control | Female | 72 | Duration of NSAID treatment >5 days (diclofenac 6 days) Duration of NSAID treatment >5 days (etoricoxib 24-62 days) Etoricoxib wash-out not respected at C3 Etoricoxib wash-out not respected at C4 | Duration of NSAID treatment not respected Duration of NSAID treatment not respected Wash-out not respected Wash-out not respected | Minor Minor Minor Minor | PP |
| 073 | Control | Female | 65 | C5 out of time (D197) Duration of NSAID treatment >5 days (ibuprofen) Ibuprofen wash-out not respected at C3 Ibuprofen wash-out not respected at C4 Ibuprofen wash-out not respected at C5 Tramadol wash-out not respected at C3 Tramadol wash-out not respected at C4 Tramadol wash-out not respected at C5 | Follow-up visit out of time Duration of NSAID treatment not respected Wash-out not respected Wash-out not respected Wash-out not respected Wash-out not respected Wash-out not respected Wash-out not respected | Minor Minor Minor Minor Major Minor Minor Major | FAS |
| 074 | Control | Female | 54 | C5 out of time (D197) | Follow-up visit out of time | Minor | PP |
| 082 | Control | Female | 69 | C5 out of time (D161) Acetaminophen wash-out not respected at C3 | Follow-up visit out of time Wash-out not respected | Minor Minor | PP |
| 085 | Control | Female | 68 | C2 out of time (D22) C5 out of time (D203) | Follow-up visit out of time Follow-up visit out of time | Major Minor | FAS |
| 090 | Control | Female | 65 | Duration of NSAID treatment >5 days (naproxen 6-18 days) Duration of NSAID treatment >5 days (ketoprofen 11 days) | Duration of NSAID treatment not respected Duration of NSAID treatment not respected | Minor Minor | PP |
| 091 | Control | Male | 51 | Forbidden treatment (triamcinolone hexacetonide for the studied knee 8 days before C5) Duration of NSAID treatment >5 days (tiaprofenic acid 6 days) | Forbidden treatment  Duration of NSAID treatment not respected | Major  Minor | FAS |
| 093 | Control | Female | 44 | C4 out of time (D140) C5 out of time (D219) | Follow-up visit out of time Follow-up visit out of time | Minor Major | FAS |
| 105 | Control | Male | 70 | Prednisone wash-out not respected at C3 | Wash-out not respected | Minor | PP |
| 108 | Control | Female | 53 | C4 missing C5 missing Forbidden treatment (ibuprofen for the studied knee) Duration of NSAID treatment >5 days (ibuprofen) Ibuprofen wash-out not respected at C3 | Follow-up visit not performed D180 follow-up visit not performed Forbidden treatment Duration of NSAID treatment not respected Wash-out not respected | Minor Major Minor Minor Minor | FAS |
| 109 | Control | Female | 80 | WOMAC A <40 mm (25) C4 missing C2 out of time (D7) C5 out of time (D210) Acetaminophen wash-out not respected at C3 Acetaminophen wash-out not respected at C5 | Inclusion criterion not respected Follow-up visit not performed Follow-up visit out of time Follow-up visit out of time Wash-out not respected Wash-out not respected | Major Minor Minor Minor Minor Major | FAS |
| 111 | Control | Female | 86 | Age <40 or >85 years (86) WOMAC A <40 mm (29) WOMAC A post C1 absent C3 missing C4 missing C5 missing | Inclusion criterion not respected Inclusion criterion not respected No post injection primary criterion Follow-up visit not performed Follow-up visit not performed D180 follow-up visit not performed | Minor Major Major+ Minor Minor Major | ITT |
| 114 | Control | Female | 73 | BMI >30 kg/m^2^ (30.8) C2 out of time (D5) C5 out of time (D252) Forbidden treatment (sodium hyaluronate in the contralateral knee 71 days before C5) | Inclusion criterion not respected Follow-up visit out of time Follow-up visit out of time Forbidden treatment | Minor Minor Major Major | FAS |
| 126 | Control | Female | 49 | Acetaminophen wash-out not respected at C4 | Wash-out not respected | Minor | PP |
| 128 | Control | Female | 47 | BMI >30 kg/m^2^ (30.9) Tramadol wash-out not respected at C5 | Inclusion criterion not respected Wash-out not respected | Minor Major | FAS |
| 134 | Control | Female | 53 | C5 out of time (D196) | Follow-up visit out of time | Minor | PP |
| 142 | Control | Female | 80 | BMI >30 kg/m^2^ (31.3) WOMAC A post C1 absent C4 missing C5 missing C2 out of time (D5) | Inclusion criterion not respected No post injection primary criterion Follow-up visit not performed D180 follow-up visit not performed Follow-up visit out of time | Minor Major+ Minor Major Minor | ITT |
| 153 | Control | Male | 59 | WOMAC A difference between two knees <20 mm (7) C4 out of time (D120) | Inclusion criterion not respected Follow-up visit out of time | Major Minor | FAS |
| 166 | Control | Female | 61 | Duration of NSAID treatment >5 days (diclofenac 6 days) | Duration of NSAID treatment not respected | Minor | PP |
| 171 | Control | Female | 61 | Duration of NSAID treatment >5 days (piroxicam 14 days and +) Duration of NSAID treatment >5 days (nabumetone 28 days) | Duration of NSAID treatment not respected Duration of NSAID treatment not respected | Minor Minor | PP |
| 173 | Control | Female | 71 | C5 missing Forbidden treatment (diclofenac for the studied knee) Duration of NSAID treatment >5 days (diclofenac 19 days) | D180 follow-up visit not performed Forbidden treatment Duration of NSAID treatment not respected | Major Minor Minor | FAS |
| 176 | Control | Female | 64 | C5 out of time (D153) Forbidden treatment (meniscus surgery 56 days before C5) Forbidden treatment (diclofenac for the studied knee) Duration of NSAID treatment >5 days (diclofenac 13 days) | Follow-up visit out of time Forbidden treatment Forbidden treatment Duration of NSAID treatment not respected | Minor Major Minor Minor | FAS |
| 178 | Control | Female | 77 | Duration of NSAID treatment >5 days (ketoprofen) Ketoprofen wash-out not respected at C3 Ketoprofen wash-out not respected at C4 Ketoprofen wash-out not respected at C5 Acetaminophen wash-out not respected at C5 | Duration of NSAID treatment not respected Wash-out not respected Wash-out not respected Wash-out not respected Wash-out not respected | Minor Minor Minor Major Major | FAS |
| 184 | Control | Female | 42 | C4 out of time (D66) | Follow-up visit out of time | Minor | PP |
| 187 | Control | Male | 65 | C4 missing C5 missing | Follow-up visit not performed D180 follow-up visit not performed | Minor Major | FAS |
| 190 | Control | Female | 63 | C4 out of time (D124) C5 out of time (D216) | Follow-up visit out of time Follow-up visit out of time | Minor Major | FAS |
| 197 | Control | Male | 65 | C2 out of time (D8) C5 out of time (D198) | Follow-up visit out of time Follow-up visit out of time | Minor Minor | PP |
| 199 | Control | Female | 62 | BMI >30 kg/m^2^ (31.6) | Inclusion criterion not respected | Minor | PP |
| 213 | Control | Male | 55 | BMI >30 kg/m^2^ (31.9) | Inclusion criterion not respected | Minor | PP |
| 221 | Control | Female | 56 | WOMAC A post C1 absent C3 missing C4 missing C5 missing | No post injection primary criterion Follow-up visit not performed Follow-up visit not performed D180 follow-up visit not performed | Major+ Minor Minor Major | ITT |
| 225 | Control | Female | 50 | Duration of NSAID treatment >5 days (celecoxib 8 days) | Duration of NSAID treatment not respected | Minor | PP |
| 227 | Control | Female | 84 | WOMAC A <40 mm (35) C4 missing C5 missing C2 out of time (D5) | Inclusion criterion not respected Follow-up visit not performed D180 follow-up visit not performed Follow-up visit out of time | Minor Minor Major Minor | FAS |
| 229 | Control | Female | 72 | WOMAC A difference between two knees <20 mm (19) C4 out of time (D106) C5 out of time (D248) | Inclusion criterion not respected Follow-up visit out of time Follow-up visit out of time | Minor Minor Major | FAS |
| 230 | Control | Female | 74 | Last X-ray at C1 (+2 days) C4 missing C5 missing | Inclusion criterion not respected Follow-up visit not performed D180 follow-up visit not performed | Minor Minor Major | FAS |
| 237 | Control | Female | 51 | C4 missing C5 missing | Follow-up visit not performed D180 follow-up visit not performed | Minor Major | FAS |
| 246 | Control | Female | 74 | C4 out of time (D115) C5 out of time (D213) | Follow-up visit out of time Follow-up visit out of time | Minor Minor | PP |
| 247 | Control | Male | 69 | Duration of NSAID treatment >5 days (celecoxib 6-163 days) Celecoxib wash-out not respected at C3 | Duration of NSAID treatment not respected Wash-out not respected | Minor Minor | PP |
| 261 | Control | Female | 69 | WOMAC post C1 absent No injection C3 missing C4 missing C5 missing | No post injection primary criterion No study injection Follow-up visit not performed Follow-up visit not performed D180 follow-up visit not performed | Major+ Major+ Minor Minor Major | ITT |
| 269 | Control | Female | 79 | WOMAC A post C1 absent No injection C3 missing C4 missing C5 missing C2 out of time (D5) | No post injection primary criterion No study injection Follow-up visit not performed Follow-up visit not performed D180 follow-up visit not performed Follow-up visit out of time | Major+ Major+ Minor Minor Major Minor | ITT |
| 276 | Control | Female | 66 | Duration of NSAID treatment >5 days (celecoxib 74 days) | Duration of NSAID treatment not respected | Minor | PP |
| 277 | Control | Female | 76 | Forbidden treatment (started chondroitin sulphate 96 days before C5 for spinal osteoarthritis) | Forbidden treatment | Major | FAS |
| 278 | Control | Female | 53 | C5 out of time (D196) | Follow-up visit out of time | Minor | PP |
| 283 | Control | Female | 72 | Time since X-ray >365 days (366) C5 out of time (D200) | Inclusion criterion not respected Follow-up visit out of time | Minor Minor | PP |
| 291 | Control | Male | 60 | C4 out of time (D112) C5 out of time (D206) | Follow-up visit out of time Follow-up visit out of time | Minor Minor | PP |
| 300 | Control | Male | 70 | C4 out of time (D125) C5 out of time (D213) | Follow-up visit out of time Follow-up visit out of time | Minor Minor | PP |
| 306 | Control | Female | 56 | BMI >30 kg/m^2^ (31.3) | Inclusion criterion not respected | Minor | PP |
| 310 | Control | Male | 72 | WOMAC A <40 mm (37) C5 out of time (D212) Forbidden treatment (arthroplasty of the studied knee 12 days before C5) Prednisone wash-out not respected at C4 | Inclusion criterion not respected Follow-up visit out of time Forbidden treatment Wash-out not respected | Minor Minor Major Minor | FAS |
| 312 | Control | Female | 68 | C5 missing | D180 follow-up visit not performed | Major | FAS |
| 317 | Control | Female | 65 | Forbidden treatment (piroxicam for the studied knee) Duration of NSAID treatment >5 days (piroxicam 7-10 days) | Forbidden treatment Duration of NSAID treatment not respected | Minor Minor | PP |
| 320 | Control | Male | 78 | Forbidden treatment (piroxicam for the studied knee) Duration of NSAID treatment >5 days (piroxicam 6-56 days) Duration of NSAID treatment >5 days (ketoprofen 6 days) Piroxicam wash-out not respected at C4 Piroxicam wash-out not respected at C5 | Forbidden treatment Duration of NSAID treatment not respected Duration of NSAID treatment not respected Wash-out not respected Wash-out not respected | Minor Minor Minor Minor Major | FAS |
| 323 | Control | Female | 78 | C4 missing C5 missing Forbidden treatment (cortivazol 16 days before C3 for the studied knee) Forbidden treatment (diclofenac for the studied knee) | Follow-up visit not performed D180 follow-up visit not performed Forbidden treatment Forbidden treatment | Minor Major Major Minor | FAS |
| 324 | Control | Female | 55 | C5 missing Forbidden treatment (diclofenac for the studied knee) Forbidden treatment (ketoprofen for the studied knee) Forbidden treatment (acetaminophen+codeine for the studied knee) Duration of NSAID treatment >5 days (ketoprofen 13 days) | D180 follow-up visit not performed Forbidden treatment Forbidden treatment Forbidden treatment Duration of NSAID treatment not respected | Major Minor Minor Minor Minor | FAS |
| 337 | Control | Male | 68 | C5 out of time (D196) | Follow-up visit out of time | Minor | PP |
| 339 | Control | Male | 66 | C3 out of time (D46) | Follow-up visit out of time | Minor | PP |
| 343 | Control | Female | 83 | WOMAC A difference between two knees <20 mm (19) | Inclusion criterion not respected | Minor | PP |
| 344 | Control | Female | 75 | Forbidden treatment (stopped chondroitin sulphate 72 days before C5) | Forbidden treatment | Major | FAS |
| 350 | Control | Male | 78 | WOMAC A post C1 absent C3 missing C4 missing C5 missing | No post injection primary criterion Follow-up visit not performed Follow-up visit not performed D180 follow-up visit not performed | Major+ Minor Minor Major | ITT |
| 365 | Control | Female | 75 | Baseline WOMAC A absent BMI >30 kg/m^2^ (33.3) C3 missing C5 out of time (D164) Prednisone wash-out not respected at C4 Acetaminophen wash-out not respected at C4 | No baseline primary criterion Inclusion criterion not respected Follow-up visit not performed Follow-up visit out of time Wash-out not respected Wash-out not respected | Major Major Minor Minor Minor Minor | ITT |
| 370 | Control | Female | 71 | C5 out of time (D164) | Follow-up visit out of time | Minor | PP |
| 374 | Control | Male | 67 | C3 out of time (D47) | Follow-up visit out of time | Minor | PP |
| 389 | Control | Female | 51 | C4 out of time (D152) C5 out of time (D217) | Follow-up visit out of time Follow-up visit out of time | Minor Major | FAS |
| 390 | Control | Male | 65 | Duration of NSAID treatment >5 days (aceclofenac 8 days) Dihydrocodeine wash-out not respected at C5 | Duration of NSAID treatment not respected Wash-out not respected | Minor Major | FAS |
| 398 | Control | Male | 50 | C5 out of time (D219) | Follow-up visit out of time | Major | FAS |

BMI = body mass index; C = Consultation; control = hylan G-F 20; D = Day; FAS = Full Analysis Set; ITT = Intention-to-Treat; NSAID = non-steroidal anti-inflammatory drug; PP = Per Protocol; SH = sodium hyaluronate; WOMAC A = Western Ontario and McMaster Universities Osteoarthritis Index pain subscale.
