# Supplementary material for: A single intra-articular injection of 2.0% non-chemically modified sodium hyaluronate vs 0.8% hylan G-F 20 in the treatment of symptomatic knee osteoarthritis: A 6-month, multicenter, randomized, controlled non-inferiority trial
Source: PLoS One. 2019 Dec 10;14(12):e0226007. doi: 10.1371/journal.pone.0226007 (PMC6903764; doi:10.1371/journal.pone.0226007)
Supplement: S3 Table — (DOCX) [file pone.0226007.s008.docx]

**S3 Table. Patient demographics and baseline characteristics (Full Analysis Set).**

| **Characteristic** | **SH**  n = 139 | **Control**  n = 141 | ***P*** |
| --- | --- | --- | --- |
| Female, n (%) | 101 (72.7) | 84 (59.6) | 0.02† |
| Age (years), mean (SD) | 67.3 (9.8) | 66.2 (10.6) | 0.4‡ |
| Body mass index (kg/m^2^), mean (SD) | 26.3 (3.0) | 26.3 (2.9) | 0.9‡ |
| Bilateral knee osteoarthritis, n (%) | 69 (49.6) | 74 (52.5) | 0.6† |
| Studied knee (right), n (%) | 82 (59.0) | 70 (49.6) | 0.1† |
| Bicompartmental knee osteoarthritis, n (%) | 40 (29.2) | 47 (33.3) | 0.5† |
| Associated patellofemoral pain syndrome, n (%) | 29 (20.9) | 39 (27.7) | 0.2† |
| Time since knee osteoarthritis diagnosis, n (%) |  |  | 0.7† |
| <1 year | 10 (7.2) | 10 (7.1) |  |
| ≥1 and <5 years | 56 (40.3) | 47 (33.3) |  |
| ≥5 and <10 years | 38 (27.3) | 43 (30.5) |  |
| ≥10 years | 35 (25.2) | 41 (29.1) |  |
| Modified Kellgren-Lawrence grade at studied knee |  |  | 0.3† |
| Grade Ib | 21 (15.1) | 24 (17.0) |  |
| Grade II | 74 (53.2) | 84 (59.6) |  |
| Grade III | 44 (31.7) | 33 (23.4) |  |
| WOMAC A (mm), mean (SD) | 58.2 (11.4) | 57.9 (11.6) | 0.9‡ |
| WOMAC B (mm), mean (SD) | 48.2 (20.8) | 48.3 (19.5) | >0.9‡ |
| WOMAC C (mm), mean (SD) | 46.8 (16.1) | 47.5 (14.9) | 0.7‡ |
| Lequesne index, mean (SD) | 11.6 (3.4) | 11.4 (3.2) | 0.5‡ |
| PtGA (mm), mean (SD) | 59.4 (16.4) | 59.1 (17.7) | 0.9‡ |

† Chi-square test; ‡ Student’s t-test.

Control = hylan G-F 20; PtGA = patient global assessment of disease activity; SD = standard deviation; SH = sodium hyaluronate; WOMAC A, B, C = Western Ontario and McMaster Universities Osteoarthritis Index pain, stiffness, function subscales, respectively.
