# Supplementary material for: A single intra-articular injection of 2.0% non-chemically modified sodium hyaluronate vs 0.8% hylan G-F 20 in the treatment of symptomatic knee osteoarthritis: A 6-month, multicenter, randomized, controlled non-inferiority trial
Source: PLoS One. 2019 Dec 10;14(12):e0226007. doi: 10.1371/journal.pone.0226007 (PMC6903764; doi:10.1371/journal.pone.0226007)
Supplement: S4 Table — (DOCX) [file pone.0226007.s009.docx]

**S4 Table. Patient demographics and baseline characteristics (Per Protocol dataset).**

| **Characteristic** | **SH**  n = 113 | **Control**  n = 112 | ***P*** |
| --- | --- | --- | --- |
| Female, n (%) | 79 (69.9) | 63 (56.3) | 0.03† |
| Age (years), mean (SD) | 67.4 (9.7) | 66.6 (10.4) | 0.6‡ |
| Body mass index (kg/m^2^), mean (SD) | 26.1 (3.0) | 26.3 (2.9) | 0.8‡ |
| Bilateral knee osteoarthritis, n (%) | 57 (50.4) | 62 (55.4) | 0.5† |
| Studied knee (right), n (%) | 69 (61.1) | 58 (51.8) | 0.2† |
| Bicompartmental knee osteoarthritis, n (%) | 30 (27.0) | 35 (31.3) | 0.5† |
| Associated patellofemoral pain syndrome, n (%) | 20 (17.7) | 30 (26.8) | 0.1† |
| Time since knee osteoarthritis diagnosis, n (%) |  |  | 0.5† |
| <1 year | 10 (8.8) | 10 (8.9) |  |
| ≥1 and <5 years | 46 (40.7) | 37 (33.0) |  |
| ≥5 and <10 years | 33 (29.2) | 33 (29.5) |  |
| ≥10 years | 24 (21.2) | 32 (28.6) |  |
| Modified Kellgren-Lawrence grade at studied knee |  |  | 0.08† |
| Grade Ib | 16 (14.2) | 21 (18.8) |  |
| Grade II | 60 (53.1) | 69 (61.6) |  |
| Grade III | 37 (32.7) | 22 (19.6) |  |
| WOMAC A (mm), mean (SD) | 57.9 (10.9) | 58.8 (10.9) | 0.5‡ |
| WOMAC B (mm), mean (SD) | 46.4 (20.6) | 48.6 (19.7) | 0.4‡ |
| WOMAC C (mm), mean (SD) | 44.8 (15.7) | 47.8 (14.1) | 0.1‡ |
| Lequesne index, mean (SD) | 11.3 (3.2) | 11.2 (3.3) | 0.9‡ |
| PtGA (mm), mean (SD) | 58.8 (16.5) | 57.4 (18.0) | 0.6‡ |

† Chi-square test; ‡ Student’s t-test.

Control = hylan G-F 20; PtGA = patient global assessment of disease activity; SD = standard deviation; SH = sodium hyaluronate; WOMAC A, B, C = Western Ontario and McMaster Universities Osteoarthritis Index pain, stiffness, function subscales, respectively.
