# Supplementary material for: A single intra-articular injection of 2.0% non-chemically modified sodium hyaluronate vs 0.8% hylan G-F 20 in the treatment of symptomatic knee osteoarthritis: A 6-month, multicenter, randomized, controlled non-inferiority trial
Source: PLoS One. 2019 Dec 10;14(12):e0226007. doi: 10.1371/journal.pone.0226007 (PMC6903764; doi:10.1371/journal.pone.0226007)
Supplement: S5 Table — (DOCX) [file pone.0226007.s010.docx]

**S5 Table. Individual patient demographics and baseline characteristics (Intention-to-Treat population).**

| **Patient** | **Group** | **Dataset** | **Age** | **Sex** | **Weight** **(kg)** | **Height** **(m)** | **BMI** **(kg/m^2^)** | **Laterality** | | **Studied knee** | **Compartmentality** | **Associated patellofemoral pain syndrome** | **Time since diagnosis (year)** | **Kellgren-Lawrence grade** |
| --- | --- | --- | --- | --- | --- | --- | --- | --- | --- | --- | --- | --- | --- | --- |
| 002 | SH | PP | 49 | Female | 81 | 1.67 | 29 | | Bilateral | Left | Unicompartmental | No | 3 | Ib |
| 003 | SH | PP | 82 | Male | 83 | 1.80 | 26 | | Bilateral | Left | Unicompartmental | No | 15 | II |
| 006 | SH | PP | 69 | Male | 74 | 1.60 | 29 | | Unilateral | Right | Unicompartmental | No | 7 | II |
| 008 | SH | PP | 71 | Female | 74 | 1.58 | 30 | | Bilateral | Right | Bicompartmental | No | 6 | II |
| 009 | SH | PP | 84 | Male | 70 | 1.70 | 24 | | Unilateral | Right | Unicompartmental | No | 10 | II |
| 012 | SH | PP | 74 | Female | 55 | 1.60 | 21 | | Unilateral | Right | Unicompartmental | No | 20 | II |
| 014 | SH | PP | 67 | Female | 60 | 1.56 | 25 | | Unilateral | Left | Unicompartmental | No | 11 | II |
| 015 | SH | PP | 54 | Female | 65 | 1.60 | 25 | | Unilateral | Right | Unicompartmental | No | 1 | II |
| 019 | SH | FAS | 69 | Female | 73 | 1.58 | 29 | | Unilateral | Right | Unicompartmental | No | 10 | II |
| 020 | SH | PP | 80 | Female | 69 | 1.55 | 29 | | Bilateral | Left | Unicompartmental | No | 10 | III |
| 022 | SH | PP | 77 | Female | 71 | 1.60 | 28 | | Bilateral | Right | Unicompartmental | No | 10 | III |
| 023 | SH | PP | 78 | Female | 75 | 1.63 | 28 | | Unilateral | Right | Bicompartmental | Yes | 9 | III |
| 025 | SH | FAS | 74 | Male | 86 | 1.77 | 27 | | Unilateral | Right | Bicompartmental | Yes | 10 | III |
| 026 | SH | FAS | 82 | Female | 64 | 1.58 | 26 | | Bilateral | Right | Bicompartmental | No | 8 | III |
| 029 | SH | FAS | 53 | Male | 111 | 1.79 | 35 | | Unilateral | Left | Bicompartmental | No | 26 | III |
| 034 | SH | ITT | 62 | Male | 90 | 1.75 | 29 | | Unilateral | Right | Unicompartmental | No | 9 | II |
| 037 | SH | PP | 73 | Male | 82 | 1.74 | 27 | | Unilateral | Right | Unicompartmental | No | 1 | III |
| 040 | SH | PP | 60 | Male | 74 | 1.76 | 24 | | Unilateral | Right | Unicompartmental | No | 0 | II |
| 041 | SH | PP | 75 | Female | 64 | 1.60 | 25 | | Unilateral | Right | Unicompartmental | No | 1 | II |
| 044 | SH | PP | 62 | Female | 62 | 1.64 | 23 | | Unilateral | Right | Unicompartmental | No | 2 | III |
| 048 | SH | PP | 69 | Female | 69 | 1.69 | 24 | | Bilateral | Left | Unicompartmental | No | 0 | II |
| 050 | SH | PP | 65 | Female | 64 | 1.58 | 26 | | Bilateral | Right | Bicompartmental | No | 5 | III |
| 052 | SH | PP | 78 | Female | 86 | 1.73 | 29 | | Bilateral | Right | Unicompartmental | No | 5 | Ib |
| 053 | SH | PP | 59 | Male | 80 | 1.72 | 27 | | Unilateral | Right | Unicompartmental | No | 2 | II |
| 055 | SH | PP | 69 | Male | 83 | 1.90 | 23 | | Bilateral | Left | Bicompartmental | Yes | 4 | III |
| 057 | SH | PP | 70 | Male | 67 | 1.65 | 25 | | Bilateral | Right | Unicompartmental | No | 3 | II |
| 058 | SH | PP | 75 | Female | 68 | 1.63 | 26 | | Bilateral | Right | Unicompartmental | No | 4 | II |
| 063 | SH | PP | 82 | Female | 75 | 1.64 | 28 | | Bilateral | Left | Unicompartmental | No | 9 | II |
| 064 | SH | FAS | 84 | Female | 61 | 1.59 | 24 | | Bilateral | Right | Unicompartmental | No | 3 | Ib |
| 065 | SH | PP | 65 | Male | 68 | 1.70 | 24 | | Bilateral | Left | Unicompartmental | No | 2 | Ib |
| 068 | SH | FAS | 72 | Female | 58 | 1.56 | 24 | | Bilateral | Left | Unicompartmental | No | 10 | II |
| 070 | SH | PP | 66 | Male | 82 | 1.78 | 26 | | Unilateral | Right | Bicompartmental | Yes | 9 | II |
| 072 | SH | PP | 70 | Male | 73 | 1.70 | 25 | | Unilateral | Right | Unicompartmental | No | 7 | III |
| 075 | SH | FAS | 65 | Female | 80 | 1.70 | 28 | | Unilateral | Left | Bicompartmental | Yes | 2 | Ib |
| 076 | SH | PP | 50 | Female | 50 | 1.62 | 19 | | Unilateral | Right | Bicompartmental | Yes | 0 | Ib |
| 081 | SH | PP | 79 | Female | 66 | 1.49 | 30 | | Bilateral | Left | Unicompartmental | No | 0 | III |
| 089 | SH | PP | 62 | Male | 64 | 1.68 | 23 | | Bilateral | Right | Unicompartmental | No | 4 | III |
| 092 | SH | PP | 77 | Female | 72 | 1.66 | 26 | | Bilateral | Right | Unicompartmental | No | 2 | III |
| 094 | SH | FAS | 69 | Female | 80 | 1.66 | 29 | | Unilateral | Right | Unicompartmental | Yes | 2 | III |
| 095 | SH | ITT | 55 | Female | 72 | 1.58 | 29 | | Bilateral | Left | Unicompartmental | Yes | 4 | II |
| 097 | SH | ITT | 60 | Female | 84 | 1.65 | 31 | | Unilateral | Left | Unicompartmental | Yes | 10 | II |
| 101 | SH | PP | 81 | Female | 52 | 1.41 | 26 | | Bilateral | Left | Unicompartmental | No | 3 | III |
| 102 | SH | PP | 51 | Female | 64 | 1.62 | 24 | | Unilateral | Right | Unicompartmental | No | 6 | II |
| 106 | SH | PP | 64 | Female | 65 | 1.57 | 26 | | Unilateral | Right | Unicompartmental | No | 2 | Ib |
| 107 | SH | PP | 65 | Female | 70 | 1.65 | 26 | | Unilateral | Left | Bicompartmental | Yes | 4 | III |
| 110 | SH | PP | 75 | Female | 82 | 1.66 | 30 | | Unilateral | Right | Unicompartmental | No | 8 | III |
| 112 | SH | FAS | 80 | Female | 60 | 1.65 | 22 | | Unilateral | Left | Bicompartmental | No | 12 | Ib |
| 115 | SH | FAS | 62 | Female | 71 | 1.66 | 26 | | Bilateral | Right | Unicompartmental | Yes | 17 | II |
| 117 | SH | FAS | 64 | Female | 85 | 1.69 | 30 | | Unilateral | Left | Unicompartmental | No | 3 | III |
| 125 | SH | PP | 60 | Female | 77 | 1.61 | 30 | | Bilateral | Left | Unicompartmental | No | 0 | II |
| 127 | SH | PP | 63 | Female | 58 | 1.67 | 21 | | Bilateral | Right | Unicompartmental | No | 3 | III |
| 133 | SH | PP | 56 | Female | 88 | 1.72 | 30 | | Unilateral | Right | Unicompartmental | No | 9 | II |
| 136 | SH | PP | 57 | Female | 63 | 1.58 | 25 | | Bilateral | Right | Bicompartmental | No | 7 | II |
| 139 | SH | PP | 75 | Female | 61 | 1.54 | 26 | | Bilateral | Right | Unicompartmental | No | 1 | II |
| 141 | SH | PP | 61 | Male | 70 | 1.65 | 26 | | Unilateral | Left | NA | No | 1 | Ib |
| 143 | SH | PP | 43 | Female | 40 | 1.60 | 16 | | Unilateral | Right | NA | No | 2 | Ib |
| 149 | SH | PP | 66 | Male | 91 | 1.76 | 29 | | Unilateral | Left | Unicompartmental | No | 11 | II |
| 151 | SH | PP | 75 | Female | 70 | 1.57 | 28 | | Bilateral | Left | Unicompartmental | Yes | 28 | II |
| 165 | SH | PP | 69 | Female | 69 | 1.53 | 29 | | Bilateral | Right | Unicompartmental | No | 1 | II |
| 170 | SH | PP | 80 | Female | 48 | 1.50 | 21 | | Bilateral | Left | Bicompartmental | No | 3 | II |
| 174 | SH | PP | 57 | Female | 47 | 1.51 | 21 | | Unilateral | Right | Unicompartmental | No | 7 | II |
| 175 | SH | PP | 78 | Male | 72 | 1.69 | 25 | | Unilateral | Left | Bicompartmental | No | 3 | III |
| 179 | SH | PP | 62 | Female | 61 | 1.65 | 22 | | Unilateral | Right | Unicompartmental | No | 1 | II |
| 180 | SH | PP | 71 | Female | 66 | 1.51 | 29 | | Unilateral | Right | Unicompartmental | No | 1 | II |
| 182 | SH | PP | 69 | Female | 56 | 1.59 | 22 | | Unilateral | Right | Bicompartmental | No | 7 | III |
| 183 | SH | PP | 63 | Female | 56 | 1.52 | 24 | | Unilateral | Left | Unicompartmental | No | 3 | II |
| 185 | SH | PP | 62 | Male | 88 | 1.81 | 27 | | Unilateral | Left | Unicompartmental | No | 9 | III |
| 188 | SH | PP | 60 | Female | 60 | 1.50 | 27 | | Bilateral | Right | Bicompartmental | No | 8 | III |
| 189 | SH | PP | 63 | Female | 61 | 1.68 | 22 | | Bilateral | Left | Unicompartmental | No | 2 | II |
| 191 | SH | PP | 70 | Male | 97 | 1.82 | 29 | | Unilateral | Left | Unicompartmental | No | 1 | Ib |
| 195 | SH | PP | 69 | Female | 73 | 1.58 | 29 | | Bilateral | Left | Unicompartmental | No | 1 | Ib |
| 196 | SH | PP | 60 | Female | 73 | 1.59 | 29 | | Bilateral | Left | Unicompartmental | No | 0 | Ib |
| 198 | SH | PP | 52 | Female | 85 | 1.69 | 30 | | Bilateral | Left | Bicompartmental | Yes | 6 | III |
| 200 | SH | FAS | 71 | Female | 73 | 1.54 | 31 | | Bilateral | Right | Bicompartmental | Yes | 6 | II |
| 202 | SH | FAS | 66 | Male | 88 | 1.71 | 30 | | Unilateral | Left | Unicompartmental | No | 3 | II |
| 204 | SH | PP | 71 | Female | 58 | 1.58 | 23 | | Unilateral | Right | Bicompartmental | No | 6 | II |
| 209 | SH | PP | 79 | Male | 83 | 1.66 | 30 | | Bilateral | Right | Unicompartmental | No | 3 | II |
| 210 | SH | PP | 74 | Female | 62 | 1.50 | 28 | | Bilateral | Right | Unicompartmental | No | 5 | II |
| 217 | SH | PP | 63 | Female | 54 | 1.56 | 22 | | Bilateral | Left | Unicompartmental | No | 10 | II |
| 218 | SH | PP | 67 | Male | 75 | 1.70 | 26 | | Unilateral | Right | Unicompartmental | No | 3 | II |
| 226 | SH | FAS | 54 | Female | 72 | 1.67 | 26 | | Unilateral | Left | Unicompartmental | No | 7 | II |
| 228 | SH | FAS | 72 | Female | 78 | 1.64 | 29 | | Unilateral | Left | Unicompartmental | No | 2 | II |
| 233 | SH | PP | 56 | Female | 71 | 1.58 | 28 | | Bilateral | Right | Unicompartmental | No | 2 | III |
| 239 | SH | PP | 67 | Female | 55 | 1.66 | 20 | | Unilateral | Right | Bicompartmental | No | 7 | II |
| 240 | SH | PP | 53 | Female | 78 | 1.65 | 29 | | Unilateral | Right | Unicompartmental | No | 2 | II |
| 245 | SH | PP | 72 | Male | 80 | 1.76 | 26 | | Unilateral | Right | Unicompartmental | No | 3 | II |
| 248 | SH | PP | 62 | Female | 70 | 1.60 | 27 | | Unilateral | Left | Unicompartmental | No | 0 | II |
| 249 | SH | FAS | 60 | Female | 79 | 1.70 | 27 | | Bilateral | Right | Bicompartmental | No | 7 | III |
| 250 | SH | PP | 60 | Female | 84 | 1.70 | 29 | | Unilateral | Right | Bicompartmental | Yes | 22 | II |
| 257 | SH | PP | 58 | Female | 70 | 1.62 | 27 | | Unilateral | Right | Bicompartmental | No | 2 | Ib |
| 259 | SH | PP | 51 | Male | 75 | 1.75 | 24 | | Unilateral | Left | Bicompartmental | Yes | 2 | III |
| 273 | SH | PP | 65 | Male | 83 | 1.70 | 29 | | Bilateral | Right | Unicompartmental | No | 11 | II |
| 275 | SH | PP | 81 | Female | 71 | 1.55 | 30 | | Unilateral | Left | Unicompartmental | No | 4 | III |
| 279 | SH | FAS | 41 | Female | 57 | 1.57 | 23 | | Bilateral | Right | Unicompartmental | Yes | 4 | II |
| 280 | SH | PP | 80 | Male | 89 | 1.78 | 28 | | Bilateral | Left | Unicompartmental | No | 1 | II |
| 281 | SH | PP | 67 | Male | 80 | 1.70 | 28 | | Bilateral | Right | Unicompartmental | No | 1 | Ib |
| 282 | SH | FAS | 79 | Female | 64 | 1.56 | 26 | | Unilateral | Right | Unicompartmental | No | 12 | II |
| 290 | SH | FAS | 66 | Male | 73 | 1.75 | 24 | | Bilateral | Left | Unicompartmental | No | 4 | II |
| 292 | SH | PP | 60 | Female | 66 | 1.58 | 26 | | Bilateral | Right | Unicompartmental | Yes | 6 | II |
| 298 | SH | PP | 77 | Female | 68 | 1.60 | 27 | | Bilateral | Left | Unicompartmental | No | 5 | II |
| 299 | SH | PP | 82 | Female | 65 | 1.60 | 25 | | Bilateral | Right | Unicompartmental | No | 2 | II |
| 302 | SH | PP | 69 | Female | 58 | 1.58 | 23 | | Bilateral | Right | Bicompartmental | No | 1 | II |
| 303 | SH | PP | 81 | Female | 67 | 1.53 | 29 | | Bilateral | Right | Unicompartmental | No | 0 | II |
| 305 | SH | PP | 73 | Female | 58 | 1.50 | 26 | | Bilateral | Right | Unicompartmental | No | 10 | II |
| 307 | SH | PP | 68 | Female | 78 | 1.65 | 29 | | Bilateral | Right | Unicompartmental | No | 4 | II |
| 309 | SH | PP | 81 | Female | 69 | 1.54 | 29 | | Bilateral | Left | Unicompartmental | No | 0 | II |
| 311 | SH | FAS | 53 | Female | 70 | 1.65 | 26 | | Bilateral | Left | Unicompartmental | No | 2 | Ib |
| 314 | SH | PP | 77 | Male | 70 | 1.60 | 27 | | Unilateral | Left | Unicompartmental | Yes | 6 | II |
| 315 | SH | PP | 62 | Female | 42 | 1.52 | 18 | | Unilateral | Left | Unicompartmental | No | 7 | III |
| 318 | SH | PP | 73 | Female | 70 | 1.59 | 28 | | Bilateral | Left | Unicompartmental | No | 3 | III |
| 319 | SH | PP | 76 | Male | 76 | 1.69 | 27 | | Bilateral | Right | Unicompartmental | No | 5 | II |
| 321 | SH | PP | 56 | Male | 83 | 1.82 | 25 | | Bilateral | Left | Bicompartmental | No | 0 | II |
| 322 | SH | PP | 82 | Female | 68 | 1.57 | 28 | | Bilateral | Right | Unicompartmental | Yes | 8 | III |
| 327 | SH | PP | 39 | Male | 84 | 1.75 | 27 | | Unilateral | Right | Unicompartmental | No | 3 | II |
| 328 | SH | PP | 59 | Male | 97 | 1.88 | 27 | | Unilateral | Right | Bicompartmental | No | 4 | II |
| 329 | SH | PP | 75 | Female | 68 | 1.70 | 24 | | Unilateral | Left | Bicompartmental | No | 11 | Ib |
| 330 | SH | PP | 59 | Male | 94 | 1.79 | 29 | | Unilateral | Right | Unicompartmental | Yes | 22 | III |
| 334 | SH | PP | 84 | Male | 88 | 1.75 | 29 | | Bilateral | Right | Bicompartmental | No | 5 | III |
| 335 | SH | PP | 61 | Female | 92 | 1.70 | 32 | | Bilateral | Right | Bicompartmental | No | 7 | II |
| 338 | SH | PP | 68 | Male | 83 | 1.74 | 27 | | Unilateral | Left | Bicompartmental | Yes | 4 | Ib |
| 340 | SH | PP | 58 | Female | 73 | 1.68 | 26 | | Unilateral | Left | Bicompartmental | Yes | 4 | II |
| 341 | SH | FAS | 68 | Female | 66 | 1.53 | 28 | | Bilateral | Right | Unicompartmental | No | 30 | II |
| 342 | SH | FAS | 81 | Female | 64 | 1.60 | 25 | | Bilateral | Left | Bicompartmental | Yes | 11 | III |
| 347 | SH | FAS | 64 | Female | 66 | 1.62 | 25 | | Unilateral | Left | Bicompartmental | Yes | 11 | Ib |
| 348 | SH | PP | 81 | Female | 60 | 1.53 | 26 | | Unilateral | Right | Bicompartmental | Yes | 6 | Ib |
| 349 | SH | PP | 63 | Male | 88 | 1.73 | 29 | | Bilateral | Left | Unicompartmental | No | 10 | III |
| 351 | SH | PP | 75 | Female | 72 | 1.59 | 28 | | Unilateral | Right | Unicompartmental | No | 16 | III |
| 358 | SH | FAS | 62 | Female | 75 | 1.65 | 28 | | Unilateral | Right | Unicompartmental | No | 4 | II |
| 360 | SH | PP | 72 | Female | 72 | 1.62 | 27 | | Bilateral | Left | Unicompartmental | No | 10 | III |
| 362 | SH | FAS | 60 | Female | 61 | 1.55 | 25 | | Unilateral | Left | Unicompartmental | No | 12 | II |
| 366 | SH | ITT | 70 | Male | 85 | 1.78 | 27 | | Unilateral | Right | Bicompartmental | No | 12 | II |
| 369 | SH | PP | 57 | Female | 82 | 1.65 | 30 | | Unilateral | Right | Unicompartmental | Yes | 12 | Ib |
| 373 | SH | PP | 46 | Male | 80 | 1.75 | 26 | | Bilateral | Left | Unicompartmental | Yes | 7 | III |
| 375 | SH | PP | 53 | Female | 58 | 1.57 | 24 | | Unilateral | Left | Unicompartmental | No | 10 | III |
| 382 | SH | FAS | 65 | Female | 78 | 1.68 | 28 | | Bilateral | Right | Bicompartmental | Yes | 5 | II |
| 383 | SH | PP | 70 | Female | 66 | 1.56 | 27 | | Bilateral | Right | Bicompartmental | Yes | 12 | III |
| 385 | SH | PP | 63 | Female | 53 | 1.55 | 22 | | Bilateral | Right | Bicompartmental | No | 10 | II |
| 387 | SH | PP | 78 | Female | 66 | 1.56 | 27 | | Bilateral | Left | Unicompartmental | No | 6 | II |
| 391 | SH | ITT | 73 | Female | 73 | 1.60 | 29 | | Unilateral | Right | Unicompartmental | Yes | 4 | III |
| 392 | SH | PP | 64 | Female | 71 | 1.65 | 26 | | Unilateral | Right | Bicompartmental | Yes | 6 | II |
| 393 | SH | PP | 74 | Female | 63 | 1.60 | 25 | | Bilateral | Right | Unicompartmental | No | 8 | III |
| 396 | SH | PP | 61 | Female | 54 | 1.50 | 24 | | Unilateral | Right | Unicompartmental | No | 10 | III |
| 397 | SH | PP | 63 | Female | 52 | 1.64 | 19 | | Unilateral | Left | Unicompartmental | No | 11 | III |
| 400 | SH | PP | 79 | Female | 71 | 1.57 | 29 | | Unilateral | Right | Unicompartmental | No | 11 | II |
| 001 | Control | PP | 80 | Female | 78 | 1.66 | 28 | | Unilateral | Right | Unicompartmental | Yes | 2 | II |
| 004 | Control | PP | 60 | Female | 58 | 1.60 | 23 | | Bilateral | Right | Unicompartmental | No | 8 | II |
| 005 | Control | PP | 61 | Male | 80 | 1.68 | 28 | | Bilateral | Right | Unicompartmental | No | 6 | II |
| 007 | Control | PP | 77 | Female | 80 | 1.60 | 31 | | Bilateral | Right | Unicompartmental | No | 11 | III |
| 010 | Control | PP | 79 | Male | 96 | 1.90 | 27 | | Unilateral | Left | Unicompartmental | No | 11 | II |
| 011 | Control | PP | 48 | Male | 82 | 1.67 | 29 | | Unilateral | Left | Unicompartmental | No | 10 | Ib |
| 013 | Control | PP | 42 | Male | 73 | 1.72 | 25 | | Unilateral | Left | Unicompartmental | No | 10 | II |
| 016 | Control | PP | 81 | Female | 60 | 1.57 | 24 | | Bilateral | Right | Unicompartmental | No | 7 | II |
| 017 | Control | PP | 69 | Male | 73 | 1.67 | 26 | | Unilateral | Right | Unicompartmental | No | 0 | II |
| 018 | Control | FAS | 54 | Male | 68 | 1.68 | 24 | | Unilateral | Right | Bicompartmental | No | 4 | II |
| 021 | Control | PP | 79 | Female | 63 | 1.54 | 27 | | Bilateral | Left | Bicompartmental | No | 30 | II |
| 024 | Control | PP | 84 | Female | 65 | 1.54 | 27 | | Unilateral | Left | Bicompartmental | Yes | 26 | III |
| 027 | Control | PP | 79 | Female | 62 | 1.65 | 23 | | Unilateral | Right | Bicompartmental | No | 10 | II |
| 028 | Control | PP | 74 | Male | 85 | 1.72 | 29 | | Unilateral | Right | Bicompartmental | No | 9 | III |
| 030 | Control | PP | 57 | Male | 80 | 1.72 | 27 | | Unilateral | Left | Bicompartmental | No | 4 | II |
| 033 | Control | PP | 60 | Female | 58 | 1.60 | 23 | | Unilateral | Right | Unicompartmental | Yes | 10 | Ib |
| 038 | Control | PP | 42 | Female | 70 | 1.67 | 25 | | Unilateral | Left | Unicompartmental | No | 0 | II |
| 039 | Control | PP | 64 | Male | 84 | 1.78 | 27 | | Unilateral | Left | Unicompartmental | No | 1 | II |
| 042 | Control | PP | 68 | Female | 48 | 1.59 | 19 | | Unilateral | Left | Unicompartmental | No | 0 | II |
| 043 | Control | PP | 55 | Male | 78 | 1.80 | 24 | | Unilateral | Right | Bicompartmental | No | 0 | II |
| 045 | Control | PP | 79 | Male | 83 | 1.80 | 26 | | Bilateral | Left | Unicompartmental | No | 2 | III |
| 046 | Control | PP | 75 | Female | 70 | 1.63 | 26 | | Bilateral | Right | Unicompartmental | No | 6 | Ib |
| 049 | Control | PP | 65 | Female | 66 | 1.59 | 26 | | Bilateral | Left | Bicompartmental | No | 5 | III |
| 051 | Control | PP | 83 | Female | 63 | 1.64 | 23 | | Bilateral | Right | Unicompartmental | Yes | 5 | Ib |
| 054 | Control | PP | 74 | Male | 78 | 1.70 | 27 | | Unilateral | Left | Unicompartmental | No | 3 | II |
| 056 | Control | PP | 72 | Female | 74 | 1.58 | 30 | | Bilateral | Left | Bicompartmental | Yes | 7 | III |
| 059 | Control | PP | 80 | Female | 83 | 1.65 | 30 | | Bilateral | Right | Bicompartmental | No | 4 | II |
| 060 | Control | PP | 56 | Male | 85 | 1.70 | 29 | | Bilateral | Right | Unicompartmental | No | 3 | II |
| 061 | Control | PP | 72 | Female | 61 | 1.58 | 24 | | Bilateral | Left | Unicompartmental | Yes | 7 | III |
| 062 | Control | PP | 70 | Male | 89 | 1.76 | 29 | | Bilateral | Right | Unicompartmental | No | 1 | II |
| 066 | Control | PP | 71 | Male | 87 | 1.80 | 27 | | Bilateral | Right | Bicompartmental | No | 12 | Ib |
| 067 | Control | PP | 58 | Male | 84 | 1.76 | 27 | | Bilateral | Left | Unicompartmental | Yes | 3 | Ib |
| 069 | Control | PP | 85 | Female | 61 | 1.52 | 26 | | Bilateral | Right | Unicompartmental | Yes | 10 | II |
| 071 | Control | PP | 70 | Female | 47 | 1.48 | 21 | | Unilateral | Right | Bicompartmental | No | 5 | Ib |
| 073 | Control | FAS | 65 | Female | 77 | 1.64 | 29 | | Unilateral | Left | Bicompartmental | Yes | 5 | Ib |
| 074 | Control | PP | 54 | Female | 40 | 1.50 | 18 | | Unilateral | Left | Bicompartmental | Yes | 9 | Ib |
| 082 | Control | PP | 69 | Female | 85 | 1.70 | 29 | | Unilateral | Left | Unicompartmental | No | 1 | II |
| 085 | Control | FAS | 68 | Female | 71 | 1.58 | 28 | | Unilateral | Right | Bicompartmental | Yes | 9 | II |
| 090 | Control | PP | 65 | Female | 72 | 1.67 | 26 | | Bilateral | Left | Unicompartmental | No | 11 | III |
| 091 | Control | FAS | 51 | Male | 80 | 1.73 | 27 | | Bilateral | Left | Unicompartmental | No | 11 | III |
| 093 | Control | FAS | 44 | Female | 65 | 1.63 | 24 | | Unilateral | Left | Unicompartmental | No | 9 | II |
| 096 | Control | PP | 56 | Male | 78 | 1.90 | 22 | | Unilateral | Left | Unicompartmental | No | 5 | II |
| 098 | Control | PP | 55 | Male | 70 | 1.68 | 25 | | Unilateral | Right | Bicompartmental | No | 10 | II |
| 103 | Control | PP | 60 | Male | 66 | 1.79 | 21 | | Bilateral | Right | Unicompartmental | Yes | 3 | II |
| 104 | Control | PP | 52 | Male | 72 | 1.78 | 23 | | Unilateral | Right | Unicompartmental | Yes | 1 | II |
| 105 | Control | PP | 70 | Male | 86 | 1.69 | 30 | | Unilateral | Right | Unicompartmental | Yes | 2 | Ib |
| 108 | Control | FAS | 53 | Female | 79 | 1.70 | 27 | | Bilateral | Right | Unicompartmental | Yes | 3 | III |
| 109 | Control | FAS | 80 | Female | 65 | 1.59 | 26 | | Unilateral | Left | Bicompartmental | No | 6 | II |
| 111 | Control | ITT | 86 | Female | 60 | 1.57 | 24 | | Unilateral | Right | Unicompartmental | No | 0 | II |
| 113 | Control | PP | 79 | Female | 70 | 1.60 | 27 | | Bilateral | Left | Bicompartmental | No | 7 | III |
| 114 | Control | FAS | 73 | Female | 77 | 1.58 | 31 | | Bilateral | Left | Bicompartmental | No | 10 | II |
| 121 | Control | PP | 72 | Female | 60 | 1.57 | 24 | | Bilateral | Left | Unicompartmental | Yes | 26 | II |
| 126 | Control | PP | 49 | Female | 74 | 1.72 | 25 | | Bilateral | Left | Unicompartmental | No | 10 | III |
| 128 | Control | FAS | 47 | Female | 83 | 1.64 | 31 | | Bilateral | Left | Unicompartmental | No | 3 | II |
| 134 | Control | PP | 53 | Female | 70 | 1.55 | 29 | | Unilateral | Right | Bicompartmental | No | 7 | II |
| 135 | Control | PP | 75 | Male | 88 | 1.74 | 29 | | Bilateral | Left | Unicompartmental | Yes | 8 | II |
| 142 | Control | ITT | 80 | Female | 80 | 1.60 | 31 | | Unilateral | Right | NA | No | 1 | III |
| 150 | Control | PP | 77 | Male | 94 | 1.80 | 29 | | Bilateral | Left | Unicompartmental | Yes | 16 | III |
| 152 | Control | PP | 52 | Female | 74 | 1.59 | 29 | | Bilateral | Left | Unicompartmental | No | 2 | Ib |
| 153 | Control | FAS | 59 | Male | 80 | 1.77 | 26 | | Bilateral | Right | Unicompartmental | No | 10 | II |
| 166 | Control | PP | 61 | Female | 76 | 1.62 | 29 | | Bilateral | Right | Unicompartmental | No | 2 | II |
| 169 | Control | PP | 65 | Male | 79 | 1.78 | 25 | | Bilateral | Left | Unicompartmental | No | 14 | III |
| 171 | Control | PP | 61 | Female | 73 | 1.58 | 29 | | Bilateral | Right | Unicompartmental | No | 6 | III |
| 173 | Control | FAS | 71 | Female | 63 | 1.58 | 25 | | Unilateral | Right | Unicompartmental | No | 6 | II |
| 176 | Control | FAS | 64 | Female | 74 | 1.63 | 28 | | Unilateral | Left | Unicompartmental | No | 2 | II |
| 177 | Control | PP | 64 | Male | 97 | 1.84 | 29 | | Unilateral | Right | Unicompartmental | No | 6 | II |
| 178 | Control | FAS | 77 | Female | 75 | 1.63 | 28 | | Bilateral | Left | Unicompartmental | No | 10 | III |
| 181 | Control | PP | 45 | Female | 63 | 1.63 | 24 | | Unilateral | Left | Unicompartmental | Yes | 10 | II |
| 184 | Control | PP | 42 | Female | 58 | 1.66 | 21 | | Unilateral | Right | Bicompartmental | Yes | 9 | II |
| 186 | Control | PP | 58 | Male | 90 | 1.83 | 27 | | Bilateral | Left | Bicompartmental | No | 11 | II |
| 187 | Control | FAS | 65 | Male | 89 | 1.77 | 28 | | Bilateral | Left | Bicompartmental | No | 7 | III |
| 190 | Control | FAS | 63 | Female | 71 | 1.58 | 28 | | Bilateral | Left | Unicompartmental | Yes | 17 | II |
| 192 | Control | PP | 84 | Female | 59 | 1.60 | 23 | | Unilateral | Left | Bicompartmental | No | 4 | II |
| 193 | Control | PP | 77 | Female | 73 | 1.70 | 25 | | Bilateral | Left | Unicompartmental | No | 13 | II |
| 194 | Control | PP | 63 | Female | 67 | 1.56 | 28 | | Bilateral | Left | Unicompartmental | No | 0 | Ib |
| 197 | Control | PP | 65 | Male | 84 | 1.70 | 29 | | Unilateral | Right | Bicompartmental | Yes | 4 | II |
| 199 | Control | PP | 62 | Female | 76 | 1.55 | 32 | | Bilateral | Left | Bicompartmental | Yes | 6 | III |
| 201 | Control | PP | 61 | Female | 75 | 1.60 | 29 | | Unilateral | Right | Bicompartmental | No | 3 | II |
| 203 | Control | PP | 67 | Female | 76 | 1.68 | 27 | | Bilateral | Left | Bicompartmental | No | 3 | II |
| 211 | Control | PP | 76 | Female | 75 | 1.72 | 25 | | Bilateral | Right | Unicompartmental | No | 12 | Ib |
| 212 | Control | PP | 83 | Female | 69 | 1.54 | 29 | | Bilateral | Left | Unicompartmental | No | 1 | Ib |
| 213 | Control | PP | 55 | Male | 101 | 1.78 | 32 | | Unilateral | Right | Bicompartmental | No | 1 | Ib |
| 221 | Control | ITT | 56 | Female | 90 | 1.87 | 26 | | Unilateral | Right | Bicompartmental | No | 2 | II |
| 225 | Control | PP | 50 | Female | 105 | 1.96 | 27 | | Unilateral | Right | Unicompartmental | No | 0 | II |
| 227 | Control | FAS | 84 | Female | 69 | 1.60 | 27 | | Unilateral | Left | Unicompartmental | No | 5 | III |
| 229 | Control | FAS | 72 | Female | 72 | 1.58 | 29 | | Bilateral | Right | Unicompartmental | Yes | 2 | III |
| 230 | Control | FAS | 74 | Female | 66 | 1.66 | 24 | | Unilateral | Right | Unicompartmental | No | 4 | III |
| 237 | Control | FAS | 51 | Female | 50 | 1.48 | 23 | | Unilateral | Left | Bicompartmental | Yes | 2 | II |
| 238 | Control | PP | 53 | Female | 79 | 1.65 | 29 | | Bilateral | Left | Unicompartmental | No | 1 | II |
| 246 | Control | PP | 74 | Female | 70 | 1.58 | 28 | | Bilateral | Right | Unicompartmental | No | 3 | II |
| 247 | Control | PP | 69 | Male | 80 | 1.75 | 26 | | Bilateral | Right | Unicompartmental | No | 4 | Ib |
| 251 | Control | PP | 73 | Male | 83 | 1.70 | 29 | | Bilateral | Left | Bicompartmental | No | 22 | II |
| 252 | Control | PP | 57 | Female | 75 | 1.61 | 29 | | Bilateral | Right | Bicompartmental | Yes | 17 | II |
| 258 | Control | PP | 75 | Female | 59 | 1.58 | 24 | | Unilateral | Right | Unicompartmental | Yes | 0 | III |
| 260 | Control | PP | 53 | Female | 57 | 1.63 | 21 | | Unilateral | Right | Bicompartmental | Yes | 6 | II |
| 261 | Control | ITT | 69 | Female | 72 | 1.68 | 26 | | Unilateral | Left | Unicompartmental | Yes | 3 | II |
| 269 | Control | ITT | 79 | Female | 60 | 1.57 | 24 | | Bilateral | Right | Bicompartmental | No | 5 | Ib |
| 274 | Control | PP | 64 | Male | 75 | 1.62 | 29 | | Unilateral | Right | Unicompartmental | No | 7 | II |
| 276 | Control | PP | 66 | Female | 50 | 1.65 | 18 | | Unilateral | Right | Unicompartmental | No | 9 | III |
| 277 | Control | FAS | 76 | Female | 49 | 1.60 | 19 | | Unilateral | Left | Unicompartmental | No | 14 | III |
| 278 | Control | PP | 53 | Female | 71 | 1.56 | 29 | | Unilateral | Left | Bicompartmental | No | 16 | II |
| 283 | Control | PP | 72 | Female | 63 | 1.55 | 26 | | Unilateral | Left | Unicompartmental | No | 6 | Ib |
| 284 | Control | PP | 82 | Female | 78 | 1.70 | 27 | | Bilateral | Left | Unicompartmental | No | 32 | II |
| 289 | Control | PP | 83 | Male | 80 | 1.78 | 25 | | Bilateral | Right | Unicompartmental | No | 12 | II |
| 291 | Control | PP | 60 | Male | 80 | 1.75 | 26 | | Bilateral | Right | Unicompartmental | No | 20 | II |
| 297 | Control | PP | 71 | Male | 69 | 1.68 | 24 | | Bilateral | Left | Unicompartmental | No | 2 | Ib |
| 300 | Control | PP | 70 | Male | 74 | 1.73 | 25 | | Bilateral | Right | Unicompartmental | No | 4 | II |
| 301 | Control | PP | 59 | Male | 82 | 1.76 | 26 | | Bilateral | Right | Unicompartmental | No | 0 | II |
| 304 | Control | PP | 64 | Female | 51 | 1.59 | 20 | | Bilateral | Left | Unicompartmental | No | 7 | II |
| 306 | Control | PP | 56 | Female | 80 | 1.60 | 31 | | Bilateral | Left | Unicompartmental | No | 5 | II |
| 308 | Control | PP | 50 | Male | 72 | 1.72 | 24 | | Bilateral | Right | Unicompartmental | No | 0 | Ib |
| 310 | Control | FAS | 72 | Male | 96 | 1.82 | 29 | | Unilateral | Right | Bicompartmental | No | 9 | III |
| 312 | Control | FAS | 68 | Female | 75 | 1.60 | 29 | | Bilateral | Right | Unicompartmental | No | 1 | Ib |
| 313 | Control | PP | 73 | Female | 50 | 1.60 | 20 | | Unilateral | Right | Unicompartmental | Yes | 4 | II |
| 317 | Control | PP | 65 | Female | 81 | 1.65 | 30 | | Unilateral | Right | Unicompartmental | No | 1 | III |
| 320 | Control | FAS | 78 | Male | 66 | 1.74 | 22 | | Unilateral | Right | Unicompartmental | No | 10 | III |
| 323 | Control | FAS | 78 | Female | 61 | 1.65 | 22 | | Bilateral | Right | Unicompartmental | No | 7 | II |
| 324 | Control | FAS | 55 | Female | 61 | 1.65 | 22 | | Unilateral | Left | Bicompartmental | Yes | 8 | II |
| 325 | Control | PP | 81 | Male | 72 | 1.77 | 23 | | Bilateral | Right | Bicompartmental | Yes | 9 | III |
| 326 | Control | PP | 70 | Male | 82 | 1.76 | 26 | | Bilateral | Left | Bicompartmental | No | 8 | II |
| 331 | Control | PP | 66 | Male | 85 | 1.86 | 25 | | Unilateral | Right | Unicompartmental | No | 8 | III |
| 333 | Control | PP | 70 | Male | 77 | 1.72 | 26 | | Unilateral | Left | Unicompartmental | No | 1 | II |
| 336 | Control | PP | 72 | Male | 72 | 1.63 | 27 | | Bilateral | Right | Unicompartmental | No | 6 | II |
| 337 | Control | PP | 68 | Male | 97 | 1.91 | 27 | | Unilateral | Right | Unicompartmental | No | 6 | II |
| 339 | Control | PP | 66 | Male | 75 | 1.65 | 28 | | Unilateral | Left | Bicompartmental | Yes | 12 | II |
| 343 | Control | PP | 83 | Female | 56 | 1.60 | 22 | | Bilateral | Right | Unicompartmental | No | 6 | II |
| 344 | Control | FAS | 75 | Female | 61 | 1.62 | 23 | | Unilateral | Left | Bicompartmental | Yes | 32 | Ib |
| 345 | Control | PP | 84 | Female | 70 | 1.63 | 26 | | Unilateral | Right | Bicompartmental | No | 10 | Ib |
| 346 | Control | PP | 68 | Male | 82 | 1.73 | 27 | | Unilateral | Right | Bicompartmental | No | 0 | Ib |
| 350 | Control | ITT | 78 | Male | 88 | 1.77 | 28 | | Bilateral | Left | Bicompartmental | No | 16 | III |
| 352 | Control | PP | 54 | Male | 98 | 1.83 | 29 | | Unilateral | Left | Unicompartmental | No | 9 | II |
| 357 | Control | PP | 71 | Female | 60 | 1.50 | 27 | | Unilateral | Left | Unicompartmental | No | 3 | II |
| 359 | Control | PP | 70 | Female | 60 | 1.50 | 27 | | Bilateral | Left | Unicompartmental | No | 1 | II |
| 361 | Control | PP | 74 | Female | 60 | 1.50 | 27 | | Bilateral | Left | Unicompartmental | No | 3 | II |
| 365 | Control | ITT | 75 | Female | 77 | 1.52 | 33 | | Unilateral | Left | Bicompartmental | Yes | 1 | Ib |
| 370 | Control | PP | 71 | Female | 74 | 1.74 | 24 | | Bilateral | Left | Bicompartmental | No | 2 | Ib |
| 371 | Control | PP | 67 | Male | 79 | 1.69 | 28 | | Bilateral | Left | Unicompartmental | Yes | 10 | II |
| 374 | Control | PP | 67 | Male | 74 | 1.73 | 25 | | Unilateral | Left | Unicompartmental | Yes | 1 | III |
| 381 | Control | PP | 56 | Female | 72 | 1.72 | 24 | | Bilateral | Right | Unicompartmental | No | 1 | II |
| 384 | Control | PP | 68 | Male | 85 | 1.75 | 28 | | Bilateral | Right | Unicompartmental | Yes | 3 | II |
| 386 | Control | PP | 65 | Male | 88 | 1.75 | 29 | | Bilateral | Right | Bicompartmental | Yes | 1 | II |
| 388 | Control | PP | 74 | Female | 70 | 1.65 | 26 | | Bilateral | Left | Bicompartmental | Yes | 11 | III |
| 389 | Control | FAS | 51 | Female | 72 | 1.58 | 29 | | Unilateral | Left | Bicompartmental | No | 3 | II |
| 390 | Control | FAS | 65 | Male | 76 | 1.63 | 29 | | Bilateral | Right | Bicompartmental | Yes | 10 | III |
| 394 | Control | PP | 68 | Female | 78 | 1.64 | 29 | | Unilateral | Left | Unicompartmental | No | 15 | II |
| 395 | Control | PP | 61 | Female | 90 | 1.76 | 29 | | Bilateral | Right | Unicompartmental | No | 10 | II |
| 398 | Control | FAS | 50 | Male | 85 | 1.80 | 26 | | Unilateral | Left | Unicompartmental | No | 4 | II |
| 399 | Control | PP | 74 | Female | 66 | 1.60 | 26 | | Unilateral | Right | Unicompartmental | No | 7 | III |

BMI = body mass index; control = hylan G-F 20; FAS= Full Analysis Set; ITT = Intention-to-Treat; NA = not available; PP = Per Protocol; SH = sodium hyaluronate.
