# Supplementary material for: A single intra-articular injection of 2.0% non-chemically modified sodium hyaluronate vs 0.8% hylan G-F 20 in the treatment of symptomatic knee osteoarthritis: A 6-month, multicenter, randomized, controlled non-inferiority trial
Source: PLoS One. 2019 Dec 10;14(12):e0226007. doi: 10.1371/journal.pone.0226007 (PMC6903764; doi:10.1371/journal.pone.0226007)
Supplement: S6 Table — (DOCX) [file pone.0226007.s011.docx]

**S6 Table. Primary and secondary efficacy criteria at 6 months post injection (Full Analysis Set).**

| **Outcome** | **SH** n = 139 | **Control** n = 141 | | **Difference** | | ***P*** |  |
| --- | --- | --- | --- | --- | --- | --- | --- |
| WOMAC A change from baseline (mm) |  | |  | |  |  | |
| Mean (SE) | -33.3 (1.7) | | -36.1 (1.9) | | -2.8 (1.9) | 0.3† | |
| 95% CI | (-36.7, -29.8) | | (-39.8, -32.4) | | (-7.9, 2.2) |  | |
| WOMAC B change from baseline (mm) |  | |  | |  |  | |
| Mean (SE) | -21.9 (2.1) | | -26.9 (1.9) | | -5.0 (2.9) | 0.08† | |
| 95% CI | (-26.1, -17.7) | | (-30.7, -23.1) | | (-10.7, 0.6) |  | |
| WOMAC C change from baseline (mm) |  | |  | |  |  | |
| Mean (SE) | -21.3 (1.6) | | -26.1 (1.8) | | -4.9 (2.4) | 0.04† | |
| 95% CI | (-24.4, -18.1) | | (-29.6, -22.6) | | (-9.5, 0.1) |  | |
| Lequesne index change from baseline |  | |  | |  |  | |
| Mean (SE) | -4.2 (0.3) | | -4.7 (0.4) | | -0.5 (0.5) | 0.3† | |
| 95% CI | (-4.8, -3.6) | | (-5.4, -4.0) | | (-1.4, 0.4) |  | |
| PtGA change from baseline (mm) |  | |  | |  |  | |
| Mean (SE) | -24.0 (2.1) | | -27.3 (2.0) | | -3.3 (2.9) | 0.3† | |
| 95% CI | (-28.1, -19.9) | | (-31.3, -23.4) | | (-9.0, 2.3) |  | |
| Global treatment efficacy (good/very good), n (%) |  | |  | |  |  | |
| By the patient | 86 (64.7) | | 84 (64.1) | |  | >0.9‡ | |
| By the investigator | 90 (67.7) | | 87 (66.4) | |  | 0.9‡ | |
| Acetaminophen use post injection (yes), n (%) | 101 (72.7) | | 97 (68.8) | |  | 0.5‡ | |
| OMERACT-OARSI responders, n (%) | 107 (81.1) | | 113 (86.3) | |  | 0.3§ | |

† Student’s t-test; ‡ Fisher’s exact test; § Chi-square test.

CI = confidence interval; control = hylan G-F 20; OMERACT-OARSI = Outcome Measures in Rheumatoid Arthritis Clinical Trials - Osteoarthritis Research Society International; PtGA = patient global assessment of disease activity; SE = standard error; SH = sodium hyaluronate; WOMAC A, B, C = Western Ontario and McMaster Universities Osteoarthritis Index pain, stiffness, function subscales, respectively.
