# Supplementary material for: A single intra-articular injection of 2.0% non-chemically modified sodium hyaluronate vs 0.8% hylan G-F 20 in the treatment of symptomatic knee osteoarthritis: A 6-month, multicenter, randomized, controlled non-inferiority trial
Source: PLoS One. 2019 Dec 10;14(12):e0226007. doi: 10.1371/journal.pone.0226007 (PMC6903764; doi:10.1371/journal.pone.0226007)
Supplement: S7 Table — (DOCX) [file pone.0226007.s012.docx]

**S7 Table. Individual WOMAC A pain values (mm) (Intention-to-Treat population).**

| **Patient** | **Group** | **Dataset** | **C1 (D0)** | **C3 (D30)** | **C4 (D90)** | **C5 (D180)** |
| --- | --- | --- | --- | --- | --- | --- |
| 002 | SH | PP | 67 | 33 | 17 | 13 |
| 003 | SH | PP | 72 | 55 | 52 | 56 |
| 006 | SH | PP | 68 | 39 | 36 | 39 |
| 008 | SH | PP | 67 | 29 | 30 | 42 |
| 009 | SH | PP | 62 | 42 | 0 | 0 |
| 012 | SH | PP | 53 | 5 | 3 | 5 |
| 014 | SH | PP | 41 | 0 | 0 | 1 |
| 015 | SH | PP | 48 | 0 | 0 | 0 |
| 019 | SH | FAS | 67 | 22 | 22 | 19 |
| 020 | SH | PP | 62 | 51 | 42 | 32 |
| 022 | SH | PP | 52 | 46 | 47 | 47 |
| 023 | SH | PP | 61 | 42 | 40 | 32 |
| 025 | SH | FAS | 68 | 48 | NA | NA |
| 026 | SH | FAS | 61 | 28 | 40 | NA |
| 029 | SH | FAS | 60 | 46 | 62 | 62 |
| 034 | SH | ITT | 62 | NA | NA | NA |
| 037 | SH | PP | 51 | 30 | 11 | 9 |
| 040 | SH | PP | 46 | 28 | 13 | 7 |
| 041 | SH | PP | 50 | 25 | 8 | 12 |
| 044 | SH | PP | 54 | 22 | 8 | 6 |
| 048 | SH | PP | 59 | 29 | 8 | 5 |
| 050 | SH | PP | 41 | 22 | 2 | 6 |
| 052 | SH | PP | 71 | 21 | 35 | 75 |
| 053 | SH | PP | 63 | 46 | 33 | 55 |
| 055 | SH | PP | 67 | 22 | 18 | 44 |
| 057 | SH | PP | 78 | 19 | 8 | 27 |
| 058 | SH | PP | 84 | 26 | 8 | 40 |
| 063 | SH | PP | 85 | 26 | 13 | 16 |
| 064 | SH | FAS | 74 | 13 | 11 | 10 |
| 065 | SH | PP | 69 | 69 | 53 | 34 |
| 068 | SH | FAS | 62 | 58 | 7 | 37 |
| 070 | SH | PP | 58 | 60 | 52 | 61 |
| 072 | SH | PP | 68 | 35 | 21 | 16 |
| 075 | SH | FAS | 59 | 42 | 36 | 22 |
| 076 | SH | PP | 48 | 39 | 34 | 25 |
| 081 | SH | PP | 57 | 6 | 15 | 2 |
| 089 | SH | PP | 52 | 36 | 17 | 18 |
| 092 | SH | PP | 61 | 19 | 55 | 41 |
| 094 | SH | FAS | 65 | 25 | 43 | 61 |
| 095 | SH | ITT | 60 | NA | NA | NA |
| 097 | SH | ITT | 81 | NA | NA | NA |
| 101 | SH | PP | 70 | 48 | 38 | 57 |
| 102 | SH | PP | 54 | 5 | 13 | 48 |
| 106 | SH | PP | 48 | 8 | 8 | 9 |
| 107 | SH | PP | 55 | 5 | 6 | 9 |
| 110 | SH | PP | 37 | 18 | 46 | 50 |
| 112 | SH | FAS | 31 | 15 | NA | 10 |
| 115 | SH | FAS | 57 | NA | NA | 44 |
| 117 | SH | FAS | 57 | 25 | 71 | NA |
| 125 | SH | PP | 40 | 1 | 1 | 1 |
| 127 | SH | PP | 43 | 11 | 11 | 9 |
| 133 | SH | PP | 61 | 44 | 35 | 35 |
| 136 | SH | PP | 57 | 24 | 14 | 20 |
| 139 | SH | PP | 40 | 66 | 24 | 9 |
| 141 | SH | PP | 70 | 67 | NA | 44 |
| 143 | SH | PP | 59 | 56 | 44 | 53 |
| 149 | SH | PP | 43 | 18 | 19 | 21 |
| 151 | SH | PP | 61 | 44 | 20 | 48 |
| 165 | SH | PP | 59 | 3 | 44 | 66 |
| 170 | SH | PP | 45 | 25 | 24 | 13 |
| 174 | SH | PP | 64 | 1 | 1 | 1 |
| 175 | SH | PP | 43 | 43 | 39 | 20 |
| 179 | SH | PP | 61 | 37 | 71 | 61 |
| 180 | SH | PP | 67 | 12 | 48 | 57 |
| 182 | SH | PP | 63 | 4 | 8 | 1 |
| 183 | SH | PP | 59 | 51 | 10 | 24 |
| 185 | SH | PP | 44 | 30 | 15 | 10 |
| 188 | SH | PP | 62 | 24 | 18 | 27 |
| 189 | SH | PP | 67 | 40 | 26 | 10 |
| 191 | SH | PP | 65 | 15 | 16 | 14 |
| 195 | SH | PP | 66 | 28 | 27 | 4 |
| 196 | SH | PP | 63 | 27 | 9 | 2 |
| 198 | SH | PP | 79 | 42 | 69 | 25 |
| 200 | SH | FAS | 71 | 46 | 36 | 25 |
| 202 | SH | FAS | 68 | 63 | 56 | 57 |
| 204 | SH | PP | 69 | 12 | 2 | 41 |
| 209 | SH | PP | 75 | 11 | 2 | 7 |
| 210 | SH | PP | 75 | 17 | 11 | 20 |
| 217 | SH | PP | 49 | 10 | 17 | 17 |
| 218 | SH | PP | 45 | 5 | 7 | 12 |
| 226 | SH | FAS | 20 | 44 | 55 | 64 |
| 228 | SH | FAS | 34 | 41 | 39 | 19 |
| 233 | SH | PP | 55 | 20 | 1 | 1 |
| 239 | SH | PP | 47 | 15 | 9 | 0 |
| 240 | SH | PP | 53 | 0 | 0 | 0 |
| 245 | SH | PP | 63 | 29 | 25 | 11 |
| 248 | SH | PP | 52 | 49 | 44 | 37 |
| 249 | SH | FAS | 85 | 54 | 33 | 25 |
| 250 | SH | PP | 88 | 46 | 42 | 36 |
| 257 | SH | PP | 47 | 24 | 25 | 21 |
| 259 | SH | PP | 69 | 61 | 51 | 47 |
| 273 | SH | PP | 54 | 35 | 23 | 25 |
| 275 | SH | PP | 60 | 41 | 30 | 48 |
| 279 | SH | FAS | 63 | 68 | 38 | 21 |
| 280 | SH | PP | 57 | 14 | 31 | 28 |
| 281 | SH | PP | 46 | 7 | 17 | 2 |
| 282 | SH | FAS | 66 | 57 | 42 | 29 |
| 290 | SH | FAS | 64 | 35 | 45 | 42 |
| 292 | SH | PP | 59 | 26 | 21 | 13 |
| 298 | SH | PP | 71 | 22 | 7 | 5 |
| 299 | SH | PP | 75 | 20 | 4 | 8 |
| 302 | SH | PP | 53 | 10 | 5 | 4 |
| 303 | SH | PP | 59 | 24 | 24 | 22 |
| 305 | SH | PP | 68 | 36 | 19 | 45 |
| 307 | SH | PP | 55 | 46 | 38 | 31 |
| 309 | SH | PP | 49 | 21 | 12 | 12 |
| 311 | SH | FAS | 67 | 20 | NA | NA |
| 314 | SH | PP | 56 | 2 | 3 | 7 |
| 315 | SH | PP | 51 | 10 | 6 | 3 |
| 318 | SH | PP | 60 | 3 | 2 | 7 |
| 319 | SH | PP | 59 | 37 | 17 | 6 |
| 321 | SH | PP | 54 | 24 | 17 | 3 |
| 322 | SH | PP | 59 | 43 | 16 | 44 |
| 327 | SH | PP | 55 | 52 | 56 | 49 |
| 328 | SH | PP | 78 | 12 | 8 | 35 |
| 329 | SH | PP | 56 | 0 | 3 | 29 |
| 330 | SH | PP | 59 | 44 | 57 | 51 |
| 334 | SH | PP | 64 | 37 | 30 | 21 |
| 335 | SH | PP | 66 | 40 | 47 | 42 |
| 338 | SH | PP | 67 | 40 | 28 | 25 |
| 340 | SH | PP | 59 | 40 | 32 | 44 |
| 341 | SH | FAS | 63 | 57 | 18 | 44 |
| 342 | SH | FAS | 59 | 10 | 13 | 27 |
| 347 | SH | FAS | 41 | 9 | 3 | 10 |
| 348 | SH | PP | 43 | 19 | 21 | 18 |
| 349 | SH | PP | 57 | 16 | 15 | 10 |
| 351 | SH | PP | 51 | 20 | 14 | 40 |
| 358 | SH | FAS | 63 | 46 | 17 | NA |
| 360 | SH | PP | 61 | 5 | 2 | 7 |
| 362 | SH | FAS | 59 | 51 | NA | NA |
| 366 | SH | ITT | NA | 23 | 7 | 9 |
| 369 | SH | PP | 41 | 28 | 13 | 1 |
| 373 | SH | PP | 52 | 41 | 9 | 10 |
| 375 | SH | PP | 41 | 31 | 20 | 3 |
| 382 | SH | FAS | 66 | 26 | 1 | 2 |
| 383 | SH | PP | 65 | 49 | 65 | 14 |
| 385 | SH | PP | 50 | 8 | 41 | 49 |
| 387 | SH | PP | 55 | 30 | 46 | 49 |
| 391 | SH | ITT | 53 | NA | NA | NA |
| 392 | SH | PP | 50 | 5 | 11 | 18 |
| 393 | SH | PP | 41 | 2 | 7 | 4 |
| 396 | SH | PP | 40 | 2 | 50 | 58 |
| 397 | SH | PP | 40 | 18 | 6 | 6 |
| 400 | SH | PP | 56 | 5 | 4 | 8 |
| 001 | Control | PP | 67 | 37 | 21 | 19 |
| 004 | Control | PP | 61 | 35 | 21 | 23 |
| 005 | Control | PP | 57 | 52 | 52 | 42 |
| 007 | Control | PP | 67 | 35 | 27 | 52 |
| 010 | Control | PP | 52 | 9 | 5 | 11 |
| 011 | Control | PP | 68 | 24 | 18 | 16 |
| 013 | Control | PP | 40 | 0 | 0 | 0 |
| 016 | Control | PP | 54 | 5 | 7 | 29 |
| 017 | Control | PP | 53 | 38 | 45 | 35 |
| 018 | Control | FAS | 71 | 80 | 84 | NA |
| 021 | Control | PP | 62 | 55 | 86 | 70 |
| 024 | Control | PP | 48 | 16 | 25 | 47 |
| 027 | Control | PP | 50 | 38 | 19 | 24 |
| 028 | Control | PP | 61 | 31 | NA | 31 |
| 030 | Control | PP | 58 | 56 | 48 | 56 |
| 033 | Control | PP | 54 | 54 | 59 | 56 |
| 038 | Control | PP | 48 | 33 | 9 | 9 |
| 039 | Control | PP | 46 | 31 | 13 | 9 |
| 042 | Control | PP | 52 | 27 | 9 | 7 |
| 043 | Control | PP | 50 | 28 | 8 | 10 |
| 045 | Control | PP | 39 | 24 | 25 | 3 |
| 046 | Control | PP | 45 | 27 | 8 | 1 |
| 049 | Control | PP | 51 | 27 | 34 | 13 |
| 051 | Control | PP | 58 | 24 | 17 | 24 |
| 054 | Control | PP | 50 | 36 | 35 | 37 |
| 056 | Control | PP | 78 | 66 | 44 | 41 |
| 059 | Control | PP | 79 | 7 | 12 | 12 |
| 060 | Control | PP | 71 | 6 | 3 | 7 |
| 061 | Control | PP | 69 | 10 | 6 | 29 |
| 062 | Control | PP | 71 | 2 | 3 | 3 |
| 066 | Control | PP | 53 | 43 | 21 | 6 |
| 067 | Control | PP | 59 | 10 | 7 | 4 |
| 069 | Control | PP | 66 | 66 | 55 | 60 |
| 071 | Control | PP | 59 | 50 | 51 | 51 |
| 073 | Control | FAS | 41 | 12 | 10 | 8 |
| 074 | Control | PP | 59 | 24 | 15 | 10 |
| 082 | Control | PP | 47 | 23 | 9 | 9 |
| 085 | Control | FAS | 67 | 26 | 6 | 3 |
| 090 | Control | PP | 60 | 60 | 75 | 39 |
| 091 | Control | FAS | 44 | 38 | 2 | 0 |
| 093 | Control | FAS | 43 | 5 | 55 | 18 |
| 096 | Control | PP | 61 | 63 | 18 | 17 |
| 098 | Control | PP | 77 | 22 | 9 | 7 |
| 103 | Control | PP | 56 | 13 | 8 | 11 |
| 104 | Control | PP | 68 | 4 | 11 | 0 |
| 105 | Control | PP | 54 | 10 | 3 | 13 |
| 108 | Control | FAS | 65 | 60 | NA | NA |
| 109 | Control | FAS | 25 | 11 | NA | 13 |
| 111 | Control | ITT | 29 | NA | NA | NA |
| 113 | Control | PP | 56 | 41 | 25 | 6 |
| 114 | Control | FAS | 64 | 18 | 2 | 9 |
| 121 | Control | PP | 40 | 48 | 33 | 49 |
| 126 | Control | PP | 80 | 59 | 52 | 48 |
| 128 | Control | FAS | 89 | 9 | 0 | 0 |
| 134 | Control | PP | 75 | 9 | 39 | 32 |
| 135 | Control | PP | 72 | 61 | 22 | 16 |
| 142 | Control | ITT | 80 | NA | NA | NA |
| 150 | Control | PP | 65 | 13 | 13 | 76 |
| 152 | Control | PP | 73 | 28 | 16 | 10 |
| 153 | Control | FAS | 62 | 14 | 12 | 38 |
| 166 | Control | PP | 48 | 6 | 17 | 17 |
| 169 | Control | PP | 63 | 45 | 77 | 59 |
| 171 | Control | PP | 65 | 74 | 90 | 83 |
| 173 | Control | FAS | 69 | 23 | 19 | NA |
| 176 | Control | FAS | 47 | 74 | 72 | 5 |
| 177 | Control | PP | 45 | 26 | 16 | 19 |
| 178 | Control | FAS | 40 | 7 | 17 | 17 |
| 181 | Control | PP | 66 | 27 | 25 | 21 |
| 184 | Control | PP | 47 | 39 | 31 | 8 |
| 186 | Control | PP | 62 | 16 | 27 | 23 |
| 187 | Control | FAS | 61 | 9 | NA | NA |
| 190 | Control | FAS | 69 | 30 | 24 | 31 |
| 192 | Control | PP | 68 | 48 | 33 | 33 |
| 193 | Control | PP | 77 | 47 | 24 | 3 |
| 194 | Control | PP | 63 | 15 | 23 | 6 |
| 197 | Control | PP | 67 | 22 | 24 | 42 |
| 199 | Control | PP | 76 | 32 | 25 | 34 |
| 201 | Control | PP | 65 | 56 | 56 | 75 |
| 203 | Control | PP | 63 | 35 | 47 | 36 |
| 211 | Control | PP | 76 | 12 | 19 | 2 |
| 212 | Control | PP | 72 | 6 | 19 | 8 |
| 213 | Control | PP | 45 | 27 | 0 | 2 |
| 221 | Control | ITT | 72 | NA | NA | NA |
| 225 | Control | PP | 61 | 18 | 55 | 55 |
| 227 | Control | FAS | 35 | 64 | NA | NA |
| 229 | Control | FAS | 52 | 29 | 42 | 35 |
| 230 | Control | FAS | 53 | 44 | NA | NA |
| 237 | Control | FAS | 53 | 59 | NA | NA |
| 238 | Control | PP | 67 | 17 | 0 | 3 |
| 246 | Control | PP | 75 | 73 | 19 | 13 |
| 247 | Control | PP | 51 | 38 | 26 | 31 |
| 251 | Control | PP | 75 | 32 | 17 | 18 |
| 252 | Control | PP | 65 | 27 | 20 | 23 |
| 258 | Control | PP | 58 | 22 | 16 | 10 |
| 260 | Control | PP | 45 | 21 | 20 | 20 |
| 261 | Control | ITT | 76 | NA | NA | NA |
| 269 | Control | ITT | 66 | NA | NA | NA |
| 274 | Control | PP | 63 | 57 | 7 | 2 |
| 276 | Control | PP | 48 | 17 | 28 | 22 |
| 277 | Control | FAS | 60 | 27 | 21 | 27 |
| 278 | Control | PP | 67 | 31 | 40 | 26 |
| 283 | Control | PP | 44 | 48 | 10 | 10 |
| 284 | Control | PP | 47 | 20 | 28 | 16 |
| 289 | Control | PP | 47 | 27 | 26 | 36 |
| 291 | Control | PP | 55 | 2 | 13 | 10 |
| 297 | Control | PP | 64 | 15 | 2 | 3 |
| 300 | Control | PP | 79 | 8 | 10 | 28 |
| 301 | Control | PP | 53 | 7 | 9 | 6 |
| 304 | Control | PP | 63 | 25 | 10 | 37 |
| 306 | Control | PP | 66 | 40 | 0 | 1 |
| 308 | Control | PP | 44 | 23 | 12 | 3 |
| 310 | Control | FAS | 37 | 20 | 16 | 16 |
| 312 | Control | FAS | 52 | 22 | 13 | NA |
| 313 | Control | PP | 58 | 18 | 60 | 42 |
| 317 | Control | PP | 58 | 8 | 26 | 11 |
| 320 | Control | FAS | 65 | 12 | 26 | 24 |
| 323 | Control | FAS | 54 | 44 | NA | NA |
| 324 | Control | FAS | 43 | 15 | 75 | NA |
| 325 | Control | PP | 70 | 16 | 8 | 7 |
| 326 | Control | PP | 53 | 6 | 7 | 7 |
| 331 | Control | PP | 42 | 0 | 0 | 1 |
| 333 | Control | PP | 45 | 21 | 3 | 12 |
| 336 | Control | PP | 53 | 51 | 25 | 15 |
| 337 | Control | PP | 74 | 57 | 40 | 30 |
| 339 | Control | PP | 69 | 41 | 34 | 39 |
| 343 | Control | PP | 46 | 20 | 18 | 19 |
| 344 | Control | FAS | 65 | 25 | 29 | 21 |
| 345 | Control | PP | 52 | 24 | 16 | 14 |
| 346 | Control | PP | 52 | 31 | 34 | 29 |
| 350 | Control | ITT | 72 | NA | NA | NA |
| 352 | Control | PP | 62 | 80 | 97 | 92 |
| 357 | Control | PP | 40 | 13 | 9 | 5 |
| 359 | Control | PP | 59 | 12 | 0 | 0 |
| 361 | Control | PP | 66 | 23 | 21 | 13 |
| 365 | Control | ITT | NA | NA | 31 | 11 |
| 370 | Control | PP | 43 | 5 | 7 | 31 |
| 371 | Control | PP | 82 | 22 | 8 | 7 |
| 374 | Control | PP | 40 | 23 | 72 | 68 |
| 381 | Control | PP | 65 | 5 | 4 | 4 |
| 384 | Control | PP | 61 | 24 | 8 | 15 |
| 386 | Control | PP | 45 | 21 | 30 | 13 |
| 388 | Control | PP | 52 | 1 | 18 | 10 |
| 389 | Control | FAS | 61 | 16 | 50 | 57 |
| 390 | Control | FAS | 60 | 16 | 3 | 26 |
| 394 | Control | PP | 55 | 2 | 0 | 4 |
| 395 | Control | PP | 55 | 0 | 0 | 6 |
| 398 | Control | FAS | 40 | 4 | 2 | 4 |
| 399 | Control | PP | 42 | 3 | 24 | 16 |

C = Consultation; control = hylan G-F 20; D = Day; FAS = Full Analysis Set; ITT = Intention-to-Treat; NA = not available; SH = sodium hyaluronate; PP = Per Protocol; WOMAC A = Western Ontario and McMaster Universities Osteoarthritis Index pain subscale.
