# Supplementary material for: A single intra-articular injection of 2.0% non-chemically modified sodium hyaluronate vs 0.8% hylan G-F 20 in the treatment of symptomatic knee osteoarthritis: A 6-month, multicenter, randomized, controlled non-inferiority trial
Source: PLoS One. 2019 Dec 10;14(12):e0226007. doi: 10.1371/journal.pone.0226007 (PMC6903764; doi:10.1371/journal.pone.0226007)
Supplement: S9 Table — (DOCX) [file pone.0226007.s014.docx]

**S9 Table. Individual WOMAC C function values (mm) (Intention-to-Treat population).**

| **Patient** | **Group** | **Dataset** | **C1 (D0)** | **C3 (D30)** | **C4 (D90)** | **C5 (D180)** |
| --- | --- | --- | --- | --- | --- | --- |
| 002 | SH | PP | 62 | 27 | 20 | 18 |
| 003 | SH | PP | 61 | 58 | 54 | 48 |
| 006 | SH | PP | 65 | 31 | 28 | 35 |
| 008 | SH | PP | 63 | 31 | 28 | 41 |
| 009 | SH | PP | 59 | 29 | 22 | 25 |
| 012 | SH | PP | 59 | 6 | 0 | 2 |
| 014 | SH | PP | 14 | 1 | 1 | 0 |
| 015 | SH | PP | 37 | 1 | 0 | 0 |
| 019 | SH | FAS | 56 | 12 | 5 | 7 |
| 020 | SH | PP | 43 | 40 | 26 | 46 |
| 022 | SH | PP | 50 | 39 | 51 | 49 |
| 023 | SH | PP | 54 | 45 | 52 | 43 |
| 025 | SH | FAS | 53 | 44 | NA | NA |
| 026 | SH | FAS | 55 | 27 | 38 | NA |
| 029 | SH | FAS | 61 | 44 | 60 | 60 |
| 034 | SH | ITT | 55 | NA | NA | NA |
| 037 | SH | PP | 42 | 35 | 14 | 19 |
| 040 | SH | PP | 49 | 33 | 17 | 10 |
| 041 | SH | PP | 52 | 31 | 13 | 10 |
| 044 | SH | PP | 49 | 27 | 11 | 9 |
| 048 | SH | PP | 31 | 29 | 7 | 4 |
| 050 | SH | PP | 25 | 9 | 2 | 2 |
| 052 | SH | PP | 64 | 17 | 55 | 55 |
| 053 | SH | PP | 57 | 48 | 39 | 50 |
| 055 | SH | PP | 49 | 20 | 20 | 45 |
| 057 | SH | PP | 32 | 12 | 7 | 22 |
| 058 | SH | PP | 42 | 15 | 9 | 34 |
| 063 | SH | PP | 58 | 27 | 18 | 15 |
| 064 | SH | FAS | 42 | 13 | 7 | 9 |
| 065 | SH | PP | 69 | 66 | 58 | 42 |
| 068 | SH | FAS | 69 | 49 | 17 | 37 |
| 070 | SH | PP | 50 | 52 | 55 | 61 |
| 072 | SH | PP | 53 | 33 | 23 | 18 |
| 075 | SH | FAS | 57 | 40 | 27 | 24 |
| 076 | SH | PP | 26 | 39 | 34 | 23 |
| 081 | SH | PP | 19 | 2 | 3 | 1 |
| 089 | SH | PP | 21 | 23 | 14 | 15 |
| 092 | SH | PP | 64 | 41 | 53 | 51 |
| 094 | SH | FAS | 57 | 38 | 57 | 69 |
| 095 | SH | ITT | 57 | NA | NA | NA |
| 097 | SH | ITT | 72 | NA | NA | NA |
| 101 | SH | PP | 59 | 42 | 14 | 46 |
| 102 | SH | PP | 71 | 13 | 46 | 74 |
| 106 | SH | PP | 29 | 11 | 10 | 12 |
| 107 | SH | PP | 51 | 6 | 7 | 7 |
| 110 | SH | PP | NA | 20 | 27 | 37 |
| 112 | SH | FAS | 33 | 16 | NA | 12 |
| 115 | SH | FAS | 41 | NA | NA | 61 |
| 117 | SH | FAS | 62 | 49 | 73 | NA |
| 125 | SH | PP | 8 | 1 | 1 | 0 |
| 127 | SH | PP | 26 | 8 | 8 | 21 |
| 133 | SH | PP | 35 | 35 | 35 | 34 |
| 136 | SH | PP | 49 | 27 | 18 | 24 |
| 139 | SH | PP | 36 | 29 | 17 | 12 |
| 141 | SH | PP | 69 | 77 | NA | 52 |
| 143 | SH | PP | 66 | 44 | 39 | 51 |
| 149 | SH | PP | 27 | 16 | 17 | 19 |
| 151 | SH | PP | 59 | 44 | 60 | 61 |
| 165 | SH | PP | 44 | 5 | 34 | 51 |
| 170 | SH | PP | 17 | 19 | 10 | 10 |
| 174 | SH | PP | 42 | 2 | 2 | 2 |
| 175 | SH | PP | 49 | 48 | 47 | 38 |
| 179 | SH | PP | 47 | 48 | 69 | 46 |
| 180 | SH | PP | 50 | 12 | 39 | 42 |
| 182 | SH | PP | 47 | 11 | 11 | 1 |
| 183 | SH | PP | 48 | 24 | 9 | 24 |
| 185 | SH | PP | 35 | 19 | 13 | 12 |
| 188 | SH | PP | 53 | 24 | 44 | 40 |
| 189 | SH | PP | 66 | 37 | 22 | 13 |
| 191 | SH | PP | 70 | 24 | 26 | 24 |
| 195 | SH | PP | 48 | 17 | 16 | 8 |
| 196 | SH | PP | 47 | 15 | 13 | 6 |
| 198 | SH | PP | 49 | 37 | 65 | 29 |
| 200 | SH | FAS | 55 | 36 | 32 | 35 |
| 202 | SH | FAS | 57 | 52 | 54 | 44 |
| 204 | SH | PP | 52 | 22 | 2 | 35 |
| 209 | SH | PP | 39 | 7 | 2 | 13 |
| 210 | SH | PP | 59 | 16 | 10 | 14 |
| 217 | SH | PP | 42 | 11 | 18 | 17 |
| 218 | SH | PP | 26 | 9 | 8 | 14 |
| 226 | SH | FAS | 17 | 44 | 54 | 54 |
| 228 | SH | FAS | 41 | 36 | 42 | 37 |
| 233 | SH | PP | 48 | 13 | 1 | 4 |
| 239 | SH | PP | 38 | 18 | 6 | 2 |
| 240 | SH | PP | 42 | 0 | 0 | 0 |
| 245 | SH | PP | 46 | 11 | 19 | 7 |
| 248 | SH | PP | 29 | 37 | 29 | 26 |
| 249 | SH | FAS | 85 | 59 | 39 | 35 |
| 250 | SH | PP | 84 | 51 | 44 | 39 |
| 257 | SH | PP | 33 | 28 | 23 | 20 |
| 259 | SH | PP | 56 | 50 | 52 | 48 |
| 273 | SH | PP | 47 | 35 | 12 | 37 |
| 275 | SH | PP | 39 | 40 | 36 | 28 |
| 279 | SH | FAS | 72 | 67 | 31 | 17 |
| 280 | SH | PP | 54 | 36 | 70 | 67 |
| 281 | SH | PP | 39 | 9 | 16 | 4 |
| 282 | SH | FAS | 68 | 54 | 42 | 29 |
| 290 | SH | FAS | 64 | 49 | 52 | 41 |
| 292 | SH | PP | 48 | 35 | 28 | 23 |
| 298 | SH | PP | 50 | 13 | 6 | 2 |
| 299 | SH | PP | 43 | 11 | 4 | 3 |
| 302 | SH | PP | 40 | 9 | 1 | 2 |
| 303 | SH | PP | 51 | 16 | 21 | 15 |
| 305 | SH | PP | 79 | 41 | 23 | 48 |
| 307 | SH | PP | 69 | 52 | 46 | 37 |
| 309 | SH | PP | 21 | 15 | 11 | 18 |
| 311 | SH | FAS | 59 | 20 | NA | NA |
| 314 | SH | PP | 37 | 5 | 6 | 8 |
| 315 | SH | PP | 25 | 9 | 7 | 6 |
| 318 | SH | PP | 49 | 8 | 3 | 4 |
| 319 | SH | PP | 56 | 21 | 17 | 4 |
| 321 | SH | PP | 24 | 20 | 6 | 3 |
| 322 | SH | PP | 47 | 37 | 22 | 40 |
| 327 | SH | PP | 50 | 45 | 45 | 35 |
| 328 | SH | PP | 49 | 5 | 4 | 24 |
| 329 | SH | PP | 15 | 0 | 0 | 8 |
| 330 | SH | PP | 33 | 24 | 14 | 36 |
| 334 | SH | PP | 62 | 42 | 36 | 35 |
| 335 | SH | PP | 65 | 59 | 59 | 58 |
| 338 | SH | PP | 50 | 37 | 34 | 24 |
| 340 | SH | PP | 53 | 36 | 27 | 49 |
| 341 | SH | FAS | 52 | 47 | 14 | 41 |
| 342 | SH | FAS | 64 | 13 | 19 | 39 |
| 347 | SH | FAS | 26 | 2 | 2 | 7 |
| 348 | SH | PP | 38 | 18 | 12 | 12 |
| 349 | SH | PP | 4 | 6 | 1 | 0 |
| 351 | SH | PP | 40 | 41 | 12 | 39 |
| 358 | SH | FAS | 52 | 43 | 11 | NA |
| 360 | SH | PP | 43 | 6 | 6 | 2 |
| 362 | SH | FAS | 69 | 48 | NA | NA |
| 366 | SH | ITT | NA | 22 | 7 | 11 |
| 369 | SH | PP | 48 | 31 | 11 | 1 |
| 373 | SH | PP | 43 | 36 | 7 | 11 |
| 375 | SH | PP | 23 | 17 | 7 | 4 |
| 382 | SH | FAS | 70 | 32 | 1 | 2 |
| 383 | SH | PP | 35 | 31 | 33 | 8 |
| 385 | SH | PP | 49 | 13 | 58 | 62 |
| 387 | SH | PP | 58 | 39 | 51 | 43 |
| 391 | SH | ITT | 45 | NA | NA | NA |
| 392 | SH | PP | 39 | 8 | 17 | 9 |
| 393 | SH | PP | 16 | 2 | 4 | 2 |
| 396 | SH | PP | 14 | 9 | 19 | 27 |
| 397 | SH | PP | 13 | 9 | 3 | 2 |
| 400 | SH | PP | 50 | 14 | 10 | 8 |
| 001 | Control | PP | 66 | 38 | 26 | 28 |
| 004 | Control | PP | 69 | 37 | 30 | 26 |
| 005 | Control | PP | 58 | 46 | 37 | 52 |
| 007 | Control | PP | 61 | 33 | 31 | 47 |
| 010 | Control | PP | 50 | 10 | 4 | 13 |
| 011 | Control | PP | 65 | 43 | 33 | 25 |
| 013 | Control | PP | 3 | 0 | 0 | 1 |
| 016 | Control | PP | 46 | 7 | 10 | 23 |
| 017 | Control | PP | 36 | 22 | 33 | 24 |
| 018 | Control | FAS | 39 | 54 | 63 | NA |
| 021 | Control | PP | 54 | 57 | 86 | 76 |
| 024 | Control | PP | 46 | 28 | 28 | 34 |
| 027 | Control | PP | 49 | 38 | 20 | 32 |
| 028 | Control | PP | 57 | 46 | NA | 42 |
| 030 | Control | PP | 61 | 55 | 53 | 53 |
| 033 | Control | PP | 55 | 55 | 52 | 54 |
| 038 | Control | PP | 45 | 39 | 12 | 10 |
| 039 | Control | PP | 44 | 34 | 16 | 11 |
| 042 | Control | PP | 47 | 31 | 11 | 7 |
| 043 | Control | PP | 44 | 29 | 12 | 11 |
| 045 | Control | PP | 27 | 14 | 21 | 2 |
| 046 | Control | PP | 37 | 23 | 5 | 5 |
| 049 | Control | PP | 20 | 20 | 19 | 5 |
| 051 | Control | PP | 43 | 31 | 16 | 18 |
| 054 | Control | PP | 55 | 36 | 37 | 31 |
| 056 | Control | PP | 60 | 49 | 42 | 63 |
| 059 | Control | PP | 43 | 8 | 13 | 18 |
| 060 | Control | PP | 29 | 4 | 3 | 4 |
| 061 | Control | PP | 41 | 13 | 5 | 12 |
| 062 | Control | PP | 43 | 14 | 7 | 3 |
| 066 | Control | PP | 39 | 34 | 24 | 11 |
| 067 | Control | PP | 53 | 10 | 9 | 5 |
| 069 | Control | PP | 65 | 65 | 59 | 63 |
| 071 | Control | PP | 57 | 56 | 51 | 58 |
| 073 | Control | FAS | 46 | 16 | 11 | 8 |
| 074 | Control | PP | 49 | 23 | 10 | 5 |
| 082 | Control | PP | 31 | 17 | 10 | 7 |
| 085 | Control | FAS | 53 | 11 | 5 | 3 |
| 090 | Control | PP | 50 | 59 | 50 | 39 |
| 091 | Control | FAS | 25 | 22 | 2 | 1 |
| 093 | Control | FAS | 28 | 6 | 59 | 21 |
| 096 | Control | PP | 41 | 64 | 18 | 18 |
| 098 | Control | PP | 63 | 31 | 10 | 8 |
| 103 | Control | PP | 65 | 10 | 17 | 7 |
| 104 | Control | PP | 30 | 1 | 0 | 1 |
| 105 | Control | PP | 53 | 9 | 2 | 10 |
| 108 | Control | FAS | 54 | 52 | NA | NA |
| 109 | Control | FAS | 30 | 20 | NA | 19 |
| 111 | Control | ITT | 35 | NA | NA | NA |
| 113 | Control | PP | 35 | 15 | 4 | 7 |
| 114 | Control | FAS | 37 | 11 | 2 | 14 |
| 121 | Control | PP | 28 | 58 | 51 | 26 |
| 126 | Control | PP | 69 | 61 | 60 | 55 |
| 128 | Control | FAS | 76 | 1 | 0 | 0 |
| 134 | Control | PP | 81 | 14 | 32 | 33 |
| 135 | Control | PP | 60 | 56 | 21 | 18 |
| 142 | Control | ITT | 76 | NA | NA | NA |
| 150 | Control | PP | 48 | 22 | 14 | 64 |
| 152 | Control | PP | 62 | 40 | 12 | 9 |
| 153 | Control | FAS | 82 | 18 | 16 | 46 |
| 166 | Control | PP | 35 | 6 | 19 | 14 |
| 169 | Control | PP | 66 | 38 | 57 | 47 |
| 171 | Control | PP | 56 | 65 | 83 | 83 |
| 173 | Control | FAS | 59 | 37 | 30 | NA |
| 176 | Control | FAS | 60 | 74 | 77 | 9 |
| 177 | Control | PP | 18 | 18 | 4 | 18 |
| 178 | Control | FAS | 36 | 7 | 15 | 20 |
| 181 | Control | PP | 63 | 40 | 19 | 20 |
| 184 | Control | PP | 33 | 20 | 13 | 6 |
| 186 | Control | PP | 47 | 16 | 24 | 23 |
| 187 | Control | FAS | 37 | 8 | NA | NA |
| 190 | Control | FAS | 64 | 28 | 23 | 28 |
| 192 | Control | PP | 64 | 57 | 51 | 44 |
| 193 | Control | PP | 60 | 15 | 16 | 6 |
| 194 | Control | PP | 52 | 16 | 7 | 3 |
| 197 | Control | PP | 56 | 24 | 28 | 39 |
| 199 | Control | PP | 55 | 27 | 29 | 40 |
| 201 | Control | PP | 47 | 48 | 48 | 72 |
| 203 | Control | PP | 54 | 39 | 45 | 36 |
| 211 | Control | PP | 62 | 4 | 20 | 6 |
| 212 | Control | PP | 48 | 5 | 10 | 11 |
| 213 | Control | PP | 40 | 25 | 1 | 2 |
| 221 | Control | ITT | 70 | NA | NA | NA |
| 225 | Control | PP | 37 | 18 | 58 | 58 |
| 227 | Control | FAS | 8 | 49 | NA | NA |
| 229 | Control | FAS | 47 | 39 | 47 | 59 |
| 230 | Control | FAS | 31 | 28 | NA | NA |
| 237 | Control | FAS | 61 | 52 | NA | NA |
| 238 | Control | PP | 62 | 4 | 1 | 1 |
| 246 | Control | PP | 66 | 52 | 11 | 10 |
| 247 | Control | PP | 39 | 31 | 23 | 35 |
| 251 | Control | PP | 70 | 27 | 15 | 16 |
| 252 | Control | PP | 58 | 21 | 9 | 8 |
| 258 | Control | PP | 40 | 33 | 32 | 27 |
| 260 | Control | PP | 45 | 32 | 15 | 14 |
| 261 | Control | ITT | 81 | NA | NA | NA |
| 269 | Control | ITT | 69 | NA | NA | NA |
| 274 | Control | PP | 64 | 24 | 5 | 2 |
| 276 | Control | PP | 32 | 14 | 19 | 19 |
| 277 | Control | FAS | 66 | 27 | 29 | 31 |
| 278 | Control | PP | 63 | 30 | 36 | 29 |
| 283 | Control | PP | 58 | 51 | 20 | 19 |
| 284 | Control | PP | 55 | 34 | 30 | 22 |
| 289 | Control | PP | 43 | 23 | 32 | 27 |
| 291 | Control | PP | 50 | 1 | 12 | 6 |
| 297 | Control | PP | 42 | 14 | 2 | 2 |
| 300 | Control | PP | 35 | 9 | 9 | 17 |
| 301 | Control | PP | 36 | 8 | 5 | 4 |
| 304 | Control | PP | 24 | 24 | 10 | 22 |
| 306 | Control | PP | 74 | 18 | 0 | 2 |
| 308 | Control | PP | 21 | 16 | 10 | 2 |
| 310 | Control | FAS | 43 | 22 | 20 | 19 |
| 312 | Control | FAS | 53 | 10 | 21 | NA |
| 313 | Control | PP | 41 | 40 | 81 | 35 |
| 317 | Control | PP | 45 | 10 | 16 | 11 |
| 320 | Control | FAS | 43 | 20 | 37 | 23 |
| 323 | Control | FAS | 40 | 43 | NA | NA |
| 324 | Control | FAS | 18 | 10 | 48 | NA |
| 325 | Control | PP | 66 | 21 | 11 | 11 |
| 326 | Control | PP | 61 | 23 | 13 | 10 |
| 331 | Control | PP | 15 | 0 | 0 | 0 |
| 333 | Control | PP | 41 | 23 | 4 | 5 |
| 336 | Control | PP | 50 | 37 | 28 | 16 |
| 337 | Control | PP | 67 | 55 | 39 | 29 |
| 339 | Control | PP | 64 | 48 | 40 | 56 |
| 343 | Control | PP | 28 | 25 | 21 | 15 |
| 344 | Control | FAS | 74 | 40 | 29 | 23 |
| 345 | Control | PP | 49 | 31 | 11 | 10 |
| 346 | Control | PP | 40 | 26 | 22 | 22 |
| 350 | Control | ITT | 85 | NA | NA | NA |
| 352 | Control | PP | 48 | 60 | 93 | 91 |
| 357 | Control | PP | 49 | 20 | 14 | 10 |
| 359 | Control | PP | 39 | 7 | 2 | 0 |
| 361 | Control | PP | 41 | 30 | 24 | 18 |
| 365 | Control | ITT | NA | NA | 28 | 9 |
| 370 | Control | PP | 23 | 1 | 5 | 29 |
| 371 | Control | PP | 58 | 25 | 8 | 11 |
| 374 | Control | PP | 53 | 26 | 45 | 41 |
| 381 | Control | PP | 43 | 6 | 3 | 7 |
| 384 | Control | PP | 52 | 16 | 10 | 16 |
| 386 | Control | PP | 29 | 17 | 26 | 13 |
| 388 | Control | PP | 42 | 0 | 26 | 14 |
| 389 | Control | FAS | 65 | 13 | 39 | 39 |
| 390 | Control | FAS | 45 | 4 | 9 | 33 |
| 394 | Control | PP | 30 | 2 | 0 | 4 |
| 395 | Control | PP | 42 | 1 | 1 | 4 |
| 398 | Control | FAS | 25 | 3 | 1 | 4 |
| 399 | Control | PP | 31 | 2 | 32 | 10 |

C = Consultation; control = hylan G-F 20; D = Day; FAS = Full Analysis Set; ITT = Intention-to-Treat; NA = not available; SH = sodium hyaluronate; PP = Per Protocol; WOMAC C = Western Ontario and McMaster Universities Osteoarthritis Index function subscale.
