# Supplementary material for: A single intra-articular injection of 2.0% non-chemically modified sodium hyaluronate vs 0.8% hylan G-F 20 in the treatment of symptomatic knee osteoarthritis: A 6-month, multicenter, randomized, controlled non-inferiority trial
Source: PLoS One. 2019 Dec 10;14(12):e0226007. doi: 10.1371/journal.pone.0226007 (PMC6903764; doi:10.1371/journal.pone.0226007)
Supplement: S13 Table — (DOCX) [file pone.0226007.s018.docx]

**S13 Table. Individual acetaminophen use post injection (Intention-to-Treat population).**

| **Patient** | **Group** | **Dataset** | **C3 (D30)** | | | |  | **C4 (D90)** | | | |  | **C5 (D180)** | | | |
| --- | --- | --- | --- | --- | --- | --- | --- | --- | --- | --- | --- | --- | --- | --- | --- | --- |
|  |  |  | **Use** | **No units** | **Unit dose (mg)** | **Total dose (mg)** |  | **Use** | **No units** | **Unit dose (mg)** | **Total dose (mg)** |  | **Use** | **No units** | **Unit dose (mg)** | **Total dose (mg)** |
| 002 | SH | PP | Oui | 24 | 1000 | 24000 |  | Oui | 8 | 1000 | 8000 |  | Non | 0 | NA | NA |
| 003 | SH | PP | Oui | 32 | 1000 | 32000 |  | Oui | 48 | 1000 | 48000 |  | Oui | 64 | 1000 | 64000 |
| 006 | SH | PP | Non | 0 | NA | NA |  | Non | 0 | NA | NA |  | Oui | 80 | 1000 | 80000 |
| 008 | SH | PP | Oui | 42 | 500 | 21000 |  | Oui | 120 | 500 | 60000 |  | Oui | 200 | 1000 | 200000 |
| 009 | SH | PP | Oui | 2 | 1000 | 2000 |  | Oui | 40 | 1000 | 40000 |  | Oui | 71 | 1000 | 71000 |
| 012 | SH | PP | Non | 0 | NA | NA |  | Non | 0 | NA | NA |  | Non | 0 | NA | NA |
| 014 | SH | PP | Non | 0 | NA | NA |  | Non | 0 | NA | NA |  | Non | 0 | NA | NA |
| 015 | SH | PP | Non | 0 | NA | NA |  | Non | 0 | NA | NA |  | Non | 0 | NA | NA |
| 019 | SH | FAS | Non | 0 | NA | NA |  | Non | 0 | NA | NA |  | Non | 0 | NA | NA |
| 020 | SH | PP | Oui | 52 | 1000 | 52000 |  | Oui | 67 | 1000 | 67000 |  | Oui | 45 | 1000 | 45000 |
| 022 | SH | PP | Oui | 28 | 500 | 14000 |  | Oui | 7 | 1000 | 7000 |  | Oui | 15 | 500 | 7500 |
| 023 | SH | PP | Oui | 28 | 500 | 14000 |  | Oui | 20 | 1000 | 20000 |  | Oui | 10 | 1000 | 10000 |
| 025 | SH | FAS | Oui | 3 | 500 | 1500 |  | NA | NA | NA | NA |  | NA | NA | NA | NA |
| 026 | SH | FAS | Non | 0 | NA | NA |  | Oui | 4 | 1000 | 4000 |  | NA | NA | NA | NA |
| 029 | SH | FAS | Non | 0 | NA | NA |  | Non | 0 | NA | NA |  | Non | 0 | NA | NA |
| 034 | SH | ITT | NA | NA | NA | NA |  | NA | NA | NA | NA |  | NA | NA | NA | NA |
| 037 | SH | PP | Oui | 17 | 1000 | 17000 |  | Oui | 19 | 1000 | 19000 |  | Oui | 31 | 1000 | 31000 |
| 040 | SH | PP | Oui | 15 | 1000 | 15000 |  | Oui | 21 | 1000 | 21000 |  | Oui | 30 | 1000 | 30000 |
| 041 | SH | PP | Oui | 15 | 1000 | 15000 |  | Oui | 22 | 1000 | 22000 |  | Oui | 40 | 1000 | 40000 |
| 044 | SH | PP | Oui | 12 | 1000 | 12000 |  | Oui | 25 | 1000 | 25000 |  | Oui | 30 | 1000 | 30000 |
| 048 | SH | PP | Non | 0 | NA | NA |  | Oui | 72 | 500 | 36000 |  | Non | 0 | NA | NA |
| 050 | SH | PP | Oui | 8 | 500 | 4000 |  | Oui | 14 | 500 | 7000 |  | Oui | 24 | 500 | 12000 |
| 052 | SH | PP | Oui | 53 | 1000 | 53000 |  | Oui | 93 | 1000 | 93000 |  | Oui | 140 | 1000 | 140000 |
| 053 | SH | PP | Oui | 80 | 1000 | 80000 |  | Oui | 120 | 1000 | 120000 |  | Oui | 320 | 1000 | 320000 |
| 055 | SH | PP | Non | 0 | NA | NA |  | Non | 0 | NA | NA |  | Non | 0 | NA | NA |
| 057 | SH | PP | Oui | 22 | 1000 | 22000 |  | Non | 0 | NA | NA |  | Non | 0 | NA | NA |
| 058 | SH | PP | Oui | 10 | 1000 | 10000 |  | Oui | 20 | 1000 | 20000 |  | Non | 0 | NA | NA |
| 063 | SH | PP | Oui | 38 | 1000 | 38000 |  | Oui | 1 | 1000 | 1000 |  | Non | 0 | NA | NA |
| 064 | SH | FAS | Oui | 14 | 1000 | 14000 |  | Non | 0 | NA | NA |  | Non | 0 | NA | NA |
| 065 | SH | PP | Oui | 42 | 1000 | 42000 |  | Non | 0 | NA | NA |  | Non | 0 | NA | NA |
| 068 | SH | FAS | Oui | 73 | 1000 | 73000 |  | Oui | 42 | 1000 | 42000 |  | Oui | 17 | 1000 | 17000 |
| 070 | SH | PP | Oui | 17 | 1000 | 17000 |  | Oui | 121 | 1000 | 121000 |  | Non | 0 | NA | NA |
| 072 | SH | PP | Oui | 25 | 1000 | 25000 |  | Oui | 33 | 1000 | 33000 |  | Oui | 37 | 1000 | 37000 |
| 075 | SH | FAS | Non | 0 | NA | NA |  | Non | 0 | NA | NA |  | Non | 0 | NA | NA |
| 076 | SH | PP | Non | 0 | NA | NA |  | Non | 0 | NA | NA |  | Non | 0 | NA | NA |
| 081 | SH | PP | Non | 0 | NA | NA |  | Oui | 37 | 500 | 18500 |  | Oui | 4 | 500 | 2000 |
| 089 | SH | PP | Oui | 22 | 1000 | 22000 |  | Non | 0 | NA | NA |  | Oui | 13 | 1000 | 13000 |
| 092 | SH | PP | Oui | 7 | 1000 | 7000 |  | Oui | 10 | 1000 | 10000 |  | Oui | 11 | 1000 | 11000 |
| 094 | SH | FAS | Non | 0 | NA | NA |  | Non | 0 | NA | NA |  | Non | 0 | NA | NA |
| 095 | SH | ITT | NA | NA | NA | NA |  | NA | NA | NA | NA |  | NA | NA | NA | NA |
| 097 | SH | ITT | NA | NA | NA | NA |  | NA | NA | NA | NA |  | NA | NA | NA | NA |
| 101 | SH | PP | Oui | 20 | 1000 | 20000 |  | Oui | 46 | 1000 | 46000 |  | Oui | 51 | 1000 | 51000 |
| 102 | SH | PP | Oui | 20 | 500 | 10000 |  | Oui | 8 | 500 | 4000 |  | Oui | 2 | 500 | 1000 |
| 106 | SH | PP | Non | 0 | NA | NA |  | Non | 0 | NA | NA |  | Non | 0 | NA | NA |
| 107 | SH | PP | Non | 0 | NA | NA |  | Non | 0 | NA | NA |  | Non | 0 | NA | NA |
| 110 | SH | PP | Non | 0 | NA | NA |  | Oui | 20 | 1000 | 20000 |  | Oui | 1 | 1000 | 1000 |
| 112 | SH | FAS | Non | 0 | NA | NA |  | NA | NA | NA | NA |  | Oui | 2 | 1000 | 2000 |
| 115 | SH | FAS | NA | NA | NA | NA |  | NA | NA | NA | NA |  | Oui | 28 | 1000 | 28000 |
| 117 | SH | FAS | Oui | 5 | 1000 | 5000 |  | Oui | 12 | 1000 | 12000 |  | NA | NA | NA | NA |
| 125 | SH | PP | Oui | 2 | 1000 | 2000 |  | Oui | 6 | 1000 | 6000 |  | Oui | 2 | 1000 | 2000 |
| 127 | SH | PP | Oui | 52 | 1000 | 52000 |  | Oui | 123 | 1000 | 123000 |  | Oui | 169 | 1000 | 169000 |
| 133 | SH | PP | Oui | 24 | 1000 | 24000 |  | Oui | 64 | 500 | 32000 |  | Oui | 128 | 500 | 64000 |
| 136 | SH | PP | Non | 0 | NA | NA |  | Oui | 32 | 500 | 16000 |  | Non | 0 | NA | NA |
| 139 | SH | PP | Oui | 10 | 1000 | 10000 |  | Oui | 5 | 1000 | 5000 |  | Oui | 10 | 1000 | 10000 |
| 141 | SH | PP | Oui | 1 | 1000 | 1000 |  | NA | NA | NA | NA |  | Oui | 2 | 1000 | 2000 |
| 143 | SH | PP | Oui | 1 | 1000 | 1000 |  | Oui | 1 | 1000 | 1000 |  | Oui | 1 | 1000 | 1000 |
| 149 | SH | PP | Non | 0 | NA | NA |  | Non | 0 | NA | NA |  | Non | 0 | NA | NA |
| 151 | SH | PP | Oui | 13 | 1000 | 13000 |  | Oui | 33 | 500 | 16500 |  | Oui | 76 | 500 | 38000 |
| 165 | SH | PP | Non | 0 | NA | NA |  | Non | 0 | NA | NA |  | Oui | 15 | 1000 | 15000 |
| 170 | SH | PP | Oui | 50 | 1000 | 50000 |  | Non | 0 | NA | NA |  | Non | 0 | NA | NA |
| 174 | SH | PP | Non | 0 | NA | NA |  | Non | 0 | NA | NA |  | Non | 0 | NA | NA |
| 175 | SH | PP | Non | 0 | NA | NA |  | Non | 0 | NA | NA |  | Non | 0 | NA | NA |
| 179 | SH | PP | Oui | 47 | 325 | 15275 |  | Oui | 90 | 325 | 29250 |  | Oui | 255 | 325 | 82875 |
| 180 | SH | PP | Non | 0 | NA | NA |  | Non | 0 | NA | NA |  | Non | 0 | NA | NA |
| 182 | SH | PP | Non | 0 | NA | NA |  | Non | 0 | NA | NA |  | Non | 0 | NA | NA |
| 183 | SH | PP | Oui | 10 | 1000 | 10000 |  | Non | 0 | NA | NA |  | Oui | 3 | 500 | 1500 |
| 185 | SH | PP | Non | 0 | NA | NA |  | Non | 0 | NA | NA |  | Non | 0 | NA | NA |
| 188 | SH | PP | Oui | 50 | 500 | 25000 |  | Oui | 30 | 500 | 15000 |  | Oui | 10 | 500 | 5000 |
| 189 | SH | PP | Oui | 24 | 1000 | 24000 |  | Non | 0 | NA | NA |  | Non | 0 | NA | NA |
| 191 | SH | PP | Oui | 10 | 1000 | 10000 |  | Oui | 16 | 1000 | 16000 |  | Non | 0 | NA | NA |
| 195 | SH | PP | Oui | 164 | 500 | 82000 |  | Oui | 58 | 500 | 29000 |  | Oui | 92 | 500 | 46000 |
| 196 | SH | PP | Oui | 75 | 1000 | 75000 |  | Oui | 273 | 1000 | 273000 |  | Oui | 234 | 1000 | 234000 |
| 198 | SH | PP | Oui | 72 | 1000 | 72000 |  | Oui | 120 | 1000 | 120000 |  | Oui | 200 | 1000 | 200000 |
| 200 | SH | FAS | Oui | 80 | 1000 | 80000 |  | Oui | 160 | 1000 | 160000 |  | Oui | 80 | 1000 | 80000 |
| 202 | SH | FAS | Oui | 72 | 1000 | 72000 |  | Oui | 40 | 1000 | 40000 |  | Oui | 400 | 1000 | 400000 |
| 204 | SH | PP | Non | 0 | NA | NA |  | Non | 0 | NA | NA |  | Oui | 80 | 500 | 40000 |
| 209 | SH | PP | Oui | 37 | 1000 | 37000 |  | Oui | 86 | 1000 | 86000 |  | Oui | 141 | 1000 | 141000 |
| 210 | SH | PP | Oui | 123 | 500 | 61500 |  | Oui | 204 | 1000 | 204000 |  | Oui | 183 | 1000 | 183000 |
| 217 | SH | PP | Non | 0 | NA | NA |  | Non | 0 | NA | NA |  | Non | 0 | NA | NA |
| 218 | SH | PP | Non | 0 | NA | NA |  | Non | 0 | NA | NA |  | Oui | 12 | 1000 | 12000 |
| 226 | SH | FAS | Non | 0 | NA | NA |  | Non | 0 | NA | NA |  | Oui | 20 | 1000 | 20000 |
| 228 | SH | FAS | Oui | 8 | 500 | 4000 |  | Non | 0 | NA | NA |  | Non | 0 | NA | NA |
| 233 | SH | PP | Oui | 58 | 500 | 29000 |  | Oui | 148 | 500 | 74000 |  | Oui | 136 | 500 | 68000 |
| 239 | SH | PP | Oui | 23 | 500 | 11500 |  | Oui | 4 | 500 | 2000 |  | Oui | 26 | 500 | 13000 |
| 240 | SH | PP | Non | 0 | NA | NA |  | Non | 0 | NA | NA |  | Non | 0 | NA | NA |
| 245 | SH | PP | Oui | 1 | 1000 | 1000 |  | Non | 0 | NA | NA |  | Non | 0 | NA | NA |
| 248 | SH | PP | Oui | 60 | 1000 | 60000 |  | Oui | 100 | 1000 | 100000 |  | Oui | 110 | 1000 | 110000 |
| 249 | SH | FAS | Oui | 13 | 1000 | 13000 |  | Non | 0 | NA | NA |  | Non | 0 | NA | NA |
| 250 | SH | PP | Non | 0 | NA | NA |  | Non | 0 | NA | NA |  | Non | 0 | NA | NA |
| 257 | SH | PP | Oui | 16 | 1000 | 16000 |  | Oui | 19 | 1000 | 19000 |  | Oui | 19 | 1000 | 19000 |
| 259 | SH | PP | Oui | 64 | 1000 | 64000 |  | Oui | 84 | 1000 | 84000 |  | Oui | 130 | 1000 | 130000 |
| 273 | SH | PP | Oui | 15 | 1000 | 15000 |  | Non | 0 | NA | NA |  | Non | 0 | NA | NA |
| 275 | SH | PP | Oui | 5 | 1000 | 5000 |  | Oui | 10 | 1000 | 10000 |  | Non | 0 | NA | NA |
| 279 | SH | FAS | Non | 0 | NA | NA |  | Non | 0 | NA | NA |  | Non | 0 | NA | NA |
| 280 | SH | PP | Non | 0 | NA | NA |  | Non | 0 | NA | NA |  | Non | 0 | NA | NA |
| 281 | SH | PP | Non | 0 | NA | NA |  | Non | 0 | NA | NA |  | Non | 0 | NA | NA |
| 282 | SH | FAS | Oui | 30 | 325 | 9750 |  | Non | 0 | NA | NA |  | Oui | 38 | 325 | 12350 |
| 290 | SH | FAS | Oui | 24 | 1000 | 24000 |  | Oui | 54 | 1000 | 54000 |  | Oui | 65 | 1000 | 65000 |
| 292 | SH | PP | Oui | 4 | 1000 | 4000 |  | Oui | 6 | 1000 | 6000 |  | Non | 0 | NA | NA |
| 298 | SH | PP | Oui | 59 | 1000 | 59000 |  | Non | 0 | NA | NA |  | Oui | 6 | 1000 | 6000 |
| 299 | SH | PP | Oui | 3 | 500 | 1500 |  | Oui | 7 | 500 | 3500 |  | Oui | 8 | 1000 | 8000 |
| 302 | SH | PP | Oui | 7 | 500 | 3500 |  | Oui | 5 | 500 | 2500 |  | Oui | 2 | 500 | 1000 |
| 303 | SH | PP | Oui | 2 | 1000 | 2000 |  | Oui | 60 | 1000 | 60000 |  | Oui | 3 | 1000 | 3000 |
| 305 | SH | PP | Oui | 13 | 1000 | 13000 |  | Oui | 5 | 1000 | 5000 |  | Non | 0 | NA | NA |
| 307 | SH | PP | Oui | 22 | 1000 | 22000 |  | Oui | 61 | 1000 | 61000 |  | Oui | 54 | 1000 | 54000 |
| 309 | SH | PP | Oui | 50 | 500 | 25000 |  | Oui | 60 | 500 | 30000 |  | Non | 0 | NA | NA |
| 311 | SH | FAS | Non | 0 | NA | NA |  | NA | NA | NA | NA |  | NA | NA | NA | NA |
| 314 | SH | PP | Non | 0 | NA | NA |  | Non | 0 | NA | NA |  | Non | 0 | NA | NA |
| 315 | SH | PP | Non | 0 | NA | NA |  | Non | 0 | NA | NA |  | Non | 0 | NA | NA |
| 318 | SH | PP | Non | 0 | NA | NA |  | Non | 0 | NA | NA |  | Non | 0 | NA | NA |
| 319 | SH | PP | Oui | 2 | 325 | 650 |  | Non | 0 | NA | NA |  | Non | 0 | NA | NA |
| 321 | SH | PP | Oui | 59 | 1000 | 59000 |  | Oui | 83 | 1000 | 83000 |  | Oui | 46 | 1000 | 46000 |
| 322 | SH | PP | Oui | 86 | 1000 | 86000 |  | Oui | 62 | 500 | 31000 |  | Oui | 83 | 500 | 41500 |
| 327 | SH | PP | Non | 0 | NA | NA |  | Non | 0 | NA | NA |  | Non | 0 | NA | NA |
| 328 | SH | PP | Non | 0 | NA | NA |  | Non | 0 | NA | NA |  | Non | 0 | NA | NA |
| 329 | SH | PP | Oui | 76 | 500 | 38000 |  | Oui | ND | ND | ND |  | Oui | ND | ND | ND |
| 330 | SH | PP | Oui | 186 | 500 | 93000 |  | Oui | 336 | 500 | 168000 |  | Oui | 500 | 1000 | 500000 |
| 334 | SH | PP | Non | 0 | NA | NA |  | Oui | 10 | 1000 | 10000 |  | Non | 0 | NA | NA |
| 335 | SH | PP | Oui | 50 | 1000 | 50000 |  | Oui | 65 | 1000 | 65000 |  | Non | 0 | NA | NA |
| 338 | SH | PP | Non | 0 | NA | NA |  | Non | 0 | NA | NA |  | Oui | 10 | 1000 | 10000 |
| 340 | SH | PP | Non | 0 | NA | NA |  | Non | 0 | NA | NA |  | Non | 0 | NA | NA |
| 341 | SH | FAS | Oui | 99 | 500 | 49500 |  | Oui | 172 | 500 | 86000 |  | Oui | 129 | 500 | 64500 |
| 342 | SH | FAS | Oui | 41 | 325 | 13325 |  | Oui | 108 | 325 | 35100 |  | Oui | 115 | 1000 | 115000 |
| 347 | SH | FAS | Non | 0 | NA | NA |  | Non | 0 | NA | NA |  | Non | 0 | NA | NA |
| 348 | SH | PP | Non | 0 | NA | NA |  | Non | 0 | NA | NA |  | Non | 0 | NA | NA |
| 349 | SH | PP | Non | 0 | NA | NA |  | Non | 0 | NA | NA |  | Non | 0 | NA | NA |
| 351 | SH | PP | Oui | 8 | 500 | 4000 |  | Oui | 4 | 500 | 2000 |  | Oui | 20 | 500 | 10000 |
| 358 | SH | FAS | Oui | 36 | 500 | 18000 |  | Oui | 83 | 1000 | 83000 |  | NA | NA | NA | NA |
| 360 | SH | PP | Oui | 26 | 1000 | 26000 |  | Oui | 19 | 1000 | 19000 |  | Oui | 43 | 1000 | 43000 |
| 362 | SH | FAS | Oui | 48 | 325 | 15600 |  | NA | NA | NA | NA |  | NA | NA | NA | NA |
| 366 | SH | ITT | Oui | 2 | 1000 | 2000 |  | Non | 0 | NA | NA |  | Non | 0 | NA | NA |
| 369 | SH | PP | Non | 0 | NA | NA |  | Non | 0 | NA | NA |  | Non | 0 | NA | NA |
| 373 | SH | PP | Non | 0 | NA | NA |  | Non | 0 | NA | NA |  | Non | 0 | NA | NA |
| 375 | SH | PP | Non | 0 | NA | NA |  | Non | 0 | NA | NA |  | Non | 0 | NA | NA |
| 382 | SH | FAS | Oui | 8 | 1000 | 8000 |  | Non | 0 | NA | NA |  | Oui | 12 | 1000 | 12000 |
| 383 | SH | PP | Oui | 28 | 1000 | 28000 |  | Oui | 80 | 1000 | 80000 |  | Oui | 44 | 1000 | 44000 |
| 385 | SH | PP | Non | 0 | NA | NA |  | Oui | 7 | 1000 | 7000 |  | Oui | 12 | 1000 | 12000 |
| 387 | SH | PP | Oui | 71 | 500 | 35500 |  | Oui | 40 | 500 | 20000 |  | Oui | 20 | 500 | 10000 |
| 391 | SH | ITT | NA | NA | NA | NA |  | NA | NA | NA | NA |  | NA | NA | NA | NA |
| 392 | SH | PP | Non | 0 | NA | NA |  | Oui | 25 | 500 | 12500 |  | Non | 0 | NA | NA |
| 393 | SH | PP | Non | 0 | NA | NA |  | Non | 0 | NA | NA |  | Non | 0 | NA | NA |
| 396 | SH | PP | Non | 0 | NA | NA |  | Oui | 42 | 1000 | 42000 |  | Oui | 90 | 1000 | 90000 |
| 397 | SH | PP | Non | 0 | NA | NA |  | Non | 0 | NA | NA |  | Non | 0 | NA | NA |
| 400 | SH | PP | Oui | 61 | 500 | 30500 |  | Oui | 91 | 500 | 45500 |  | Oui | 95 | 500 | 47500 |
| 001 | Control | PP | Oui | 36 | 1000 | 36000 |  | Oui | 24 | 1000 | 24000 |  | Oui | 48 | 1000 | 48000 |
| 004 | Control | PP | Oui | 16 | 1000 | 16000 |  | Non | 0 | NA | NA |  | Non | 0 | NA | NA |
| 005 | Control | PP | Oui | 60 | 500 | 30000 |  | Oui | 180 | 500 | 90000 |  | Oui | 220 | 1000 | 220000 |
| 007 | Control | PP | Oui | 60 | 1000 | 60000 |  | Oui | 120 | 1000 | 120000 |  | Oui | 240 | 1000 | 240000 |
| 010 | Control | PP | Oui | 65 | 500 | 32500 |  | Oui | 42 | 500 | 21000 |  | Non | 0 | NA | NA |
| 011 | Control | PP | Non | 0 | NA | NA |  | Non | 0 | NA | NA |  | Non | 0 | NA | NA |
| 013 | Control | PP | Non | 0 | NA | NA |  | Non | 0 | NA | NA |  | Non | 0 | NA | NA |
| 016 | Control | PP | Non | 0 | NA | NA |  | Non | 0 | NA | NA |  | Non | 0 | NA | NA |
| 017 | Control | PP | Non | 0 | NA | NA |  | Oui | 65 | 1000 | 65000 |  | Oui | 49 | 1000 | 49000 |
| 018 | Control | FAS | Oui | 76 | 500 | 38000 |  | Oui | 284 | 500 | 142000 |  | NA | NA | NA | NA |
| 021 | Control | PP | Oui | 8 | 500 | 4000 |  | Oui | 15 | 1000 | 15000 |  | Oui | 10 | 500 | 5000 |
| 024 | Control | PP | Non | 0 | NA | NA |  | Non | 0 | NA | NA |  | Oui | 3 | 1000 | 3000 |
| 027 | Control | PP | Oui | 1 | 500 | 500 |  | Non | 0 | NA | NA |  | Non | 0 | NA | NA |
| 028 | Control | PP | Non | 0 | NA | NA |  | NA | NA | NA | NA |  | Non | 0 | NA | NA |
| 030 | Control | PP | Oui | 31 | 500 | 15500 |  | Oui | 91 | 500 | 45500 |  | Oui | 90 | 500 | 45000 |
| 033 | Control | PP | Oui | 51 | 500 | 25500 |  | Oui | 100 | 500 | 50000 |  | Oui | 30 | 500 | 15000 |
| 038 | Control | PP | Oui | 25 | 1000 | 25000 |  | Oui | 21 | 1000 | 21000 |  | Oui | 32 | 1000 | 32000 |
| 039 | Control | PP | Oui | 18 | 1000 | 18000 |  | Oui | 28 | 1000 | 28000 |  | Oui | 33 | 1000 | 33000 |
| 042 | Control | PP | Oui | 20 | 1000 | 20000 |  | Oui | 29 | 1000 | 29000 |  | Oui | 29 | 1000 | 29000 |
| 043 | Control | PP | Oui | 19 | 1000 | 19000 |  | Oui | 23 | 1000 | 23000 |  | Oui | 32 | 1000 | 32000 |
| 045 | Control | PP | Oui | 2 | 1000 | 2000 |  | Non | 0 | NA | NA |  | Oui | 6 | 1000 | 6000 |
| 046 | Control | PP | Oui | 42 | 1000 | 42000 |  | Oui | 114 | 1000 | 114000 |  | Non | 0 | NA | NA |
| 049 | Control | PP | Oui | ND | 1000 | ND |  | Oui | ND | 1000 | ND |  | Oui | 88 | 1000 | 88000 |
| 051 | Control | PP | Oui | 16 | 1000 | 16000 |  | Oui | 58 | 1000 | 58000 |  | Oui | 80 | 1000 | 80000 |
| 054 | Control | PP | Oui | 80 | 1000 | 80000 |  | Oui | 80 | 1000 | 80000 |  | Oui | 80 | 1000 | 80000 |
| 056 | Control | PP | Oui | 50 | 1000 | 50000 |  | Oui | 140 | 1000 | 140000 |  | Oui | 180 | 1000 | 180000 |
| 059 | Control | PP | Non | 0 | NA | NA |  | Non | 0 | NA | NA |  | Non | 0 | NA | NA |
| 060 | Control | PP | Non | 0 | NA | NA |  | Non | 0 | NA | NA |  | Non | 0 | NA | NA |
| 061 | Control | PP | Non | 0 | NA | NA |  | Non | 0 | NA | NA |  | Non | 0 | NA | NA |
| 062 | Control | PP | Non | 0 | NA | NA |  | Oui | 3 | 1000 | 3000 |  | Non | 0 | NA | NA |
| 066 | Control | PP | Oui | 13 | 1000 | 13000 |  | Oui | 5 | 1000 | 5000 |  | Oui | 6 | 1000 | 6000 |
| 067 | Control | PP | Non | 0 | NA | NA |  | Non | 0 | NA | NA |  | Non | 0 | NA | NA |
| 069 | Control | PP | Oui | 1 | 1000 | 1000 |  | Oui | 10 | 1000 | 10000 |  | Oui | 24 | 1000 | 24000 |
| 071 | Control | PP | Oui | 17 | 1000 | 17000 |  | Oui | 66 | 1000 | 66000 |  | Oui | 131 | 1000 | 131000 |
| 073 | Control | FAS | Non | 0 | NA | NA |  | Non | 0 | NA | NA |  | Non | 0 | NA | NA |
| 074 | Control | PP | Non | 0 | NA | NA |  | Non | 0 | NA | NA |  | Non | 0 | NA | NA |
| 082 | Control | PP | Oui | 8 | 500 | 4000 |  | Non | 0 | NA | NA |  | Non | 0 | NA | NA |
| 085 | Control | FAS | Oui | 9 | 500 | 4500 |  | Oui | 8 | 1000 | 8000 |  | Non | 0 | NA | NA |
| 090 | Control | PP | Oui | 19 | 1000 | 19000 |  | Oui | 14 | 1000 | 14000 |  | Oui | 10 | 1000 | 10000 |
| 091 | Control | FAS | Oui | 22 | 1000 | 22000 |  | Oui | 5 | 1000 | 5000 |  | Oui | 13 | 1000 | 13000 |
| 093 | Control | FAS | Non | 0 | NA | NA |  | Non | 0 | NA | NA |  | Non | 0 | NA | NA |
| 096 | Control | PP | Non | 0 | NA | NA |  | Non | 0 | NA | NA |  | Non | 0 | NA | NA |
| 098 | Control | PP | Oui | 41 | 1000 | 41000 |  | Non | 0 | NA | NA |  | Non | 0 | NA | NA |
| 103 | Control | PP | Non | 0 | NA | NA |  | Non | 0 | NA | NA |  | Non | 0 | NA | NA |
| 104 | Control | PP | Non | 0 | NA | NA |  | Non | 0 | NA | NA |  | Non | 0 | NA | NA |
| 105 | Control | PP | Non | 0 | NA | NA |  | Non | 0 | NA | NA |  | Non | 0 | NA | NA |
| 108 | Control | FAS | Non | 0 | NA | NA |  | NA | NA | NA | NA |  | NA | NA | NA | NA |
| 109 | Control | FAS | Oui | 60 | 1000 | 60000 |  | NA | NA | NA | NA |  | Oui | 3 | 1000 | 3000 |
| 111 | Control | ITT | NA | NA | NA | NA |  | NA | NA | NA | NA |  | NA | NA | NA | NA |
| 113 | Control | PP | Oui | 2 | 1000 | 2000 |  | Non | 0 | NA | NA |  | Oui | 354 | 500 | 177000 |
| 114 | Control | FAS | Oui | 26 | 1000 | 26000 |  | Oui | 12 | 1000 | 12000 |  | Oui | 55 | 1000 | 55000 |
| 121 | Control | PP | Oui | 102 | 500 | 51000 |  | Oui | 248 | 500 | 124000 |  | Oui | 232 | 500 | 116000 |
| 126 | Control | PP | Oui | 64 | 1000 | 64000 |  | Oui | 90 | 1000 | 90000 |  | Oui | 150 | 1000 | 150000 |
| 128 | Control | FAS | Oui | 10 | 1000 | 10000 |  | Oui | 0 | 1000 | 0 |  | Oui | 60 | 1000 | 60000 |
| 134 | Control | PP | Oui | 64 | 1000 | 64000 |  | Oui | 32 | 500 | 16000 |  | Oui | 96 | 500 | 48000 |
| 135 | Control | PP | Non | 0 | NA | NA |  | Non | 0 | NA | NA |  | Oui | 64 | 500 | 32000 |
| 142 | Control | ITT | Oui | ND | 1000 | ND |  | NA | NA | NA | NA |  | NA | NA | NA | NA |
| 150 | Control | PP | Non | 0 | NA | NA |  | Non | 0 | NA | NA |  | Oui | 5 | 1000 | 5000 |
| 152 | Control | PP | Oui | 4 | 325 | 1300 |  | Oui | 1 | 1000 | 1000 |  | Oui | 3 | 1000 | 3000 |
| 153 | Control | FAS | Non | 0 | NA | NA |  | Non | 0 | NA | NA |  | Non | 0 | NA | NA |
| 166 | Control | PP | Non | 0 | NA | NA |  | Oui | 10 | 500 | 5000 |  | Non | 0 | NA | NA |
| 169 | Control | PP | Non | 0 | NA | NA |  | Non | 0 | NA | NA |  | Non | 0 | NA | NA |
| 171 | Control | PP | Oui | 65 | 1000 | 65000 |  | Oui | 48 | 1000 | 48000 |  | Oui | 208 | 1000 | 208000 |
| 173 | Control | FAS | Oui | 21 | 500 | 10500 |  | Oui | 21 | 500 | 10500 |  | NA | NA | NA | NA |
| 176 | Control | FAS | Oui | 23 | 1000 | 23000 |  | Oui | 4 | 1000 | 4000 |  | Oui | ND | 1000 | ND |
| 177 | Control | PP | Non | 0 | NA | NA |  | Non | 0 | NA | NA |  | Non | 0 | NA | NA |
| 178 | Control | FAS | Oui | 77 | 1000 | 77000 |  | Oui | 128 | 1000 | 128000 |  | Oui | 47 | 1000 | 47000 |
| 181 | Control | PP | Non | 0 | NA | NA |  | Non | 0 | NA | NA |  | Non | 0 | NA | NA |
| 184 | Control | PP | Non | 0 | NA | NA |  | Non | 0 | NA | NA |  | Non | 0 | NA | NA |
| 186 | Control | PP | Oui | 16 | 500 | 8000 |  | Non | 0 | NA | NA |  | Oui | 16 | 500 | 8000 |
| 187 | Control | FAS | Oui | 4 | 1000 | 4000 |  | NA | NA | NA | NA |  | NA | NA | NA | NA |
| 190 | Control | FAS | Oui | 16 | 1000 | 16000 |  | Non | 0 | NA | NA |  | Oui | 16 | 1000 | 16000 |
| 192 | Control | PP | Oui | 36 | 1000 | 36000 |  | Oui | 90 | 1000 | 90000 |  | Oui | 80 | 1000 | 80000 |
| 193 | Control | PP | Non | 0 | NA | NA |  | Non | 0 | NA | NA |  | Non | 0 | NA | NA |
| 194 | Control | PP | Non | 0 | NA | NA |  | Non | 0 | NA | NA |  | Oui | 24 | 1000 | 24000 |
| 197 | Control | PP | Non | 0 | NA | NA |  | Oui | 24 | 1000 | 24000 |  | Non | 0 | NA | NA |
| 199 | Control | PP | Oui | 40 | 1000 | 40000 |  | Oui | 40 | 1000 | 40000 |  | Oui | 72 | 1000 | 72000 |
| 201 | Control | PP | Oui | 40 | 1000 | 40000 |  | Oui | 160 | 1000 | 160000 |  | Oui | 240 | 1000 | 240000 |
| 203 | Control | PP | Oui | 10 | 500 | 5000 |  | Oui | 160 | 1000 | 160000 |  | Oui | 180 | 1000 | 180000 |
| 211 | Control | PP | Oui | 44 | 1000 | 44000 |  | Oui | 7 | 1000 | 7000 |  | Non | 0 | NA | NA |
| 212 | Control | PP | Oui | 10 | 500 | 5000 |  | Oui | 74 | 500 | 37000 |  | Oui | 92 | 500 | 46000 |
| 213 | Control | PP | Non | 0 | NA | NA |  | Non | 0 | NA | NA |  | Non | 0 | NA | NA |
| 221 | Control | ITT | NA | NA | NA | NA |  | NA | NA | NA | NA |  | NA | NA | NA | NA |
| 225 | Control | PP | Non | 0 | NA | NA |  | Non | 0 | NA | NA |  | Non | 0 | NA | NA |
| 227 | Control | FAS | Non | 0 | NA | NA |  | NA | NA | NA | NA |  | NA | NA | NA | NA |
| 229 | Control | FAS | Non | 0 | NA | NA |  | Oui | 8 | 1000 | 8000 |  | Oui | 90 | 1000 | 90000 |
| 230 | Control | FAS | Oui | 2 | 1000 | 2000 |  | NA | NA | NA | NA |  | NA | NA | NA | NA |
| 237 | Control | FAS | Oui | 95 | 500 | 47500 |  | NA | NA | NA | NA |  | NA | NA | NA | NA |
| 238 | Control | PP | Oui | 19 | 500 | 9500 |  | Oui | 6 | 500 | 3000 |  | Non | 0 | NA | NA |
| 246 | Control | PP | Non | 0 | NA | NA |  | Non | 0 | NA | NA |  | Oui | 10 | 1000 | 10000 |
| 247 | Control | PP | Oui | 13 | 1000 | 13000 |  | Oui | 19 | 1000 | 19000 |  | Oui | 5 | 1000 | 5000 |
| 251 | Control | PP | Non | 0 | NA | NA |  | Non | 0 | NA | NA |  | Non | 0 | NA | NA |
| 252 | Control | PP | Non | 0 | NA | NA |  | Non | 0 | NA | NA |  | Non | 0 | NA | NA |
| 258 | Control | PP | Oui | 38 | 1000 | 38000 |  | Oui | 1 | 1000 | 1000 |  | Oui | 7 | 1000 | 7000 |
| 260 | Control | PP | Oui | 9 | 1000 | 9000 |  | Oui | 10 | 1000 | 10000 |  | Oui | 11 | 1000 | 11000 |
| 261 | Control | ITT | NA | NA | NA | NA |  | NA | NA | NA | NA |  | NA | NA | NA | NA |
| 269 | Control | ITT | NA | NA | NA | NA |  | NA | NA | NA | NA |  | NA | NA | NA | NA |
| 274 | Control | PP | Non | 0 | NA | NA |  | Non | 0 | NA | NA |  | Non | 0 | NA | NA |
| 276 | Control | PP | Oui | 4 | 1000 | 4000 |  | Oui | 24 | 500 | 12000 |  | Oui | 20 | 500 | 10000 |
| 277 | Control | FAS | Oui | 4 | 500 | 2000 |  | Oui | 14 | 500 | 7000 |  | Oui | 22 | 500 | 11000 |
| 278 | Control | PP | Non | 0 | NA | NA |  | Non | 0 | NA | NA |  | Non | 0 | NA | NA |
| 283 | Control | PP | Oui | 63 | 1000 | 63000 |  | Oui | 30 | 1000 | 30000 |  | Non | 0 | NA | NA |
| 284 | Control | PP | Oui | 15 | 1000 | 15000 |  | Oui | 60 | 500 | 30000 |  | Oui | 50 | 500 | 25000 |
| 289 | Control | PP | Oui | 3 | 1000 | 3000 |  | Non | 0 | NA | NA |  | Oui | 3 | 1000 | 3000 |
| 291 | Control | PP | Non | 0 | NA | NA |  | Non | 0 | NA | NA |  | Non | 0 | NA | NA |
| 297 | Control | PP | Oui | 2 | 1000 | 2000 |  | Non | 0 | NA | NA |  | Non | 0 | NA | NA |
| 300 | Control | PP | Non | 0 | NA | NA |  | Non | 0 | NA | NA |  | Oui | 5 | 1000 | 5000 |
| 301 | Control | PP | Non | 0 | NA | NA |  | Non | 0 | NA | NA |  | Non | 0 | NA | NA |
| 304 | Control | PP | Oui | 1 | 1000 | 1000 |  | Non | 0 | NA | NA |  | Non | 0 | NA | NA |
| 306 | Control | PP | Oui | 52 | 1000 | 52000 |  | Oui | 85 | 1000 | 85000 |  | Oui | 100 | 1000 | 100000 |
| 308 | Control | PP | Non | 0 | NA | NA |  | Non | 0 | NA | NA |  | Non | 0 | NA | NA |
| 310 | Control | FAS | Oui | 75 | 300 | 22500 |  | Oui | 152 | 300 | 45600 |  | Oui | 182 | 300 | 54600 |
| 312 | Control | FAS | Oui | 5 | 500 | 2500 |  | Oui | 7 | 500 | 3500 |  | NA | NA | NA | NA |
| 313 | Control | PP | Oui | 16 | 1000 | 16000 |  | Oui | ND | ND | ND |  | Non | 0 | NA | NA |
| 317 | Control | PP | Oui | 8 | 1000 | 8000 |  | Oui | 28 | 1000 | 28000 |  | Non | 0 | NA | NA |
| 320 | Control | FAS | Oui | 2 | 1000 | 2000 |  | Oui | 12 | 1000 | 12000 |  | Oui | 25 | 1000 | 25000 |
| 323 | Control | FAS | Oui | 19 | 1000 | 19000 |  | NA | NA | NA | NA |  | NA | NA | NA | NA |
| 324 | Control | FAS | Oui | 4 | 1000 | 4000 |  | Oui | 2 | 1000 | 2000 |  | NA | NA | NA | NA |
| 325 | Control | PP | Non | 0 | NA | NA |  | Non | 0 | NA | NA |  | Non | 0 | NA | NA |
| 326 | Control | PP | Non | 0 | NA | NA |  | Non | 0 | NA | NA |  | Non | 0 | NA | NA |
| 331 | Control | PP | Non | 0 | NA | NA |  | Non | 0 | NA | NA |  | Non | 0 | NA | NA |
| 333 | Control | PP | Non | 0 | NA | NA |  | Non | 0 | NA | NA |  | Non | 0 | NA | NA |
| 336 | Control | PP | Non | 0 | NA | NA |  | Non | 0 | NA | NA |  | Non | 0 | NA | NA |
| 337 | Control | PP | Oui | 41 | 1000 | 41000 |  | Oui | 6 | 1000 | 6000 |  | Non | 0 | NA | NA |
| 339 | Control | PP | Non | 0 | NA | NA |  | Non | 0 | NA | NA |  | Non | 0 | NA | NA |
| 343 | Control | PP | Oui | 5 | 1000 | 5000 |  | Oui | 10 | 1000 | 10000 |  | Oui | 5 | 1000 | 5000 |
| 344 | Control | FAS | Oui | 60 | 1000 | 60000 |  | Oui | 20 | 500 | 10000 |  | Non | 0 | NA | NA |
| 345 | Control | PP | Oui | 41 | 1000 | 41000 |  | Non | 0 | NA | NA |  | Non | 0 | NA | NA |
| 346 | Control | PP | Non | 0 | NA | NA |  | Non | 0 | NA | NA |  | Non | 0 | NA | NA |
| 350 | Control | ITT | NA | NA | NA | NA |  | NA | NA | NA | NA |  | NA | NA | NA | NA |
| 352 | Control | PP | Non | 0 | NA | NA |  | Oui | ND | ND | ND |  | Oui | ND | ND | ND |
| 357 | Control | PP | Non | 0 | NA | NA |  | Non | 0 | NA | NA |  | Oui | 4 | 1000 | 4000 |
| 359 | Control | PP | Non | 0 | NA | NA |  | Non | 0 | NA | NA |  | Non | 0 | NA | NA |
| 361 | Control | PP | Non | 0 | NA | NA |  | Non | 0 | NA | NA |  | Non | 0 | NA | NA |
| 365 | Control | ITT | NA | NA | NA | NA |  | Oui | 84 | 1000 | 84000 |  | Oui | 71 | 1000 | 71000 |
| 370 | Control | PP | Non | 0 | NA | NA |  | Non | 0 | NA | NA |  | Non | 0 | NA | NA |
| 371 | Control | PP | Oui | 6 | 1000 | 6000 |  | Oui | 18 | 1000 | 18000 |  | Oui | 25 | 1000 | 25000 |
| 374 | Control | PP | Non | 0 | NA | NA |  | Oui | 240 | 1000 | 240000 |  | Oui | 150 | 1000 | 150000 |
| 381 | Control | PP | Oui | 11 | 1000 | 11000 |  | Non | 0 | NA | NA |  | Oui | 2 | 1000 | 2000 |
| 384 | Control | PP | Oui | 14 | 1000 | 14000 |  | Oui | 3 | 1000 | 3000 |  | Oui | 36 | 1000 | 36000 |
| 386 | Control | PP | Non | 0 | NA | NA |  | Non | 0 | NA | NA |  | Non | 0 | NA | NA |
| 388 | Control | PP | Non | 0 | NA | NA |  | Oui | 3 | 500 | 1500 |  | Oui | 20 | 500 | 10000 |
| 389 | Control | FAS | Non | 0 | NA | NA |  | Non | 0 | NA | NA |  | Non | 0 | NA | NA |
| 390 | Control | FAS | Oui | 8 | 1000 | 8000 |  | Oui | 21 | 1000 | 21000 |  | Oui | 52 | 1000 | 52000 |
| 394 | Control | PP | Oui | 2 | 1000 | 2000 |  | Non | 0 | NA | NA |  | Oui | 2 | 1000 | 2000 |
| 395 | Control | PP | Oui | 5 | 400 | 2000 |  | Non | 0 | NA | NA |  | Non | 0 | NA | NA |
| 398 | Control | FAS | Oui | 5 | 1000 | 5000 |  | Non | 0 | NA | NA |  | Non | 0 | NA | NA |
| 399 | Control | PP | Oui | 5 | 1000 | 5000 |  | Oui | 19 | 1000 | 19000 |  | Non | 0 | NA | NA |

C = Consultation; control = hylan G-F 20; D = Day; FAS = Full Analysis Set; ITT = Intention-to-Treat; NA = not available/applicable; SH = sodium hyaluronate;
PP = Per Protocol.
