# Supplementary material for: A single intra-articular injection of 2.0% non-chemically modified sodium hyaluronate vs 0.8% hylan G-F 20 in the treatment of symptomatic knee osteoarthritis: A 6-month, multicenter, randomized, controlled non-inferiority trial
Source: PLoS One. 2019 Dec 10;14(12):e0226007. doi: 10.1371/journal.pone.0226007 (PMC6903764; doi:10.1371/journal.pone.0226007)
Supplement: S15 Table — (DOCX) [file pone.0226007.s020.docx]

**S15 Table. Treatment-emergent adverse events (as number of patients) by System Organ Class (injected patients).**

| **MedDRA System Organ Class** MedDRA Preferred Term | | **SH n (%)** | **Control n (%)** | ***P*** |
| --- | --- | --- | --- | --- |
| Infections and infestations | | 6 (4.2) | 5 (3.4) | 0.8† |
|  | Tonsillitis | - | 1 (0.7) |  |
|  | Bronchitis | 3 (2.1) | 3 (2.1) |  |
|  | Intervertebral discitis | 1 (0.7) | - |  |
|  | Infective exacerbation of chronic obstructive airways disease | - | 1 (0.7) |  |
|  | Respiratory tract infection | 1 (0.7) | - |  |
|  | Nasopharyngitis | 1 (0.7) | - |  |
| Metabolism and nutrition disorders | | 1 (0.7) | - | 0.5† |
|  | Vitamin D deficiency | 1 (0.7) | - |  |
| Psychiatric disorders | | 1 (0.7) | 1 (0.7) | >0.9† |
|  | Anxiety | - | 1 (0.7) |  |
|  | Depression | 1 (0.7) | - |  |
| Nervous system disorders | | 3 (2.1) | - | 0.1† |
|  | Cerebrovascular accident | 1 (0.7) | - |  |
|  | Sciatica | 2 (1.4) | - |  |
| Vascular disorders | | - | 1 (0.7) | >0.9† |
|  | Hypertension | - | 1 (0.7) |  |
| Respiratory, thoracic and mediastinal disorders | | - | 1 (0.7) | >0.9† |
|  | Interstitial lung disease | - | 1 (0.7) |  |
| Gastrointestinal disorders | | - | 2 (1.4) | 0.5† |
|  | Diarrhoea | - | 1 (0.7) |  |
|  | Toothache | - | 1 (0.7) |  |
| Musculoskeletal and connective tissue disorders | | 17 (12.0) | 25 (17.1) | 0.2† |
|  | Arthralgia (LLT: coxalgia) | - | 1 (0.7) |  |
|  | Arthralgia [contralateral knee, not studied] | 1 (0.7) | 1 (0.7) |  |
|  | Arthralgia [studied knee or knees] | 11 (7.7) | 9 (6.2) |  |
|  | Spinal osteoarthritis | 1 (0.7) | 3 (2.1) |  |
|  | Chondrocalcinosis | 1 (0.7) | - |  |
|  | Back pain (LLT: loin pain) | 4 (2.8) | 6 (4.1) |  |
|  | Musculoskeletal pain | 1 (0.7) | - |  |
|  | Bone pain | - | 1 (0.7) |  |
|  | Joint effusion [contralateral knee] | - | 1 (0.7) |  |
|  | Metatarsalgia | - | 1 (0.7) |  |
|  | Osteoarthritis (LLT: osteoarthritis flare up) | 1 (0.7) | 5 (3.4) |  |
|  | Periarthritis | 1 (0.7) | - |  |
| General disorders and administration site conditions | | 15 (10.6) | 23 (15.8) | 0.2† |
|  | Pre-existing condition improved | 1 (0.7) | - |  |
|  | Pain | 1 (0.7) | 2 (1.4) |  |
|  | Injection site joint pain | 11 (7.7) | 16 (11.0) |  |
|  | Injection site joint effusion | 2 (1.4) | 2 (1.4) |  |
|  | Fatigue | 1 (0.7) | - |  |
|  | Pyrexia | - | 1 (0.7) |  |
|  | Injection site joint inflammation | 4 (2.8) | 6 (4.1) |  |
|  | Influenza like illness | - | 1 (0.7) |  |
| Investigations | | 1 (0.7) | - | 0.5† |
|  | Arthroscopy [contralateral knee, not studied] | 1 (0.7) | - |  |
| Injury, poisoning and procedural complications | | 3 (2.1) | - | 0.1† |
|  | Fall | 1 (0.7) | - |  |
|  | Ligament sprain | 1 (0.7) | - |  |
|  | Femur fracture | 1 (0.7) | - |  |
|  | Synovial rupture | 1 (0.7) | - |  |
| Surgical and medical procedures | | 2 (1.4) | 5 (3.4) | 0.4† |
|  | Meniscus removal [studied knee] | - | 1 (0.7) |  |
|  | Knee arthroplasty [studied knee] | - | 1 (0.7) |  |
|  | Bunion operation | 1 (0.7) | 1 (0.7) |  |
|  | Inguinal hernia repair | - | 1 (0.7) |  |
|  | Drug therapy changed | - | 1 (0.7) |  |
|  | Prophylaxis against gastrointestinal ulcer | 1 (0.7) | 1 (0.7) |  |
| Any types of adverse event | | 39 (27.5) | 51 (34.9) |  |
| Patients injected | | 142 (100.0) | 146 (100.0) |  |

† Fisher’s exact test.

Control = hylan G-F 20; LLT = Lowest Level Term; MedDRA = Medical Dictionary for Regulatory Activities; SH = sodium hyaluronate.
