# Supplementary material for: A single intra-articular injection of 2.0% non-chemically modified sodium hyaluronate vs 0.8% hylan G-F 20 in the treatment of symptomatic knee osteoarthritis: A 6-month, multicenter, randomized, controlled non-inferiority trial
Source: PLoS One. 2019 Dec 10;14(12):e0226007. doi: 10.1371/journal.pone.0226007 (PMC6903764; doi:10.1371/journal.pone.0226007)
Supplement: S16 Table — (DOCX) [file pone.0226007.s021.docx]

**S16 Table. Individual adverse events (Intention-to-Treat population).**

| **Patient** | **Group** | **C2** | **Start** | **End** | **Description** | **Seriousness** | **Intensity** | **Causality** | **Action taken** | **Outcome** |
| --- | --- | --- | --- | --- | --- | --- | --- | --- | --- | --- |
| 019 | SH | D0 | NA | NA | Tramadol discontinued | NA | NA | NA | TOPALGIC | NA |
| 019 | SH | D0 | D154 | D178 | Chondrocalcinosis of left and right knees | Not serious | Severe | Excluded | Colchicine, NSAID; these failed, so cortisone 15 mg/day. Hospitalized 3 weeks for this. | Ongoing |
| 023 | SH | D0 | D158 | D169 | Bronchitis | Not serious | Moderate | Excluded | AUGMENTIN | Recovery |
| 052 | SH | D0 | NA | NA | Right knee pain | NA | NA | NA | NAPROSYN | NA |
| 089 | SH | D0 | NA | NA | Right knee pain | NA | NA | NA | VOLTAREN | NA |
| 089 | SH | D0 | D86 | D90 | Bronchitis | Not serious | Moderate | Excluded | SOLUPRED 20 x 2 | Recovery |
| 089 | SH | D0 | D28 | D32 | Lumbar pain | Not serious | Moderate | Excluded | APRANAX + omeprazole | Recovery |
| 089 | SH | D0 | D28 | D32 | Gastroprotection | NA | NA | NA | Omeprazole | NA |
| 097 | SH | D6 | D4 | NA | Left knee pain at night [non-treatment emergent] | Not serious | Moderate | Excluded | Left the trial; puncture + cortivazol (ALTIM) infiltration given on 24/09/2011 | Ongoing |
| 097 | SH | D6 | D4 | NA | Voluminous water on the left knee [non-treatment emergent] | Not serious | Moderate | Excluded | Left the trial; puncture + cortivazol (ALTIM) infiltration given on 24/09/2011 | Ongoing |
| 101 | SH | D0 | NA | NA | Vitamin D deficiency | NA | NA | NA | ZYMAD | NA |
| 115 | SH | D2 | D254 | D254 | Arthroscopy of left knee | Not serious | Moderate | Excluded | Arthroscopy | Recovery |
| 117 | SH | D0 | NA | NA | Left knee pain | NA | NA | NA | ALTIM | NA |
| 125 | SH | D0 | D140 | D144 | Rhinoplasty | Not serious | Mild | Excluded | BECOTIDE | Recovery |
| 125 | SH | D0 | D44 | D55 | Rhinoplasty | Not serious | Mild | Excluded | BECOTIDE CODOLIPRANE | Recovery |
| 141 | SH | D2 | NA | NA | Stroke | NA | NA | NA | ASPEGIC | NA |
| 179 | SH | D0 | NA | NA | Pain in both knees | NA | NA | NA | ARCOXIA | NA |
| 182 | SH | D0 | D68 | D98 | Left cruralgia | Not serious | Moderate | Excluded | None | Ongoing |
| 183 | SH | D0 | D147 | D153 | Rupture of synovial cyst in left knee | Not serious | Moderate | Unlikely | Application of ice + acetaminophen | Ongoing |
| 185 | SH | D0 | D79 | NA | Depression | Not serious | Mild | Excluded | SEROPLEX | Ongoing |
| 195 | SH | D0 | D111 | D118 | Bronchitis | Not serious | Moderate | Excluded | Amoxicillin + SOLUPRED | Recovery |
| 202 | SH | D1 | NA | NA | Knee pain | NA | NA | NA | ALTIM | NA |
| 210 | SH | D0 | D126 | D136 | Spinal osteoarthritis | Not serious | Mild | Excluded | ARCOXIA | Recovery |
| 210 | SH | D0 | D57 | D66 | Ankle sprain | Not serious | Mild | Excluded | ARCOXIA | Recovery |
| 210 | SH | D0 | D126 | D126 | Pain in right scapulohumeral area | Not serious | Mild | Excluded | ALTIM | Recovery |
| 218 | SH | D4 | D167 | D173 | Spondylodiscitis | Not serious | Severe | Excluded | Antibiotics + hospitalization | Ongoing |
| 218 | SH | D4 | NA | NA | Pain [non-treatment emergent] | NA | NA | NA | ASPEGIC | NA |
| 226 | SH | D4 | NA | NA | Pain in both knees | NA | NA | NA | VOLTAREN | NA |
| 228 | SH | D7 | D49 | NA | Right lumbar sciatic pain | Not serious | Moderate | Excluded | NSAID | Ongoing |
| 282 | SH | D4 | NA | NA | Knee pain | NA | NA | NA | BIPROFENID 100 LP | NA |
| 282 | SH | D4 | NA | NA | Lumbar pain [non-treatment emergent] | NA | NA | NA | PROFENID 100 | NA |
| 282 | SH | D4 | NA | NA | Lumbar pain | NA | NA | NA | BIPROFENID 100 LP | NA |
| 282 | SH | D4 | NA | NA | Congestive episode in knee [non-treatment emergent] | NA | NA | NA | PROFENID 100 | NA |
| 290 | SH | D2 | NA | NA | Knee pain | NA | NA | NA | Tramadol | NA |
| 303 | SH | D0 | NA | NA | Pain | NA | NA | NA | NAPROSYN | NA |
| 303 | SH | D0 | NA | NA | Pain in right scapula | NA | NA | NA | ALTIM | NA |
| 315 | SH | D3 | D31 | D31 | Fall on public road | Serious | Severe | Excluded | NA | Hospitalization |
| 315 | SH | D3 | D31 | D37 | Fracture of the femur | Serious | Severe | Excluded | Surgery | Hospitalization |
| 348 | SH | D0 | NA | NA | Pain once in cinema | NA | NA | NA | PROPOFAN | NA |
| 358 | SH | D0 | NA | NA | Right knee pain | NA | NA | NA | ALTIM | NA |
| 360 | SH | D0 | NA | NA | Right knee pain | NA | NA | NA | CELEBREX | NA |
| 360 | SH | D0 | D53 | NA | Lumbar pain | Not serious | Moderate | Excluded | DOLIPRANE | Ongoing |
| 369 | SH | D2 | D13 | NA | Lumbar pain | Not serious | Moderate | Excluded | CARTREX | Ongoing |
| 383 | SH | D0 | D35 | D48 | Acute right knee inflammation | Not serious | Mild | Excluded | Ketoprofen | Recovery |
| 385 | SH | D0 | D1 | D4 | General fatigue | Not serious | Moderate | Unlikely | None | Recovery |
| 392 | SH | D0 | NA | NA | Hallux valgus surgery on right leg, 29/7/2012 | NA | NA | NA | Acetaminophen | NA |
| 396 | SH | D0 | NA | NA | Pain in both knees | NA | NA | NA | ASPIRIN | NA |
| 396 | SH | D0 | D5 | D17 | Respiratory infection | Not serious | Mild | Excluded | Antibiotics + SOLUPRED | Recovery |
| 016 | Control | D0 | D139 | D144 | Bronchitis | Not serious | Moderate | Excluded | SOLUPRED DOLIPRANE | Recovery |
| 016 | Control | D0 | NA | NA | Lumbar pain | NA | NA | NA | DOLIPRANE | NA |
| 016 | Control | D0 | D87 | D88 | Toothache | Not serious | Severe | Excluded | DAFALGAN CODEINE | Recovery |
| 017 | Control | D0 | NA | NA | Knee pain | NA | NA | NA | BIPROFENID | NA |
| 017 | Control | D0 | NA | NA | Ulcer prophylaxis | NA | NA | NA | LANZOR | NA |
| 018 | Control | D0 | NA | NA | Knee pain | NA | NA | NA | ALTIM | NA |
| 018 | Control | D0 | NA | NA | Degenerative lumbar pain | NA | NA | NA | FELDENE | NA |
| 021 | Control | D0 | NA | NA | Knee pain | NA | NA | NA | BIPROFENID | NA |
| 028 | Control | D0 | D205 | D206 | Lumbar pain | Not serious | Moderate | Excluded | Aceclofenac 100 mg | Recovery |
| 060 | Control | D0 | NA | NA | Arterial hypertension | NA | NA | NA | LERCAN | NA |
| 061 | Control | D0 | D14 | NA | Spinal osteoarthritis | Not serious | Mild | Excluded | NSAID | Ongoing |
| 082 | Control | D2 | NA | NA | Influenza | NA | NA | NA | Acetaminophen | NA |
| 090 | Control | D0 | D2 | D29 | Metatarsal pain | Not serious | Moderate | Excluded | APRANAX then ketoprofen | Ongoing |
| 091 | Control | D0 | NA | NA | Ear nose throat infection | NA | NA | NA | FLANID G | NA |
| 091 | Control | D0 | D183 | D183 | Effusion in left knee | Not serious | Moderate | Excluded | Hexatrione | Recovery |
| 096 | Control | D0 | D19 | D47 | Effusion in left knee | Not serious | Moderate | Excluded | Puncture on 29/02/2012 | Ongoing |
| 103 | Control | D0 | D127 | D127 | Surgery for inguinal hernia | Not serious | Moderate | Excluded | Surgery | Recovery |
| 103 | Control | D0 | NA | NA | Osteoporosis | NA | NA | NA | ACLASTA | NA |
| 105 | Control | D0 | D25 | D40 | Bronchitis | Not serious | Mild | Excluded | SOLUPRED + BIOCALYPTOL | Recovery |
| 108 | Control | D0 | NA | NA | Right knee pain | NA | NA | NA | Left the trial; ibuprofen | NA |
| 108 | Control | D0 | NA | NA | Major acute inflammation in the studied knee | NA | NA | NA | Left the trial; cortivazol (ALTIM) administered on 25/05/2012 | NA |
| 113 | Control | D3 | D146 | D146 | Hallux valgus surgery on right leg | Not serious | Moderate | Excluded | Surgery | Recovery |
| 113 | Control | D3 | D153 | D153 | Hallux valgus surgery on left leg | Not serious | Moderate | Excluded | None | Recovery |
| 114 | Control | D5 | D180 | D182 | Effusion in right knee | Not serious | Moderate | Excluded | SYNOVIAL injection 1 ampoule after puncture | Recovery |
| 128 | Control | D0 | D156 | NA | Lumbar pain | Not serious | Moderate | Excluded | TOPALGIC MIOREL | Ongoing |
| 150 | Control | D1 | D19 | D22 | Bronchitis | Not serious | Mild | Excluded | Prednisolone | Recovery |
| 166 | Control | D2 | D173 | D178 | Pain | Not serious | Moderate | Excluded | VOLTAREN | Recovery |
| 171 | Control | D0 | NA | NA | Spinal pain | NA | NA | NA | Piroxicam | NA |
| 173 | Control | D0 | NA | NA | Knee pain | NA | NA | NA | VOLTAREN | NA |
| 176 | Control | D0 | D97 | D97 | Arthroscopy for left meniscus fracture revealed incidentally in left knee | Not serious | Moderate | Excluded | Partial meniscectomy | Ongoing |
| 176 | Control | D0 | NA | NA | Aggravation of knee pain | NA | NA | NA | VOLTAREN | NA |
| 225 | Control | D3 | D168 | D175 | Lumbar pain | Not serious | Moderate | Excluded | CELEBREX | Recovery |
| 229 | Control | D2 | NA | NA | Chronic obstructive pulmonary disease | NA | NA | NA | Amoxicillin | NA |
| 230 | Control | D2 | D56 | D56 | Acute water on the right knee, punctured and infiltrated | Not serious | Severe | Excluded | Left the trial; puncture + infiltration of steroid | Ongoing |
| 247 | Control | D1 | D4 | D166 | Pain in left knee | Not serious | Moderate | Excluded | CELEBREX | Recovery |
| 269 | Control | D5 | NA | NA | Oral anticoagulants and effusion [non-treatment emergent] | NA | NA | NA | Left the trial | NA |
| 276 | Control | D1 | D4 | NA | Pain in the hip | Not serious | Moderate | Excluded | CELEBREX | Ongoing |
| 277 | Control | D4 | D92 | NA | Lumbar osteoarthritis | Not serious | Moderate | Excluded | CHONDROSULF | Ongoing |
| 310 | Control | D3 | D200 | D200 | Right knee prosthesis | Serious | Mild | Excluded | Surgery | Hospitalization |
| 310 | Control | D3 | D72 | D100 | Acute right knee osteoarthritis | Not serious | Moderate | Excluded | CORTANCYL | Recovery |
| 317 | Control | D0 | NA | NA | Knee pain | NA | NA | NA | PROXALYOC | NA |
| 320 | Control | D0 | NA | NA | Right knee pain | NA | NA | NA | BREXIN | NA |
| 320 | Control | D0 | D43 | D48 | Lumbar pain | Not serious | Moderate | Excluded | PROFENID 100 | Recovery |
| 323 | Control | D0 | NA | NA | Right knee pain | NA | NA | NA | ALTIM | NA |
| 324 | Control | D0 | NA | NA | Knee pain | NA | NA | NA | VOLTAREN | NA |
| 344 | Control | D3 | D102 | D108 | Diarrhea | Not serious | Moderate | Excluded | None | Recovery |
| 344 | Control | D3 | NA | NA | Lumbar pain | Not serious | Severe | Excluded | CLARADOL CAFFEINE | Ongoing |
| 350 | Control | D1 | D23 | D42 | Isolated viral pyrexia | Serious | Moderate | Unlikely | Left the trial; antibiotic | Hospitalization |
| 365 | Control | D1 | D69 | 105 | Interstitial lung disease | Not serious | Mild | Excluded | Prednisone | Recovery |
| 381 | Control | D0 | NA | NA | Anxiety | NA | NA | NA | Zolpidem | NA |
| 390 | Control | D0 | D22 | NA | Generalized pain | Not serious | Moderate | Excluded | NSAID + analgesics | Ongoing |

C = Consultation; control = hylan G-F 20; D = Day; NA = not available/applicable; NSAID = non-steroidal anti-inflammatory drug; SH = sodium hyaluronate.
