# Supplementary material for: A single intra-articular injection of 2.0% non-chemically modified sodium hyaluronate vs 0.8% hylan G-F 20 in the treatment of symptomatic knee osteoarthritis: A 6-month, multicenter, randomized, controlled non-inferiority trial
Source: PLoS One. 2019 Dec 10;14(12):e0226007. doi: 10.1371/journal.pone.0226007 (PMC6903764; doi:10.1371/journal.pone.0226007)
Supplement: S17 Table — (DOCX) [file pone.0226007.s022.docx]

**S17 Table. Individual local post injection reactions (injected patients).**

| **Patient** | **Group** | **Dataset** | **Pain** | **Inflammation** | **Effusion** | **Puncture** | **Septic arthritis** | **Resolution** | **Hospitalization** |
| --- | --- | --- | --- | --- | --- | --- | --- | --- | --- |
| 002 | SH | PP | No | No | No | No | No | NA | No |
| 003 | SH | PP | No | No | No | No | No | NA | No |
| 006 | SH | PP | No | No | No | No | No | NA | No |
| 008 | SH | PP | No | No | No | No | No | NA | No |
| 009 | SH | PP | No | No | No | No | No | NA | No |
| 012 | SH | PP | No | No | No | No | No | NA | No |
| 014 | SH | PP | No | No | No | No | No | NA | No |
| 015 | SH | PP | Yes | No | No | No | No | Yes, spontaneously | No |
| 019 | SH | FAS | Yes | No | No | No | No | Yes, spontaneously | No |
| 020 | SH | PP | No | No | No | No | No | NA | No |
| 022 | SH | PP | No | No | No | No | No | NA | No |
| 023 | SH | PP | No | No | No | No | No | NA | No |
| 025 | SH | FAS | No | No | No | No | No | NA | No |
| 026 | SH | FAS | No | No | No | No | No | NA | No |
| 029 | SH | FAS | No | No | No | No | No | NA | No |
| 037 | SH | PP | No | No | No | No | No | NA | No |
| 040 | SH | PP | No | No | No | No | No | NA | No |
| 041 | SH | PP | No | No | No | No | No | NA | No |
| 044 | SH | PP | No | No | No | No | No | NA | No |
| 048 | SH | PP | No | No | No | No | No | NA | No |
| 050 | SH | PP | No | No | No | No | No | NA | No |
| 052 | SH | PP | No | No | No | No | No | NA | No |
| 053 | SH | PP | No | No | No | No | No | NA | No |
| 055 | SH | PP | No | No | No | No | No | NA | No |
| 057 | SH | PP | No | No | No | No | No | NA | No |
| 058 | SH | PP | No | No | No | No | No | NA | No |
| 063 | SH | PP | No | No | No | No | No | NA | No |
| 064 | SH | FAS | No | No | No | No | No | NA | No |
| 065 | SH | PP | Yes | No | No | No | No | Yes, spontaneously | No |
| 068 | SH | FAS | No | No | No | No | No | NA | No |
| 070 | SH | PP | No | No | No | No | No | NA | No |
| 072 | SH | PP | No | No | No | No | No | NA | No |
| 075 | SH | FAS | No | No | No | No | No | NA | No |
| 076 | SH | PP | No | No | No | No | No | NA | No |
| 081 | SH | PP | No | No | No | No | No | NA | No |
| 089 | SH | PP | No | No | No | No | No | NA | No |
| 092 | SH | PP | No | No | No | No | No | NA | No |
| 094 | SH | FAS | No | No | No | No | No | NA | No |
| 095 | SH | ITT | NA | NA | NA | NA | NA | NA | NA |
| 101 | SH | PP | No | No | No | No | No | NA | No |
| 102 | SH | PP | No | No | No | No | No | NA | No |
| 106 | SH | PP | No | No | No | No | No | NA | No |
| 107 | SH | PP | No | No | No | No | No | NA | No |
| 110 | SH | PP | No | No | No | No | No | NA | No |
| 112 | SH | FAS | No | No | No | No | No | NA | No |
| 115 | SH | FAS | NA | NA | NA | NA | NA | NA | NA |
| 117 | SH | FAS | No | No | No | No | No | NA | No |
| 125 | SH | PP | No | No | No | No | No | NA | No |
| 127 | SH | PP | No | No | No | No | No | NA | No |
| 133 | SH | PP | Yes | No | No | No | No | Yes, spontaneously | No |
| 136 | SH | PP | No | No | No | No | No | NA | No |
| 139 | SH | PP | No | No | No | No | No | NA | No |
| 141 | SH | PP | No | No | No | No | No | NA | No |
| 143 | SH | PP | No | No | No | No | No | NA | No |
| 149 | SH | PP | No | No | No | No | No | NA | No |
| 151 | SH | PP | No | No | No | No | No | NA | No |
| 165 | SH | PP | No | No | No | No | No | NA | No |
| 170 | SH | PP | No | No | No | No | No | NA | No |
| 174 | SH | PP | Yes | No | No | No | No | Yes, spontaneously | No |
| 175 | SH | PP | No | No | No | No | No | NA | No |
| 179 | SH | PP | No | No | No | No | No | NA | No |
| 180 | SH | PP | No | No | No | No | No | NA | No |
| 182 | SH | PP | No | No | No | No | No | NA | No |
| 183 | SH | PP | Yes | Yes | No | No | No | Yes, spontaneously | No |
| 185 | SH | PP | No | No | No | No | No | NA | No |
| 188 | SH | PP | No | No | No | No | No | NA | No |
| 189 | SH | PP | No | No | No | No | No | NA | No |
| 191 | SH | PP | No | No | No | No | No | NA | No |
| 195 | SH | PP | No | No | No | No | No | NA | No |
| 196 | SH | PP | No | No | No | No | No | NA | No |
| 198 | SH | PP | Yes | Yes | No | No | No | Yes, spontaneously | No |
| 200 | SH | FAS | No | No | No | No | No | NA | No |
| 202 | SH | FAS | No | No | No | No | No | NA | No |
| 204 | SH | PP | No | No | No | No | No | NA | No |
| 209 | SH | PP | No | No | No | No | No | NA | No |
| 210 | SH | PP | No | No | No | No | No | NA | No |
| 217 | SH | PP | No | No | No | No | No | NA | No |
| 218 | SH | PP | No | No | No | No | No | NA | No |
| 226 | SH | FAS | No | Yes | Yes | No | No | Yes, spontaneously | No |
| 228 | SH | FAS | Yes | No | Yes | No | No | Yes, spontaneously | No |
| 233 | SH | PP | No | No | No | No | No | NA | No |
| 239 | SH | PP | No | No | No | No | No | NA | No |
| 240 | SH | PP | No | No | No | No | No | NA | No |
| 245 | SH | PP | No | No | No | No | No | NA | No |
| 248 | SH | PP | No | No | No | No | No | NA | No |
| 249 | SH | FAS | No | No | No | No | No | NA | No |
| 250 | SH | PP | No | No | No | No | No | NA | No |
| 257 | SH | PP | No | No | No | No | No | NA | No |
| 259 | SH | PP | No | No | No | No | No | NA | No |
| 273 | SH | PP | No | No | No | No | No | NA | No |
| 275 | SH | PP | No | No | No | No | No | NA | No |
| 279 | SH | FAS | No | No | No | No | No | NA | No |
| 280 | SH | PP | No | No | No | No | No | NA | No |
| 281 | SH | PP | No | No | No | No | No | NA | No |
| 282 | SH | FAS | No | No | No | No | No | NA | No |
| 290 | SH | FAS | No | No | No | No | No | NA | No |
| 292 | SH | PP | No | No | No | No | No | NA | No |
| 298 | SH | PP | No | No | No | No | No | NA | No |
| 299 | SH | PP | No | No | No | No | No | NA | No |
| 302 | SH | PP | No | No | No | No | No | NA | No |
| 303 | SH | PP | No | No | No | No | No | NA | No |
| 305 | SH | PP | No | No | No | No | No | NA | No |
| 307 | SH | PP | No | No | No | No | No | NA | No |
| 309 | SH | PP | No | No | No | No | No | NA | No |
| 311 | SH | FAS | No | No | No | No | No | NA | No |
| 314 | SH | PP | No | No | No | No | No | NA | No |
| 315 | SH | PP | No | No | No | No | No | NA | No |
| 318 | SH | PP | No | No | No | No | No | NA | No |
| 319 | SH | PP | No | No | No | No | No | NA | No |
| 321 | SH | PP | No | No | No | No | No | NA | No |
| 322 | SH | PP | No | No | No | No | No | NA | No |
| 327 | SH | PP | No | No | No | No | No | NA | No |
| 328 | SH | PP | No | No | No | No | No | NA | No |
| 329 | SH | PP | No | No | No | No | No | NA | No |
| 330 | SH | PP | No | No | No | No | No | NA | No |
| 334 | SH | PP | No | No | No | No | No | NA | No |
| 335 | SH | PP | No | No | No | No | No | NA | No |
| 338 | SH | PP | No | No | No | No | No | NA | No |
| 340 | SH | PP | No | No | No | No | No | NA | No |
| 341 | SH | FAS | No | No | No | No | No | NA | No |
| 342 | SH | FAS | No | No | No | No | No | NA | No |
| 347 | SH | FAS | No | No | No | No | No | NA | No |
| 348 | SH | PP | No | No | No | No | No | NA | No |
| 349 | SH | PP | No | No | No | No | No | NA | No |
| 351 | SH | PP | No | No | No | No | No | NA | No |
| 358 | SH | FAS | No | No | No | No | No | NA | No |
| 360 | SH | PP | No | No | No | No | No | NA | No |
| 362 | SH | FAS | Yes | Yes | No | No | No | Yes, spontaneously | No |
| 366 | SH | ITT | No | No | No | No | No | NA | No |
| 369 | SH | PP | No | No | No | No | No | NA | No |
| 373 | SH | PP | No | No | No | No | No | NA | No |
| 375 | SH | PP | No | No | No | No | No | NA | No |
| 382 | SH | FAS | No | No | No | No | No | NA | No |
| 383 | SH | PP | No | No | No | No | No | NA | No |
| 385 | SH | PP | No | No | No | No | No | NA | No |
| 387 | SH | PP | No | No | No | No | No | NA | No |
| 391 | SH | ITT | NA | NA | NA | NA | NA | NA | NA |
| 392 | SH | PP | No | No | No | No | No | NA | No |
| 393 | SH | PP | Yes | No | No | No | No | Yes, spontaneously | No |
| 396 | SH | PP | No | No | No | No | No | NA | No |
| 397 | SH | PP | Yes | No | No | No | No | Yes, spontaneously | No |
| 400 | SH | PP | No | No | No | No | No | NA | No |
| 001 | Control | PP | No | No | No | No | No | NA | No |
| 004 | Control | PP | No | No | No | No | No | NA | No |
| 005 | Control | PP | No | No | No | No | No | NA | No |
| 007 | Control | PP | No | No | No | No | No | NA | No |
| 010 | Control | PP | No | No | No | No | No | NA | No |
| 011 | Control | PP | No | No | No | No | No | NA | No |
| 013 | Control | PP | No | No | No | No | No | NA | No |
| 016 | Control | PP | No | No | No | No | No | NA | No |
| 017 | Control | PP | Yes | No | No | No | No | Yes, spontaneously | No |
| 018 | Control | FAS | Yes | No | No | No | No | Yes, spontaneously | No |
| 021 | Control | PP | No | No | No | No | No | NA | No |
| 024 | Control | PP | No | Yes | No | No | No | Yes, spontaneously | No |
| 027 | Control | PP | No | No | No | No | No | NA | No |
| 028 | Control | PP | No | No | No | No | No | NA | No |
| 030 | Control | PP | Yes | No | No | No | No | Yes, spontaneously | No |
| 033 | Control | PP | No | No | No | No | No | NA | No |
| 038 | Control | PP | No | No | No | No | No | NA | No |
| 039 | Control | PP | No | No | No | No | No | NA | No |
| 042 | Control | PP | No | No | No | No | No | NA | No |
| 043 | Control | PP | No | No | No | No | No | NA | No |
| 045 | Control | PP | No | No | No | No | No | NA | No |
| 046 | Control | PP | No | No | No | No | No | NA | No |
| 049 | Control | PP | No | No | No | No | No | NA | No |
| 051 | Control | PP | No | No | No | No | No | NA | No |
| 054 | Control | PP | No | No | No | No | No | NA | No |
| 056 | Control | PP | Yes | No | No | No | No | Yes, spontaneously | No |
| 059 | Control | PP | No | No | No | No | No | NA | No |
| 060 | Control | PP | No | No | No | No | No | NA | No |
| 061 | Control | PP | No | No | No | No | No | NA | No |
| 062 | Control | PP | No | No | No | No | No | NA | No |
| 066 | Control | PP | Yes | No | No | No | No | Yes, spontaneously | No |
| 067 | Control | PP | No | No | No | No | No | NA | No |
| 069 | Control | PP | Yes | Yes | No | No | No | Yes, spontaneously | No |
| 071 | Control | PP | No | No | No | No | No | NA | No |
| 073 | Control | FAS | No | No | No | No | No | NA | No |
| 074 | Control | PP | No | No | No | No | No | NA | No |
| 082 | Control | PP | No | No | No | No | No | NA | No |
| 085 | Control | FAS | No | No | No | No | No | NA | No |
| 090 | Control | PP | Yes | No | No | No | No | Yes, spontaneously | No |
| 091 | Control | FAS | No | No | No | No | No | NA | No |
| 093 | Control | FAS | No | No | No | No | No | NA | No |
| 096 | Control | PP | No | No | Yes | Yes | No | Yes, after puncture | No |
| 098 | Control | PP | No | No | No | No | No | NA | No |
| 103 | Control | PP | No | No | No | No | No | NA | No |
| 104 | Control | PP | No | No | No | No | No | NA | No |
| 105 | Control | PP | No | No | No | No | No | NA | No |
| 108 | Control | FAS | No | No | No | No | No | NA | No |
| 109 | Control | FAS | No | No | No | No | No | NA | No |
| 111 | Control | ITT | NA | NA | NA | NA | NA | NA | NA |
| 113 | Control | PP | No | No | No | No | No | NA | No |
| 114 | Control | FAS | No | No | No | No | No | NA | No |
| 121 | Control | PP | Yes | No | No | No | No | Yes, spontaneous | No |
| 126 | Control | PP | No | No | No | No | No | NA | No |
| 128 | Control | FAS | No | No | No | No | No | NA | No |
| 134 | Control | PP | No | No | No | No | No | NA | No |
| 135 | Control | PP | No | No | No | No | No | NA | No |
| 142 | Control | ITT | No | No | No | No | No | NA | No |
| 150 | Control | PP | No | No | No | No | No | NA | No |
| 152 | Control | PP | Yes | Yes | No | No | No | Yes, spontaneous | No |
| 153 | Control | FAS | No | No | No | No | No | NA | No |
| 166 | Control | PP | No | No | No | No | No | NA | No |
| 169 | Control | PP | No | No | No | No | No | NA | No |
| 171 | Control | PP | No | No | No | No | No | NA | No |
| 173 | Control | FAS | Yes | No | No | No | No | Yes, spontaneous | No |
| 176 | Control | FAS | No | No | No | No | No | NA | No |
| 177 | Control | PP | No | No | No | No | No | NA | No |
| 178 | Control | FAS | No | No | No | No | No | NA | No |
| 181 | Control | PP | Yes | No | No | No | No | Yes, spontaneous | No |
| 184 | Control | PP | Yes | Yes | No | No | No | Yes, spontaneous | No |
| 186 | Control | PP | No | No | No | No | No | NA | No |
| 187 | Control | FAS | No | No | No | No | No | NA | No |
| 190 | Control | FAS | No | No | No | No | No | NA | No |
| 192 | Control | PP | No | No | No | No | No | NA | No |
| 193 | Control | PP | No | No | No | No | No | NA | No |
| 194 | Control | PP | No | No | No | No | No | NA | No |
| 197 | Control | PP | No | No | No | No | No | NA | No |
| 199 | Control | PP | No | No | No | No | No | NA | No |
| 201 | Control | PP | Yes | No | No | No | No | Yes, spontaneous | No |
| 203 | Control | PP | Yes | No | No | No | No | Yes, spontaneous | No |
| 211 | Control | PP | No | No | No | No | No | NA | No |
| 212 | Control | PP | No | No | No | No | No | NA | No |
| 213 | Control | PP | No | No | No | No | No | NA | No |
| 221 | Control | ITT | NA | NA | NA | NA | NA | NA | NA |
| 225 | Control | PP | No | No | No | No | No | NA | No |
| 227 | Control | FAS | No | No | No | No | No | NA | No |
| 229 | Control | FAS | No | No | No | No | No | NA | No |
| 230 | Control | FAS | No | No | No | No | No | NA | No |
| 237 | Control | FAS | No | No | No | No | No | NA | No |
| 238 | Control | PP | No | No | No | No | No | NA | No |
| 246 | Control | PP | No | No | No | No | No | NA | No |
| 247 | Control | PP | No | No | No | No | No | NA | No |
| 251 | Control | PP | No | No | No | No | No | NA | No |
| 252 | Control | PP | No | No | No | No | No | NA | No |
| 258 | Control | PP | No | No | No | No | No | NA | No |
| 260 | Control | PP | No | No | No | No | No | NA | No |
| 274 | Control | PP | No | No | No | No | No | NA | No |
| 276 | Control | PP | No | No | No | No | No | NA | No |
| 277 | Control | FAS | No | Yes | No | No | No | Yes, spontaneous | No |
| 278 | Control | PP | Yes | No | No | No | No | Yes, spontaneous | No |
| 283 | Control | PP | No | No | No | No | No | NA | No |
| 284 | Control | PP | No | No | No | No | No | NA | No |
| 289 | Control | PP | No | No | No | No | No | NA | No |
| 291 | Control | PP | No | No | No | No | No | NA | No |
| 297 | Control | PP | No | No | No | No | No | NA | No |
| 300 | Control | PP | No | No | No | No | No | NA | No |
| 301 | Control | PP | No | No | No | No | No | NA | No |
| 304 | Control | PP | No | No | No | No | No | NA | No |
| 306 | Control | PP | No | No | No | No | No | NA | No |
| 308 | Control | PP | No | No | No | No | No | NA | No |
| 310 | Control | FAS | No | No | No | No | No | NA | No |
| 312 | Control | FAS | No | No | No | No | No | NA | No |
| 313 | Control | PP | No | No | No | No | No | NA | No |
| 317 | Control | PP | No | No | No | No | No | NA | No |
| 320 | Control | FAS | No | No | No | No | No | NA | No |
| 323 | Control | FAS | No | No | No | No | No | NA | No |
| 324 | Control | FAS | No | No | No | No | No | NA | No |
| 325 | Control | PP | No | No | No | No | No | NA | No |
| 326 | Control | PP | No | No | No | No | No | NA | No |
| 331 | Control | PP | No | No | No | No | No | NA | No |
| 333 | Control | PP | No | No | No | No | No | NA | No |
| 336 | Control | PP | No | No | No | No | No | NA | No |
| 337 | Control | PP | No | No | No | No | No | NA | No |
| 339 | Control | PP | No | No | No | No | No | NA | No |
| 343 | Control | PP | No | No | No | No | No | NA | No |
| 344 | Control | FAS | No | No | No | No | No | NA | No |
| 345 | Control | PP | No | No | No | No | No | NA | No |
| 346 | Control | PP | No | No | No | No | No | NA | No |
| 350 | Control | ITT | NA | NA | NA | NA | NA | NA | NA |
| 352 | Control | PP | No | No | No | No | No | NA | No |
| 357 | Control | PP | No | No | No | No | No | NA | No |
| 359 | Control | PP | No | No | No | No | No | NA | No |
| 361 | Control | PP | No | No | No | No | No | NA | No |
| 365 | Control | ITT | NA | NA | NA | NA | NA | NA | NA |
| 370 | Control | PP | No | No | No | No | No | NA | No |
| 371 | Control | PP | No | No | No | No | No | NA | No |
| 374 | Control | PP | No | No | No | No | No | NA | No |
| 381 | Control | PP | No | No | No | No | No | NA | No |
| 384 | Control | PP | No | No | No | No | No | NA | No |
| 386 | Control | PP | No | No | No | No | No | NA | No |
| 388 | Control | PP | No | No | No | No | No | NA | No |
| 389 | Control | FAS | No | No | No | No | No | NA | No |
| 390 | Control | FAS | No | No | No | No | No | NA | No |
| 394 | Control | PP | No | No | No | No | No | NA | No |
| 395 | Control | PP | Yes | Yes | Yes | No | No | Yes, spontaneous | No |
| 398 | Control | FAS | No | No | No | No | No | NA | No |
| 399 | Control | PP | No | No | No | No | No | NA | No |

Control = hylan G-F 20; FAS= Full Analysis Set; ITT = Intention-to-Treat; NA = not available/applicable; PP = Per Protocol; SH = sodium hyaluronate.
